# A cavity-reduced prefusion RSV F bivalent vaccine elicits durable and protective immunity

**DOI:** 10.1038/s44321-026-00511-8

**Published:** 2026-08-29

**Authors:** Lijie Liu, Mengrong Yan, Ruoxu Liang, Qingxin Wu, Changbin Qu, Huiqi Wu, Zhi Liao, Huiyu Chen, Jingjing Zou, Zeyun Sun, Dongyu Niu, Liyuan Wu, Li Zhao, Jialing Dai, Jialun Shen, Long Zhang, Wei Zhang, Wei Peng

**Affiliations:** 1 State Key Laboratory of Respiratory Disease, the First Affiliated Hospital of Guangzhou Medical University, Guangzhou Medical University, Guangzhou, China; 2 Guangzhou National Laboratory, Guangzhou, China; 3 Division of Life Sciences and Medicine, University of Science and Technology of China, Hefei, China; 4 School of Pharmaceutical Science, University of South China, Hengyang, China; 5 School of Chemistry and Chemical Engineering, University of South China, Hengyang, China; 6 Institute of Pathogenic Biology, Hengyang Medical College, Hunan Provincial Key Laboratory for Special Pathogens Prevention and Control, University of South China, Hengyang, China; 7 KingMed School of Laboratory Medicine, Guangzhou Medical University, Guangzhou, China; 8 State Key Laboratory of Medicinal Chemical Biology, Nankai University, Tianjin, China

**Keywords:** Immunology, Microbiology, Virology & Host Pathogen Interaction

## Abstract

Respiratory syncytial virus (RSV) remains a major cause of severe respiratory disease, and stabilization of the prefusion (preF) conformation of the F glycoprotein is central for vaccine development. Here, we report a structure-guided engineering strategy that enhances preF stability by reducing the hydrophobic cavity within the trimeric F protein. Targeted modifications at metastability-associated sites generated RVF-88, a disulfide-free stabilized preF immunogen that preserves key neutralizing epitopes, including antigenic site Ø, while exhibiting improved structural integrity and long-term storage stability. Formulated as an unadjuvanted bivalent vaccine, RVF-88 elicited potent neutralizing antibody responses and durable immune protection lasting up to 5 months in mice. Vaccination also protected both mice and cotton rats against RSV challenge. Structural analyses confirmed the intended cavity-reduction design, revealing a reduced apical hydrophobic cavity volume and surface area while maintaining the prefusion architecture. Together, these findings establish hydrophobic cavity reduction as a rational strategy for stabilizing prefusion RSV F and provide a promising next-generation vaccine candidate with improved stability and immunogenicity for further clinical development.

## Synopsis

A region-prioritized structural optimization strategy enabled development of a low-mutation, disulfide-free RSV prefusion F immunogen with preserved antigenicity and protective activity in vivo.

- A low-mutation, disulfide-free RSV preF immunogen (RVF-88) was identified through region-prioritized structural optimization and experimental screening.
- Reduced apical hydrophobic cavity volume and surface area were observed in RVF-88 crystal structures, consistent with the intended cavity-reduction design strategy.
- A bivalent RVF-88 vaccine elicited strong RSV A and RSV B neutralizing responses in mice and cotton rats without adjuvant.
- Compatibility with CHO-based production workflows and favorable biophysical properties were maintained in RSV A- and B-lineage RVF-88 antigens.

## Abstract (web summary)

A region-prioritized structural optimization strategy enabled development of a low-mutation, disulfide-free RSV prefusion F immunogen with preserved antigenicity and protective activity in vivo.

## Graphical abstract

**Figure:**
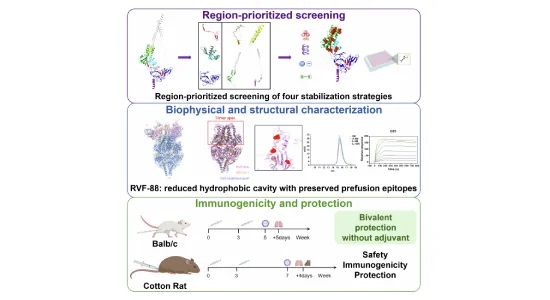

The paper explained **Problem**

Respiratory syncytial virus (RSV) remains a major cause of severe lower respiratory tract disease in infants, older adults, and immunocompromised individuals worldwide. Stabilization of the RSV fusion (F) glycoprotein in its prefusion (preF) conformation is central to vaccine development because key prefusion-specific epitopes are targets of potent neutralizing antibodies. However, the prefusion RSV F trimer is intrinsically metastable and readily undergoes conformational rearrangement. Therefore, alternative prefusion stabilization strategies that preserve key neutralizing epitopes while maintaining favorable antigenic and biophysical properties remain of considerable interest for RSV vaccine development.

**Results**

In this study, we developed a region-prioritized, structure-guided optimization strategy to engineer a disulfide-free prefusion RSV F immunogen termed RVF-88. Comparative structural analysis was first used to identify metastability-associated regions for focused mutational screening across multiple stabilization modalities, including hydrophobic cavity filling, proline substitution, electrostatic tuning, and disulfide engineering. Experimental screening subsequently identified a comparatively low-mutation lead construct that retained prefusion-specific antigenicity, showed improved resilience to thermal and storage stress, and preserved key neutralizing epitopes. Structural analyses demonstrated that RVF-88 adopts a prefusion-like trimeric architecture with reduced apical hydrophobic cavity volume and surface area consistent with the intended cavity-reduction design.

A bivalent vaccine formulation incorporating RSV A- and B-lineage RVF-88 variants elicited strong neutralizing antibody responses in mice and cotton rats without adjuvant and significantly reduced pulmonary viral burden following RSV challenge. In addition, the engineered antigens were compatible with CHO-based production workflows relevant to translational development.

**Impact**

This study establishes an alternative structure-guided strategy for engineering stabilized RSV prefusion F antigens without relying on exogenous disulfide locks. The findings demonstrate that comparatively low-mutation designs can preserve antigenic integrity, maintain favorable biophysical properties, and induce protective immunity in vivo. More broadly, the region-prioritized optimization strategy described here may inform future engineering of stabilized prefusion viral glycoproteins.

## Introduction

Respiratory syncytial virus (RSV) is a major viral pathogen responsible for severe lower respiratory tract infections worldwide, particularly in infants, older adults, and individuals with underlying medical conditions. According to the global disease burden estimates, RSV is associated with ~3.6 million hospitalizations and ~100,000 deaths annually among children under five years of age, with nearly 97% of these occurring in low- and middle-income countries, highlighting the urgent need for durable and scalable preventive strategies. Correspondingly, a 2019 global analysis estimated about 33 million RSV-associated acute lower respiratory tract infection episodes and over 100,000 deaths in this age group, further emphasizing the critical public health challenge posed by RSV (Li et al, 2022).

The fusion (F) glycoprotein on the RSV surface mediates viral entry into host cells and is the principal target of neutralizing antibodies in human sera, making it a prime candidate for vaccine development (McLellan et al, 2013b; Ngwuta et al, 2015). RSV F adopts two principal conformational states: a metastable prefusion (preF) form and a stable postfusion (postF) form. While most potent neutralizing antibodies recognize epitopes unique to the preF conformation, this state is metastable and readily undergoes irreversible rearrangement into the postF form (McLellan et al, 2013b; Ngwuta et al, 2015). Stabilizing the F protein in its prefusion conformation preserves key prefusion-specific epitopes associated with potent neutralizing antibody responses (McLellan et al, 2013a; Ngwuta et al, 2015). Because these epitopes are critical for eliciting protective immune responses, prefusion stabilization has emerged as a central strategic objective in RSV vaccine development. Epidemiological evidence confirms that RSV subgroups A and B co-circulate each season, with their relative dominance varying by region and year (Cantú-Flores et al, 2022; Langedijk et al, 2024). Although the RSV F protein is highly conserved (> 90% sequence identity) between the two subgroups (McLellan et al, 2013c; Oraby et al, 2025) and RSV F-based immunogens can induce cross-neutralizing responses, sequence differences remain concentrated in key prefusion epitopes, particularly site Ø, which can influence subgroup-specific antigenic recognition (Oraby et al, 2025; Hashimoto and Hosoya, 2017). Therefore, despite RSV A and B generally being considered to belong to the same serotype, a bivalent design may still be advantageous for achieving more balanced protection against both subgroups.

Structure-guided antigen design has greatly advanced RSV vaccine development. The landmark DS-Cav1 construct, which introduced an engineered disulfide bond and cavity-filling mutations, demonstrated the immunological superiority of the preF conformation (McLellan et al, 2013a) but suffered from low yield and conformational drift toward postF during storage, complicating large-scale manufacturing (Flynn et al, 2016; Zhang et al, 2018). Subsequent efforts have yielded rationally stabilized variants, such as uncleaved prefusion-closed (UFC) trimers that maintain the closed preF conformation without disulfide crosslinks, tyrosine-crosslinked forms with extended stability, and variants incorporating multiple proline substitutions (e.g., preF7P) that achieve high expression and robust immunogenicity (Lee et al, 2024; Gidwani et al, 2024; Li et al, 2025; Huang et al, 2025). Recently, the clinical success of two recombinant prefusion F-based vaccines (AREXVY and ABRYSVO) has established a benchmark for RSV vaccine design. Building on this benchmark, improving antigen stability has emerged as a key priority, as intrinsically stable antigens can support durable vaccine performance and mitigate loss of prefusion epitope integrity and immunogenicity during storage.

In broad terms, previously reported RSV preF designs have relied on one or more established stabilization principles, including covalent locking, local rigidification, cavity filling, and electrostatic optimization (McLellan et al, 2013a; Krarup et al, 2015; Huang et al, 2025). Here, we used comparative structural analysis to establish a structure-guided optimization strategy for RSV F, in which selected regions were prioritized for focused mutational evaluation across multiple stabilization mechanisms. Using this strategy, we identified a lead prefusion-stabilized variant with improved prefusion antigenicity, favorable expression, and enhanced resilience to thermal and storage stress, supporting a practical low-mutation design for bivalent vaccine development.

## Results

### Structure-guided regional prioritization and focused mutational design of prefusion RSV F antigens

**Figure 1:**
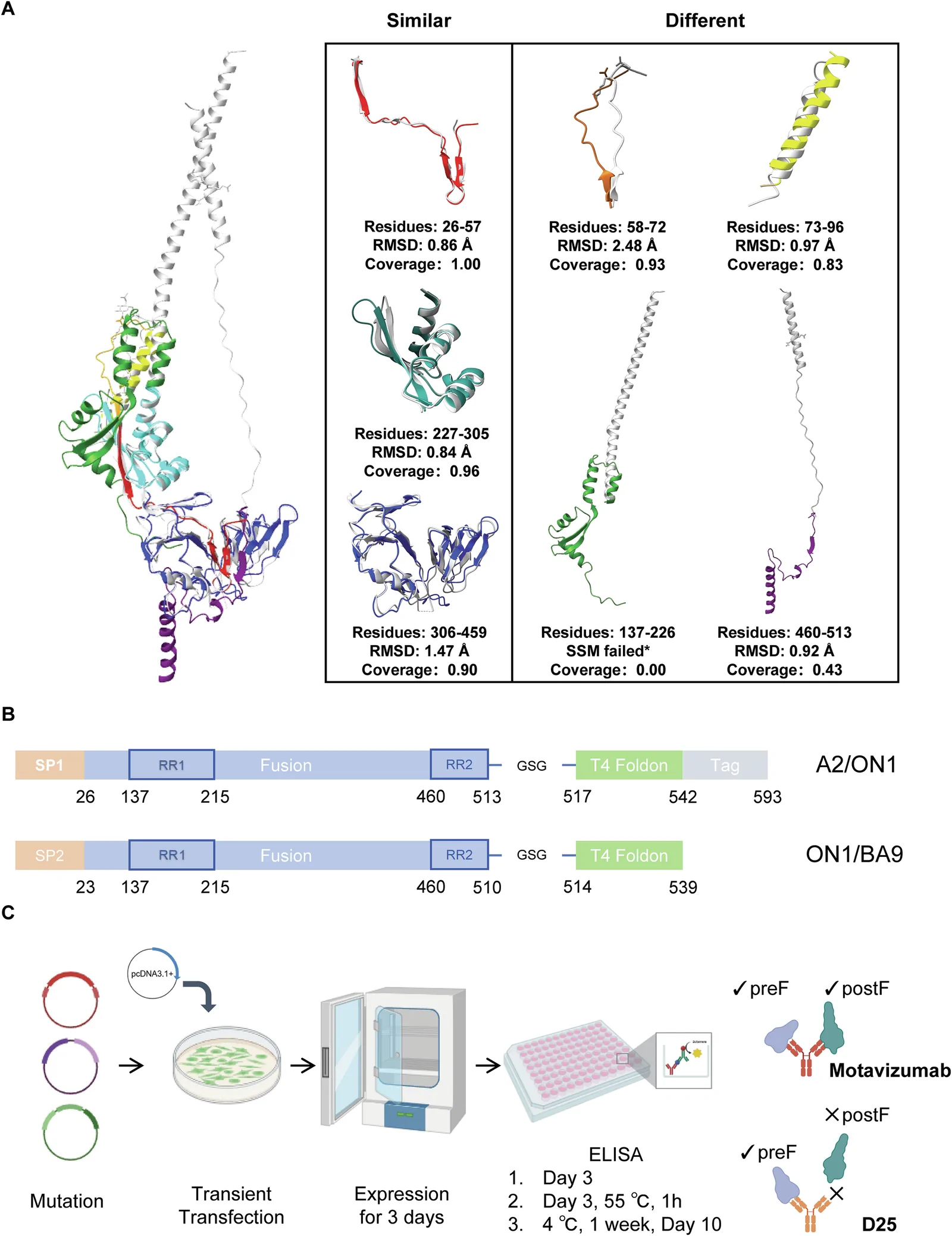
Structure-guided design of prefusion-stabilizing RSV F mutations and screening workflow of RSV F protein variants. (**A**) Structure-based classification of monomeric preF and postF protein models. Left: superimposed structures of preF (PDB: 4JHW) and postF (PDB: 3RRR). The postfusion conformation is shown in light gray throughout. Each prefusion segment is shown in a distinct color: residues 26–57 (red), 58–72 (chocolate), 73–96 (yellow), 137–226 (forest green), 227–305 (teal), 306–459 (blue), and 460–513 (purple). Segments were classified as similar or different based on SSM-based superposition statistics including coverage, backbone RMSD, and quality factor Q; classification criteria and complete alignment statistics are provided in Appendix Table S1. (**B**) Schematic of RSV antigen design. The antigen is derived from the ectodomain (aa 26–513, light blue) of F proteins from the RSV A2 strain and from representative RSV-A and RSV-B lineages (ON1 and BA9, respectively), and includes the RR1 region (residues 137–215) and RR2 region (residues 460–513) (boxed in blue). The N-terminus contains either the native signal peptide (residues 1–25) or the IL-2 signal peptide (residues 1–22). The C-terminus is fused via a GSG linker to a trimerization domain from T4 fibritin (foldon, shown in green), followed by optional His-tag and StrepII-tag sequences for purification. (**C**) Screening workflow of RSV F protein variants. pcDNA3.1 vectors encoding the designed F protein constructs were transiently transfected into HEK293T cells. After 3 days of culture, supernatants from 96-well plates were collected. Portions were subjected to heat treatment at 55 °C for 1 h or stored at 4 °C for 7 days (corresponding to day 10 post-transfection). Binding to motavizumab (recognizing both prefusion and postfusion conformations) and D25 (specific for the prefusion conformation) was measured by sandwich ELISA.

To enable structure-guided engineering of a prefusion-stabilized recombinant F glycoprotein, the crystal structures of the prefusion F ectodomain in complex with a neutralizing antibody fragment (PDB: 4JHW) and the postfusion F protein (PDB: 3RRR) were used as reference templates for comparative analysis of the ectodomain (McLellan et al, 2013b, 2011). Drawing on differences between the prefusion and postfusion conformations and their spatial placement along the ectodomain, we partitioned  the structurally resolved regions of the F-protein ectodomain sequence into seven segments (Fig. 1A). Based on structural superposition by secondary-structure matching (SSM), residues 26–57, 227–305, and 306–459 were classified as similar between the prefusion and postfusion conformations, while residues 58–72, 73–96, 137–226, and 460–513 showed substantial conformational divergence and were classified as different (Appendix Table S1). Rather than treating these differences as descriptive structural features alone, we used this organization to prioritize conformationally divergent regions, together with adjacent supporting framework, for focused mutational design.

Using this region-prioritized strategy, we designed variants spanning four established stabilization classes commonly applied to class I viral fusion glycoproteins: engineered disulfide bonds, proline substitutions, hydrophobic cavity filling, and electrostatic optimization at the trimer interface. In particular, regions exhibiting substantial conformational divergence between the prefusion and postfusion states (residues 58–96, 137–226 and 460–513) were prioritized, while neighboring structural elements predicted to provide conformational support, particularly the apex-adjacent 227–305 region, were also considered. Accordingly, we engineered 16 interprotomer and intraprotomer disulfide pairs to impose covalent constraints and stabilize the prefusion trimer (Appendix Fig. S1A). For proline substitutions, we targeted hinge- or turn-like positions to reduce local flexibility; in total, 23 such substitutions were designed, comprising 16 single-site proline substitutions and 7 double-proline combinatorial substitutions at adjacent positions (Appendix Fig. S1B). For hydrophobic cavity filling in preF, cavities were classified as surface-exposed cavities and interprotomer interface voids. To reinforce hydrophobic core packing, increase expression yield, and enhance conformational stability, native residues were replaced with higher-volume hydrophobic side chains (Met, Leu, Ile, Phe, Tyr, His, Trp), with each of the 20 targeted sites screened against all seven substitutions (Appendix Fig. S1C). As for electrostatic substitutions, D486 is positioned at the core of the prefusion trimer interface, precisely at the site of tight protomer packing. Based on this, we systematically substituted D486 with each of the other 19 amino acids, with the aim of more effectively eliminating interfacial destabilizing forces (Appendix Fig. S1D).

To further evaluate our preF designs, we used the ectodomain of respiratory syncytial virus subgroup A (strain A2; residues 26–513) as the template for mutational engineering. A C-terminal T4 fibritin foldon trimerization motif was appended to promote trimer assembly, together with His and StrepII tags to facilitate detection and purification, enabling soluble expression of prefusion-stabilized RSV F variants (Fig. 1B). Individual mutants were cloned into pcDNA3.1 and transiently expressed in HEK293T cells in a 96-well format. After 3 days of transfection, clarified supernatants were analyzed by sandwich ELISA to assess antigenicity, specifically quantifying binding to the prefusion-specific antibody D25, with motavizumab as a parallel readout related to total F antigen output (Fig. 1C), thereby enabling comparative evaluation of multiple design classes within the same assay framework.

### Systematic screening and combinatorial optimization prioritized RVF-88A as a candidate design

In the primary ELISA screen, fold changes in binding relative to wild-type (WT, A2 strain) were used to infer antigen properties: binding to the prefusion-specific monoclonal antibody D25 reported prefusion-specific antigenicity, whereas binding to motavizumab was used to approximate total F yield (prefusion plus postfusion). This dual readout enabled rapid functional screening of structure-guided variants. At this stage, we examined individual F substitutions, including engineered cysteine pairs to introduce disulfide bonds, and assessed prefusion antigenicity and short-term stability using a 7-day screening window at 4 °C, a duration previously established for identifying stabilized RSV F variants (McLellan et al, 2013a). Mutants were prioritized for subsequent characterization that demonstrated increased representation of the prefusion state relative to WT, relatively high expression, and improved retention of D25 reactivity after storage.

**Figure 2:**
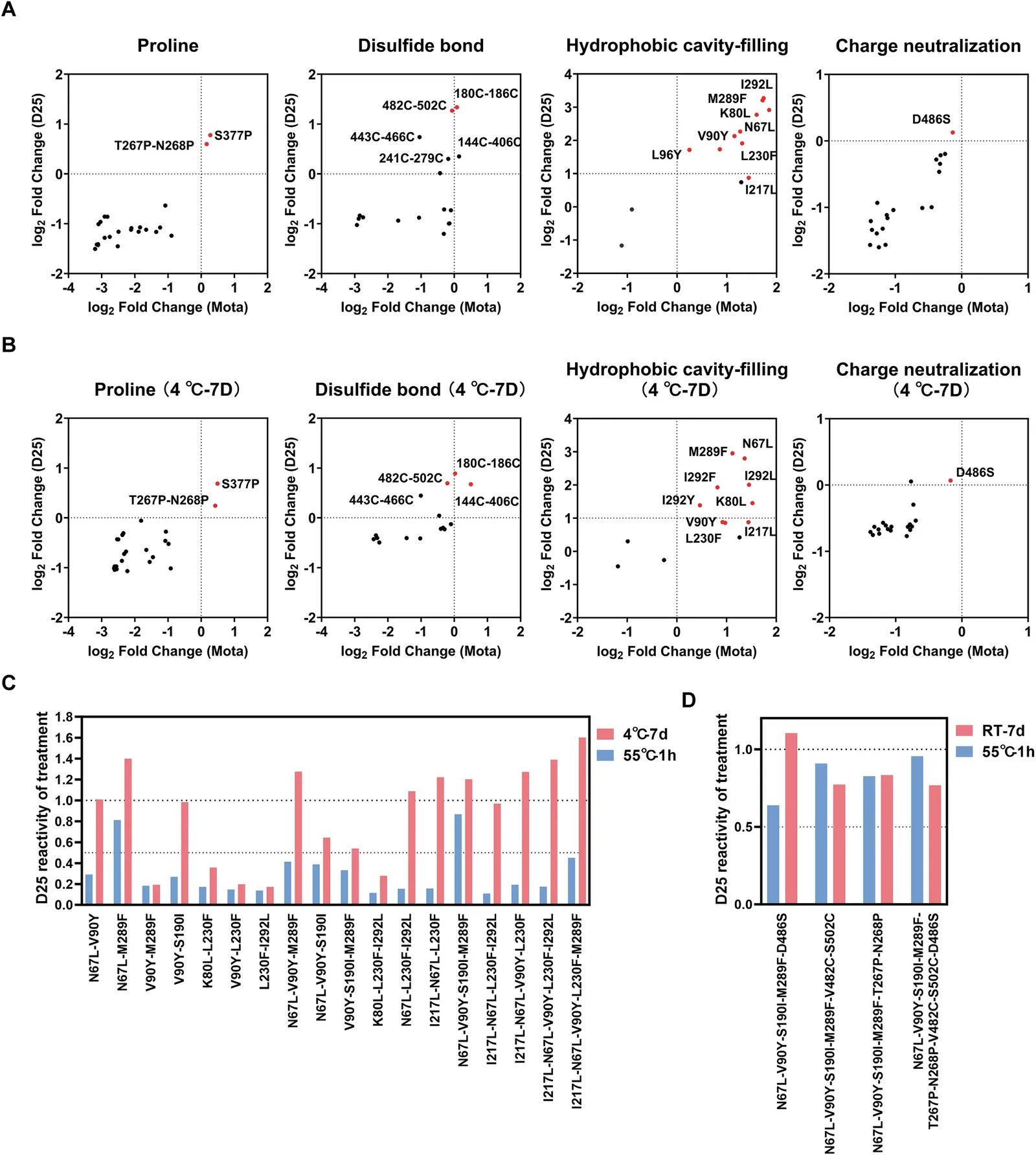
Four screening strategies for designing prefusion-stabilizing RSV F constructs. (**A**, **B**) ELISA reactivity of HEK293T supernatants against the prefusion-specific mAb D25 (site Ø) and motavizumab (site II; binds both preF and postF), measured 72 h after transfection (**A**) and after storage at 4 °C for 7 d (**B**). Axes show log_2_-transformed mutant-to-WT ratios of OD_450_ for motavizumab (*x*) and D25 (*y*). Dashed lines indicate quadrant boundaries and category-specific selection thresholds: for all categories, vertical and horizontal dashed lines are set at *x* = 0 and *y* = 0; for hydrophobic cavity-filling variants, an additional horizontal dashed line at *y* = 1 indicates the twofold increase in D25 binding used for candidate selection. Red symbols indicate shortlisted candidates selected for downstream combinatorial analysis. (**C**, **D**) Retention of D25 binding after thermal challenge (55 °C, 1 h) or storage (4 °C or room temperature, 7 d). The *y* axis reports the mean post-/pre-treatment OD_450_ ratio. (**C**) shows combinations comprising cavity-filling substitutions only; (**D**) shows combinations incorporating cavity-filling substitutions with proline, disulfide, or electrostatic strategies. Data information: Data are calculated based on the mean of OD_450_, *n* = 3. Source data are available online for this figure.

Among proline-based designs, the double substitution T267P–N268P increased D25 binding, indicating enhanced prefusion-specific antigenicity. T267 and N268 localize to the L5 loop at the distal end of the heptad-repeat A (HRA) segment, corresponding to a hinge/turn site at the junction with the downstream helix, despite the broadly similar local architecture in the prefusion and postfusion conformations. We therefore propose that introducing consecutive prolines restricts local flexibility at this hinge, thereby stabilizing the prefusion state and disfavoring large-scale HRA refolding. Among engineered disulfides, V482C–S502C and S180C–S186C showed strong D25 reactivity (Fig. 2A,B). Of note, V482C–S502C appears more geometrically incompatible with the postfusion conformation than S180C–S186C, suggesting a stronger constraint on the prefusion-to-postfusion transition. Because V482C–S502C lies near the C terminus of F, it is positioned away from surface-exposed neutralizing epitopes, potentially reducing the likelihood of epitope perturbation.

From the panel of 126 individual hydrophobic cavity substitutions, 30 variants across 11 positions showed favorable D25 binding under the supernatant ELISA screening conditions. These favorable substitutions were identified across multiple prioritized regions, including three conformationally divergent regions and one adjacent supporting region, consistent with the region-prioritized design rationale. They included N67L/I/W/F/H/Y, K80M/L/F/W, I79L/F/W, V90L/I/Y, L83I, L93F, L96M/I/Y, I217L, L230F, M289L/F/Y, and I292M/L/F/Y (Appendix Fig. S2 and Appendix Table S2). A subset of 13 substitutions, selected based on D25 binding strength and positional representation, was further evaluated by ELISA alongside DS-Cav1, DS, and Cav1 using the same HEK293T supernatant-based screening workflow. Most selected variants showed higher D25 or motavizumab reactivity than DS-Cav1 both at 72 h post-transfection and after 7 days at 4 °C, providing additional support for their prioritization in downstream combinatorial analysis (Appendix Fig. S3). Combinatorial designs preferentially incorporated substitutions from distinct prioritized regions, although combinations of substitutions within the same local structural region were also retained when supported by the screening results. In addition, S190I was incorporated based on prior reports showing that cavity-filling substitutions at position 190 can confer prefusion stabilization, including S190F in DS-Cav1 and S190I in construct pXCS847 (McLellan et al, 2013a; Che et al, 2023); its favorable effect on thermal stability was subsequently supported in combinatorial screening (Fig. 2C). Within the electrostatic class, D486S displayed the strongest D25 binding signal, together with stability upon storage (Fig. 2A,B). For hydrophobic cavity-filling combination mutants, constructs such as N67L-M289F and N67L-V90Y-S190I-M289F retained more than 60% of D25 binding activity even after incubation at 55 °C for 1 h or storage at 4 °C for 7 days (Fig. 2C).

Furthermore, the hydrophobic cavity-filling substitution combination N67L-V90Y-S190I-M289F served as a core background onto which mutations from other stabilization strategies were introduced and screened. At the transient expression level in HEK293T cell supernatants, the combination of hydrophobic cavity-filling and electrostatic mutations N67L-V90Y-S190I-M289F-D486S showed strong D25 binding after thermal challenge and during ambient (room-temperature) storage (Fig. 2D). However, biophysical characterization of purified proteins revealed that adding a single engineered disulfide bond to the cavity-filling core provided only marginal improvements in size-exclusion chromatography (SEC) homogeneity under thermal or storage stress. More complex multi-substitution combinations incorporating proline substitutions and disulfide bonds showed more consistent homogeneity (Appendix Fig. S4), but at the cost of a substantially higher number of non-native mutations. Importantly, within the combinations tested on the cavity-filling core background, addition of D486S was associated with improved stability, particularly in purified-protein analyses under storage stress. We therefore prioritized the five-substitution design (N67L, V90Y, S190I, M289F, and D486S), termed RVF-88, for mechanistic validation as a selected optimized construct within the tested design framework. Introducing single-site substitutions at positions 67, 90, 190, 289, and 486 on both the WT and RVF-88 backbones further supported that the selected residues at their respective positions consistently ranked among the better-performing alternatives tested in terms of prefusion antigenicity, expression level, and conformational stability under thermal and storage stress (Appendix Fig. S5).

### In vitro performance of RVF-88A/B confirms the constructs as viable clinical vaccine candidates

**Figure 3:**
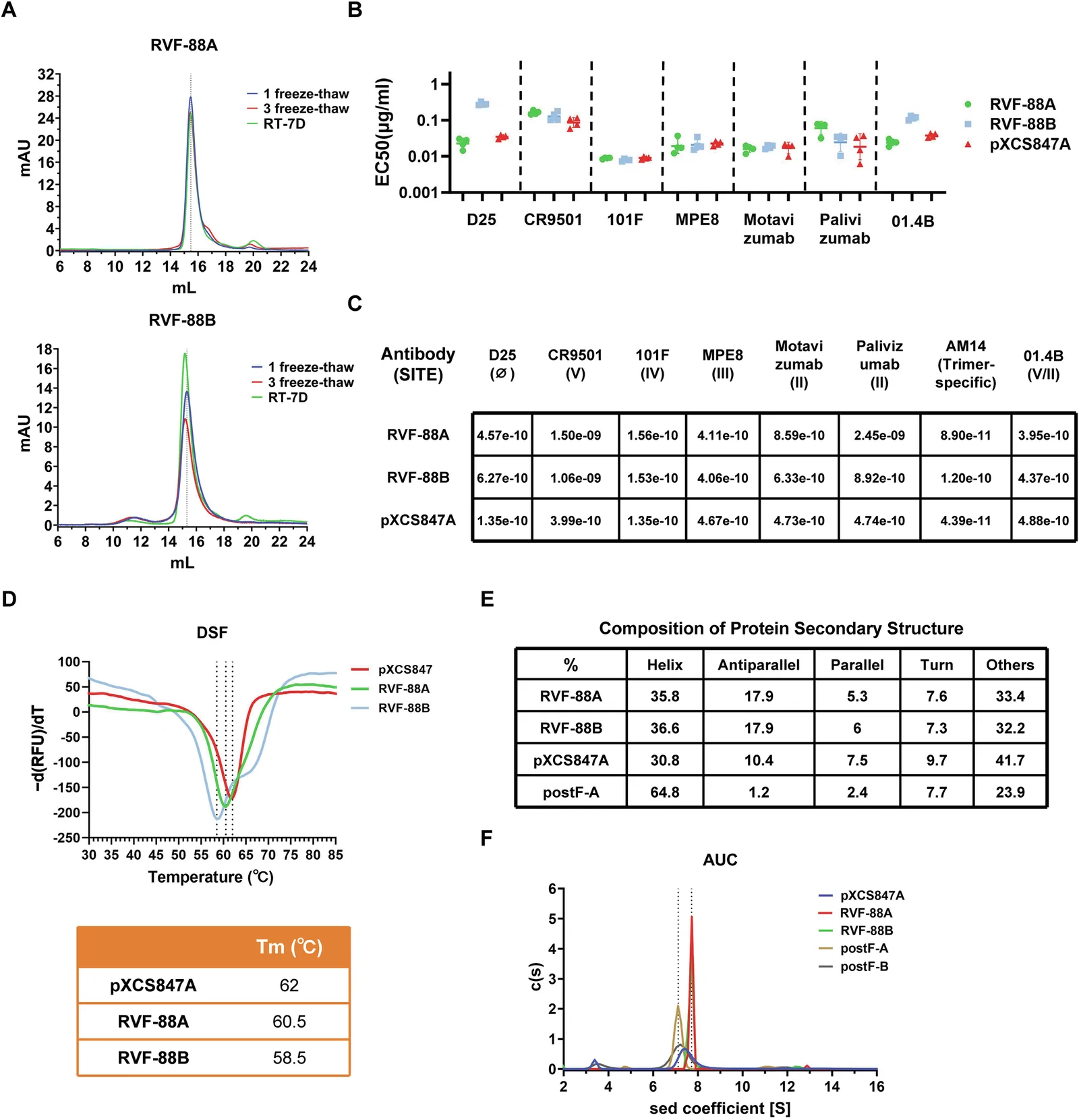
Biophysical and antigenic characterization of RVF-88A and RVF-88B. (**A**) SEC profiles of CHO-expressed RVF-88A (upper) and RVF-88B (lower) under freeze–thaw and ambient storage stress conditions. Traces are color-coded by condition: blue (one freeze–thaw cycle), red (three freeze–thaw cycles), and green (RT-7D, ambient temperature storage for 7 days). (**B**) ELISA-derived EC₅₀ values (µg ml^−1^) for RVF-88A (green circles), RVF-88B (blue squares), and pXCS847A (red triangles) binding to a panel of monoclonal antibodies. Data are presented as geometric mean EC_50_ values with geometric SD from four independent experiments, each performed in technical duplicate (*n* = 4). (**C**) SPR-derived binding affinities (K_D_ values) of RVF-88A, RVF-88B, and pXCS847A to the same antibody panel. (**D**) Differential scanning fluorimetry (DSF) melting curves for RVF-88A (green), RVF-88B (blue), and pXCS847A (red); dashed lines indicate melting temperatures (T_m_) values, which are also summarized in the accompanying table. (**E**) Secondary-structure estimates from circular dichroism (CD) spectroscopy for RVF-88A and RVF-88B, shown as the fraction of α-helix and remaining components, with postF-A as reference. (**F**) Sedimentation velocity analytical ultracentrifugation (SV-AUC) c(s) distributions for RVF-88A and RVF-88B, with postF indicated for comparison; dashed lines mark the fitted sedimentation coefficients. Source data are available online for this figure.

To enable translational development and broad subgroup coverage, we generated two RSV vaccine constructs based on the prevalent RSV-A ON1 and RSV-B BA9 lineages, termed RVF-88A and RVF-88B. To evaluate whether the RVF-88 mutation combination maintained favorable biophysical properties across different RSV F sequence backgrounds, corresponding constructs based on the A2 and BA9 backbones were transiently expressed in HEK293F cells and analyzed by SEC after ambient storage for 7 days. Both constructs showed predominant monodisperse peaks without evident degradation or major aggregation (Fig. 3A). Both antigens were subsequently expressed without purification tags in a clinical-grade CHO cell line. Stable monoclonal cell lines were screened for antigen expression, and one clone for each antigen with high antigen yield and reliable passage stability was selected for cell bank establishment and large-scale production. The purified RVF-88A and RVF-88B proteins exhibited high purity and retained stability after storage at 4 °C for 6 months and at −80 °C for 12 months, with RVF-88A showing a single symmetric main peak by SEC and RVF-88B maintaining a predominant monodisperse peak with minor secondary species under some conditions; both constructs maintained prefusion antigenicity throughout, as confirmed by CD and ELISA (Appendix Fig. S6).

Antigenicity was assessed by complementary ELISA and surface plasmon resonance (SPR) using a panel of monoclonal antibodies spanning major prefusion neutralizing epitopes, including the prefusion apex (D25, site Ø; AM14, prefusion-specific quaternary epitope) and sites II–V (motavizumab and palivizumab, site II; MPE8, site III; 101F, site IV; CR9501, site V and 01.4B, site V/II). Both RVF-88A and RVF-88B bound these antibodies with sub-nanomolar to low-nanomolar affinities. In particular, both constructs strongly bound the Ø-site-specific antibody D25, with RVF-88A (K_D_ = 0.457 nM, EC_50_ = 0.151 nM) and RVF-88B (K_D_ = 0.627 nM, EC_50_ = 1.82 nM), comparable with the binding profile of Pfizer’s pXCS847A variant (Fig. 3B,C; Appendix Figs. S7–S10 and Appendix Table S3).

Biophysical characterization further supported a prefusion-like profile of RVF-88A and RVF-88B. Differential scanning fluorimetry (DSF) revealed melting temperatures of 60.5 °C for RVF-88A and 58.5 °C for RVF-88B (Fig. 3D). Circular dichroism spectroscopy estimated α-helical contents of 35.8% and 36.6%, respectively, substantially lower than that of postF-A (64.8%), consistent with a prefusion-like secondary-structure signature (Fig. 3E). Sedimentation velocity analysis corroborated these findings, with RVF-88A (MW = 180 kDa, S = 7.72) and RVF-88B (MW = 182 kDa, S = 7.69) displaying sedimentation coefficients distinct from postF (MW = 180 kDa, S = 7.12), supporting that RVF-88A and RVF-88B predominantly adopt the preF conformation and construct as viable clinical vaccine candidates (Fig. 3F).

### RVF-88 elicits robust and durable immunogenicity in mice for up to 5 months

**Figure 4:**
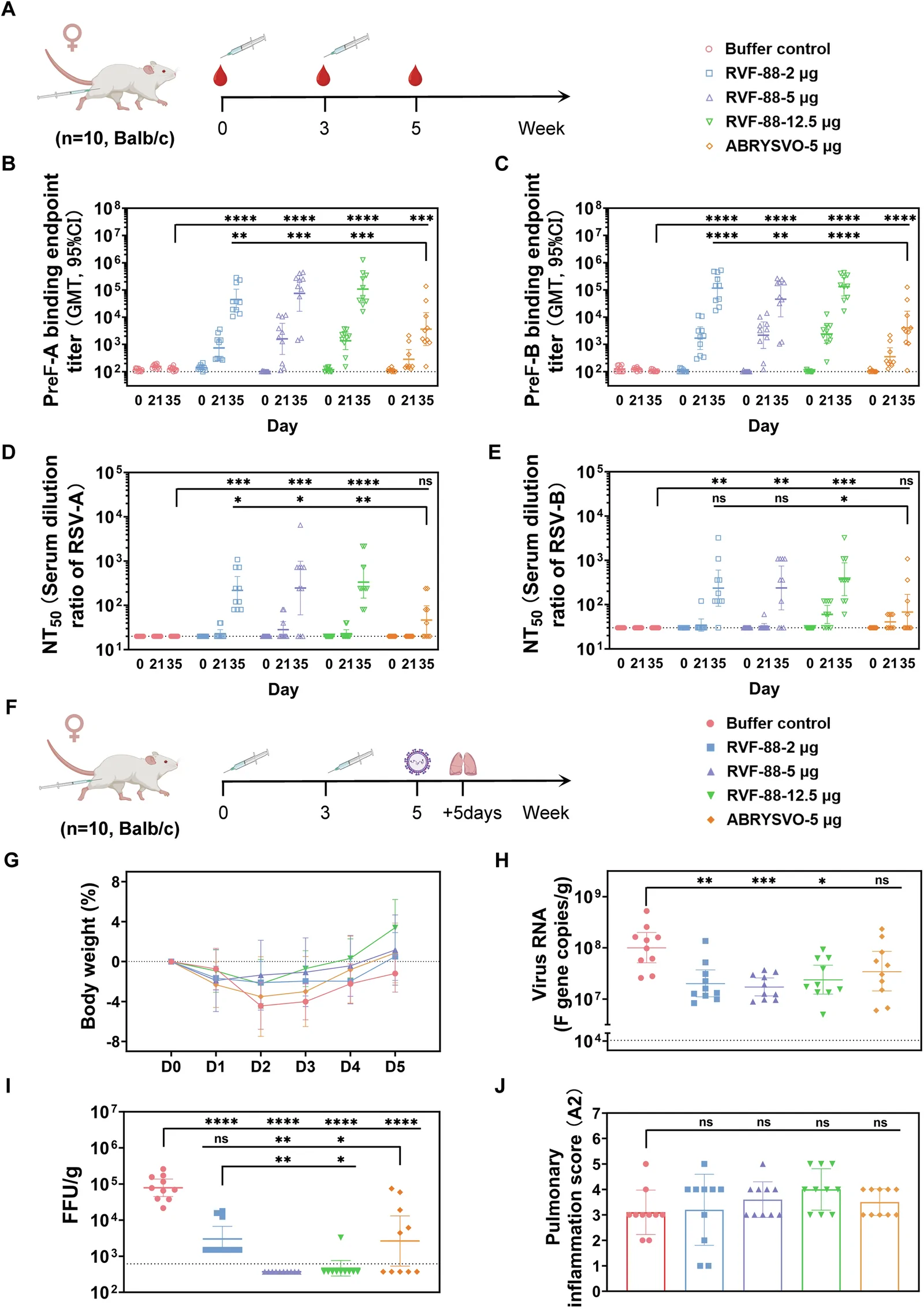
Immunogenicity evaluation and protective efficacy of RVF-88 in BALB/c mice. (**A**) Female BALB/c mice (*n* = 10 per group) were immunized intramuscularly on days 0 and 21 with bivalent RVF-88 (2, 5, or 12.5 μg total antigen), ABRYSVO (5 μg), or buffer. Serum was collected on days 0, 21, and 35. (**B**, **C**) PreF-A-specific (**B**) and PreF-B-specific (**C**) serum binding IgG titers measured using RVF-88A and RVF-88B, respectively, as coating antigens at the indicated time points (*n* = 10, except for the ABRYSVO group at day 21 with *n* = 9 due to sample unavailability). Day 35 (**B**): RVF-88-2 μg vs. Buffer control, *P* < 0.0001; RVF-88-5 μg vs. Buffer control, *P* < 0.0001; RVF-88-12.5 μg vs. Buffer control, *P* < 0.0001; ABRYSVO-5 μg vs. Buffer control, *P* = 0.0001; RVF-88-2 μg vs. ABRYSVO-5 μg, *P* = 0.0060; RVF-88-5 μg vs. ABRYSVO-5 μg, *P* = 0.0006; RVF-88-12.5 μg vs. ABRYSVO-5 μg, *P* = 0.0001. Day 35 (**C**): RVF-88-2 μg vs. Buffer control, *P* < 0.0001; RVF-88-5 μg vs. Buffer control, *P* < 0.0001; RVF-88-12.5 μg vs. Buffer control, *P* < 0.0001; ABRYSVO-5 μg vs. Buffer control, *P* < 0.0001; RVF-88-2 μg vs. ABRYSVO-5 μg, *P* < 0.0001; RVF-88-5 μg vs. ABRYSVO-5 μg, *P* = 0.0060; RVF-88-12.5 μg vs. ABRYSVO-5 μg, *P* < 0.0001. (**D**, **E**) Serum neutralization titers (NT_50_) against RSV A2 (**D**) and RSV B9320 (**E**) at the indicated time points (*n* = 10, except for the ABRYSVO group at day 21 with *n* = 9 due to sample unavailability). Day 35 (**D**): RVF-88-2 μg vs. Buffer control, *P* = 0.0005; RVF-88-5 μg vs. Buffer control, *P* = 0.0003; RVF-88-12.5 μg vs. Buffer control, *P* < 0.0001; RVF-88-2 μg vs. ABRYSVO-5 μg, *P* = 0.0468; RVF-88-5 μg vs. ABRYSVO-5 μg, *P* = 0.0266; RVF-88-12.5 μg vs. ABRYSVO-5 μg, *P* = 0.0056. Day 35 (**E**): RVF-88-2 μg vs. Buffer control, *P* = 0.0038; RVF-88-5 μg vs. Buffer control, *P* = 0.0036; RVF-88-12.5 μg vs. Buffer control, *P* = 0.0003; RVF-88-12.5 μg vs. ABRYSVO-5 μg, *P* = 0.0228. The corresponding GMTs and 95% CIs are provided in Appendix Table S4, and individual data points are included in the Source Data files. (**F**) Female BALB/c mice (*n* = 10 per group) were immunized with bivalent RVF-88 (2 μg, 5 μg, or 12.5 μg), ABRYSVO (5 μg), or buffer on days 0 and 21, and challenged with RSV A2 2 weeks after the boost (day 35); lungs were collected on day 5 post-challenge. (**G**) Percentage change from initial body weight of the treatment groups following challenge (*n* = 10). (**H**) Lung viral load (RSV F gene copies g^−1^) quantified by qRT-PCR (*n* = 10). RVF-88-2 μg vs. Buffer control, *P* = 0.0032; RVF-88-5 μg vs. Buffer control, *P* = 0.0009; RVF-88-12.5 μg vs. Buffer control, *P* = 0.0100. (**I**) Lung infectious viral titers (FFU g^−1^) of BALB/c mice from the treatment groups (*n* = 10). RVF-88-2 μg vs. Buffer control, *P* < 0.0001; RVF-88-5 μg vs. Buffer control, *P* < 0.0001; RVF-88-12.5 μg vs. Buffer control, *P* < 0.0001; ABRYSVO-5 μg vs. Buffer control, *P* < 0.0001; RVF-88-2 μg vs. RVF-88-5 μg, *P* = 0.0033; RVF-88-2 μg vs. RVF-88-12.5 μg, *P* = 0.0104; RVF-88-5 μg vs. ABRYSVO-5 μg, *P* = 0.0064; RVF-88-12.5 μg vs. ABRYSVO-5 μg, *P* = 0.0194. (**J**) Pulmonary inflammation score in the lung tissue of the treatment groups after challenge (*n* = 10). Data information: Data are presented as geometric mean with 95% confidence intervals (CIs) (**B**–**E**, **H**, **I**), or mean ± standard deviation (SD) (**G**, **J**). For (**B**–**E**), statistical comparisons shown are for day 35 only. Dashed lines indicate the limit of detection in (**B**–**E**, **H**, **I**), and no change from initial body weight (0%) in (**G**). Statistical significance was determined using one-way ANOVA with Tukey’s multiple comparisons test; *P* values are indicated (ns not significant, *P < 0.05, **P < 0.01, ***P < 0.001, ****P < 0.0001). Group symbols and colors are defined in the legend. Source data are available online for this figure.

To assess immunogenicity, RVF-88 was formulated as a bivalent vaccine comprising RVF-88A and RVF-88B in a 1:1 mixture (by mass) and administered intramuscularly to mice (*n* = 10 per group) on days 0 and 21 at total antigen doses of 2, 5, or 12.5 μg (Fig. 4A). Buffer and 5 μg ABRYSVO served as controls. RVF-88 induced strong antigen-specific and neutralizing antibody responses against both RSV-A and RSV-B. Compared to the buffer group, RVF-88 induced neutralizing antibody responses against both RSV A2 and RSV B9320 across all tested doses, whereas the ABRYSVO group showed more limited increases under the same conditions. Although RVF-88 immunogenicity and protection generally showed dose-dependent trends, differences among the 2, 5, and 12.5 μg groups did not reach statistical significance (*P* > 0.05) across most binding antibody, neutralization, and viral-burden endpoints under the tested conditions. Consistent with this, RVF-88 dose groups were broadly comparable to ABRYSVO across the measured immunogenicity and protection readouts, with significantly higher responses observed for selected binding and neutralizing antibody endpoints. Two weeks after the second immunization (day 35), the 5 μg RVF-88 group showed higher binding antibody geometric mean titers (GMT) than ABRYSVO against RSV-A (75,155 versus 33,635) and RSV-B (45,619 versus 4032) (Fig. 4B,C). Serum neutralization titers (NT_50_ GMT) were numerically higher for RVF-88 than for ABRYSVO against RSV A2 (246 versus 51) and RSV B9320 (238 versus 74), with a statistically significant difference for RSV A2 at the matched 5 μg dose (Fig. 4D,E; Appendix Table S4).

In addition, to evaluate the protection of RVF-88 in vivo, mice were immunized with the vaccine and challenged with RSV A2 2 weeks after the second immunization. Lungs were harvested on day 5 post-challenge (Fig. 4F). On the second day after challenge, the greatest body weight loss was observed in the buffer control group, followed by the ABRYSVO group, while the RVF-88 groups showed minimal weight loss (Fig. 4G). Compared with the buffer control group, RVF-88 vaccination reduced lung viral RNA copies by 4.2- to 5.9-fold, whereas ABRYSVO showed a 2.9-fold reduction (Fig. 4H). Notably, all RVF-88 dose groups showed statistically significant reductions in lung viral RNA copies, whereas the reduction observed in the ABRYSVO group did not reach statistical significance (Fig. 4H). In addition, lung viral titers in all RVF-88 immunized groups were reduced to minimal levels, representing highly significant protection compared with the buffer control group (*P* < 0.0001). In particular, the 5 μg and 12.5 μg RVF-88 doses demonstrated strong efficacy by substantially reducing pulmonary viral titers, with viral levels reduced to below the limit of detection (LOD) in most animals (Fig. 4I). These findings suggest that the RVF-88 vaccine induces robust immunogenicity and provides effective protection against RSV A2 challenge in the mouse model. Pathological analysis was performed on lung tissues collected on day 5 post-challenge to assess any tissue damage across different treatment groups. Histopathological scoring revealed no increase in lung pathology in RVF-88 immunized mice compared with buffer or ABRYSVO under the conditions tested (Fig. 4J; Appendix Fig. S11). ELISpot (Appendix Fig. S12) showed detectable RSV-specific IFN-γ responses following RVF-88 vaccination, although differences versus controls were not significant.

To assess the durability of the immune response induced by the RVF-88 vaccine, BALB/c mice were immunized intramuscularly with 5 μg of bivalent RVF-88 at weeks 0 and 3. Blood samples were collected at weeks 0, 3, 5, 7, 9, 13, 18, and 22 to monitor antibody levels in the serum (Appendix Fig. S13A). Antigen-specific IgG titers peaked at week 13, with titers of 68,622 against RSV preF-A and 64,910 against RSV preF-B (Appendix Fig. S13B,C). Neutralizing antibody titers increased by week 9 and were maintained thereafter (Appendix Fig. S13D,E). These data indicate that RVF-88-based vaccination provides sustained binding and neutralizing antibody responses through week 22 (154 days) in the BALB/c mouse model.

### Immunogenicity and protective efficacy of RVF-88 in cotton rats

**Figure 5:**
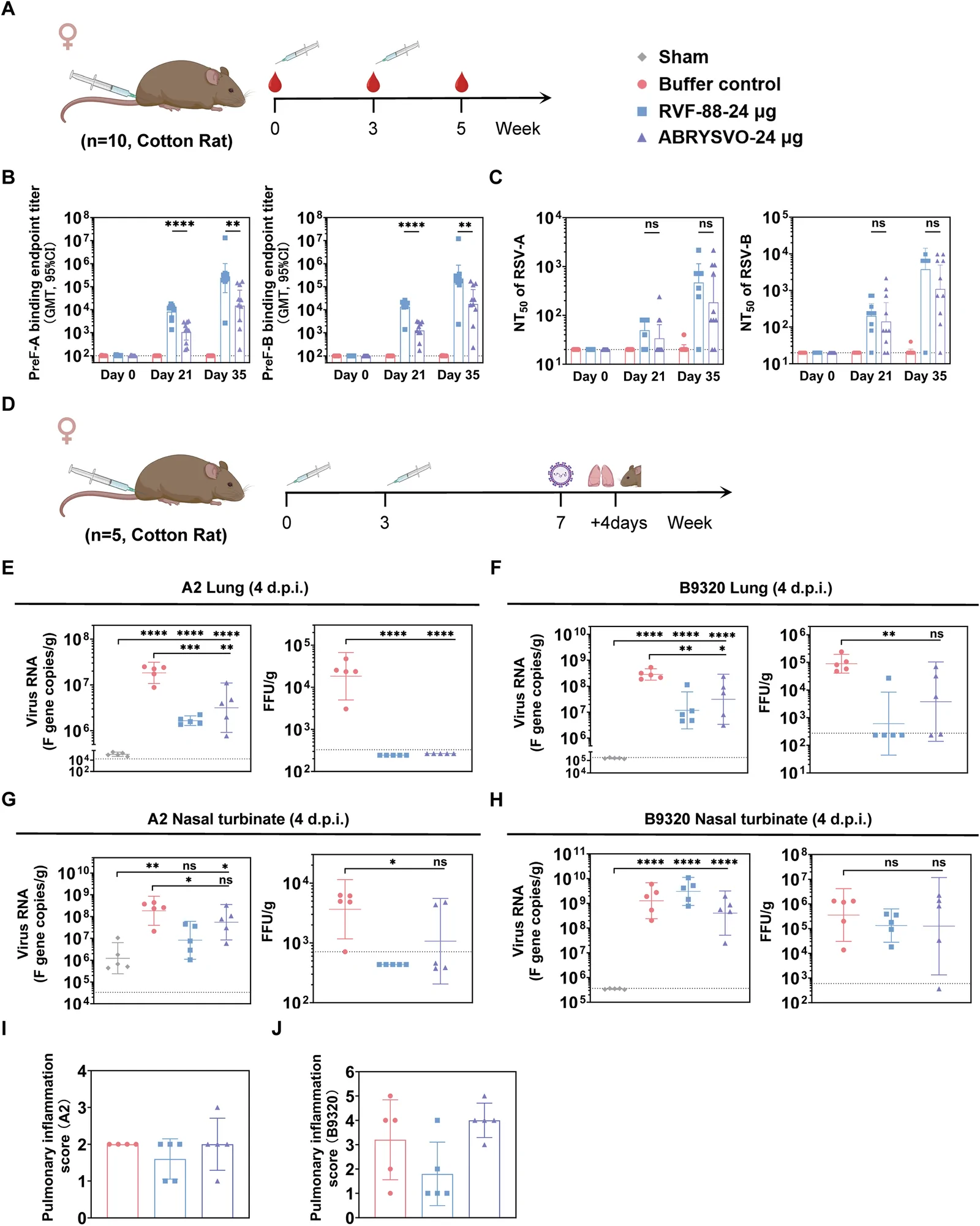
Immunogenicity and protection conferred by RVF-88 in cotton rats. (**A**) Female cotton rats (*n* = 10 per group) were immunized intramuscularly twice, with a 3-week interval between doses, using bivalent RVF-88 (24 μg total antigen), ABRYSVO (24 μg), or buffer as a placebo control; serum was collected on days 0, 21, and 35. (**B**) Antigen-specific serum binding IgG titers against preF-A (left) and preF-B (right), *n* = 10. Day 21 (preF-A): RVF-88 vs. ABRYSVO, *P* < 0.0001. Day 35 (preF-A): RVF-88 vs. ABRYSVO, *P* = 0.0035. Day 21 (preF-B): RVF-88 vs. ABRYSVO, *P* < 0.0001. Day 35 (preF-B): RVF-88 vs. ABRYSVO, *P* = 0.0081. (**C**) Serum neutralization titers (NT_50_) against RSV A2 (left) and RSV B9320 (right), *n* = 10. The corresponding GMTs and 95% CIs are provided in Appendix Table S4, and individual values are included in the Source Data files. (**D**) Schematic of the viral challenge study in female cotton rats (*n* = 5 per group), showing immunization at weeks 0 and 3, intranasal challenge with RSV A2 or RSV B9320 at week 7, and sample collection at day 4 post-challenge. (**E**, **F**) Lung viral RNA (copies g^−1^, left) and infectious viral titers (FFU g^−1^, right) after RSV A2 (**E**) or RSV B9320 (**F**) challenge (*n* = 5). Lung viral RNA (A2): RVF-88 vs. Buffer control, *P* = 0.0002; ABRYSVO vs. Buffer control, *P* = 0.0034; Sham vs. Buffer control, *P* < 0.0001; RVF-88 vs. Sham, *P* < 0.0001; ABRYSVO vs. Sham, *P* < 0.0001. Viral titers (A2): RVF-88 vs. Buffer control, *P* < 0.0001; ABRYSVO vs. Buffer control, *P* < 0.0001. Lung viral RNA (B9320): RVF-88 vs. Buffer control, *P* = 0.0021; ABRYSVO vs. Buffer control, *P* = 0.0332; Sham vs. Buffer control, *P* < 0.0001; RVF-88 vs. Sham, *P* < 0.0001; ABRYSVO vs. Sham, *P* < 0.0001. Viral titers (B9320): RVF-88 vs. Buffer control, *P* = 0.0059. (**G**, **H**) Nasal turbinate viral RNA (copies g^−1^, left) and infectious viral titers (FFU g^−1^, right) after RSV A2 (**G**) or RSV B9320 (**H**) challenge (*n* = 5). Nasal turbinate viral RNA (A2): RVF-88 vs. Buffer control, *P* = 0.0458; Sham vs. Buffer control, *P* = 0.0014; ABRYSVO vs. Sham, *P* = 0.0150. Viral titers (A2): RVF-88 vs. Buffer control, *P* = 0.0186. Nasal turbinate viral RNA (B9320): Sham vs. Buffer control, *P* < 0.0001; RVF-88 vs. Sham, *P* < 0.0001; ABRYSVO vs. Sham, *P* < 0.0001. (**I**, **J**) Lung inflammation scores in cotton rats following RSV A2 (**I**) or RSV B9320 (**J**) challenge (*n* = 5, except for the RSV A2 challenge Buffer control group with *n* = 4 due to sample unavailability). Data information: Data are presented as geometric means with 95% confidence intervals (CIs) (**B**, **C**, **E**–**H**), or mean ± standard deviation (SD) for pathology scores (**I**, **J**). Dashed lines indicate the limit of detection. Statistical significance was determined using one-way ANOVA with Tukey’s multiple comparisons test; *P* values are indicated (ns not significant, *P < 0.05, **P < 0.01, ***P < 0.001, ****P < 0.0001). Group symbols and colors are defined in the legend. Source data are available online for this figure.

The immunogenicity of RVF-88 was further assessed in cotton rats. Animals were immunized intramuscularly twice, 3 weeks apart, with 24 μg of RVF-88 (total antigen), with buffer and a dose-matched ABRYSVO group as controls (Fig. 5A). As shown in Fig. 5B,C and Appendix Table S4, both RVF-88 and ABRYSVO elicited robust antigen-specific antibody responses against RSV preF-A and preF-B, as well as serum neutralization (NT_50_) against RSV A2 and RSV B9320 strains. Two weeks after the booster immunization (day 35), RVF-88 induced significantly higher antigen-specific binding antibody titers than ABRYSVO against both preF-A and preF-B (*P* < 0.01). Neutralizing antibody titers against RSV A2 and RSV B9320 were also numerically higher in the RVF-88 group than in the ABRYSVO group (2.6-fold and 3.4-fold, respectively), although these differences did not reach statistical significance.

To evaluate protection, cotton rats (*n* = 5 per group) were intranasally challenged 4 weeks after the second immunization with either 1 × 10^6^ PFU of RSV A2 or 3 × 10^5^ PFU of RSV B9320 (Fig. 5D). Viral burden analyses in cotton rats showed that RVF-88 significantly reduced both lung viral RNA loads and lung viral titers after A2 and B9320 challenge relative to the buffer control. ABRYSVO significantly reduced both endpoints after the A2 challenge. However, after the B9320 challenge, ABRYSVO significantly reduced lung viral RNA loads but not lung viral titers relative to the buffer control (Fig. 5E,F). In nasal turbinates, RVF-88 significantly reduced both viral load and viral titer compared with the buffer control after A2 challenge (Fig. 5G), whereas neither RVF-88 nor ABRYSVO significantly reduced nasal viral burden after B9320 challenge (Fig. 5H). Representative H&E-stained lung sections from buffer-, ABRYSVO-, and RVF-88-immunized cotton rats after viral challenge were generally consistent with the quantitative histopathology scores shown in Fig. 5I,J, and did not indicate increased lung inflammatory pathology in the RVF-88 group under the tested conditions (Appendix Fig. S14). These findings demonstrate that RVF-88 induces broad immune responses and confers protection in the cotton rat model, with significant virological improvement in the lung after both A2 and B9320 challenge and in the nasal turbinate after A2 challenge, showing a broader pattern of statistically significant protection than ABRYSVO under the conditions tested.

### Cryo-EM and crystal structures support the prefusion architecture and apical cavity-reduction design of RVF-88

**Figure 6:**
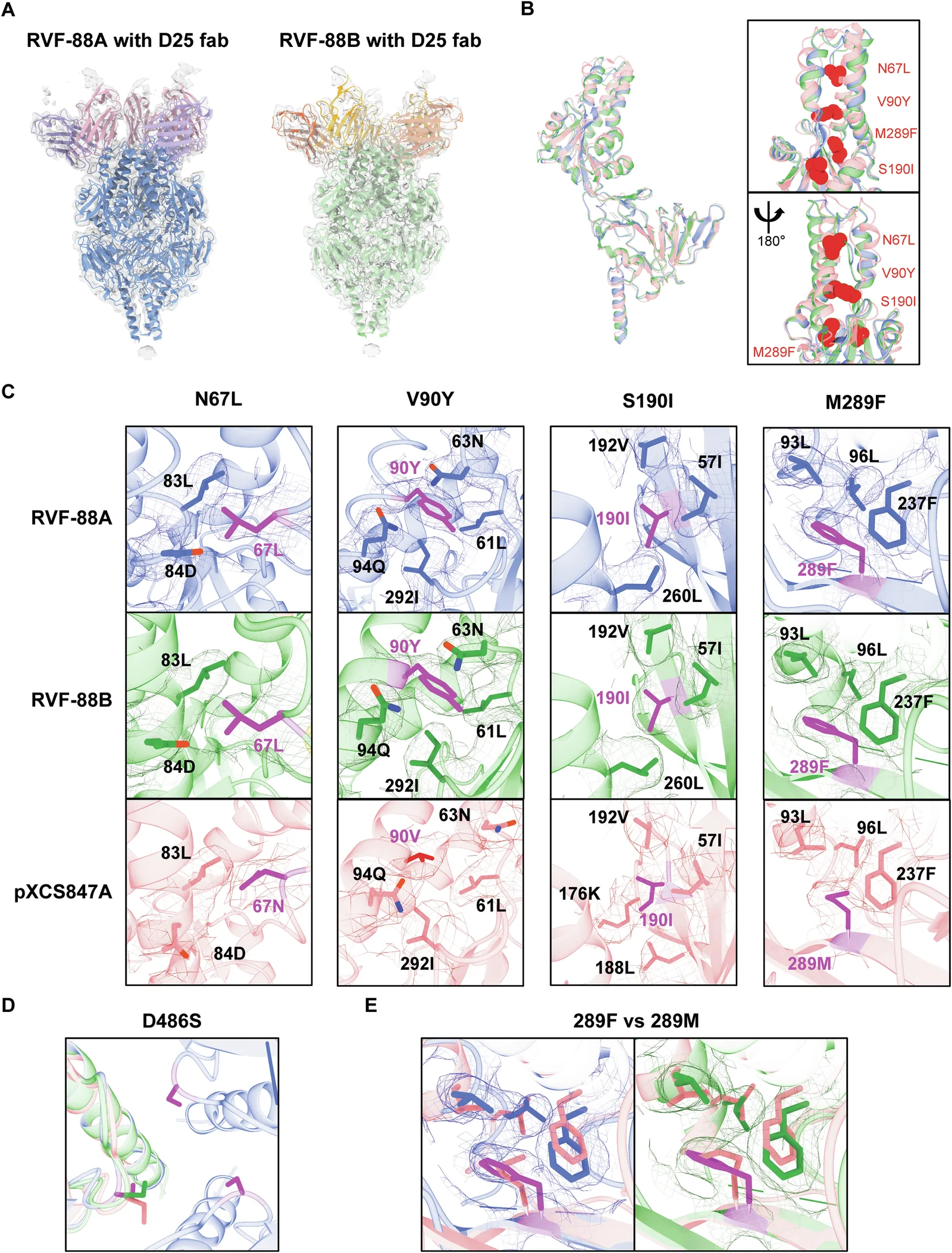
Cryo-EM structures of RVF-88A and RVF-88B in complex with D25 Fab. (**A**) Cryo-EM density maps of RVF-88A (left, plum) and RVF-88B (right, green) bound to the D25 Fab (heavy chain in violet and coral, light chain in pink and orange), resolved to 2.96 Å and 2.77 Å, respectively (gold-standard FSC = 0.143). Density corresponding to the D25 Fab constant domains and the C-terminal foldon motif was unresolved due to flexibility and therefore not modeled. (**B**) Overlay of cryo-EM models of RVF-88A (plum), RVF-88B (green), and pXCS847A (light pink; PDB ID: 7UJA) visualized using UCSF Chimera/ChimeraX. The overall conformations of RVF-88A and RVF-88B closely overlapped with that of pXCS847A, with minor structural deviations detected at the apex region (highlighted in the inset); the four apex-site residues at the top are displayed as red spheres. (**C**) Local views around residues 67, 90, 190 and 289 in RVF-88A, RVF-88B, and pXCS847A. Site residues are highlighted in magenta (sticks), together with residues within 4 Å of the displayed atoms (sticks). Cryo-EM density is shown as a mesh. Heteroatoms are colored by element: oxygen in orange, nitrogen in blue, and sulfur in yellow. (**D**) Local superposition at D486S for RVF-88A, RVF-88B, and pXCS847A, showing similar backbone and side-chain conformations within the map resolution. (**E**) Position 289 comparison: Phe289 (RVF-88A in plum, left; RVF-88B in green, right) versus Met289 (pXCS847A in light pink). Phe289 exhibits tighter packing against nearby residues, filling a local hydrophobic cavity relative to Met289.

To gain further structural insights into RVF-88, cryo-electron microscopy (cryo-EM) was performed to determine the structures of RVF-88A in complex with the D25 Fab and RVF-88B in complex with the D25 Fab, at overall resolutions of 2.96 and 2.77 Å, respectively (gold-standard FSC = 0.143) (Fig. 6A). As reported previously, the C-terminal foldon of F1 and the Fab constant domains showed insufficient density due to flexibility and were not modeled. The stabilizing mutations preserved the integrity of local secondary structures, and the overall RVF-88 architecture was highly similar to that of pXCS847A (PDB 7UJA), with Cα RMSDs of 1.18 Å (F1) and 1.45 Å (F2) for RVF-88A and 1.17 Å (F1) and 1.56 Å (F2) for RVF-88B over the ordered ectodomain (Fig. 6B). To complement the D25-bound cryo-EM analysis, antibody-free crystal structures of RVF-88A and RVF-88B were also analyzed and showed trimeric prefusion-like architectures consistent with the D25-bound cryo-EM structures (Appendix Fig. S15A,B). Local cavity analysis of the trimeric prefusion apex showed that RVF-88A had lower total cavity volume and surface area than selected prefusion F reference structures within the same local search region and under identical detection parameters, consistent with the intended cavity-reduction design at the prefusion apex (Appendix Fig. S15C,D and Appendix Table S5).

The cavity-filling substitutions at positions 67, 90, 190, and 289 (N67L, V90Y, S190I, and M289F) reinforce local packing and secondary-structure integrity at the apex (Fig. 6C). Residue 67 is located in the apex/apex-proximal region of prefusion F, a region previously implicated in preF conformational stability (Krarup et al, 2015). In RVF-88, N67 was replaced with leucine (N67L), which is oriented inward and is positioned near residues in the α1 region, whereas the corresponding N67 in DS-Cav1 is observed in a different local environment near the α4 region (Appendix Fig. S15E). Notably, residue 67 does not directly contact the D25 epitope, as its side chain is oriented inward; accordingly, the mutation had minimal impact on antibody recognition, consistent with preserved epitope presentation in the RVF-88–D25 structure. The S190I substitution places a β-branched hydrophobic side chain within the local cavity at site 190, where close contacts with neighboring residues are observed in the RVF-88–D25 structure. Although S190I has also been used as a cavity-filling substitution in pXCS847A, the neighboring contacts around I190 differ between the two constructs, indicating that the same substitution can be accommodated through distinct local structural environments. The D486S substitution is consistent with reduced electrostatic repulsion at the trimer interface by eliminating clustered acidic side chains (Fig. 6D).

V90, located in the α1 helix (residues 74–96) of F2 near the apex/site Ø, was replaced with an aromatic residue to fill a nearby cavity and enhance hydrophobic packing with residues L61, N63, and I292, consistent with the intended cavity-filling design. The cryo-EM model further supports that M289F, at a conserved position, enhances aromatic side-chain packing and expands local hydrophobic contacts (Fig. 6E). The positioning of the F289 side chain brings it into closer proximity with L93, L96, and F237, facilitating hydrophobic interactions that reinforce a hydrophobic pocket adjacent to helix α1. This tighter packing is consistent with reduced propensity for conformational triggering and stabilization of the prefusion state. Overall, the five stabilizing substitutions are distributed across distinct structural regions of the F protein, with four located within conformationally dynamic segments. Structural analysis of the RVF-88–D25 Fab complex supports preservation of the prefusion-specific epitope, indicating that these substitutions effectively stabilize the F protein in a prefusion trimeric state.

## Discussion

The present study describes the design and characterization of a prefusion-stabilized RSV F vaccine candidate, RVF-88, identified through a region-prioritized and experimentally filtered optimization workflow. The final construct embodies what we refer to as a “light-lock” design concept, defined here as prefusion stabilization without the introduction of exogenous covalent disulfide locks. This concept differs from disulfide-containing prefusion F designs, such as DS-Cav1, which incorporate engineered cysteines together with additional stabilizing mutations. In RVF-88, multiple established stabilization classes were evaluated within the same optimization framework, and the final construct converged on a comparatively low-mutation combination of apex-centered cavity-reducing hydrophobic substitutions and D486-centered electrostatic tuning. While disulfide-stabilized constructs have significantly advanced RSV vaccine development, the long-term maintenance of prefusion-specific antigenicity can remain sensitive to construct context and storage conditions, as illustrated by reports of storage-associated conformational changes and reduced D25 reactivity for DS-Cav1 after prolonged storage at 4 °C (Flynn et al, 2016). In addition, recent work has continued to explore alternative prefusion-stabilization strategies, including proline-based and hydrophobic-substitution approaches, to improve RSV preF antigen stability and manufacturability (Huang et al, 2025; Song et al, 2025). In this context, RVF-88 provides an alternative prefusion-stabilization strategy that preserves key antigenic and structural features in the assays performed here without introducing exogenous cysteines. Nevertheless, whether this comparatively low-mutation, non-disulfide design offers advantages in large-scale manufacturability, long-term stability, or epitope preservation relative to existing benchmarks will require further empirical evaluation.

At the molecular level, the five stabilizing substitutions selected in RVF-88 (N67L, V90Y, S190I, M289F, D486S) define a modified stabilization strategy centered on apical cavity reduction and local electrostatic reinforcement, rather than extensive covalent locking. This focus on the prefusion head is consistent with prior head-focused RSV F immunogen studies, including LC2DM, which highlighted the immunological importance of the apex containing sites Ø and V (Lin et al, 2025). Because the prefusion apex contains dominant neutralizing surfaces yet also exhibits intrinsic conformational flexibility, targeted structural tuning of this region may help preserve prefusion antigenic architecture while reducing excessive apex mobility. Consistent with this design rationale, the antibody-free crystal structures of RVF-88A and RVF-88B showed trimeric prefusion-like architectures, and cavity analysis revealed reduced apical hydrophobic cavity volume and surface area compared with the reference prefusion F structure. Although this cavity reduction does not by itself prove enhanced thermodynamic stability, the observed reduction in apical void volume is consistent with the intended cavity-reduction design and may contribute to maintenance of the prefusion architecture. In structural terms, L67, Y90, I190, and F289 cooperate across the head (apex) region of the prefusion F trimer—spanning the upper, middle, and lower tiers of the apical helical assembly—to intensify hydrophobic contacts within the apex. Relative to the cryo-EM reference of pXCS847A (PDB: 7UJA), L67 (vs N67) sits closer to the α1 segment, enhancing local side-chain packing at the top of the apex; Y90 and F289 contribute aromatic rings that can engage a broader set of nearby nonpolar residues, expanding van der Waals complementarity in the middle and basal portions of the apex bundle. Although S190I was previously used as a cavity-filling substitution in the pXCS847A framework, the RVF-88 structure suggests a distinct local accommodation of this residue, indicating that its structural contribution may depend on the surrounding mutation context. The relative contribution of S190I at the single-mutation level was not directly resolved in the present study and could be further clarified by future back-mutation or targeted mutational analyses. These substitutions support a molecular rationale for apex-centered packing reinforcement by reducing the apical hydrophobic cavity volume and are consistent with the intended cavity-filling design, which may contribute to local stabilization of the prefusion apex. D486S, in contrast, is consistent with electrostatic optimization within the D486–E487–D489 acidic patch, which may reduce intrapatch repulsion and enhance thermal robustness, complementing the cavity-filling strategy (Che et al, 2023). Taken together, the final construct converged on a combination of apex-centered hydrophobic packing reinforcement together with D486-centered electrostatic tuning, providing useful structural insights into prefusion RSV F antigen engineering.

Although selected disulfide- and proline-based variants improved individual readouts, they were not prioritized as the basis of the final construct because, under the comparative criteria used here, they offered a less favorable balance of stability gain, purified-protein behavior, mutation burden, and antigenic preservation than the final low-mutation design. Notably, the structural stabilization achieved without interprotomer disulfides mirrors the mechanistic insights of recent “UFC” frameworks (Lee et al, 2024), but here emerges from a distinct convergence on region-prioritized packing reinforcement and electrostatic tuning, supporting the broader principle that localized energetic balancing may effectively substitute for rigid covalent locks.

In vivo, bivalent RVF-88, containing RSV A- and B-lineage variants at a 1:1 mass ratio, elicited strong neutralizing antibody responses in both mice and cotton rats and reduced pulmonary viral burden following challenge. Robust immunogenicity was observed even in the absence of an adjuvant, supporting the antigenic integrity of the engineered preF. Despite robust serum neutralization, RSV replication in cotton rats tends to persist in the nasal cavity than in the lung, and no reduction in nasal turbinate titers was observed upon RSV-B (B9320) challenge. These findings are consistent with previous reports suggesting that vaccine-mediated protection may manifest more readily in the lower airway than in the upper airway (Boukhvalova et al, 2009; Sun et al, 2024). Taken together, these observations are consistent with the possibility that stronger mucosal immunity may be required for more effective upper-airway protection. Accordingly, mucosa-directed strategies may be beneficial, such as intranasal delivery or adjuvants that enhance local IgA and Fc-mediated functions (van Erp et al, 2019; Sefat et al, 2025). Compared with the licensed ABRYSVO formulation, RVF-88 induced higher geometric mean titers against both RSV A2 and RSV B9320, consistent with prior evidence that preservation of prefusion-specific epitopes correlates with neutralization breadth and potency (Ngwuta et al, 2015; Che et al, 2023). The higher binding antibody titers observed in RVF-88-vaccinated animals may be partially influenced by the use of RVF-88A and RVF-88B as coating antigens in the ELISA assays, which could favor detection of homologous antibody responses. Nevertheless, functional neutralization assays and in vivo challenge studies further supported the immunogenicity and protective potential of RVF-88. During long-term follow-up, some individual animals showed undetectable serum neutralizing antibody titers at later time points, potentially reflecting individual variability in antibody responses and the detection limits of the assay. Importantly, most animals maintained detectable neutralizing activity throughout the observation period. These findings support the potential of a comparatively low-mutation, disulfide-free design strategy for RSV vaccines.

Beyond RSV, the stabilization design of RVF-88—anchored on localized hydrophobic reinforcement and apical cavity reduction—may provide a transferable concept for stabilizing other class I fusion proteins that undergo large-scale conformational rearrangements and contain metastable prefusion cavities, such as hMPV F and PIV F (Battles et al, 2017; Stewart-Jones et al, 2018). Mechanistically, the D486-centered region appears to play a dual functional role in both structural stabilization and small-molecule sensitivity. This region overlaps with the binding pockets of reported RSV fusion inhibitors such as VP-14637, JNJ-2408068, and GS-5806 (Presatovir), underscoring its evolutionary conservation and structural importance (Douglas et al, 2005; Mackman et al, 2015; Perron et al, 2016). Future studies combining molecular dynamics simulations and high-resolution inhibitor-binding analyses are warranted to dissect how this conserved pocket mediates the balance between conformational stability and fusion triggering (Battles et al, 2016). Moreover, the newly identified M289 substitution—though located outside the major conformationally divergent regions between prefusion and postfusion RSV F—may enhance overall prefusion robustness through long-range hydrophobic coupling, highlighting a previously underappreciated site that merits further biophysical characterization.

As a vaccine candidate, RVF-88 establishes a foundation for bivalent or multivalent formulations addressing A/B co-circulation while maintaining manufacturing feasibility. RVF-88 warrants further evaluation in aspects relevant to late-stage development, including long-term stability, batch-to-batch consistency, and formulation compatibility, which will inform potential translation into clinical testing (Beugeling et al, 2019; Ferguson et al, 2024). The present results support RVF-88 as a rationally engineered prefusion F antigen, demonstrating that a comparatively low-mutation design can yield a stable antigen with preserved prefusion epitopes, providing a feasible basis for next-generation multivalent development. As prior work has reported that engineered disulfides can substantially enhance RSV F prefusion stability (Che et al, 2023), our data suggest a complementary stabilization modality: hydrophobic cavity-filling substitutions associated with improved core packing, disulfides and proline substitutions preferentially linked to enhanced thermal resilience, and strategically placed electrostatic substitutions appearing to support stability during ambient storage. A combinatorial design integrating all four modalities delivered the balanced gains across thermal and storage conditions, supporting its suitability as a vaccine immunogen for further development. Beyond the light-lock strategy, RVF-88-based engineered antigens incorporating additional engineered disulfide bonds or proline substitutions may further improve prefusion stability, particularly thermal robustness, and may inform future optimization of RSV preF immunogens for in vivo-expression platforms, including adenoviral vectors and mRNA vaccine systems.

## Methods

**Table:** Reagents and tools table

Reagent/resource	Reference or source	Identifier or catalog number
**Experimental models**		
BALB/cJGpt	GemPharmatech Co., Ltd (Guangdong, China)	N000020
Cotton Rat	Envigo RMS	N/A
HEK293T	Laboratory stock	N/A
HEK293F	Laboratory stock	N/A
HEp-2 cells	Laboratory stock	N/A
RSV A2	Laboratory stock	N/A
RSV B9320	Laboratory stock	N/A
CHO-K1 cells	Canton Biologics Co., Ltd.	N/A
**Recombinant DNA**		
pcDNA3.1(+) expression vector	GenScript	N/A
Antibody heavy-chain expression plasmids pcDNA3.4(+)	This study	N/A
Antibody light-chain expression plasmids pcDNA3.4(+)	This study	N/A
Codon-optimized RSV F gene, RSV A2 strain	GenScript	GenBank: P03420
Codon-optimized RSV F gene, RSV-A ON1 lineage	GenScript	GenBank: AEQ98747
Codon-optimized RSV F gene, RSV-B BA9 lineage	GenScript	GenBank: BBL36060
Codon-optimized RSV N gene, RSV-B lineage	GenScript	GenBank: AY353550
**Antibodies**		
HRP-conjugated 6*His, His-Tag Monoclonal antibody	Proteintech	HRP-66005
Goat Anti-Mouse IgG (H+L) HRP	GLGBIO	EA0102
Goat Anti-Human IgG Secondary Antibody (HRP)	Sino Biological	SSA002
Palivizumab	TargetMol Chemicals Inc.	188039-54-5
anti-Cotton Rat IgG h+l Antibody (Chicken) - HRP Conjugated	Immunology Consultants Laboratory	CCOT-25P
RSV FITC-conjugated antibody	Genetex	GTX36375
**Oligonucleotides and other sequence-based reagents**		
primers for qPCR	This study	“Methods”
**Chemicals, enzymes, and other reagents**		
ABRYSVO	PFIZER	04150187389187
DMEM	Gibco	C11995500CP
RPMI-1640 medium	Cytiva	SH30819.01
SMM 293-TII Expression medium	Sino Biological	M293TII
Fetal bovine serum (FBS)	Sigma	F0193
Penicillin-streptomycin	Gibco	15140-122
L-Glutamine	Gibco	25030081
HEPES	Gibco	15630-080
Trypsin-EDTA (0.25%), phenol red	Gibco	25200-072
ELISA coating buffer (10x)	Solarbio	C1055
PBST buffer (10x, pH 7.4)	Biosharp	BL314B
1x DPBS	Biosharp	BL310A
PBS buffer	Laboratory stock	N/A
Bovine serum albumin (BSA)	ABcone	B24726
Single-component TMB substrate	Solarbio	PR1200
ELISA stop solution	Solarbio	C1058; Lot 240005004
Triton X-100	BioFroxx	1139
4% paraformaldehyde (PFA)	Biosharp	BL539A
Nulen PlusTrans transfection reagent	Shanghai Nulen Biotechnology Co., Ltd.	CT801
Polyethylenimine (PEI)	Yeasen Biotechnology (Shanghai) Co., Ltd	MW25000
High Affinity Ni-Charged Resin FF	GenScript	L00666
Protein A affinity column	GenScript	L00210
0.22 μm sterile syringe filters	Biosharp	BS-PES25-22-S
imidazole	Aladdin	I108706-500g
SYPRO Orange dye	Sigma-Aldrich	S5692
CM5 sensor chip	Cytiva/GE Healthcare	N/A
EDC/NHS amine coupling reagents	Cytiva	N/A
Ethanolamine	N/A	N/A
Tween-20	Solarbio	T8221
Quantifoil R1.2/1.3 300 mesh copper grids with thin carbon support film	Quantifoil	N/A
Whatman No. 1 filter paper	Whatman	1001
TRIzol reagent	Invitrogen	15596018CN
Chloroform	Guangzhou Chemical Reagent	GD10
Absolute ethanol	Guangzhou Chemical Reagent	HB15
Isopropanol	Guangzhou Chemical Reagent	HC16
HiScript II One Step qRT-PCR SYBR Green Kit	Vazyme	Q221
BD Mouse IFN-gamma ELISpot Kit/Set	BD Biosciences	551083
BD ELISPOT AEC Substrate Set	BD Biosciences	551951
PMA + Ionomycin	Dakewe Biotech	2030421
Mouse spleen lymphocyte isolation kit	Tianjin Haoyang Biotechnology Co., Ltd.	LTS1092PK
SDS-PAGE protein loading buffer (5x)	Guangdong Eyou Biopharma Co., Ltd.	LB3-0001
Prestained protein marker	Guangdong Eyou Biopharma Co., Ltd.	N6617
YoungPAGE protein precast gel, 4-12%	GenScript	M01002
MOPS running buffer	GenScript	M00680-500
96-well ELISA plate	NEST Biotechnology	514201
96-well Clear Flat Bottom TC-treated Microplate	CORNING	3585
Disposable cell counting chamber	Watson	177-112C
Peroxidase Labeling Kit - NH_2_	Dojindo	LK11
ClonExpress ® Ultra One Step Cloning Kit	Vazyme	C115
2 × Phanta Flash Master Mix	Vazyme	P510
Agar	BioFroxx	8211GR500
Yeast extract	ThermoScientific	LP0021
Tryptone	ThermoScientific	LP0042
Glycerol	Biosharp	56-81-5
Superose 6 Increase 10/300 GL column	Cytiva	
Endofree Maxi Plasmid Kit	TIANGEN	DP117
TIANprep Rapid Mini Plasmid Kit	TIANGEN	DP105
**Software**		
PyMOL	Schrödinger	Version 2.6.2
CCP4	Collaborative Computational Project No. 4	Version 8.0.015
UNICORN	Cytiva	Version 7.5
GraphPad Prism	GraphPad Software	Version 10.1.2
BeStSel	ELTE Eötvös Loránd University	https://bestsel.elte.hu/index.php
SEDFIT	SEDFIT	Version 16.1c
Biacore Insight Evaluation Software	Cytiva/GE Healthcare	Version 4.0.8
EPU	Thermo Fisher Scientific	N/A
cryoSPARC	Structura Biotechnology	Version 4.7.1
UCSF Chimera	UCSF	Version 1.17
Coot	Coot	Version 1.3.1
PHENIX	PHENIX	Version 2.0
UCSF ChimeraX	UCSF	Version 1.12
BioRender	BioRender	https://www.biorender.com
**Other**		
Microplate reader	Thermo Fisher Scientific	Multiskan FC
Low-temperature incubator	YAMATO	IN812C
ELISpot reader	CTL	S6 Entry Analyzer
Far-UV circular dichroism (CD) spectra	Applied Photophysics Chirascan	V100
ProteomeLab XL-I analytical ultracentrifuge	Beckman Coulter	ProteomeLab XL-I
AN-60 Ti rotor	Beckman Coulter	AN-60 Ti
Biacore 8K instrument	GE Healthcare/Cytiva	Biacore 8K
Vitrobot	Thermo Fisher Scientific	N/A
Bio-Rad real-time PCR system	Bio-Rad	CFX96 Touch
AKTA pure protein purification system	Cytiva	N/A
PDB structures	RCSB PDB	4JHW/4MMU/7UJA
NanoDrop	Thermo Scientific	840-317400
Applied Biosystems QuantStudio 3	Thermo Scientific	A28137

### Ethics statements

All animal experiments involving mice and cotton rats were approved by the Ethics Committee of Guangdong Laboratory Animals Monitoring Institute (GZLAB-AUCP-2022-10-A01) and grouped randomly. All live RSV experiments took place under BSL-2 containment at Guangzhou National Laboratory (GZNL). All procedures were carried out in strict accordance with the recommendations outlined in the Guide for the Care and Use of Laboratory Animals.

### Structural comparison and segment classification

To quantitatively assess the conformational differences between the prefusion (PDB: 4JHW) and postfusion (PDB: 3RRR) states of RSV F, SSM-based structural superpositions were performed for seven functionally defined segments using the SUPERPOSE module implemented in CCP4 (version 8.0.015) (Krissinel and Henrick, 2004; Agirre et al, 2023). For each segment, the number of structurally equivalent residues used in the least-squares fit (Nalign), the coverage (Nalign/L, where L is the total number of residues in the segment), the backbone RMSD over aligned residues, and the SSM quality factor Q were recorded. Segments were classified as similar regions when all four of the following criteria were simultaneously satisfied: (1) SSM superposition succeeded; (2) coverage ≥0.90; (3) backbone RMSD ≤ 1.50 Å; and (4) Q ≥ 0.02. Segments failing any one of these criteria, or for which SSM superposition could not be achieved due to mismatched secondary structure, were classified as different regions. Complete alignment statistics for all segments are provided in Appendix Table S1.

### Cloning, expression, and purification of antibodies and proteins

The codon-optimized wild-type RSV F gene (A2 strain backbone, accession no. P03420, ON1 strain backbone, accession no. AEQ98747, or BA9 strain backbone, accession no. BBL36060) was synthesized by GenScript and cloned into the pcDNA3.1 expression vector. These constructs contain three naturally occurring amino acid substitutions (P102A, I379V, and M447V) to improve protein expression, and incorporates an N-terminal Kozak sequence and C-terminal tags (T4 foldon, 6×His, and StrepII) (Frank et al, 2001; McLellan et al, 2011, 2013b). Mutant constructs were subsequently generated from this template by site-directed mutagenesis PCR. Plasmid DNA was prepared using endotoxin-free kits (TIANGEN) prior to transfection. The RSV postF construct was engineered by deleting the fusion peptide (residues 137–145), following previously described ΔFP postfusion designs (McLellan et al, 2011), and by removing the C-terminal T4 fibritin foldon trimerization motif.

For small-scale expression screening, HEK293T cells were seeded in 96-well microplates (CORNING) and transiently transfected with the constructs using Nulen PlusTrans Transfection Reagent (Nulen Biotechnology Co., Ltd, Shanghai). Cells were maintained at 37 °C with 5% CO₂ in a humidified incubator, and culture supernatants were collected 3 days post-transfection. A portion of each supernatant was immediately analyzed by ELISA to compare prefusion-specific antigenicity and total F expression, while the remaining aliquots were subjected to stability testing, either heat-treated at 55 °C for 1 h or stored at 4 °C for 1 week before further analysis, consistent with established RSV F screening protocols (McLellan et al, 2013a) to facilitate discrimination of relative variant stability during early-stage screening.

For large-scale protein production, the constructs were transiently transfected into HEK293F cells. Supernatants were harvested 5 days post-transfection, and soluble proteins were purified by nickel affinity chromatography using self-packed High Affinity Ni-Charged Resin FF (GenScript, 2 ml bed volume) followed by size-exclusion chromatography (SEC) using a Superose 6 Increase 10/300 GL column (Cytiva) equilibrated in phosphate-buffered saline (PBS) on ÄKTA pure system (Cytiva), and data were collected using UNICORN 7.5 software. Protein purity was verified by SDS-PAGE.

For antibody expression, heavy- and light-chain sequences of D25, CR9501, 101 F, MPE8, motavizumab, AM14, and 01.4B were cloned into the pcDNA3.4 expression vector and transiently transfected into HEK293F cells with polyethylenimine (PEI). Culture supernatants were collected 7 days post-transfection and applied to Protein A affinity columns (GenScript). After washing with 1× PBS, bound antibodies were eluted with 0.1 M glycine-HCl (pH 2.7–3.0) and immediately neutralized with 1 M Tris-HCl (pH 8.0). Antibodies were further polished by SEC on a Superose 6 Increase 10/300 GL column in 1× PBS, and final purity was confirmed by SDS-PAGE. The monoclonal antibody palivizumab was purchased from Targetmol.

### Cell lines, viruses, and animals

RSV A2 and B9320 strains were propagated in HEp-2 cells maintained in Dulbecco’s modified Eagle’s medium (DMEM, Gibco) supplemented with 2% fetal bovine serum (FBS, Sigma) and 1% penicillin–streptomycin (p/s, Gibco) at 37 °C in 5% CO₂. Viral stocks were harvested upon >50% cytopathic effect, clarified by centrifugation, aliquoted, and stored at −80 °C. Viral titers were determined by fluorescent focus assay in HEp-2 cells.

HEK293T cells were used primarily for transient transfection, and were cultured in DMEM (Gibco) supplemented with 10% heat-inactivated fetal bovine serum and 1% (v/v) penicillin–streptomycin at 37 °C with 5% CO₂. Suspension-adapted HEK293F cells were employed for large-scale recombinant protein expression and maintained in SMM 293-TII (Sino Biological) at 37 °C with 5% CO₂ under shaking conditions. CHO-K1 cells were adapted to serum-free suspension culture and served as a platform for stable protein production and process development. All cell lines were authenticated and confirmed free of mycoplasma contamination prior to use.

Female BALB/c mice (6–8 weeks old) were obtained from GuangDong GemPharmatech Co., Ltd. Female cotton rats (6–8 weeks old) were obtained from Envigo RMS LLC. Mice and cotton rats were housed at the Laboratory Animal Center of Guangdong Laboratory. All animals were housed under specific pathogen-free (SPF) conditions with ad libitum access to water and a standard chow diet. Environmental parameters were controlled with a 12-h light/dark cycle, a temperature range of 20–25 °C, and a relative humidity maintained at 40–70%.

### Enzyme-linked immunosorbent assay (ELISA)

ELISAs were performed to evaluate the binding of RSV F variants to monoclonal antibodies or sera. For screening ELISA, 96-well plates (NEST) were coated overnight at 4 °C with prefusion-specific antibody D25 or the broadly reactive antibody motavizumab (2 µg ml^−1^, 100 µL per well) in coating buffer (Solarbio). For serum binding (indirect) ELISA, plates were coated overnight at 4 °C with 2 µg ml^−1^ of purified RVF-88A or RVF-88B as the coating antigens for the respective assays. Plates were washed three times with 1× PBST (PBS containing 0.05% Tween-20; 120 rpm, 3 min) and blocked with 200 µL of 2% BSA (ABcone) at 37 °C for 2 h. For screening assays, cell culture supernatants harvested 3 days post-transfection of HEK293T cells expressing RSV F mutants were added (100 µL per well) and incubated at 37 °C for 1 h. For binding assays, monoclonal antibodies were applied in fivefold or tenfold serial dilutions. Tenfold serial dilutions were initiated from 10 μg/mL. Fivefold serial dilutions were performed with antibody-specific starting concentrations as follows: D25 (20 μg/mL), CR9501 (100 μg/mL), 101F (4 μg/mL), MPE8 (4 μg/mL), motavizumab (0.8 μg/mL), palivizumab (4 μg/mL), AM14 (100 μg/mL), and 01.4B (4 μg/mL). Sera were applied starting at a 1:100 dilution followed by tenfold serial dilutions (100 µL per well), and incubated at 37 °C for 1 h. After washing, HRP-conjugated secondary antibodies, including goat anti-6×His tag antibody (Proteintech), goat anti-human IgG, goat anti-rabbit IgG, goat anti-mouse IgG (GLGBIO), or chicken anti-cotton rat IgG (Immunology Consultants Laboratory), all at 1:5000 dilution, were added and incubated at 37 °C for 1 h. Plates were washed three times with 1× PBST after each incubation step, developed with 100 µL of TMB substrate (Solarbio) for 10 min at room temperature, and reactions were terminated with 50 µL of stop solution. Absorbance at 450 nm (OD_450_) was measured using a microplate reader (Thermo Scientific Multiskan FC).

For all ELISA-based screening assays, binding signals were normalized to the concurrently processed wild-type RSV F (A2 strain) control within each experimental batch, and the results are expressed as log_2_-transformed fold changes relative to wild-type F. This within-batch normalization was applied to minimize the influence of batch-to-batch variation inherent to transient transfection-based expression and to enable direct comparison of antigenicity across independently conducted screening rounds.

### Size-exclusion chromatography (SEC) for protein stability assessment

SEC was used to assess the homogeneity and stability of candidate proteins under multiple stress conditions on a Superose 6 Increase column (Cytiva) with 1× PBS as the mobile phase. Prior to injection, all samples were centrifuged at 12,000 rpm for 5 min; no visible precipitate was observed under any tested condition.

For the initial stability screen of HEK293F-expressed constructs, samples formulated in 1× PBS were subjected to one freeze–thaw cycle, three freeze–thaw cycles, ambient storage for 7 days (RT-7D), or heat challenge at 55 °C for 1 h. Freeze–thaw cycles were performed by thawing at room temperature until fully liquefied before re-freezing at −80 °C.

For both accelerated and long-term stability assessment of CHO-expressed RVF-88A and RVF-88B, proteins were derived from a single purified batch, aliquoted, and stored at −80 °C until allocation to their respective storage conditions. All samples were formulated in 20 mM phosphate buffer and buffer-exchanged into 1× PBS prior to SEC analysis. Accelerated stability samples were stored at 4 °C for 6 months for RVF-88A and at −20 °C for 6 months for RVF-88B (A-6M), as the accelerated stability condition for RVF-88B was set at −20 °C based on its formulation characteristics. Long-term stability samples were stored at −80 °C for 6 months (L-6M) and 12 months (L-12M). SEC analyses for all time points and conditions were performed in a single analytical session to ensure direct comparability and eliminate inter-session instrument variability.

### Differential scanning fluorimetry (DSF)

The thermal stability (Tm) of purified proteins was determined by differential scanning fluorimetry (DSF). Purified protein at a final concentration of 0.1 mg ml^−1^ was mixed with SYPRO Orange dye (1:1000, Sigma-Aldrich) in a 96-well optical qPCR plate. All protein dilutions were prepared in PBS. Measurements were performed on a Bio-Rad real-time PCR instrument with a temperature ramp from 25 to 95 °C, increasing by 0.5 °C every 30 s with continuous fluorescence acquisition. Melting curves and their first derivatives (-dF/dT) were generated using the instrument software (Melt Curve Derivative Results), and the apparent melting temperature (T_m_) was defined as the temperature at the peak of the -dF/dT curve. Data were replotted in GraphPad Prism (version 10.1.2) for visualization.

### Surface plasmon resonance (SPR)

SPR experiments were performed on a Biacore 8 K instrument (Cytiva) at room temperature using 1× PBS, pH 7.4, 0.05% Tween-20 as running buffer. A CM5 chip (Cytiva) was prepared by EDC/NHS activation and coupling of Protein A (100 µg ml^−1^, 10 mM acetate pH 5.0) to 150 RU, followed by ethanolamine quenching. RSV F-specific monoclonal antibodies were captured to ~100 RU on the analytical flow cell; the reference cell lacked capture. Antigens (RVF-88A, RVF-88B, pXCS847A) were injected at 1.5625–200 nM (twofold series) at 30 µL/min with 60 s association and 180 s dissociation under multicycle kinetics. Surfaces were regenerated with 10 mM glycine-HCl (pH 1.5, 30 s). Sensorgrams were processed by double referencing, and binding kinetics (k_a_: Association rate constant, k_d_: Dissociation rate constant, K_D_: Equilibrium dissociation constant calculated as k_d_/k_a_) were determined by global fitting to a 1:1 Langmuir model using Biacore Insight Evaluation Software (v4.0.8) (Appendix Table S3).

### Circular dichroism (CD) spectroscopy

Far-UV circular dichroism (CD) spectra were recorded on a circular dichroism spectrometer (Applied Photophysics Chirascan V100). Protein samples of RVF-88A, RVF-88B, pXCS847A, and postF-A were prepared at 0.1–0.2 mg ml^−1^ in 1× PBS buffer (pH 7.4). For the stability studies, CD measurements were performed immediately upon completion of each storage period to capture the conformational state at that time point. Measurements were performed in a path-length quartz cuvette over a wavelength range of 190–260 nm. Spectra were acquired with a bandwidth of 1 nm and a data pitch of 1 nm, and were averaged over two accumulations. Baseline spectra of buffer alone were subtracted, and data were converted to mean residue ellipticity [θ] (deg·cm^2^·dmol^−1^). Secondary structure composition (α-helix, β-sheet, and random coil fractions) was estimated using the BeStSel algorithms (Kardos et al, 2025).

### Analytical ultracentrifugation (AUC)

Sedimentation velocity analytical ultracentrifugation (SV-AUC) was performed on a ProteomeLab XL-I instrument (Beckman Coulter) to characterize the conformational state and oligomeric distribution of RSV F variant proteins. Protein samples (380 μL, A₂₈₀ = 0.6–0.8) and matching buffer controls (400 μL of 1× PBS, pH 7.4) were loaded into 12 mm double-sector epoxy centerpieces equipped with sapphire windows. Centrifugation was performed in an AN-60 Ti rotor at 25,000 rpm and 20 °C for 12 h, with absorbance scans collected at 280 nm at 1 min intervals. Data were analyzed using the continuous c(s) distribution model in SEDFIT (v16.1c, NIBIB/NIH) (Schuck et al, 2002), which enabled resolution of distinct sedimenting species, estimation of their molecular weights, and assessment of conformational integrity and potential aggregation.

### Immunization and challenge studies in mice and cotton rats

Female BALB/c mice (6–8 weeks old) were immunized intramuscularly at weeks 0 and 3 with RVF-88 (2, 5, or 12.5 μg; 1:1 mixture by mass of RVF-88A and RVF-88B), ABRYSVO (5 μg; obtained from PFIZER CORPORATION HONG KONG LIMITED, Cat#04150187389187, lot#HK3115 and LK1864), or buffer as a placebo control. Serum samples were collected at weeks 0, 3, and 5, heat-inactivated at 56 °C for 30 min, and analyzed for antigen-specific and neutralizing antibody responses by ELISA and live-virus neutralization assays. At week 5, splenocytes were isolated for ELISpot analysis. For protection studies, mice were intranasally challenged 2 weeks after the second immunization with RSV A2 (10⁶ PFU), and lungs were harvested on day 5 post-challenge for viral load and viral titer quantification; body weight was monitored daily. For durability studies, additional mice received two doses of 5 μg RVF-88, and sera were collected at weeks 0, 3, 5, 7, 9, 13, 18, and 22 for antibody analyses.

In parallel, female cotton rats (6 weeks old) were immunized at weeks 0 and 3 with buffer, RVF-88 (24 μg), or ABRYSVO (24 μg). Serum antibody responses were assessed as described above. For challenge studies, cotton rats were intranasally infected 4 weeks after the second immunization with RSV A2 (10⁶ PFU) or RSV B9320 (3 × 10⁵ PFU). Body weight was monitored daily, and lung and nasal turbinate tissues were collected on day 4 post-challenge for viral quantification. Half of the right lung lobes and nasal turbinates were weighed, homogenized in 1 ml sterile 1× PBS, and used for viral load and viral titer quantification. The intact left lung was fixed in 4% paraformaldehyde, processed for hematoxylin and eosin (H&E) staining, and examined microscopically for histopathological changes.

### Neutralization assays

Neutralizing antibody titers were determined using a cytopathic effect (CPE)-based assay. Serum samples collected from immunized mice or cotton rats were heat-inactivated at 56 °C for 30 min and serially diluted threefold in DMEM supplemented with 2% fetal bovine serum (FBS) and 1% penicillin–streptomycin. Equal volumes of diluted sera were mixed with 2666 TCID_50_ ml^−1^ of RSV A (A2 strain) or 40000 TCID_50_ ml^−1^ of RSV B (B9320 strain) and incubated at 33 °C for 2 h to allow neutralization.

The virus-serum mixtures (150 µL per well) were then added to wells containing 3 × 10⁴ HEp-2 cells per well, followed by culture at 33 °C in a 5% CO₂ incubator for 3–5 days. Each serum sample was tested in duplicate wells. Virus control wells (virus without serum) and cell control wells (cells only) were included in each assay. The development of virus-induced CPE was monitored microscopically, and the neutralizing antibody titer was defined as the highest serum dilution that completely inhibited CPE in 50% of replicate wells compared with the virus control.

### ELISpot

Antigen-specific IFN-γ-secreting splenocytes were quantified using a commercial ELISPOT kit (BD™ Mouse IFN-γ ELISPOT Set, BD Biosciences) strictly following the manufacturer’s instructions. Briefly, single-cell suspensions were prepared from mouse spleens and plated onto IFN-γ capture antibody–coated ELISPOT plates supplied with the kit. Cells were stimulated ex vivo with a pooled set of four peptides from the respiratory syncytial virus (RSV) F protein (TYMLTNSELL (Khan et al, 2019), KYKNAVTEL (Schmidt et al, 2018), ELQLLMQSTPPTNNR (Durant et al, 2014), VSVLTSKVLDLKNYI (Castilow et al, 2008)), while PBS served as the negative control. After antigen challenge, plates were processed according to the kit protocol to develop IFN-γ spots. Spot-forming units (SFU) were enumerated on an automated ELISPOT reader (CTL S6 ENtry Analyzers) with uniform image analysis settings across plates.

### RNA extraction and quantitative real-time PCR (qRT–PCR)

Lung tissues (~0.1 g) were homogenized in grinding tubes, and 250 μL of the homogenate was mixed with 750 μL of TRIzol reagent (Invitrogen). The mixture was vortexed vigorously, followed by the addition of 150 μL of chloroform, and vigorous mixing. The samples were incubated at room temperature for 10 min and then centrifuged at 12,000×*g* for 15 min at 4 °C. The aqueous phase was carefully transferred to a new tube, and RNA was precipitated by adding an equal volume of isopropanol. The samples were incubated at room temperature for 10 min, followed by centrifugation at 12,000×g for 10 min at 4 °C. The RNA pellet was washed with 75% ethanol, air-dried, and dissolved in RNase-free water. RNA concentration and purity were determined using a NanoDrop spectrophotometer (Thermo Fisher Scientific).

For quantification of RSV viral load, qRT–PCR was performed using the Vazyme Q221 kit. RSV F (RSV A2) and RSV N (RSV B9320) gene copy numbers were quantified on QuantStudio™ 3 RealTime PCR Detection System (Applied Biosystems), with plasmid constructs containing wild-type RSV F gene (RSV A2, accession no. P03420) and RSV N gene (RSV B9320, GenBank: AY353550) cloned into the pcDNA3.1 vector used to generate standard curves. Primer sequences used for qRT-PCR analysis are listed below: RSV A2 F gene forward primer, 5′-CGAGCCAGAAGAGAACTACCA-3′; RSV A2 F gene reverse primer, 5′-CCTTCTAGGTGCAGGACCTTA-3′; RSV B N gene forward primer, 5′-TGCTAGTGTCCAGGCAGAAA-3′; RSV B N gene reverse primer, 5′-TGCCTAGGACCACACTTGAG-3′. The amplification was performed as follows: 50 °C for 15 min, 95 °C for 5 min followed by 40 cycles of 95 °C for 10 s and 60 °C for 30 s. Melt curve analysis was subsequently performed at 95 °C for 15 s, 60 °C for 60 s, and 95 °C for 15 s. Viral loads were expressed on a log_10_ scale as viral copies per gram, using standard curves generated from plasmid DNA.

### Lung and nasal turbinate viral titration via fluorescent focus assay

Lung or nasal turbinate tissue samples from infected mice or cotton rats were homogenized and then serially diluted fourfold in DMEM containing 2% FBS and 1% penicillin–streptomycin. The diluted samples were then added to wells containing 3 × 10⁴ HEp-2 cells per well and cultured at 33 °C with 5% CO₂ for 24 h. Each sample was tested in duplicate wells. Subsequently, 100 μL of 4% paraformaldehyde (Biosharp) was added to each well for viral fixation, followed by a 12-h incubation at room temperature. The wells were then washed with 1× PBS, and permeabilization was performed using 100 μL of 0.3% Triton-X100 solution (BioFroxx) per well for 15 min at room temperature. After washing, blocking was carried out with 100 μL of 5% BSA-PBS per well for 2 h at room temperature. Respiratory virus antibody-FITC (1:100 dilution, Genetex) was added to each well (100 μL per well) and incubated overnight at 4 °C. After 1 h of room temperature incubation, the wells were washed three times with 1× PBS (3 min per wash), and the number of fluorescent foci in each well was observed under a fluorescence inverted microscope.

The number of positive foci was recorded, and lung viral titers were calculated using the following formula: Lung viral titer (per gram of tissue)=(fluorescent focus count×corresponding dilution factor)/0.1×(1/lung tissue weight). A well was considered positive if the fluorescent focus count was ≥10. For the maximum dilution, the fluorescent focus count from positive wells was used for calculation. Wells with <10 fluorescent foci were considered negative, and their counts were used for baseline statistical analysis. The limit of detection for viral titer assays was defined based on assay sensitivity (1 FFU detection threshold) and adjusted using species-specific minimum tissue weights.

### H&E staining and histopathological scoring

Fresh lung tissues were fixed in 4% paraformaldehyde for at least 24 h, trimmed, and embedded in paraffin following a standard protocol of graded ethanol dehydration, xylene clearing, and paraffin infiltration. Paraffin blocks were sectioned at 4 μm using a rotary microtome, mounted on glass slides, and dried at 60 °C. Sections were deparaffinized in xylene, rehydrated through a graded ethanol series, and stained with Harris hematoxylin for nuclear visualization, followed by differentiation in acid alcohol, bluing in ammonia water, and counterstaining with eosin for cytoplasmic components. After dehydration, clearing, and mounting with neutral resin, sections were examined under a light microscope, and representative images were recorded.

Histopathological changes were evaluated by examining inflammatory infiltration in the airways, blood vessels, and interstitial spaces. Lesions were semi-quantitatively scored according to the severity and extent of lung involvement as follows: 1, minimal inflammation, with less than 20% of lung tissue affected; 2, mild inflammation, with 20–40% of lung tissue affected; 3, moderate inflammation, with 40–60% of lung tissue affected; 4, marked inflammation, with more than 60% of lung tissue affected; and 5, severe inflammation, characterized by extensive tissue damage or structural disruption. Scoring was performed in a blinded manner by two independent investigators.

### Cryo-EM sample preparation, data collection, and structure determination

Complexes of RSV F protein with the D25 Fab were prepared at a final concentration of 0.8 mg ml^−1^ (molar ratio 1:1) and incubated at 4 °C for 30 min. Quantifoil R1.2/1.3 300-mesh copper grids coated with a thin carbon support film were glow-discharged immediately prior to use. A 4 μL aliquot of the complex was applied to each grid, blotted for 3.5 s with Whatman No. 1 filter paper at 100% humidity, and vitrified using a Vitrobot (Thermo Fisher Scientific). Cryo-EM grids were screened, and data were collected on a Titan Krios G4 electron microscope (Thermo Fisher) operated at 300 kV, equipped with a Falcon 4 direct electron detector in counting mode. Images were recorded at a nominal magnification of ×165,000, corresponding to a calibrated pixel size of 0.73 Å, with a total exposure of ~50 e⁻/Å² and an exposure rate of ~10 e⁻/Å²/s using EPU (Thermo Fisher) for automated acquisition. As shown in Appendix Figs. S16 and S17, movies were motion-corrected and CTF parameters estimated in cryoSPARC (Structura Biotechnology) (Punjani et al, 2017). Particles were picked, Fourier-cropped to a 192-pixel box, and subjected to 2D classification; selected particles were then re-extracted at the original pixel size (0.73 Å/pixel, 384-pixel box) for ab initio reconstruction and heterogeneous refinement. The best class was used for homogeneous refinement followed by non-uniform refinement (NU-refinement) (Punjani et al, 2020) in cryoSPARC, which yielded the final reconstructions at overall resolutions of 2.96 Å for RVF-88A and 2.77 Å for RVF-88B, according to the gold-standard FSC 0.143 criterion (Appendix Table S6).

Model building was performed by docking the previously reported RSV F prefusion structure (PDB ID: 7UJA) into the cryo-EM maps using UCSF Chimera. Manual adjustments were carried out in Coot (Emsley et al, 2010), followed by real-space refinement in PHENIX (Liebschner et al, 2019) with default parameters. Figures were prepared using UCSF Chimera (Pettersen et al, 2004) and ChimeraX (Meng et al, 2023).

### Crystallization analysis

For crystallization, 1 µL of RVF-88A or RVF-88B (5 mg/mL) was mixed with 1 μL of reservoir solution containing 140 mM potassium formate, 14% PEG3350, 60 mM magnesium chloride hexahydrate, 30 mM Tris hydrochloride pH 8.5, 9% Polyethylene glycol 4000. The crystal of the RVF-88B suitable for diffraction were obtained in a condition containing 70 mM HEPES pH 7.5, 14% (w/v) PEG4000, 7% (v/v) isopropanol, 30 mM magnesium formate dihydrate, 4.5% (w/v) polyethylene glycol 3350. Single crystals were carefully harvested and were soaked in a well solution supplemented with 20% (v/v) ethylene glycol as a cryoprotectant for 10 s before plunge freezing in liquid nitrogen.

X-ray data were collected from beamline BL18U1 at the Shanghai Synchrotron Radiation Facility (SSRF) at 100 K. Data integration and scaling were performed using XDSgui (Kursula, 2004; Kabsch, 2010). The structure was determined by molecular replacement with the Phaser module in PHENIX using the prefusion RSV F (RCSB Protein Data Bank code: 7KQD) as a search template (McCoy et al, 2007; Adams et al, 2010). The output model was subjected to iterative cycles of manual model adjustment using Coot (Emsley and Cowtan, 2004), and refinement was conducted using PHENIX (Appendix Table S7).

### Statistical analysis

Statistical analyses were performed using GraphPad Prism 10.1.2 software. Data are presented as mean ± SD unless otherwise indicated. Animals were randomly assigned to experimental groups. The number of biological replicates or independent experiments is indicated in the corresponding figure legends. Comparisons among multiple groups were performed using one-way ANOVA with Tukey’s multiple comparisons test. Statistical significance was defined as follows: ns not significant, *P < 0.05, **P < 0.01, ***P < 0.001, ****P < 0.0001.

## Supplementary information

Appendix

Peer Review File

Source data Fig. 2

Source data Fig. 3

Source data Fig. 4

Source data Fig. 5

## Data Availability

The cryo-EM structures of RVF-88A–D25 Fab and RVF-88B–D25 Fab, together with the antibody-free crystal structures of RVF-88A and RVF-88B, have been deposited in the Protein Data Bank (PDB) under accession codes 28DJ, 27RI, 25XQ, and 25XR, respectively. Corresponding cryo-EM maps have been deposited in the Electron Microscopy Data Bank (EMDB) under accession codes EMD-81660 and EMD-81358. Previously reported structures used for comparative analysis, including 4JHW, 3RRR, 4MMU, and 7UJA, were obtained from the PDB. The source data of this paper are collected in the following database record: biostudies:S-SCDT-10_1038-S44321-026-00511-8.

## References

[CR2] Adams PD, Afonine PV, Bunkóczi G, Chen VB, Davis IW, Echols N, Headd JJ, Hung L-W, Kapral GJ, Grosse-Kunstleve RW et al (2010) PHENIX: a comprehensive Python-based system for macromolecular structure solution. Acta Cryst D 66:213–221 10.1107/S0907444909052925 PMC2815670 20124702

[CR3] Agirre J, Atanasova M, Bagdonas H, Ballard CB, Baslé A, Beilsten-Edmands J, Borges RJ, Brown DG, Burgos-Mármol JJ, Berrisford JM et al (2023) The CCP4 suite: integrative software for macromolecular crystallography. Acta Cryst D 79:449–461 10.1107/S2059798323003595 PMC10233625 37259835

[CR4] Battles MB, Langedijk JP, Furmanova-Hollenstein P, Chaiwatpongsakorn S, Costello HM, Kwanten L, Vranckx L, Vink P, Jaensch S, Jonckers THM et al (2016) Molecular mechanism of respiratory syncytial virus fusion inhibitors. Nat Chem Biol 12:87–93 10.1038/nchembio.1982 PMC4731865 26641933

[CR5] Battles MB, Más V, Olmedillas E, Cano O, Vázquez M, Rodríguez L, Melero JA, McLellan JS (2017) Structure and immunogenicity of pre-fusion-stabilized human metapneumovirus F glycoprotein. Nat Commun 8:1528 10.1038/s41467-017-01708-9 PMC5688127 29142300

[CR6] Beugeling M, Amssoms K, Cox F, De Clerck B, Van Gulck E, Verwoerd JA, Kraus G, Roymans D, Baert L, Frijlink HW et al (2019) Development of a stable respiratory syncytial virus pre-fusion protein powder suitable for a core-shell implant with a delayed release in mice: a proof of concept study. Pharmaceutics 11:510 10.3390/pharmaceutics11100510 PMC6835792 31623333

[CR7] Boukhvalova MS, Prince GA, Blanco JCG (2009) The cotton rat model of respiratory viral infections. Biologicals 37:152–159 10.1016/j.biologicals.2009.02.017 PMC2882635 19394861

[CR8] Cantú-Flores K, Rivera-Alfaro G, Muñoz-Escalante JC, Noyola DE (2022) Global distribution of respiratory syncytial virus A and B infections: a systematic review. Pathog Glob Health 116:398–409 10.1080/20477724.2022.2038053 PMC9518599 35156555

[CR9] Castilow EM, Olson MR, Meyerholz DK, Varga SM (2008) Differential role of gamma interferon in inhibiting pulmonary eosinophilia and exacerbating systemic disease in fusion protein-immunized mice undergoing challenge infection with respiratory syncytial virus. J Virol 82:2196–2207 10.1128/JVI.01949-07 PMC2258940 18094193

[CR10] Che Y, Gribenko AV, Song X, Handke LD, Efferen KS, Tompkins K, Kodali S, Nunez L, Prasad AK, Phelan LM et al (2023) Rational design of a highly immunogenic prefusion-stabilized F glycoprotein antigen for a respiratory syncytial virus vaccine. Sci Transl Med 15:eade6422 10.1126/scitranslmed.ade6422 37023209

[CR11] Douglas JL, Panis ML, Ho E, Lin K-Y, Krawczyk SH, Grant DM, Cai R, Swaminathan S, Chen X, Cihlar T (2005) Small molecules VP-14637 and JNJ-2408068 inhibit respiratory syncytial virus fusion by similar mechanisms. Antimicrob Agents Chemother 49:2460–2466 10.1128/AAC.49.6.2460-2466.2005 PMC1140497 15917547

[CR12] Durant LR, Pereira C, Boakye A, Makris S, Kausar F, Goritzka M, Johansson C (2014) DNGR-1 is dispensable for CD8+ T-cell priming during respiratory syncytial virus infection. Eur J Immunol 44:2340–2348 10.1002/eji.201444454 24777856

[CR13] Emsley P, Cowtan K (2004) Coot: model-building tools for molecular graphics. Acta Cryst D 60:2126–2132 10.1107/S0907444904019158 15572765

[CR14] Emsley P, Lohkamp B, Scott WG, Cowtan K (2010) Features and development of Coot. Acta Cryst D 66:486–501 10.1107/S0907444910007493 PMC2852313 20383002

[CR15] Ferguson M, Murray A, Pliamm L, Rombo L, Sanmartin Berglund J, David M-P, De Schrevel N, Maschino F, Kotb S, Olivier A et al (2024) Lot-to-lot immunogenicity consistency of the respiratory syncytial virus prefusion F protein vaccine in older adults. Vaccin X 18:100494 10.1016/j.jvacx.2024.100494 PMC11090873 38746060

[CR16] Flynn JA, Durr E, Swoyer R, Cejas PJ, Horton MS, Galli JD, Cosmi SA, Espeseth AS, Bett AJ, Zhang L (2016) Stability characterization of a vaccine antigen based on the respiratory syncytial virus fusion glycoprotein. PLoS ONE 11:e0164789 10.1371/journal.pone.0164789 PMC5072732 27764150

[CR17] Frank S, Kammerer RA, Mechling D, Schulthess T, Landwehr R, Bann J, Guo Y, Lustig A, Bächinger HP, Engel J (2001) Stabilization of short collagen-like triple helices by protein engineering1. J Mol Biol 308:1081–1089 10.1006/jmbi.2001.4644 11352592

[CR18] Gidwani SV, Brahmbhatt D, Zomback A, Bassie M, Martinez J, Zhuang J, Schulze J, McLellan JS, Mariani R, Alff P et al (2024) Engineered dityrosine-bonding of the RSV prefusion F protein imparts stability and potency advantages. Nat Commun 15:2202 10.1038/s41467-024-46295-8 PMC10940300 38485927

[CR19] Hashimoto K, Hosoya M (2017) Neutralizing epitopes of RSV and palivizumab resistance in Japan. Fukushima J Med Sci 63:127–134 10.5387/fms.2017-09 PMC5792496 28867684

[CR20] Huang Q, Lang Q, Li Y, Wang F, Han X, Luo L, Duan X, Cao X, Wang Y, Bai Y et al (2025) Highly scalable prefusion-stabilized RSV F vaccine with enhanced immunogenicity and robust protection. Nat Commun 16:7805 10.1038/s41467-025-63084-z PMC12370985 40841372

[CR21] Kabsch W (2010) XDS. Acta Cryst D 66:125–132 10.1107/S0907444909047337 PMC2815665 20124692

[CR22] Kardos J, Nyiri MP, Moussong É, Wien F, Molnár T, Murvai N, Tóth V, Vadászi H, Kun J, Jamme F et al (2025) Guide to the structural characterization of protein aggregates and amyloid fibrils by CD spectroscopy. Protein Sci 34:e70066 10.1002/pro.70066 PMC11836901 39968955

[CR23] Khan IU, Ahmad F, Zhang S, Lu P, Wang J, Xie J, Zhu N (2019) Respiratory syncytial virus F and G protein core fragments fused to HBsAg-binding protein (SBP) induce a Th1-dominant immune response without vaccine-enhanced disease. Int Immunol 31:199–209 10.1093/intimm/dxy078 30462215

[CR24] Krarup A, Truan D, Furmanova-Hollenstein P, Bogaert L, Bouchier P, Bisschop IJM, Widjojoatmodjo MN, Zahn R, Schuitemaker H, McLellan JS et al (2015) A highly stable prefusion RSV F vaccine derived from structural analysis of the fusion mechanism. Nat Commun 6:8143 10.1038/ncomms9143 PMC4569726 26333350

[CR25] Krissinel E, Henrick K (2004) Secondary-structure matching (SSM), a new tool for fast protein structure alignment in three dimensions. Acta Cryst D 60:2256–2268 10.1107/S0907444904026460 15572779

[CR26] Kursula P (2004) XDSi: a graphical interface for the data processing program XDS. J Appl Crystallogr 37:347–348

[CR27] Langedijk AC, Vrancken B, Lebbink RJ, Wilkins D, Kelly EJ, Baraldi E, Mascareñas de Los Santos AH, Danilenko DM, Choi EH, Palomino MA et al (2024) The genomic evolutionary dynamics and global circulation patterns of respiratory syncytial virus. Nat Commun 15:3083 10.1038/s41467-024-47118-6 PMC11006891 38600104

[CR28] Lee Y-Z, Han J, Zhang Y-N, Ward G, Braz Gomes K, Auclair S, Stanfield RL, He L, Wilson IA, Zhu J (2024) Rational design of uncleaved prefusion-closed trimer vaccines for human respiratory syncytial virus and metapneumovirus. Nat Commun 15:9939 10.1038/s41467-024-54287-x PMC11569192 39550381

[CR29] Li J, Ma X, Xu Z, Guo W, Peng R, Zhang Y, Meng Y, Zhao J, Wang Q, Li S et al (2025) Rational design of respiratory syncytial virus dimeric F-subunit vaccines in protein and mRNA forms. eBioMedicine 119:105902 10.1016/j.ebiom.2025.105902 PMC12410573 40886405

[CR30] Li Y, Wang X, Blau DM, Caballero MT, Feikin DR, Gill CJ, Madhi SA, Omer SB, Simões EAF, Campbell H et al (2022) Global, regional, and national disease burden estimates of acute lower respiratory infections due to respiratory syncytial virus in children younger than 5 years in 2019: a systematic analysis. Lancet 399:2047–2064 10.1016/S0140-6736(22)00478-0 PMC7613574 35598608

[CR31] Liebschner D, Afonine PV, Baker ML, Bunkóczi G, Chen VB, Croll TI, Hintze B, Hung L-W, Jain S, McCoy AJ et al (2019) Macromolecular structure determination using X-rays, neutrons and electrons: recent developments in Phenix. Acta Cryst D 75:861–877 10.1107/S2059798319011471 PMC6778852 31588918

[CR32] Lin M, Yin Y, Zhao X, Wang C, Zhu X, Zhan L, Chen L, Wang S, Lin X, Zhang J et al (2025) A truncated pre-F protein mRNA vaccine elicits an enhanced immune response and protection against respiratory syncytial virus. Nat Commun 16:1386 10.1038/s41467-025-56302-1 PMC11799228 39910047

[CR33] Mackman RL, Sangi M, Sperandio D, Parrish JP, Eisenberg E, Perron M, Hui H, Zhang L, Siegel D, Yang H et al (2015) Discovery of an oral respiratory syncytial virus (RSV) fusion inhibitor (GS-5806) and clinical proof of concept in a human RSV challenge study. J Med Chem 58:1630–1643 10.1021/jm5017768 25574686

[CR35] McCoy AJ, Grosse-Kunstleve RW, Adams PD, Winn MD, Storoni LC, Read RJ (2007) Phaser crystallographic software. J Appl Crystallogr 40:658–674 10.1107/S0021889807021206 PMC2483472 19461840

[CR36] McLellan JS, Chen M, Joyce MG, Sastry M, Stewart-Jones GBE, Yang Y, Zhang B, Chen L, Srivatsan S, Zheng A et al (2013a) Structure-based design of a fusion glycoprotein vaccine for respiratory syncytial virus. Science 342:592–598 10.1126/science.1243283 PMC4461862 24179220

[CR37] McLellan JS, Chen M, Leung S, Graepel KW, Du X, Yang Y, Zhou T, Baxa U, Yasuda E, Beaumont T et al (2013b) Structure of RSV fusion glycoprotein trimer bound to a prefusion-specific neutralizing antibody. Science 340:1113–1117 10.1126/science.1234914 PMC4459498 23618766

[CR38] McLellan JS, Ray WC, Peeples ME (2013c) Structure and Function of Respiratory Syncytial Virus Surface Glycoproteins. In: Anderson LJ, Graham BS (eds) Challenges and opportunities for respiratory syncytial virus vaccines. Springer, Berlin, Heidelberg, pp 83–104 10.1007/978-3-642-38919-1_4 PMC4211642 24362685

[CR39] McLellan JS, Yang Y, Graham BS, Kwong PD (2011) Structure of respiratory syncytial virus fusion glycoprotein in the postfusion conformation reveals preservation of neutralizing epitopes. J Virol 85:7788–7796 10.1128/JVI.00555-11 PMC3147929 21613394

[CR40] Meng EC, Goddard TD, Pettersen EF, Couch GS, Pearson ZJ, Morris JH, Ferrin TE (2023) UCSF ChimeraX: Tools for structure building and analysis. Protein Sci 32:e4792 10.1002/pro.4792 PMC10588335 37774136

[CR41] Ngwuta JO, Chen M, Modjarrad K, Joyce MG, Kanekiyo M, Kumar A, Yassine HM, Moin SM, Killikelly AM, Chuang G-Y et al (2015) Prefusion F–specific antibodies determine the magnitude of RSV neutralizing activity in human sera. Sci Transl Med 7:309ra162 10.1126/scitranslmed.aac4241 PMC4672383 26468324

[CR42] Oraby AK, Stojic A, Elawar F, Bilawchuk LM, McClelland RD, Erwin K, Granoski MJ, Griffiths CD, Frederick JD, Arutyunova E et al (2025) A single amino acid mutation alters multiple neutralization epitopes in the respiratory syncytial virus fusion glycoprotein. NPJ Viruses 3:33 10.1038/s44298-025-00119-8 PMC12015481 40295799

[CR43] Perron M, Stray K, Kinkade A, Theodore D, Lee G, Eisenberg E, Sangi M, Gilbert BE, Jordan R, Piedra PA et al (2016) GS-5806 inhibits a broad range of respiratory syncytial virus clinical isolates by blocking the virus-cell fusion process. Antimicrob Agents Chemother 60:1264–1273 10.1128/AAC.01497-15 PMC4776015 26666922

[CR44] Pettersen EF, Goddard TD, Huang CC, Couch GS, Greenblatt DM, Meng EC, Ferrin TE (2004) UCSF Chimera—A visualization system for exploratory research and analysis. J Comput Chem 25:1605–1612 10.1002/jcc.20084 15264254

[CR45] Punjani A, Rubinstein JL, Fleet DJ, Brubaker MA (2017) cryoSPARC: algorithms for rapid unsupervised cryo-EM structure determination. Nat Methods 14:290–296 10.1038/nmeth.4169 28165473

[CR46] Punjani A, Zhang H, Fleet DJ (2020) Non-uniform refinement: adaptive regularization improves single-particle cryo-EM reconstruction. Nat Methods 17:1214–1221 10.1038/s41592-020-00990-8 33257830

[CR47] Schmidt ME, Knudson CJ, Hartwig SM, Pewe LL, Meyerholz DK, Langlois RA, Harty JT, Varga SM (2018) Memory CD8 T cells mediate severe immunopathology following respiratory syncytial virus infection. PLoS Pathog 14:e1006810 10.1371/journal.ppat.1006810 PMC5766251 29293660

[CR48] Schuck P, Perugini MA, Gonzales NR, Howlett GJ, Schubert D (2002) Size-distribution analysis of proteins by analytical ultracentrifugation: strategies and application to model systems. Biophys J 82:1096–1111 10.1016/S0006-3495(02)75469-6 PMC1301916 11806949

[CR49] Sefat KMSR, Kulkarni R, Trinh J, Leekha A, Kumar M, Wu H, McBride T, Aideyan L, Avadhanula V, Piedra PA et al (2025) Mucosal vaccines with STING-agonist liposomal formulations inhibit RSV (respiratory syncytial virus) replication in cotton rats. Vaccine 56:127183 10.1016/j.vaccine.2025.127183 40318351

[CR50] Song Q, Yang H, Zhu H, Hu Y, Shen W, Cheng H, Cai J, Qiu M, Li Y, Li Y et al (2025) Hydrophobic residue substitutions enhance the stability and in vivo immunogenicity of respiratory syncytial virus fusion protein. J Virol 99:e00087-25 10.1128/jvi.00087-25 PMC12172430 40434102

[CR51] Stewart-Jones GBE, Chuang G-Y, Xu K, Zhou T, Acharya P, Tsybovsky Y, Ou L, Zhang B, Fernandez-Rodriguez B, Gilardi V et al (2018) Structure-based design of a quadrivalent fusion glycoprotein vaccine for human parainfluenza virus types 1–4. Proc Natl Acad Sci USA 115:12265–12270 10.1073/pnas.1811980115 PMC6275507 30420505

[CR52] Sun Y, Liu L, Qiang H, Sun H, Jiang Y, Ren L, Jiang Z, Lei S, Chen L, Wang Y et al (2024) A potent broad-spectrum neutralizing antibody targeting a conserved region of the prefusion RSV F protein. Nat Commun 15:10085 10.1038/s41467-024-54384-x PMC11582626 39572535

[CR53] van Erp EA, Luytjes W, Ferwerda G, van Kasteren PB (2019) Fc-mediated antibody effector functions during respiratory syncytial virus infection and disease. Front Immunol 10:548 10.3389/fimmu.2019.00548 PMC6438959 30967872

[CR54] Zhang L, Durr E, Galli JD, Cosmi S, Cejas PJ, Luo B, Touch S, Parmet P, Fridman A, Espeseth AS et al (2018) Design and characterization of a fusion glycoprotein vaccine for respiratory syncytial virus with improved stability. Vaccine 36:8119–8130 10.1016/j.vaccine.2018.10.032 30340881

