# Supplementary material for: A cavity-reduced prefusion RSV F bivalent vaccine elicits durable and protective immunity
Source: EMBO Mol Med. 2026 Aug 29;18(10):3979–4000. doi: 10.1038/s44321-026-00511-8 (PMC13658822; doi:10.1038/s44321-026-00511-8)
Supplement: Supplementary file 1 [file 44321_2026_511_MOESM1_ESM.pdf]

Appendix

Appendix Figure S1. Structure-guided stabilization strategies and design of RSV F protein variants. (Page 3)

Appendix Figure S2. Antigenicity of hydrophobic cavity-filling F protein variants. (Page 5)

Appendix Figure S3. Antigenicity of selected substitutions compared with DS, Cav1 and DS-Cav1. (Page 6)

Appendix Figure S4. Protein stability of multi-strategy combination mutants of N67L-V90Y-S190I-M289F. (Page 7)

Appendix Figure S5. ELISA binding of amino acid substitutions at positions of RVF-88A to D25 antibody. (Page 9)

Appendix Figure S6. Structural and antigenic stability of RVF-88A and RVF-88B under long-term storage. (Page 11)

Appendix Figure S7. ELISA binding profiles of RVF-88A, RVF-88B, and pXCS847A to a panel of monoclonal antibodies. (Page 13)

Appendix Figure S8. SPR binding profiles of RVF-88A to monoclonal antibodies. (Page 14)

Appendix Figure S9. SPR binding profiles of RVF-88B to monoclonal antibodies. (Page 15)

Appendix Figure S10. SPR binding profiles of pXCS847A to monoclonal antibodies. (Page 16)

Appendix Figure S11. Histological analysis of lung tissue in BALB/c mice following immunization and viral challenge. (Page 17)

Appendix Figure S12. ELISpot analysis of IFN-γ responses in BALB/c mice following RVF-88 and control immunization. (Page 18)

Appendix Figure S13. Evaluation of long-term antibody responses in BALB/c mice immunized with bivalent RVF-88. (Page 19)

Appendix Figure S14. Histological analysis of lung tissue in cotton rats following immunization and viral challenge. (Page 20)

Appendix Figure S15. Crystal structures and cavity analysis of RVF-88. (Page 21)

Appendix Figure S16. The single-particle cryo-EM workflow and analysis of RVF-88A in complex with D25 Fab. (Page 23)

Appendix Figure S17. The single-particle cryo-EM workflow and analysis of RVF-88B in complex with D25 Fab. (Page 25)

Appendix Table S1. SSM-based structural alignment statistics for seven segments of RSV F comparing the prefusion (PDB: 4JHW) and postfusion (PDB: 3RRR). (Page 27)

Appendix Table S2. Region-based single-substitution screening of prioritized RSV F segments. (Page 28)

Appendix Table S3. Binding kinetics of antibody interactions with RVF-88A, RVF-88B and pXCS847A as measured by surface plasmon resonance (SPR). (Page 29)

Appendix Table S4. Serum neutralizing antibody titers in mouse and cotton rat immunogenicity studies. (Page 30)

Appendix Table S5. Summary of local cavity properties in the trimeric prefusion apex of RVF-88A and selected RSV F reference structures. (Page 31)

Appendix Table S6. Cryo-EM data collection, 3D reconstruction and refinement statistics. (Page 32)

Appendix Table S7. Crystallographic statistics and refinement statistics of RVF-88A and RVF-88B. (Page 33)

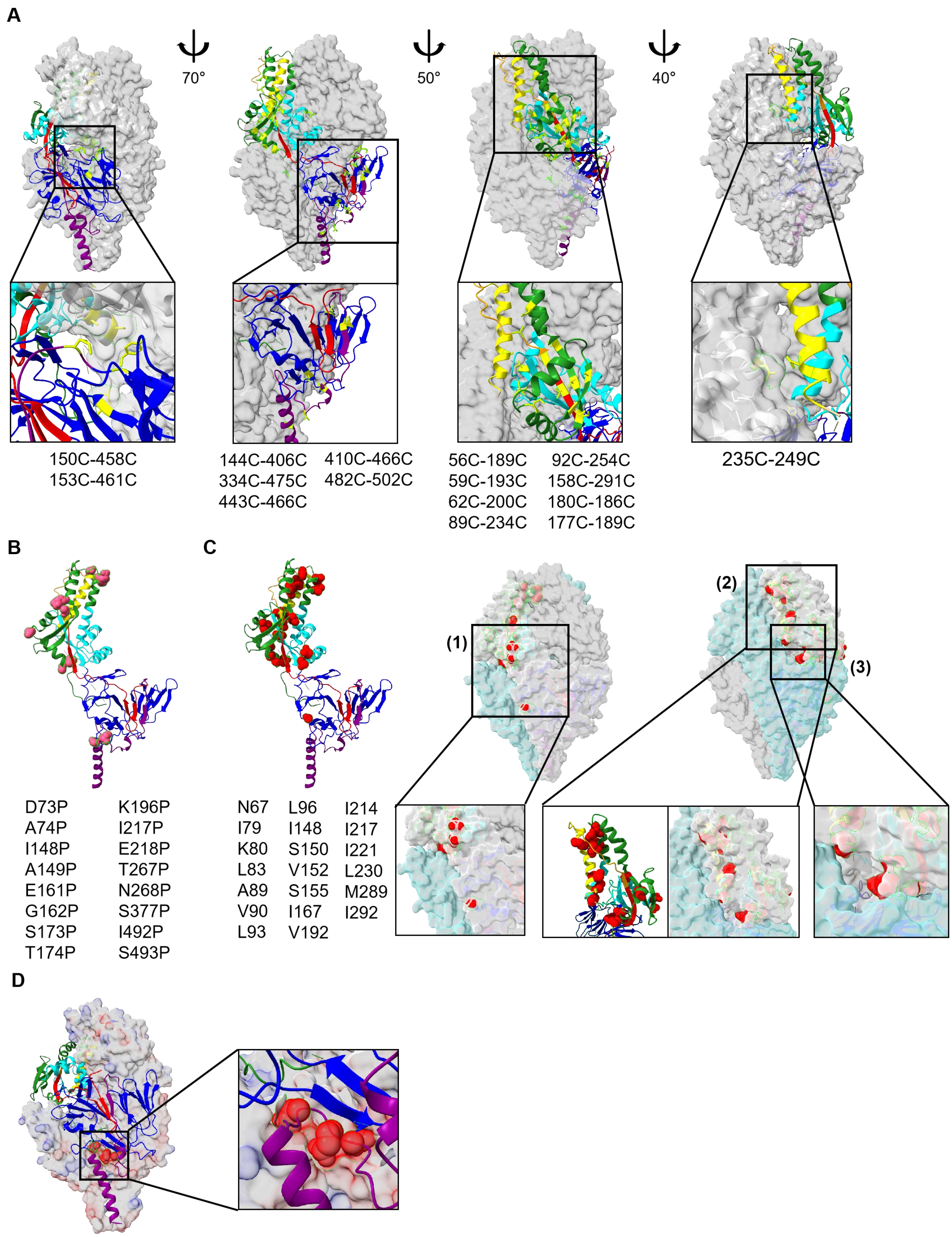
A
70°
50°
40°
150C-458C
153C-461C
144C-406C
334C-475C
443C-466C
410C-466C
482C-502C
56C-189C
59C-193C
62C-200C
89C-234C
92C-254C
158C-291C
180C-186C
177C-189C
235C-249C
B
D73P
A74P
I148P
A149P
E161P
G162P
S173P
T174P
K196P
I217P
E218P
T267P
N268P
S377P
I492P
S493P
C
N67
I79
K80
L83
A89
V90
L93
L96
I148
S150
V152
S155
I167
V192
I214
I217
I221
L230
M289
I292
(1)
(2)
(3)
D

Appendix Figure S1. Structure-guided stabilization strategies and design of RSV F protein variants.

(A) Positions of disulfide bond mutations. At least one residue involved in each engineered disulfide is located within regions of large conformational difference (aa 73–226 and 460–513). Mutated residues are shown as yellow residues and atoms/bonds with a fluorescent green outline. The leftmost and rightmost panels depict examples of inter-chain disulfide bonds. (B) Positions of proline substitutions, displayed as pink spheres with a fluorescent green outline. A total of 23 proline-substitution designs were generated, comprising 16 single-site substitutions and 7 double-proline combinatorial substitutions at adjacent positions: D73P-A74P, I148P-A149P, E161P-G162P, S173P-T174P, I217P-E218P, T267P-N268P, and I492P-S493P. Only the 16 single-site substitutions are displayed individually; double-proline pairs are indicated by their proximal positions on the structure. (C) Positions of hydrophobic cavity–filling mutations. Mutated residues are shown as red spheres with a fluorescent green outline. The substitutions were designed to fill three types of hydrophobic cavities: (1) surface-exposed cavities, (2) internal cavities in the upper head region, and (3) cavities at interprotomer interfaces between chains (blue surface). For each of the 20 targeted sites, substitutions to bulkier hydrophobic residues (Met, Leu, Ile, Phe, Tyr, His, and Trp) were screened; all seven amino acids were tested at each position with minor exceptions. (D) Positions of electrostatic charge–altering mutations. Electrostatic potential of the prefusion F trimer was calculated and mapped onto the solvent-accessible surface using the Coulombic surface coloring tool in ChimeraX. Positive regions are shown in blue, negative regions in red, and neutral regions in white. Sites of electrostatic clashes are highlighted as red spheres with a fluorescent green outline.

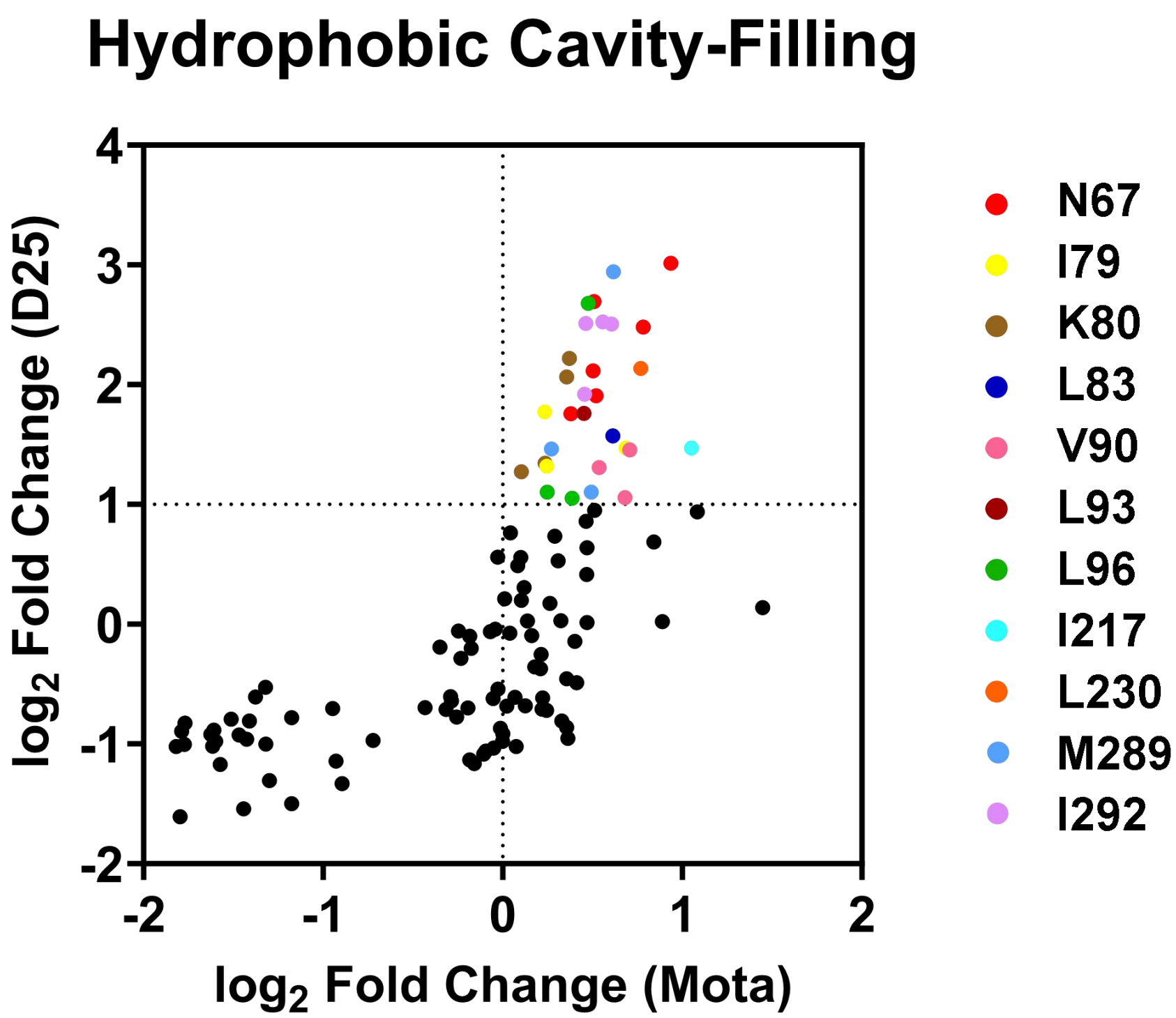

Appendix Figure S2. Antigenicity of hydrophobic cavity-filling F protein variants.

Scatter plots of ELISA OD_{450} readouts obtained from HEK293T supernatants, assayed against the prefusion-specific antibody D25 (site Ø) and motavizumab (Mota; site II of preF and postF) 72 h after transfection. Boundaries are set at x = 0 and y = 1, where y = 1 corresponds to a twofold increase in D25 binding relative to WT. Variants in the upper-right quadrant are a priori designated as favorable, indicating enhanced antigenicity (D25 signal) without loss of Mota reactivity; the remaining quadrants are prioritized, in order, upper-left, lower-right, and lower-left. Colored symbols denote shortlisted candidates and sites are listed. Data information: Data were calculated based on the mean of OD_{450} (n = 3).

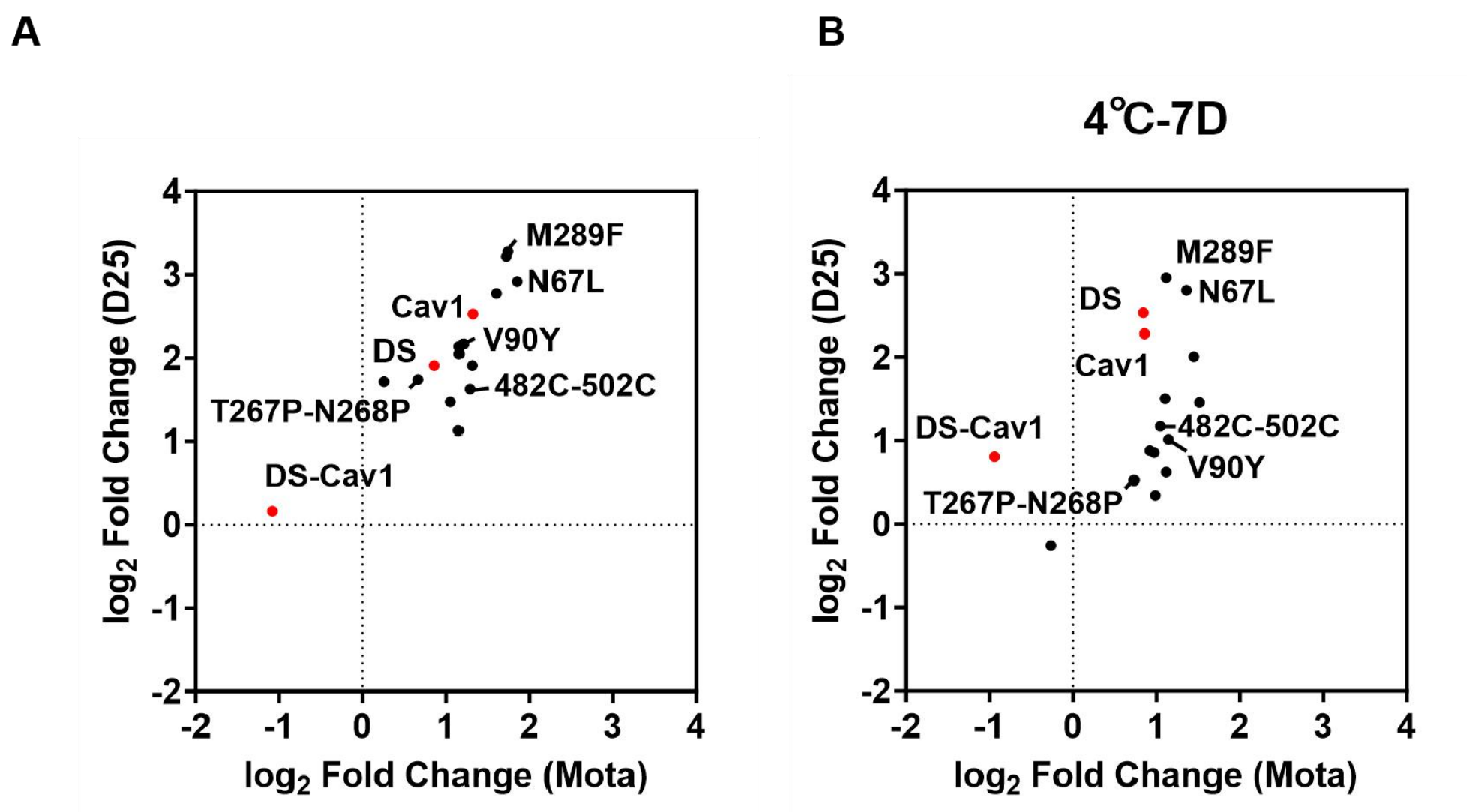

Appendix Figure S3. Antigenicity of selected substitutions compared with DS, Cav1 and DS-Cav1.

Scatter plots of ELISA OD_{450} readouts obtained from HEK293T supernatants, assayed against the prefusion-specific antibody D25 (site Ø) and motavizumab (Mota; site II of preF and postF), measured 72 h after transfection (A) and after storage at 4 °C for 7 d (B). Axes show $\log_2$-transformed mutant-to-WT ratios of OD_{450} for motavizumab (x) and D25 (y). Dashed lines indicate quadrant boundaries and selection thresholds: vertical and horizontal dashed lines are set at x = 0 and y = 0, indicating no change relative to WT. Red symbols indicate DS, Cav1 and DS-Cav1. Data information: Data are calculated based on the mean of OD_{450} (n = 3).

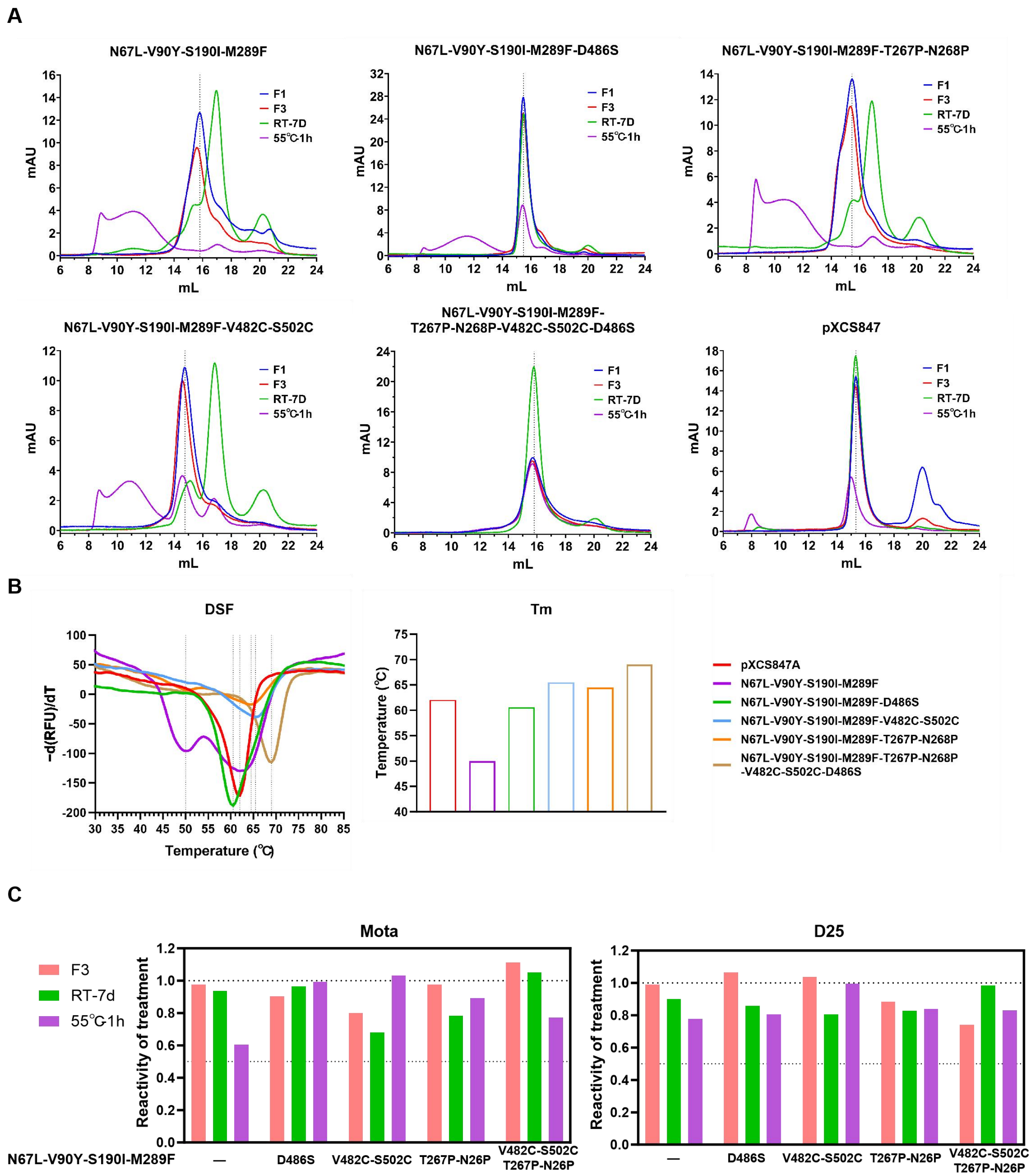

Appendix Figure S4. Protein stability of multi-strategy combination mutants of N67L-V90Y-S190I-M289F.

(A) Size-exclusion chromatography (SEC) of five combination mutants under stability challenges, shown as normalized UV_{280} chromatograms. Conditions include the baseline sample (one freeze–thaw cycle shown in blue line) and stressed samples including three freeze–thaw cycles (red line), storage at 4 °C for 7 days (green line), and heat exposure at 55 °C for 1 hour (purple line). A vertical dashed line denotes the baseline monomer-peak retention time and

serves as a reference across conditions. (B) Apparent melting temperatures (T_m) determined by differential scanning fluorimetry (DSF, left) for the five combination mutants and Pfizer construct pXCS847A. Dashed lines denote the melting temperatures of mutants, which are summarized in the bar graph (right), enabling direct comparison. (C) Bar plots show ELISA results representing the binding reactivity of five constructs to the indicated antibodies following three stress challenges: three freeze–thaw cycles (red), storage at room temperature for 7 days (green), and heat treatment at 55 °C for 1 h (purple). The y-axis indicates the mean ratio of OD_{450} values measured after treatment relative to those measured before treatment (post/pre). Data information: Data are calculated based on the mean of OD_{450} (n = 3).

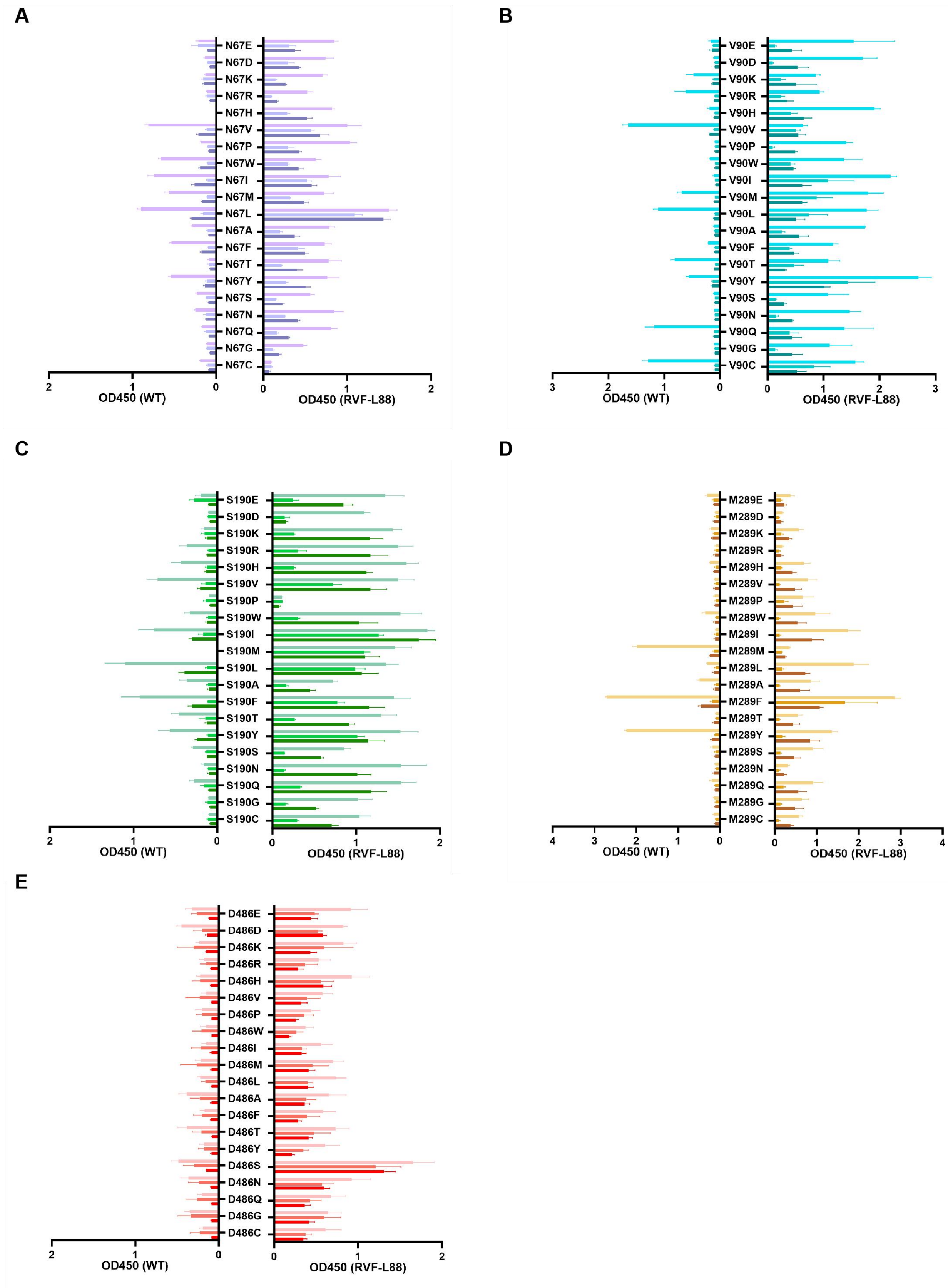

A
N67E
N67D
N67K
N67R
N67H
N67V
N67P
N67W
N67I
N67M
N67L
N67A
N67F
N67T
N67Y
N67S
N67N
N67Q
N67G
N67C
2
1
0
0
1
2
OD450 (WT)
OD450 (RVF-L88)
B
V90E
V90D
V90K
V90R
V90H
V90V
V90P
V90W
V90I
V90M
V90L
V90A
V90F
V90T
V90Y
V90S
V90N
V90Q
V90G
V90C
3
2
1
0
0
1
2
3
OD450 (WT)
OD450 (RVF-L88)
C
S190E
S190D
S190K
S190R
S190H
S190V
S190P
S190W
S190I
S190M
S190L
S190A
S190F
S190T
S190Y
S190S
S190N
S190Q
S190G
S190C
2
1
0
0
1
2
OD450 (WT)
OD450 (RVF-L88)
D
M289E
M289D
M289K
M289R
M289H
M289V
M289P
M289W
M289I
M289M
M289L
M289A
M289F
M289T
M289Y
M289S
M289N
M289Q
M289G
M289C
4
3
2
1
0
0
1
2
3
4
OD450 (WT)
OD450 (RVF-L88)
E
D486E
D486D
D486K
D486R
D486H
D486V
D486P
D486W
D486I
D486M
D486L
D486A
D486F
D486T
D486Y
D486S
D486N
D486Q
D486G
D486C
2
1
0
0
1
2
OD450 (WT)
OD450 (RVF-L88)

Appendix Figure S5. ELISA binding of amino acid substitutions at RVF-88A positions to the D25 antibody.

Bar plots represent the D25 reactivity of HEK293T cell supernatants transiently expressing F mutants. The left panel shows mutations based on wild-type (WT) F protein, while the right panel shows mutations based on RVF-88A. Panel (A) shows all amino acid substitutions at position N67 (purple), (B) at site V90 (blue), (C) at site S190 (green), (D) at site M289 (yellow), and (E) at site D486 (red). Light color bars represent samples taken 3 days post-transfection, intermediate color bars represent samples after 3 days with heat treatment at 55°C for 1 hour, and dark color bars represent samples after 3 days followed by storage at 4°C for 7 days. Data are shown as mean ± SD from independent replicates (n = 3).

A

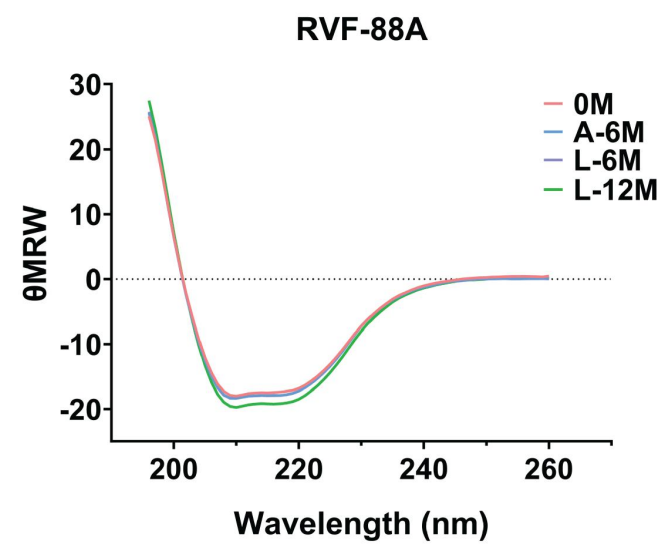

Composition of RVF-88A Secondary Structure

%	Helix	Antiparallel	Parallel	Turn	Others
0M	35	6.1	6.8	8.5	43.6
A-6M	35	9.8	3.6	7.6	43.9
L-6M	32.8	10.4	3.4	8.4	45.1
L-12M	34.4	11.1	4	7.9	42.7

B

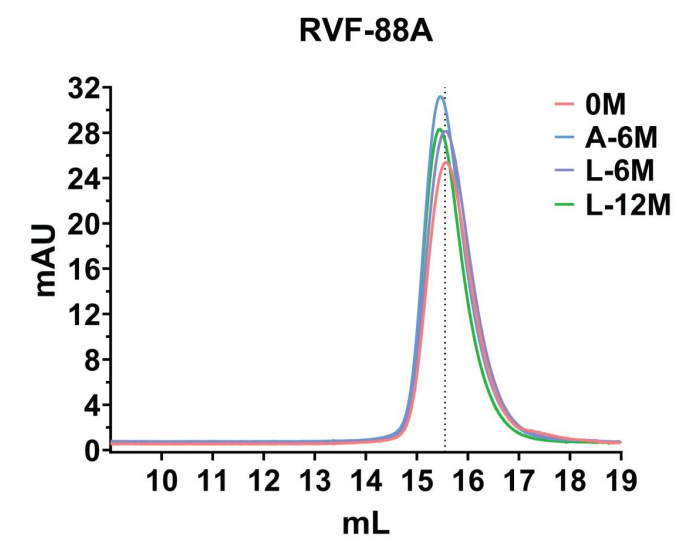

C

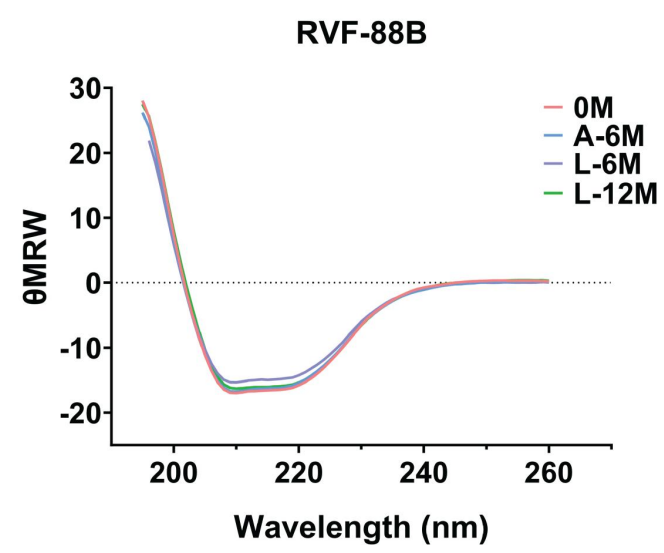

Composition of RVF-88B Secondary Structure

%	Helix	Antiparallel	Parallel	Turn	Others
0M	36.6	17.9	6	7.3	32.2
A-6M	39.2	15.8	4	7.3	33.8
L-6M	36.6	10.5	2.3	7.5	43.2
L-12M	40.9	14.9	6.4	7.3	30.4

D

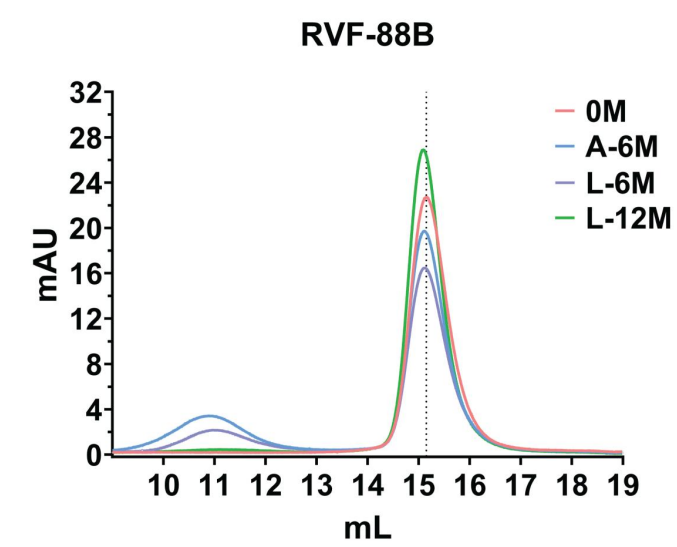

E F

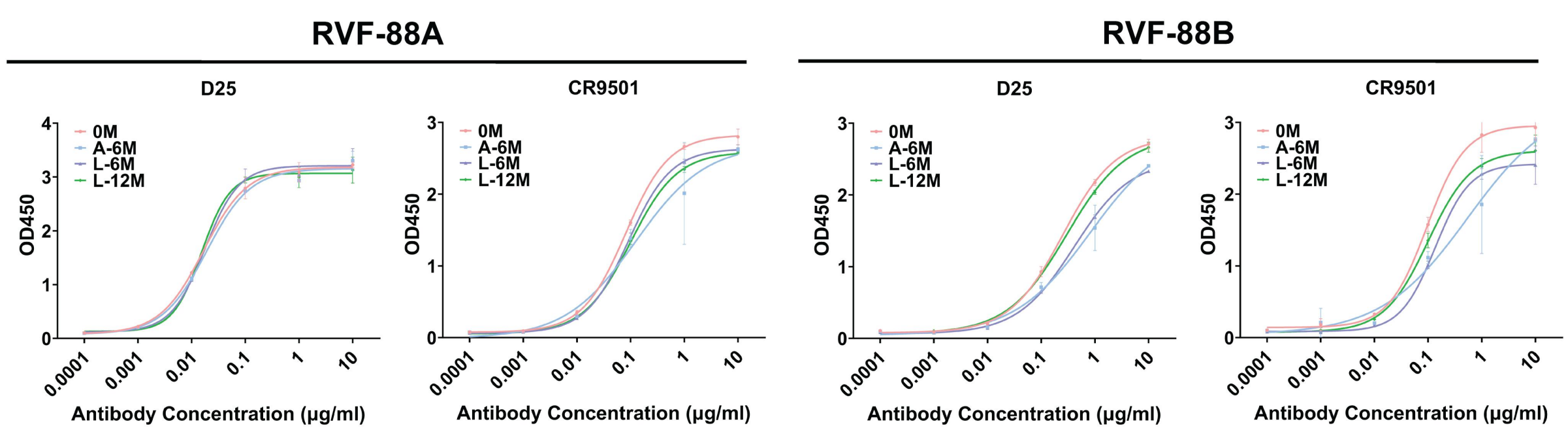

Appendix Figure S6. Structural and antigenic stability of RVF-88A and RVF-88B under long-term storage.

All samples were derived from a single purified batch and subjected to the indicated storage conditions. (A and C) Secondary structure analysis of RVF-88A stability samples (A) and RVF-88B stability samples (C) by circular dichroism (CD). Spectra (190–260 nm) are color-coded by storage condition: pink (0M, fresh untreated), blue (A-6M, accelerated stability: 4 °C for 6 months for RVF-88A, −20°C for 6 months for RVF-88B), purple (L-6M, long-term stability at –80 °C for 6 months), and green (L-12M, long-term stability at –80 °C for 12 months). Deconvolution indicates minimal changes in α/β/coil content across conditions, consistent with preservation of a prefusion-like fold. (B and D) Size-exclusion chromatography (SEC) of RVF-88A stability samples (B) and RVF-88B stability samples (D). UV_{280} traces versus elution volume show a single symmetric main peak with minimal shifts across conditions and no evident aggregate/fragment peaks in RVF-88A, indicating good solution homogeneity. For RVF-88B, panel D shows a low-intensity secondary peak/shoulder under some storage conditions, while the principal monodisperse peak remains predominant, consistent with overall preserved assembly

with minor secondary species present. (E and F) ELISA binding of RVF-88A stability samples (E) and RVF-88B stability samples (F) to monoclonal antibodies D25 (left) and CR9501 (right). Antibody concentration (x-axis) versus OD_{450} (y-axis). Pink denotes fresh, untreated; blue, 4 °C for 6 months; purple, -80 °C for 6 months; green, -80 °C for 12 months. EC_{50} values are comparable across conditions, indicating preserved antigenicity and epitope integrity over long-term storage. Data are shown as mean ± SD from independent replicates (n = 2).

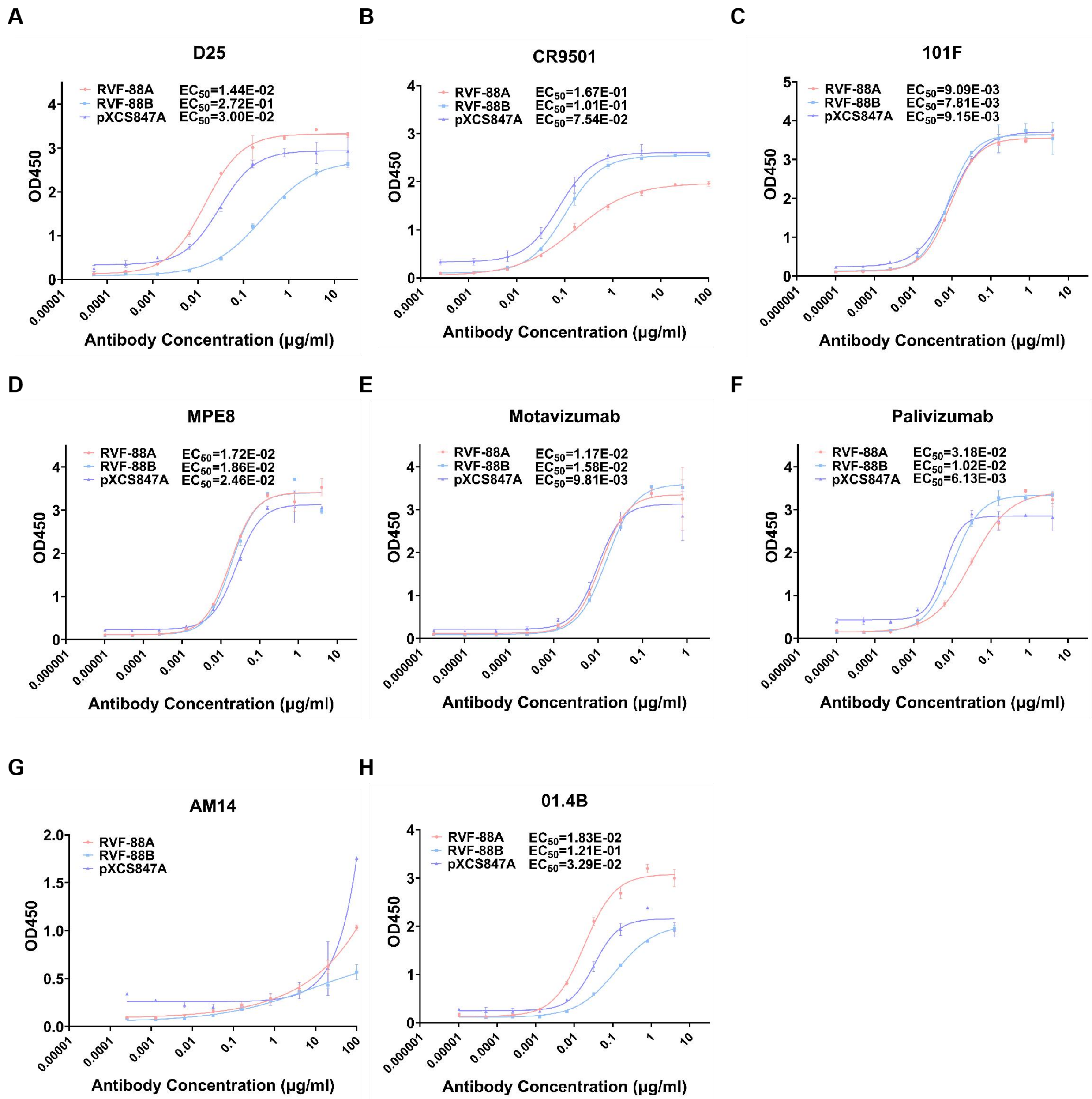

Appendix Figure S7. ELISA binding profiles of RVF-88A, RVF-88B, and pXCS847A to a panel of monoclonal antibodies.

ELISA binding curves are shown with antibody concentration on the x-axis and OD_{450} on the y-axis, with corresponding EC_{50} values (μg/ml) indicated in each panel. Panels depict binding of RVF-88A (red), RVF-88B (blue), and pXCS847A (purple) to eight monoclonal antibodies: D25 (A), CR9501 (B), 101F (C), MPE8 (D), motavizumab (E), palivizumab (F), AM14 (G), and 01.4B (H). Representative ELISA binding curves from one independent experiment are shown. Data are shown as mean ± SD of duplicate technical replicates, with fitted EC_{50} values indicated in each panel. Quantitative EC_{50} values from four independent experiments are summarized in Fig. 3B.

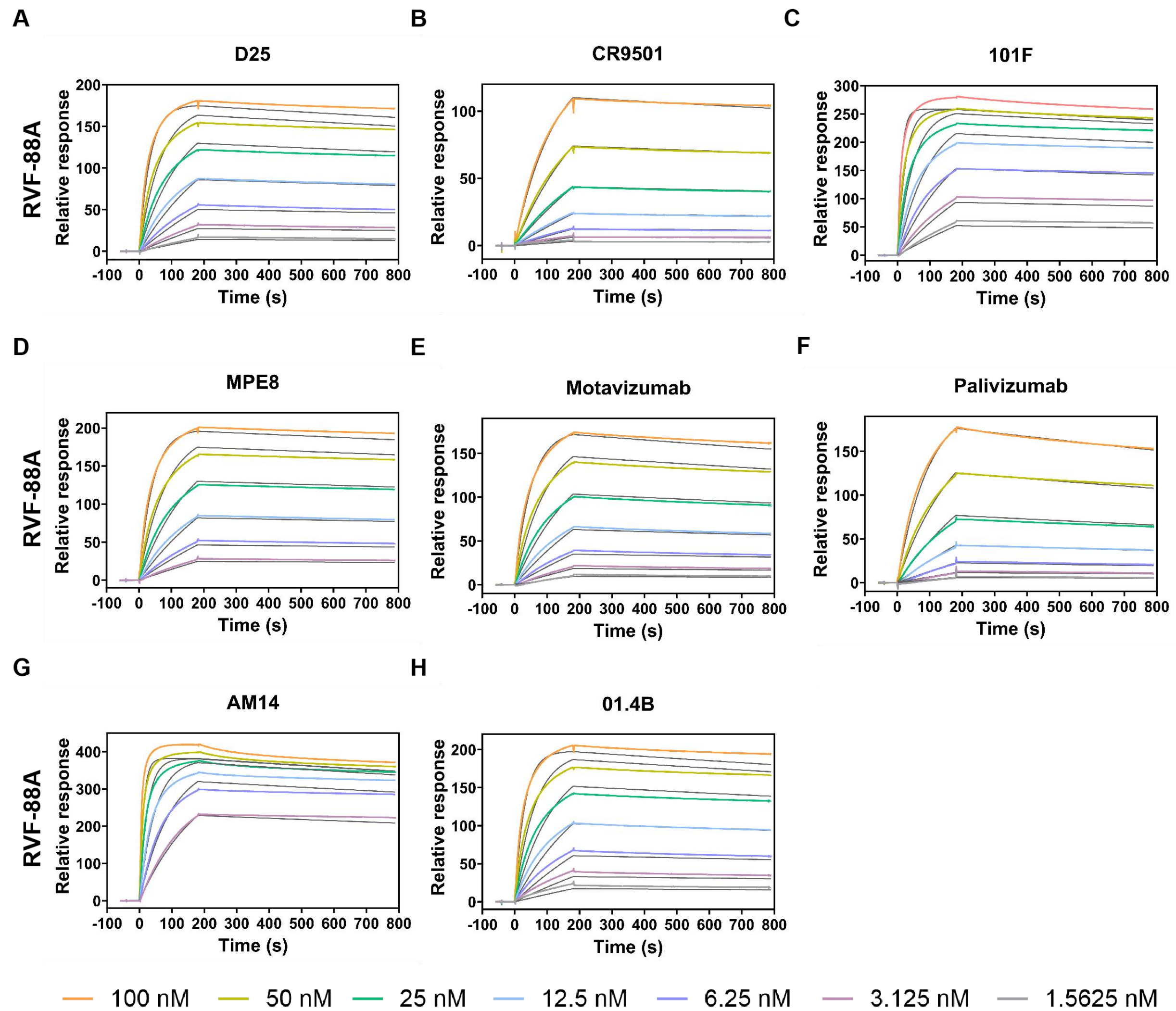

Appendix Figure S8. SPR binding profiles of RVF-88A to monoclonal antibodies.

SPR sensorgrams for RVF-88A binding to eight antibodies are shown: D25 (A), CR9501 (B), 101F (C), MPE8 (D), motavizumab (E), palivizumab (F), AM14 (G), and 01.4B (H). Colored traces represent serial analyte concentrations (100 nM to 1.5625 nM), and black curves denote global curve fitting used to derive kinetic parameters (K_D). The results demonstrate the epitope-specific binding profile of RVF-88A across the antibody panel.

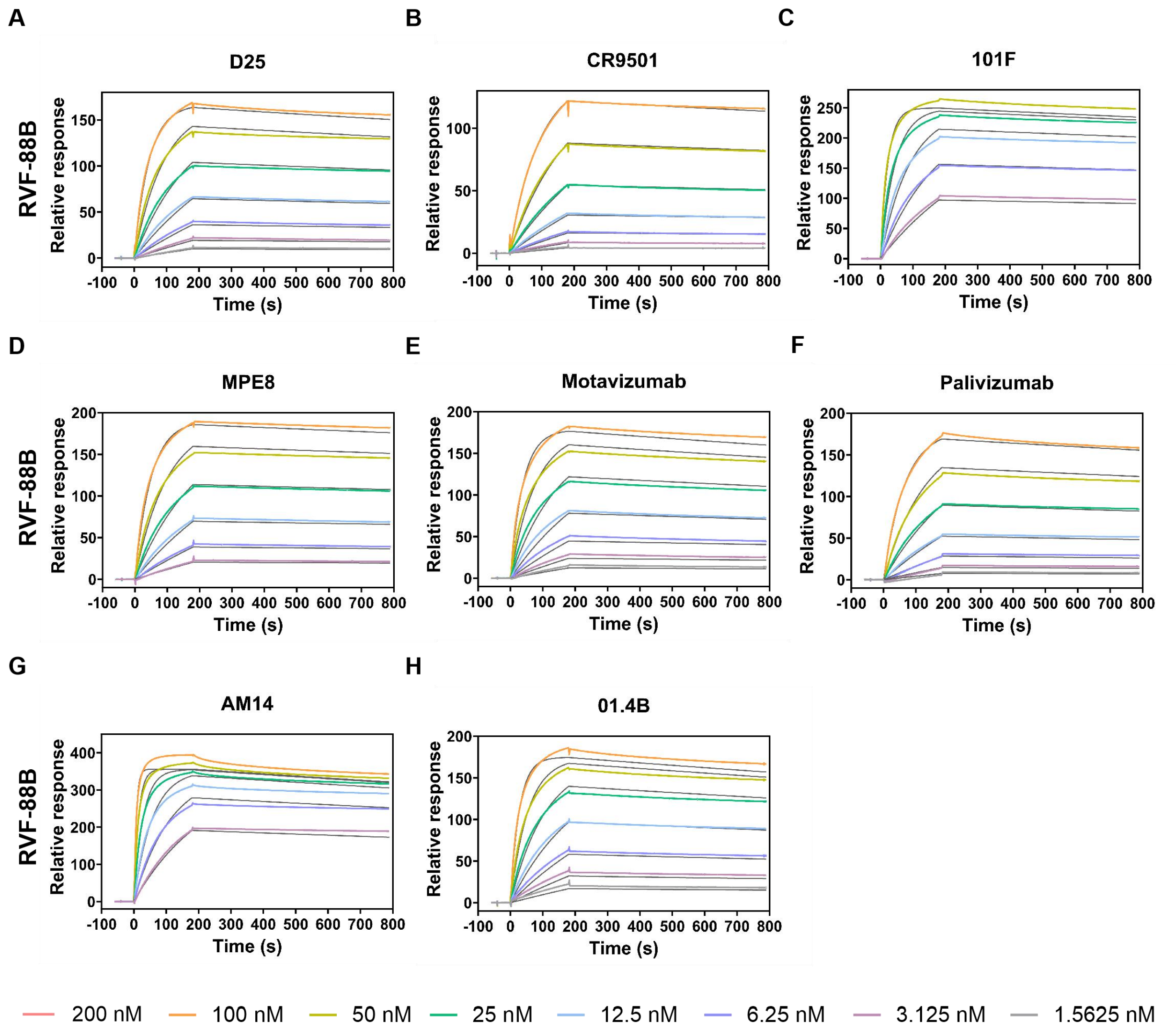

Appendix Figure S9. SPR binding profiles of RVF-88B to monoclonal antibodies.

SPR sensorgrams for RVF-88B binding to eight antibodies are shown: D25 (A), CR9501 (B), 101F (C), MPE8 (D), motavizumab (E), palivizumab (F), AM14 (G), and 01.4B (H). Binding was assessed at serial analyte concentrations from 200 nM to 1.5625 nM (colored traces), with black lines indicating global curve fits. Derived K_D values confirm that RVF-88B retains preF-specific antigenicity, with binding patterns comparable to RVF-88A and pXCS847A.

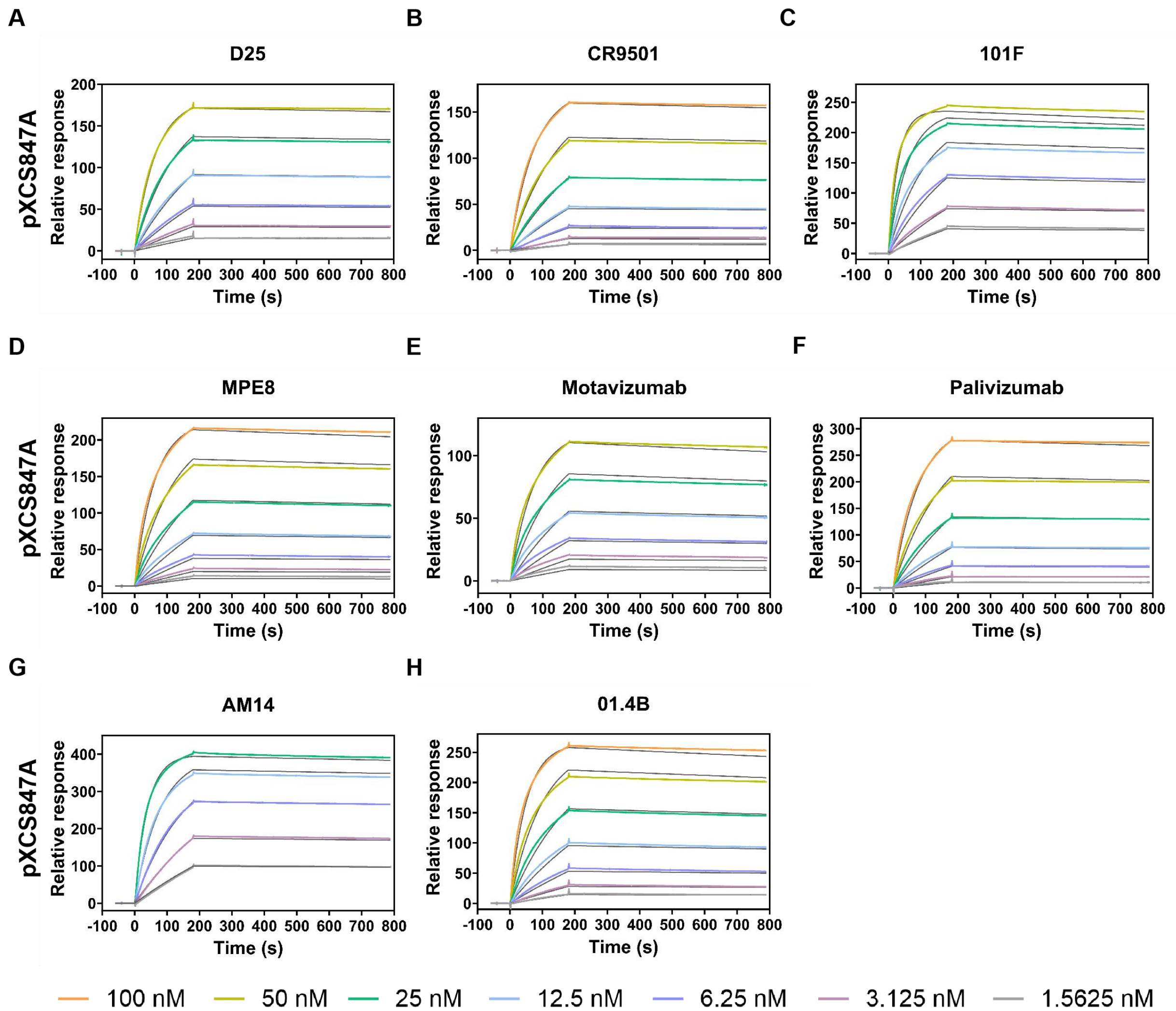

Appendix Figure S10. SPR binding profiles of pXCS847A to monoclonal antibodies.

SPR sensorgrams for pXCS847A binding to eight antibodies are shown: D25 (A), CR9501 (B), 101F (C), MPE8 (D), motavizumab (E), palivizumab (F), AM14 (G), and 01.4B (H). Serial analyte concentrations (100 nM to 1.5625 nM) are indicated by colored traces, with black curves representing fitted binding models. The binding kinetics provide a reference profile for comparison with RVF-88A and RVF-88B.

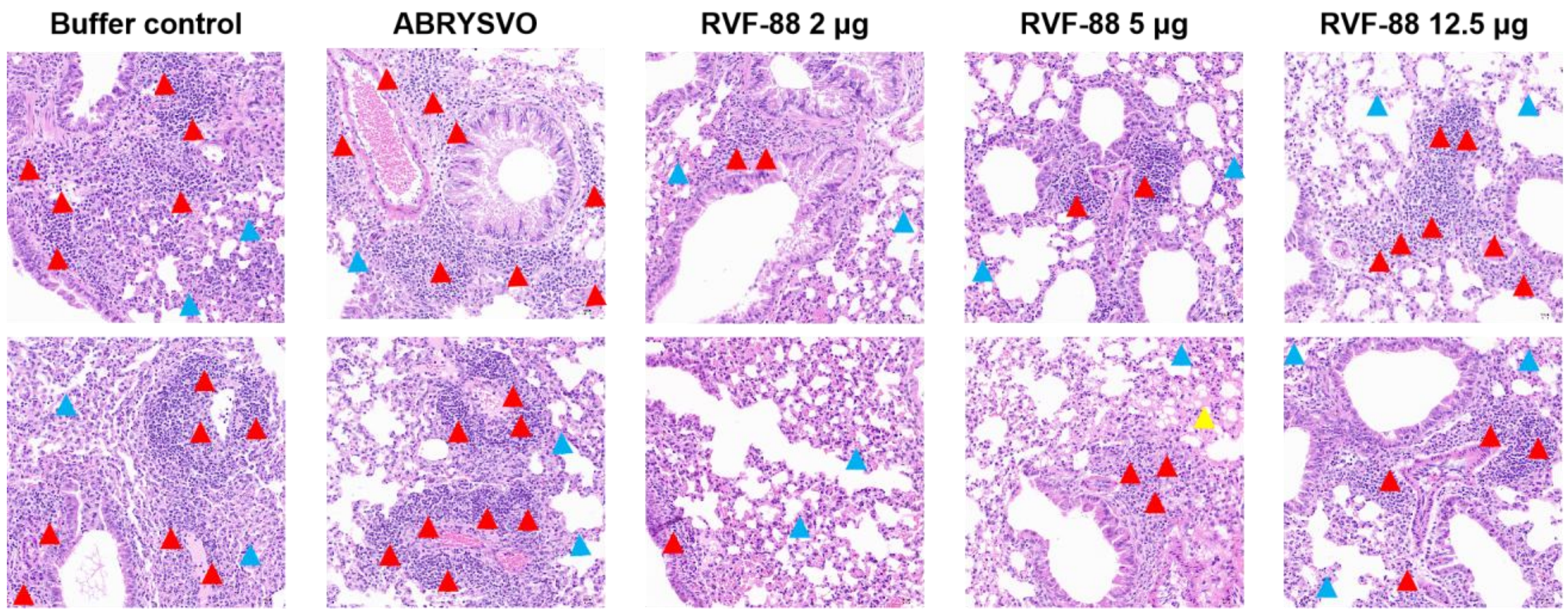

Appendix Figure S11. Histological analysis of lung tissue in BALB/c mice following immunization and viral challenge.

Lung tissue sections are stained with H&E, showing moderate structural abnormalities. Each group consists of two images, representing different areas of the lung. In the images, alveolar walls are thickened and alveolar epithelial cell proliferation is observed, as indicated by the blue arrows. A small amount of proteinaceous mucus is visible in some alveoli, indicated by the yellow arrows. The bronchial epithelial cells are arranged in a tight, orderly manner, with no evidence of desquamation. Additionally, slight infiltration of inflammatory cells is observed within the tissue, marked by red arrows. Groups shown include: Buffer control, ABRYSVO, and RVF-88 at 2 μg, 5 μg, and 12.5 μg doses.

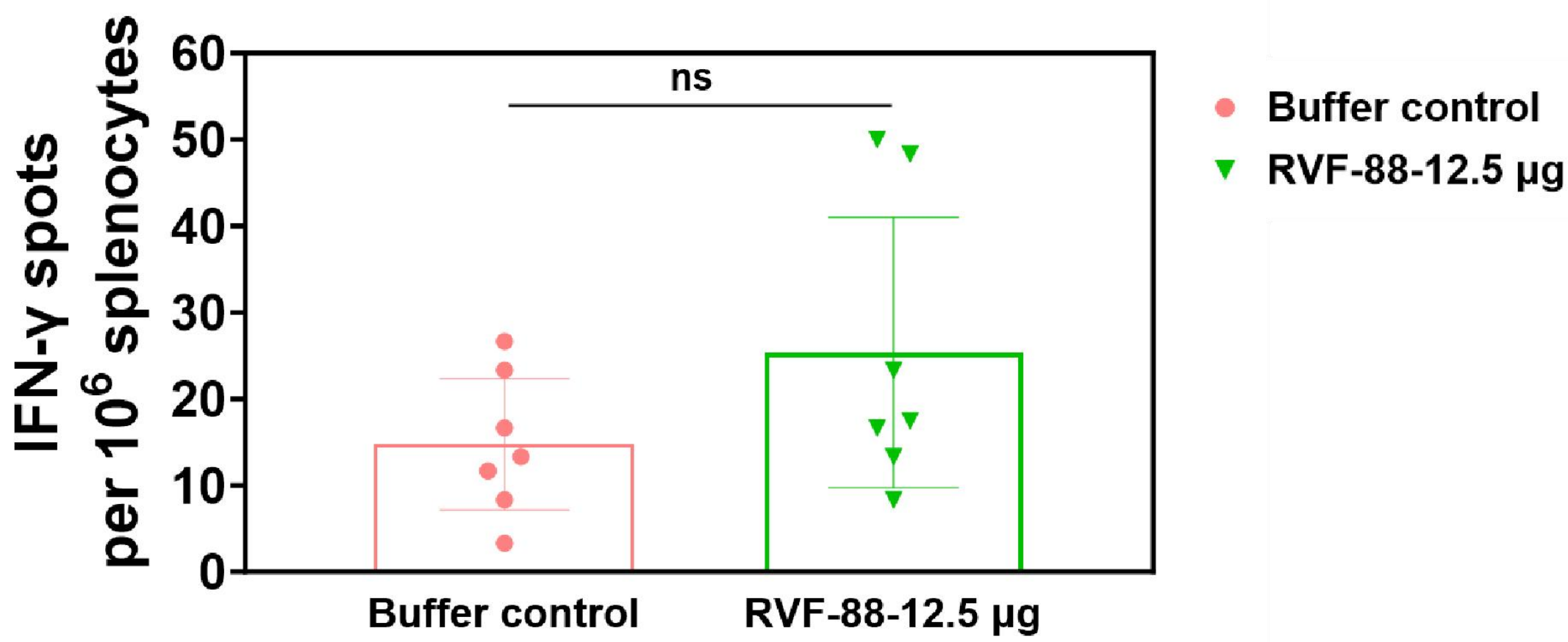

Appendix Figure S12. ELISpot analysis of IFN-γ responses in BALB/c mice following RVF-88 and control immunization.

Splenic spot-forming units (SFU) after stimulation with a peptide pool spanning RSV F. Buffer control (red circles) and RVF-88 (green triangles). Points show individual mice; bars indicate mean ± 95% confidence interval (CI); n=7 per group. Two-group comparisons were performed using two-sided Welch's t-test (no equal-variance assumption); ns denotes not significant.

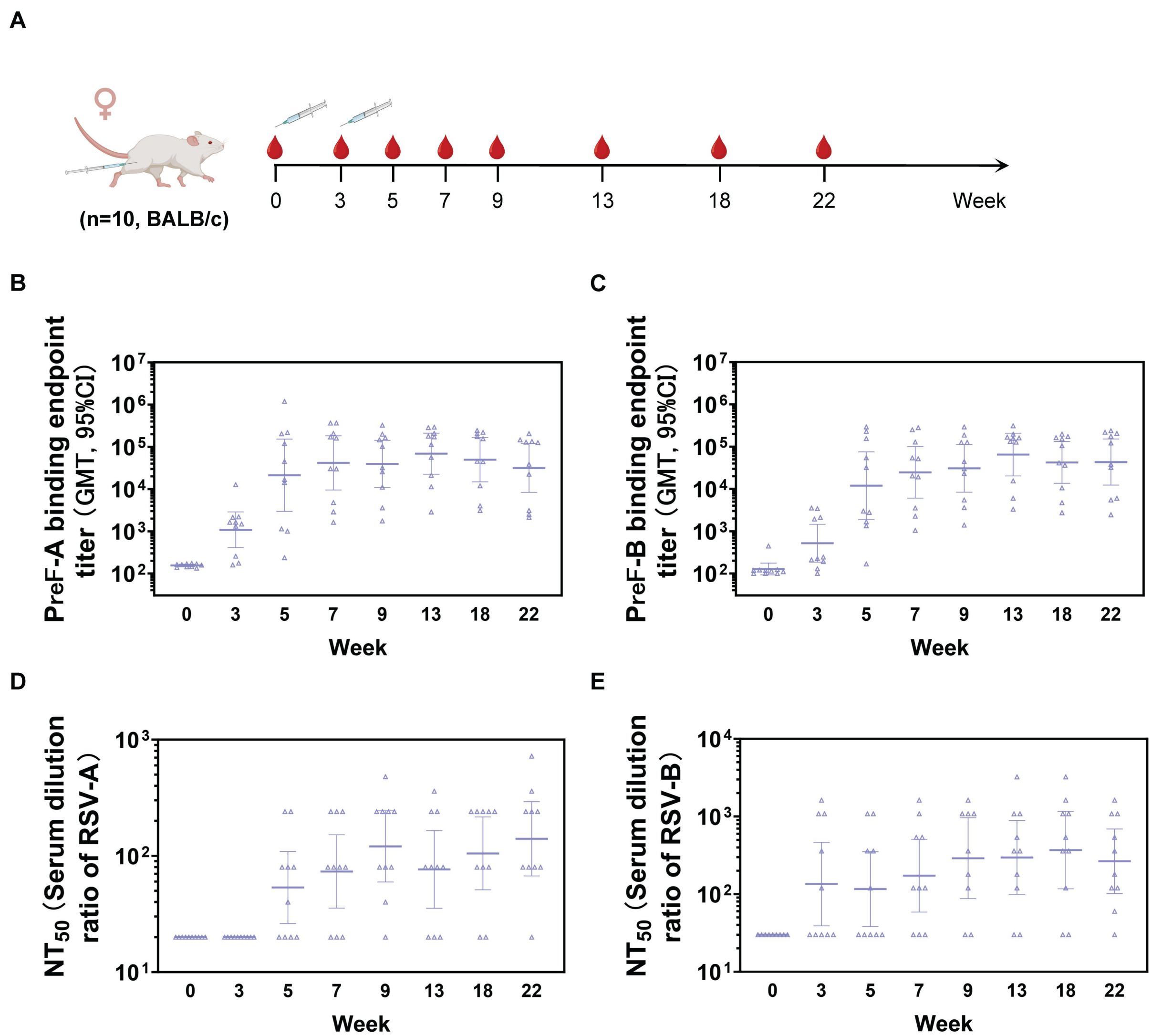

Appendix Figure S13. Evaluation of long-term antibody responses in BALB/c mice immunized with bivalent RVF-88.

(A) Female BALB/c mice (n = 10 per group) were immunized twice with 5 µg of bivalent RVF-88, with a 3-week interval between doses. Blood samples were collected at weeks 0, 3, 5, 7, 9, 13, 18, and 22 to monitor antibody responses over time. (B and C) PreF-A-specific (B) and PreF-B-specific (C) antibody titers in the serum of mice immunized with 5 µg of bivalent RVF-88. (D and E) Neutralizing antibody titers against RSV A2 (D) and RSV B9320 (E) in the serum of mice immunized with 5 µg of bivalent RVF-88. Results are expressed as geometric mean titers (GMTs) with 95% confidence intervals (CIs).

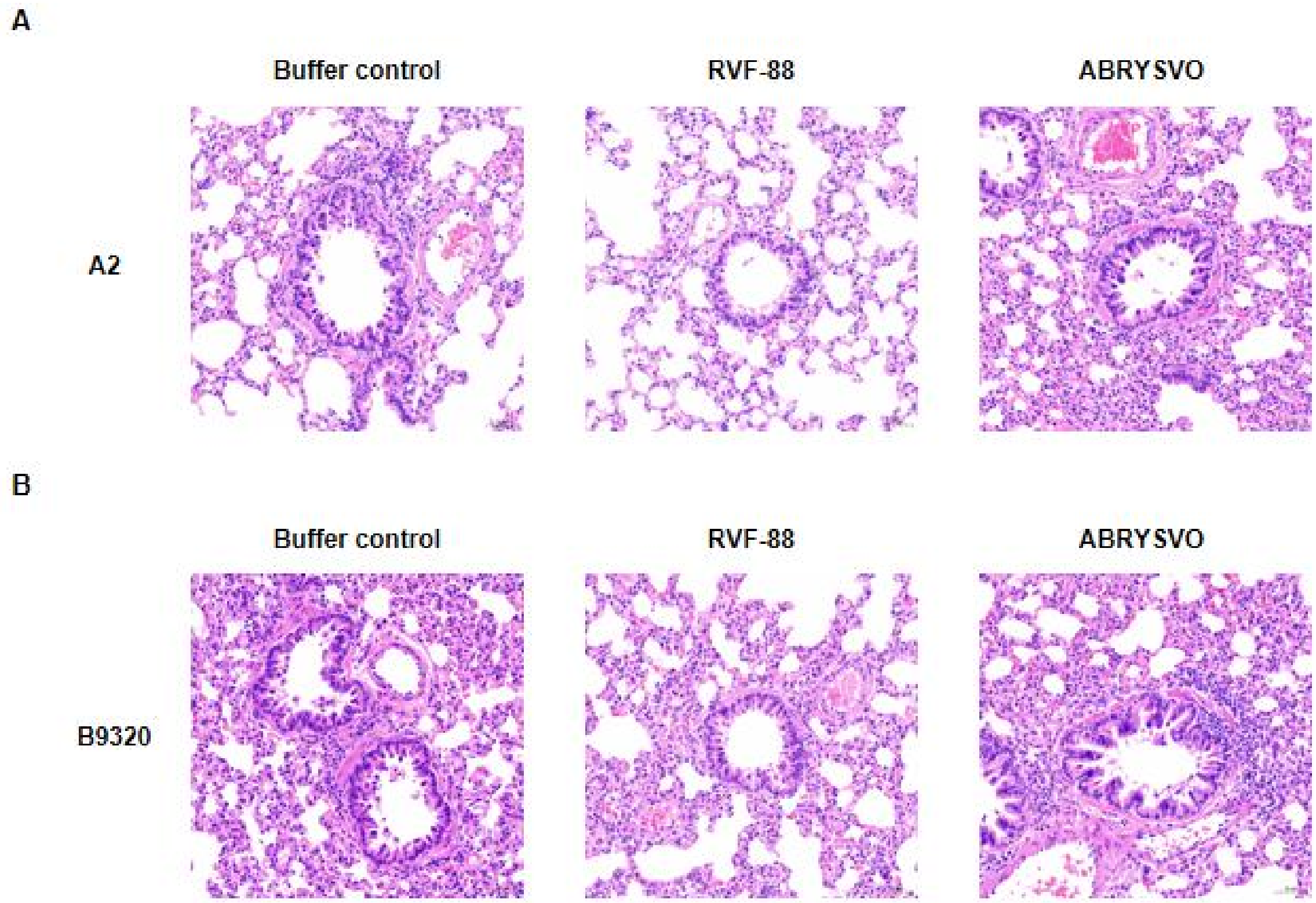

Appendix Figure S14. Histological analysis of lung tissue in cotton rats following immunization and viral challenge.

Representative hematoxylin and eosin (H&E) staining of lung sections from cotton rats immunized with buffer, ABRYSVO, or RVF-88, and challenged intranasally with RSV A2 (A) or RSV B9320 (B). Representative images are shown for qualitative assessment of lung histopathology, while quantitative histopathology scoring is shown in Fig. 5I and 5J.

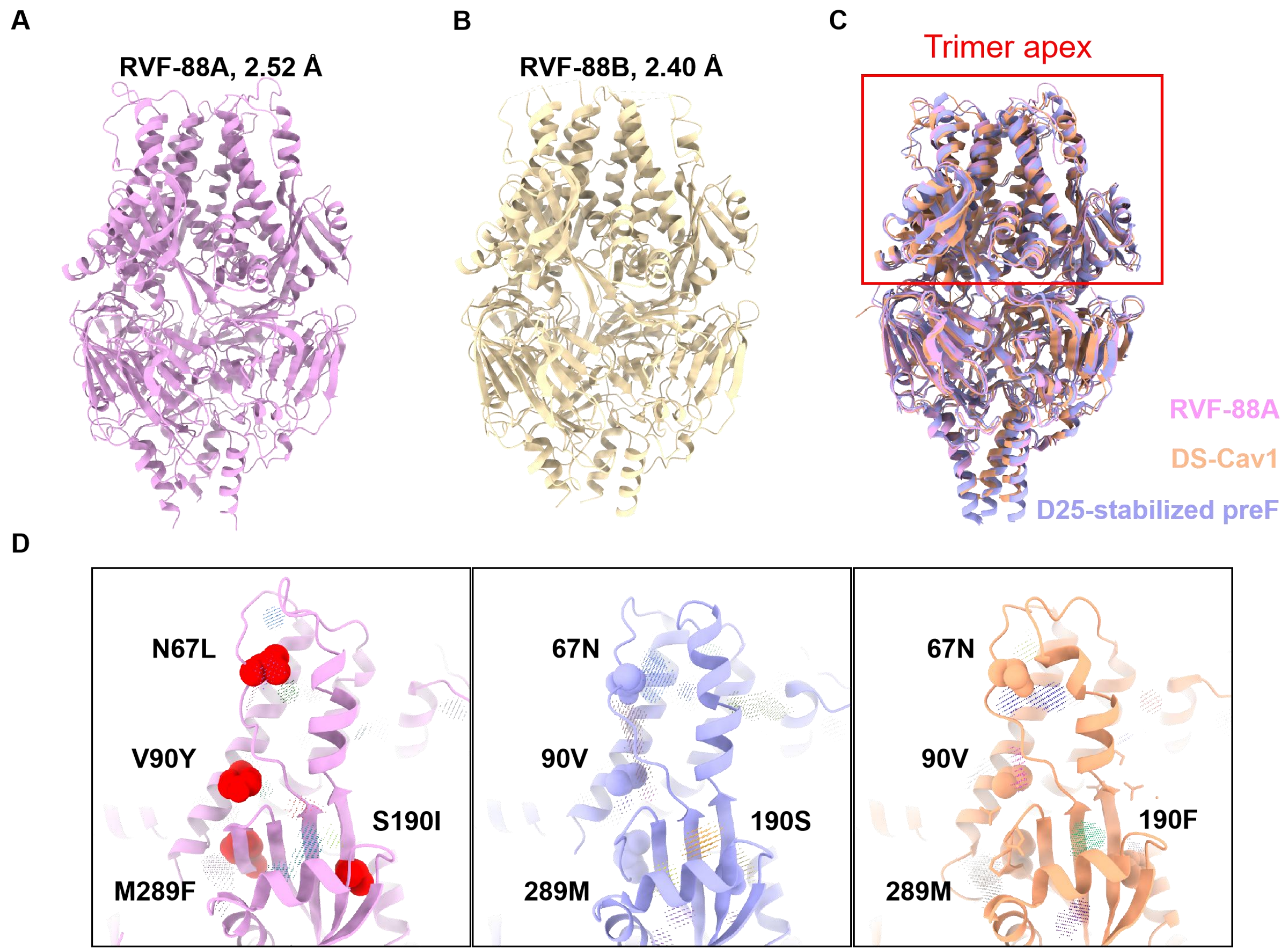

Site	RVF-88A			D25-stabilized preF PDB: 4JHW			DS-Cav1 PDB: 4MMU		
	No. of detected cavities	Total cavity volume (Å³)	Total cavity surface area (Å²)	No. of detected cavities	Total cavity volume (Å³)	Total cavity surface area (Å²)	No. of detected cavities	Total cavity volume (Å³)	Total cavity surface area (Å²)
67	4	58.53	107.36	1	76.68	95.79	2	110.38	156.04
90	1	12.31	29.54	3	112.1	148.35	1	20.95	35.76
190	0	/	/	1	45.79	54.78	1	7.78	13.38
289	2	167.62	194.95	1	107.78	135.21	2	223.99	238.11

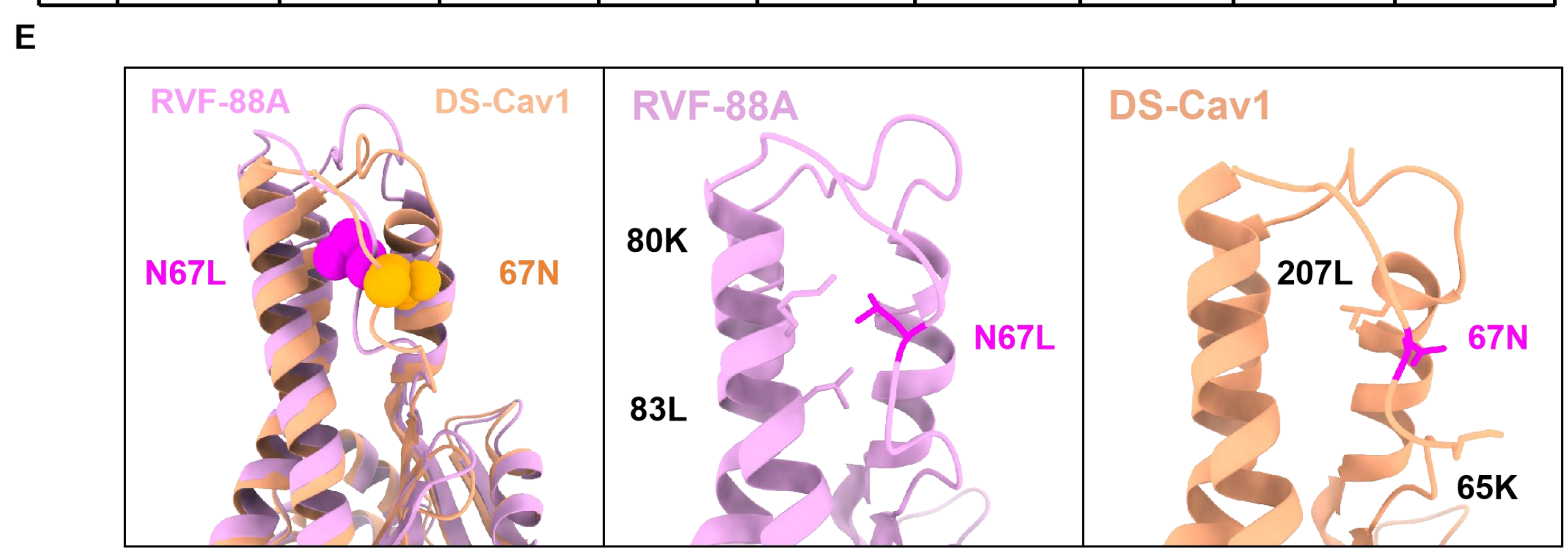

Appendix Figure S15. Crystal structures and cavity analysis of RVF-88.

(A and B) Crystal structures of RVF-88A (A) and RVF-88B (B) are shown as plum and wheat

cartoons, respectively. (C) Structural superposition of RVF-88A (plum), DS-Cav1 (orange, PDB: 4MMU), and D25-stabilized prefusion RSV F (light blue, PDB: 4JHW). The overall trimeric prefusion architectures are highly similar, with local structural differences observed near the trimer apex (highlighted in the red inset). (D) Local cavity analysis at the trimer apex of RVF-88A (left), D25-stabilized prefusion RSV F (middle), and DS-Cav1 (right). Surface cavities identified within the analyzed region are shown as colored dots. Mutation sites are displayed as spheres. The accompanying table summarizes the number of detected cavities together with the corresponding cavity volume and surface area measurements. (E) Local structural comparison at residue 67 between RVF-88A and DS-Cav1. Left, superposition of RVF-88A (plum) and DS-Cav1 (orange), with residue 67 displayed as spheres. Right, local views of residue 67 in RVF-88A and DS-Cav1 shown separately. Site residues are highlighted in magenta (stick) together with surrounding residues within 4 Å of the displayed atoms (stick).

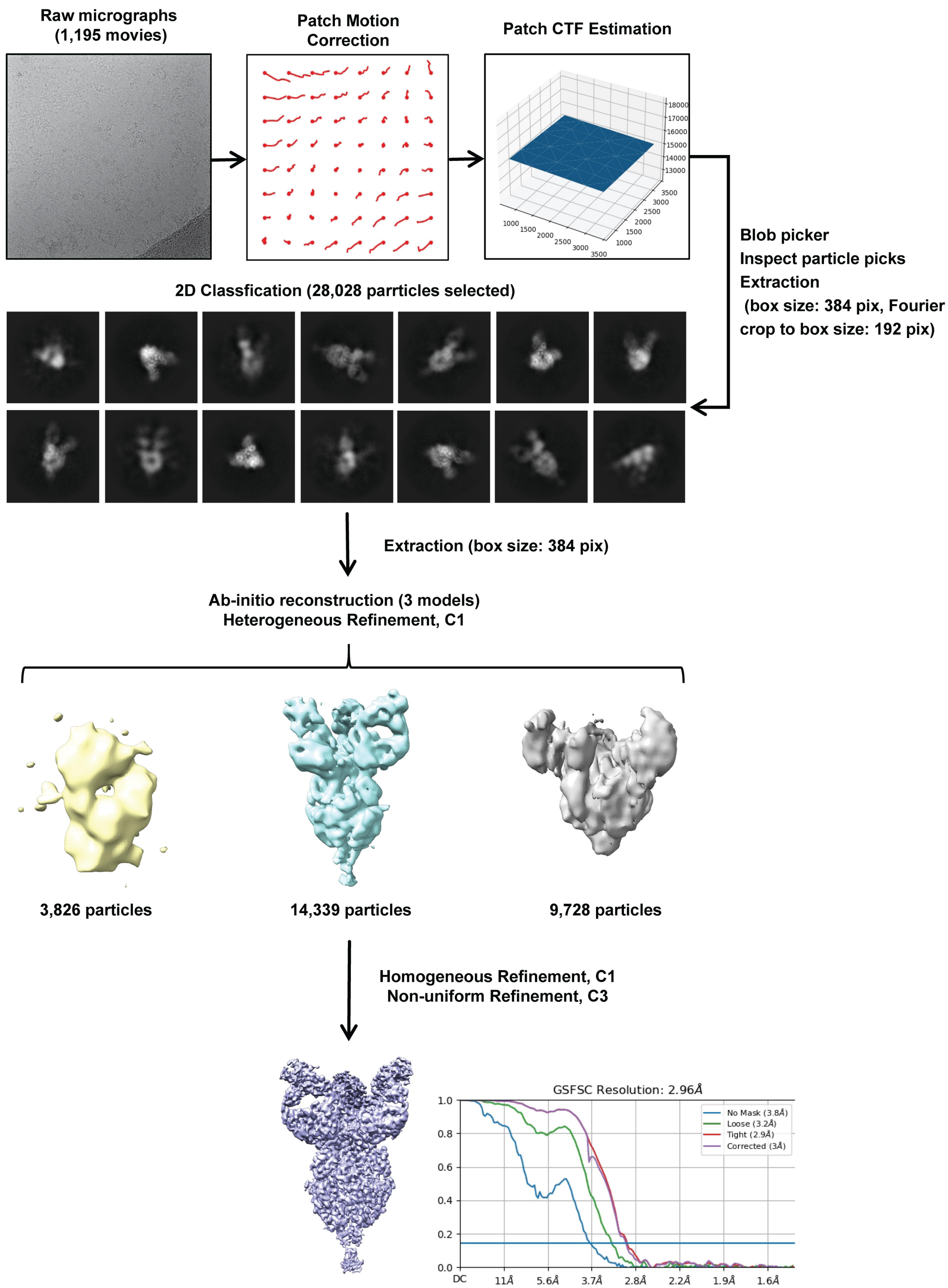

Appendix Figure S16. The single-particle cryo-EM workflow and analysis of RVF-88A in complex with D25 Fab.

Raw micrographs (1,195 movies) were processed by patch motion correction and patch CTF estimation. Particles were initially identified by blob picking and subjected to 2D classification (box size 384 px, Fourier-cropped to 192 px), yielding 28,028 high-quality particle images. These particles were re-extracted at the full box size (384 px) for ab initio three-class 3D reconstruction (C1 symmetry), resulting in classes containing 3,826, 14,339 and 9,728 particles. The dominant class underwent homogeneous refinement (C1), followed by non-uniform refinement with imposed C3 symmetry, producing a high-resolution Coulomb-potential map. The final gold-standard FSC indicated a resolution of 2.96 Å.

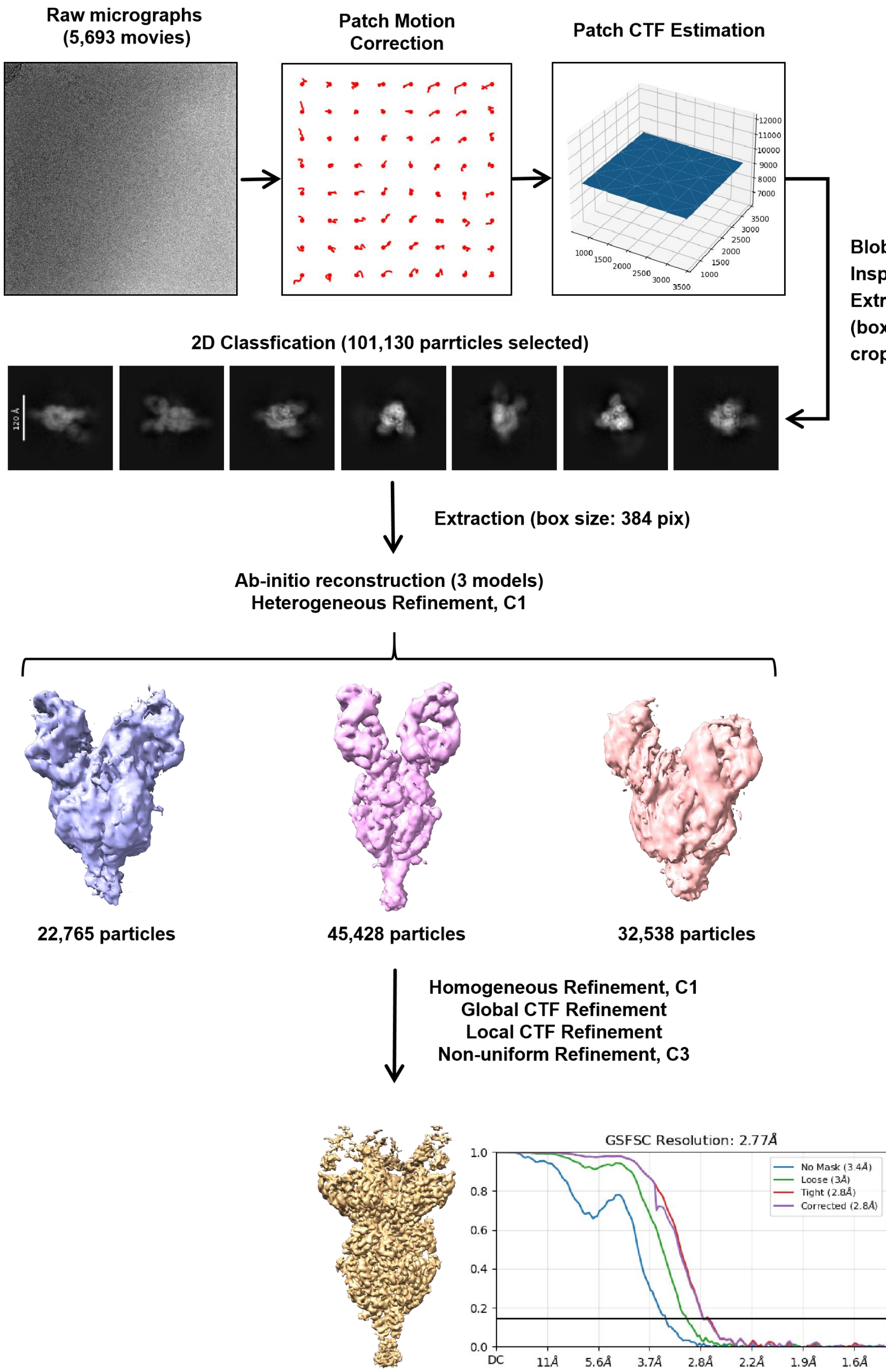

Appendix Figure S17. The single-particle cryo-EM workflow and analysis of RVF-88B in complex with D25 Fab.

Raw micrographs (5,693 movies) were processed by patch motion correction and patch CTF estimation. Particles were initially identified using blob picking, inspected, and extracted for 2D

classification (box size 384 px, Fourier-cropped to 192 px), yielding 101,130 selected particle images. These particles were then re-extracted at the full box size (384 px) and subjected to ab initio three-model reconstruction (C1 symmetry), followed by heterogeneous refinement, producing classes containing 22,765, 45,428 and 32,538 particles. The predominant class was refined using homogeneous refinement (C1), global CTF refinement, local CTF refinement, and non-uniform refinement with imposed C3 symmetry, resulting in a high-resolution Coulomb-potential map. The final gold-standard FSC curve indicated a resolution of 2.77 Å.

Appendix Table S1. SSM-based structural alignment statistics for seven segments of RSV F comparing the prefusion (PDB: 4JHW) and postfusion (PDB: 3RRR).

Segment	L (aa)	Nalign	Coverage	RMSD (Å)	Q score	Category
26–57	32	32	1.00	0.86	0.029	Similar
58–72	15	14	0.93	2.48	0.004	Different
73–96	24	20	0.83	0.97	0.011	Different
147–226	80	–	0	–	–	Different*
227–305	79	76	0.96	0.84	0.033	Similar
306–459	154	139	0.90	1.47	0.097	Similar
460–513	54	23	0.43	0.92	0.003	Different

Nalign, number of residues considered structurally equivalent and used in the least-squares fit; Coverage, Nalign/L where L is the total number of residues in the segment; RMSD, backbone root-mean-square deviation over aligned residues; Q, SSM structural similarity quality score. *SSM superposition was not achieved due to mismatched secondary structure.

Appendix Table S2. Region-based single-substitution screening of prioritized RSV F segments.

Region category	Residue range	Structural rationale	Candidate positions screened	Favorable substitutions identified by supernatant ELISA	Substitutions advanced for further combinatorial screening
Conformationally divergent region I	aa 58–72	Local pre/post divergence near a head-supporting element	N67	N67L/I/W/F/H/Y	N67L
Conformationally divergent region II	aa 73–96	Pre/post divergent head-proximal segment	I79, K80, L83, A89, V90, L93, L96	I79L/F/W; K80M/L/F/W; L83I; V90L/I/Y; L93F; L96M/I/Y	K80L, V90Y
Conformationally divergent region III	aa 137–226	Conformationally divergent region associated with prefusion head architecture	I148, S150, V152, S155, I167, V192, I214, I217, I221	I217L	I217L
Adjacent supporting region	aa 227–305	Structurally adjacent to the prefusion apex; may support head architecture	L230, M289, I292	L230F; M289L/F/Y; I292M/L/F/Y	L230F, M289F, I292L

Appendix Table S3. Binding kinetics of antibody interactions with RVF-88A, RVF-88B and pXCS847A as measured by surface plasmon resonance (SPR).

Kinetics model	Capture Solution	Analyte Solution	Kinetics Chi² (RU²)	k_a ($M^{-1}s^{-1}$)	k_d (s^{-1})	K_D (M)	Rmax (RU)	tc
1:1 binding	D25	RVF-88A	2.99E+01	3.00E+05	1.37E-04	4.57E-10	176.6	3.16E+13
1:1 binding	CR9501	RVF-88A	5.91E-01	8.06E+04	1.21E-04	1.50E-09	145	9.23E+11
1:1 binding	101F	RVF-88A	1.13E+02	8.18E+05	1.27E-04	1.56E-10	258.9	1.65E+14
1:1 binding	MPE8	RVF-88A	2.53E+01	2.37E+05	9.75E-05	4.11E-10	199.5	3.81E+12
1:1 binding	motavizumab	RVF-88A	1.35E+01	1.97E+05	1.69E-04	8.59E-10	178.3	2.06E+13
1:1 binding	palivizumab	RVF-88A	3.99E+00	1.02E+05	2.50E-04	2.45E-09	213.5	2.11E+09
1:1 binding	AM14	RVF-88A	2.11E+02	1.66E+06	1.48E-04	8.90E-11	382.4	3.48E+13
1:1 binding	01.4B	RVF-88A	6.27E+01	3.60E+05	1.42E-04	3.95E-10	195.9	5.78E+13
1:1 binding	D25	RVF-88B	9.89E+00	2.18E+05	1.37E-04	6.27E-10	167.9	9.70E+12
1:1 binding	CR9501	RVF-88B	7.65E-01	1.08E+05	1.14E-04	1.06E-09	143.4	2.13E+12
1:1 binding	101F	RVF-88B	1.20E+02	7.90E+05	1.21E-04	1.53E-10	263.7	5.63E+12
1:1 binding	MPE8	RVF-88B	1.64E+01	2.12E+05	8.62E-05	4.06E-10	189.3	1.13E+13
1:1 binding	motavizumab	RVF-88B	2.82E+01	2.57E+05	1.63E-04	6.33E-10	179.6	9.62E+13
1:1 binding	palivizumab	RVF-88B	1.13E+01	1.53E+05	1.36E-04	8.92E-10	182	6.64E+12
1:1 binding	AM14	RVF-88B	2.10E+02	1.40E+06	1.68E-04	1.20E-10	354.7	5.43E+13
1:1 binding	01.4B	RVF-88B	2.86E+01	3.83E+05	1.67E-04	4.37E-10	174.2	2.02E+13
1:1 binding	D25	pXCS847A	4.54E+00	3.09E+05	4.18E-05	1.35E-10	183.2	3.35E+12
1:1 binding	CR9501	pXCS847A	3.17E+00	1.33E+05	5.29E-05	3.99E-10	176.4	7.98E+13
1:1 binding	101F	pXCS847A	4.40E+01	6.74E+05	9.10E-05	1.35E-10	236.6	2.66E+13
1:1 binding	MPE8	pXCS847A	1.92E+01	1.63E+05	7.60E-05	4.67E-10	227.2	4.03E+12
1:1 binding	motavizumab	pXCS847A	1.97E+01	2.04E+05	9.66E-05	4.73E-10	141.1	3.16E+14
1:1 binding	palivizumab	pXCS847A	1.23E+01	1.26E+05	5.95E-05	4.74E-10	311.7	2.45E+11
1:1 binding	AM14	pXCS847A	3.23E+01	1.02E+06	4.50E-05	4.39E-11	398.8	2.36E+14
1:1 binding	01.4B	pXCS847A	3.02E+01	1.99E+05	9.72E-05	4.88E-10	266.6	1.71E+13

Appendix Table S4. Serum neutralizing antibody titers in mouse and cotton rat immunogenicity studies.

Animal model	Group	Dose/μg	Virus tested		Day 0	Day 21	Day 35
Mouse	Buffer	0	RSV A2	Geometric mean (95% CI)	20	20	20
			RSV B9320		30	30	30
				n	10	10	10
	RVF-88	2	RSV A2	Geometric mean (95% CI)	20	23（19-28）	218（106-448）
			RSV B9320		30	34（25-47）	235（91-604）
				n	10	10	10
		5	RSV A2	Geometric mean (95% CI)	20	28（19-43）	246（61-991）
			RSV B9320		30	32（27-38）	238（76-742）
				n	10	10	10
		12.5	RSV A2	Geometric mean (95% CI)	20	23（19-28）	334（145-767）
			RSV B9320		30	60（38-96）	375（159-881）
				n	10	10	10
	ABRYSVO	5	RSV A2	Geometric mean (95% CI)	20	20	51（26-109）
			RSV B9320		30	41（31-54）	74（30-178）
				n	10	9*	10
Cotton Rat	Buffer	0	RSV A2	Geometric mean (95% CI)	20	20	20
			RSV B9320		20	20	20
				n	10	10	10
	RVF-88	24	RSV A2	Geometric mean (95% CI)	20	49（31-79）	469（193-1142）
			RSV B9320		20	203（95-434）	3786（1010-14186）
				n	10	10	10
	ABRYSVO	24	RSV A2	Geometric mean (95% CI)	20	34（18-64）	184（54-625）
			RSV B9320		20	142（43-464）	1099（244-4953）
				n	10	10	10

*One sample was unavailable at the time of analysis.

Appendix Table S5. Summary of local cavity properties in the trimeric prefusion apex of RVF-88A and selected RSV F reference structures.

Structure	PDB	Resolution (Å)	Region analyzed (aa)	Condition	No. of detected cavities	Total cavity volume (Å³)	Total cavity surface area (Å²)	Total points	Largest cavity volume (Å³)
RVF-88A	25XQ	2.52	Trimeric apical/core box (residues 26–305)	Antibody-free crystal structure	49	2369.71	2884.28	10971	271.51
D25-stabilized preF	4JHW	3.60	Trimeric apical/core box (residues 26–305)	D25-stabilized preF reference; D25 removed before analysis	50	3789.65	4121.47	17545	899.42
DS-Cav1	4MMU	3.00	Trimeric apical/core box (residues 26–305)	Antibody-free crystal structure of preF variant	40	3907.66	3728.63	18091	1812.67

Appendix Table S6. Cryo-EM data collection, 3D reconstruction and refinement statistics.

	RVF-88A-D25 Fab	RVF-88B-D25 Fab
**Data collection and processing**		
Magnification	165,000	165,000
Voltage (kV)	300	300
Electron exposure ($e^-/Å^2$)	50	50
Exposure rate	10 $e^-/Å^2/s$	10 $e^-/Å^2/s$
Number of frames	40	40
Slit width of energy filter	10 eV	10 eV
Defocus range (μm)	-0.8 to -2.4	-0.8 to -2.4
Pixel size (Å)	0.729	0.729
Symmetry imposed	C3	C3
Micrographs used	1,195	5,693
Initial particle images (no.)	154,765	700,174
Final particle images (no.)	14,339	45,428
Map resolution (Å)		
FSC threshold	0.143/0.5	0.143/0.5
Masked	2.93/3.15	2.74/2.96
Unmasked	2.97/3.37	2.78/3.14
Map resolution range (Å)	2.93-3.15	2.74-2.96
**Refinement**		
Initial model used (PDB code)	7UJA	7UJA
Software	Phenix, Coot	Phenix, Coot
Model resolution (Å)	3.15	2.96
FSC threshold	0.5	0.5
Map sharpening B factor ($Å^2$)	68.7	80.9
Model composition		
Chains	9	9
Non-hydrogen atoms	15,822	15,840
Protein residues	2052	2049
Ligands	0	0
B factors ($Å^2$)		
Protein	83.48/247.36/134.98	84.35/251.44/143.11
Ligand	/	/
R.m.s. deviations		
Bond lengths (Å)	0.003	0.005
Bond angles (°)	0.644	0.696
Validation		
MolProbity score	1.98	2.18
Clashscore	7.16	7.75
Rotamer outliers (%)	1.92	2.74
Ramachandran plot		
Favored (%)	94.67	93.63
Allowed (%)	5.13	6.12
Outliers (%)	0.20	0.25
CaBLAM outliers (%)	3.69	3.30
CC (mask)	0.87	0.84
CC (volume)	0.87	0.85

Appendix Table S7. Crystallographic data collection and refinement statistics of RVF-88A and RVF-88B.

	RVF-88A	RVF-88B
Wavelength (Å)	0.97946	0.96183
Cell parameters	105.39; 159.19; 116.57	97.66; 178.77; 116.55
(a, b, c, α, β, γ)	90; 102.86; 90	90; 97.90; 90
Space group	$P2_1$	$P2_1$
Completeness (%)	96.9(97.0)	99.5(98.8)
I/σ	4.66(2.56)	6.35(0.56)
Resolution (Å)	50.0-2.52(2.67-2.52)	50.0-2.40(2.54-2.40)
No. of unique reflections	127749	154097
Rwork	0.2093	0.2249
Rfree	0.2669	0.2633
Bond length (Å)	0.009	0.004
Bond angle (°)	0.982	0.624
Ramachandran outliers (%)	0.11	0.12
