# Supplementary material for: A cavity-reduced prefusion RSV F bivalent vaccine elicits durable and protective immunity
Source: EMBO Mol Med. 2026 Aug 29;18(10):3979–4000. doi: 10.1038/s44321-026-00511-8 (PMC13658822; doi:10.1038/s44321-026-00511-8)
Supplement: Supplementary file 2 [file 44321_2026_511_MOESM2_ESM.pdf]

EMBO Molecular Medicine

A cavity-reduced prefusion RSV F bivalent vaccine elicits durable and protective immunity

Lijie Liu, Mengrong Yan, Ruoxu Liang, Qingxin Wu, Changbin Qu, Huiqi Wu, Zhi Liao, Huiyu Chen, Jingjing Zou, Zeyun Sun, Dongyu Niu, Liyuan Wu, Li Zhao, Jialing Dai, Jialun Shen, Long Zhang, Wei Zhang, and Wei Peng

Corresponding author(s): Lijie Liu (liu_lijie@gzlab.ac.cn) , Long Zhang (zhang_long@gzlab.ac.cn), Wei Zhang (zhang_wei2@gzlab.ac.cn), Wei Peng (peng_wei@gzlab.ac.cn)

Review Timeline:

Submission Date:	10th Feb 26
Editorial Decision:	20th Mar 26
Revision Received:	30th Jun 26
Editorial Decision:	20th Jul 26
Revision Received:	7th Aug 26
Accepted:	11th Aug 26

Editor: Zeljko Durdevic

Transaction Report:

(Note: With the exception of the correction of typographical or spelling errors that could be a source of ambiguity, letters and reports are not edited. Depending on transfer agreements, referee reports obtained elsewhere may or may not be included in this compilation. Referee reports are anonymous unless the Referee chooses to sign their reports.)

20th Mar 2026

Dear Dr. Peng,

Thank you for the submission of your manuscript to EMBO Molecular Medicine. We have now received feedback from the three reviewers who agreed to evaluate your manuscript. All three referees recognize interest of the study but also raise important concerns that should be addressed in a major revision. If you would like to discuss further the points raised by the referees, I am available to do so via email or video. Let me know if you are interested in this option.

Further consideration of a revision that addresses reviewers' concerns in full will entail a second round of review. EMBO Molecular Medicine encourages a single round of revision only and therefore, acceptance or rejection of the manuscript will depend on the completeness of your responses included in the next, final version of the manuscript. For this reason, and to save you from any frustrations in the end, I would strongly advise against returning an incomplete revision.

We would welcome the submission of a revised version within three months for further consideration. Please let us know if you require longer to complete the revision.

I look forward to receiving your revised manuscript.

Yours sincerely,

Zeljko Durdevic

Zeljko Durdevic
Senior Editor
EMBO Molecular Medicine

*****
When preparing your revised manuscript, please refer to our guidelines: https://link.springer.com/journal/44321/submission-guidelines#cms-Revised-submissions. We perform an initial quality control of all revised manuscripts before re-review; failure to include requested items will delay the evaluation of your revision.

We require:

1) A .docx formatted version of the manuscript text (including legends for main figures, EV figures and tables). Please make sure that the changes are highlighted to be clearly visible.

2) Individual production quality figure files as .eps, .tif, .jpg (one file per figure). For guidance, download the 'Figure Guide PDF': https://media.springernature.com/original/springer-cms/rest/v1/content/27825798/data/v1.

3) A .docx formatted letter INCLUDING the reviewers' reports and your detailed point-by-point responses to their comments. As part of the EMBO Press transparent editorial process, the point-by-point response is part of the Review Process File (RPF), which will be published alongside your paper.

4) A complete author checklist, which you can download from our author guidelines. Please insert information in the checklist that is also reflected in the manuscript. The completed author checklist will also be part of the RPF.

5) Please note that all corresponding authors are required to supply an ORCID ID for their name upon submission of a revised manuscript.

6) It is mandatory to include a 'Data Availability' section after the Materials and Methods. Before submitting your revision, primary datasets produced in this study need to be deposited in an appropriate public database, and the accession numbers and database listed under 'Data Availability'. Please remember to provide a reviewer password if the datasets are not yet public.

In case you have no data that requires deposition in a public database, please state so in this section. Note that the Data Availability Section is restricted to new primary data that are part of this study.

7) For data quantification: please specify the name of the statistical test used to generate error bars and P values, the number (n) of independent experiments (specify technical or biological replicates) underlying each data point and the test used to calculate p-values in each figure legend. The figure legends should contain a basic description of n, P and the test applied. Graphs must include a description of the bars and the error bars (s.d., s.e.m.).

8) At EMBO Press we ask authors to provide source data for the main manuscript figures. You will receive a separate email with instructions for providing source data with your revised manuscript, including how to upload and organize the files.

9) Our journal encourages inclusion of *data citations in the reference list* to directly cite datasets that were re-used and obtained from public databases. Data citations in the article text are distinct from normal bibliographical citations and should directly link to the database records from which the data can be accessed. In the main text, data citations are formatted as follows: "Data ref: Smith et al, 2001" or "Data ref: NCBI Sequence Read Archive PRJNA342805, 2017". In the Reference list, data citations must be labeled with "[DATASET]". A data reference must provide the database name, accession number/identifiers and a resolvable link to the landing page from which the data can be accessed at the end of the reference.

10) We replaced Supplementary Information with Expanded View (EV) Figures and Tables that are collapsible/expandable online. A maximum of 5 EV Figures can be typeset. EV Figures should be cited as 'Figure EV1, Figure EV2" etc... in the text and their respective legends should be included in the main text after the legends of regular figures.

- For the figures that you do NOT wish to display as Expanded View figures, they should be bundled together with their legends in a single PDF file called *Appendix*, which should start with a short Table of Content. Appendix figures should be referred to in the main text as: "Appendix Figure S1, Appendix Figure S2" etc.

- Additional Tables/Datasets should be labeled and referred to as Table EV1, Dataset EV1, etc. Legends have to be provided in a separate tab in case of .xls files. Alternatively, the legend can be supplied as a separate text file (README) and zipped together with the Table/Dataset file.

11) The paper explained: EMBO Molecular Medicine articles are accompanied by a summary of the articles to emphasize the major findings in the paper and their medical implications for the non-specialist reader. Please provide a draft summary of your article highlighting

- the medical issue you are addressing,

- the results obtained and

- their clinical impact.

This may be edited to ensure that readers understand the significance and context of the research. Please refer to any of our published articles for an example.

12) Author contributions: You will be asked to provide CRediT (Contributor Role Taxonomy) terms in the submission system. These replace a narrative author contribution section in the manuscript.

13) A Conflict of Interest statement should be provided in the main text.

14) Every published paper includes a 'Synopsis' to further enhance discoverability. Synopses are displayed on the journal webpage and are freely accessible to all readers. They include a short stand first (maximum of 300 characters, including space) as well as 2-5 one-sentences bullet points that summarizes the paper. Please write the bullet points to summarize the key NEW findings. They should be designed to be complementary to the abstract - i.e. not repeat the same text. We encourage inclusion of key acronyms and quantitative information (maximum of 30 words / bullet point). Please use the passive voice. Please attach these in a separate file or send them by email, we will incorporate them accordingly.

Please also suggest a striking image or visual abstract to illustrate your article as a PNG file 550 px wide x 300-600 px high.

15) Include a Reagents and Tools Table as part of the Methods section, which can be downloaded from our author guidelines.

EMBO Molecular Medicine has a "scooping protection" policy, whereby similar findings that are published by others during review or revision are not a criterion for rejection. Should you decide to submit a revised version, I do ask that you get in touch after three months if you have not completed it, to update us on the status.

***** Reviewer's comments *****

Referee #1 (Remarks for Author):

Review 'Structure-guided rational design of a compact prefusion RSV F bivalent vaccine with enhanced immunogenicity'. This article describes the design and testing of a potential new RSV prefusion F vaccine. The study is well carried out and described in a detailed way. In general I feel the writers could have done more comparison with the DS-Cav1 molecule, which was the first prefusion stabilized version of RSV F, as they also refer to this molecule in the introduction and the discussion.
Below you can find my comments (mostly minor comments).

Line 177: What do you mean with 7 days (day10)?
Line 189, what is the rationale for looking at 7days? In Flynn et al, they wait 14d, Zhang et al 1 month. How would DS-Cav1 score in this analysis?
Fig2a and b, what do the other colors mean? Please signify or change to black. It seems that some of these should be red?
Fig 3a. Line 286; should be upper and lower instead of left and right; it is not clear what F1 and F3 means.
Fig 3c: square in stead of site zero symbol; no site mentioned for AM14
It would be interesting to also confirm lack of postfusion f with a site I specific antibody.
Fig 4 b and c, what antigens are used for coating? RVF L88A and B? Maybe also check binding to another prefusion F like DS-Cav1?
Line 312: the dose dependent effect is only minimal, probably no significant difference?
Fig 4d, some mice do not show neutralizing titers at d35, with 5 µg vaccine, do you have an explanation for this?
What was the reason not to include an adjuvant? Overall neutralization titers seem quite low
Figure 4G is redundant, showing 4H is enough.
Line 326, maintained at lower level? Not at the detection limit for 5 and 12.5 µg of RVF-L88? Could you indicate the detection limit on the graph? (4J) (also for the viral titers in the cotton rat experiment)
PDF issue with Fig s12, some letters in the Y-axis appear very bold
5C: what does the dotted line mean
Line 388: Weight loss is still quite minimal, as is expected with cotton rats
Line 401: yet in 5K, there is no difference in the inflammation scores between the different conditions. This is not consistent with the text.
Why don't you show the increased packing with the cavity-filling substitution at site 190 in Fig. 6c?
Line 510: How is manufacturing complexity improved, you did not show that this construct leads to greater yields compared to other constructs.
Line 511: yet you never compare to DS-Cav1
Line 654: you probably mean Targetmol

Referee #2 (Comments on Novelty/Model System for Author):

Overall, the choice for current cotton rat model is well accepted in studying RSV related infections, except that body weight change is not significance in cotton rat and I would suggest the author to remove the body weight change graph from their manuscript.

Referee #2 (Remarks for Author):

This manuscript describes a method for screening and rational design of RSV pre-F proteins based on known structures, aiming to develop an immunogen with fewer mutations compared to DS-Cav2 or ABRYSVO while maintaining the necessary pre-F-specific immunogenicity. The authors named this immunogen L88 (composed of two antigens, L88A and L88B) and systematically conducted preclinical studies on the adjuvant-free vaccine formulated with this combination. The full text is generally divided into two parts: the screening process of candidate immunogens mainly based on tool antibody ELISA, and the in vivo and in vitro evaluation of the final construct selected by the authors. Overall, the idea of this manuscript is easy to read, and the data are relatively abundant.
However, its most critical shortcoming is that neither the screening method, the selection strategy of candidate mutation sites, nor the evaluation method and results of the immunogen can well reflect the novelty of this manuscript. In addition, I have concerns about the effectiveness and necessity of the screening strategy adopted. The authors need to clearly indicate why the finally selected five-mutation construct is rationally optimal, and discuss the logical relationship between the selection of this

construct and the screening strategy as well as the inevitability of obtaining this construct. The authors pointed out in the discussion that they avoided the use of disulfide bonds in their design to optimize antigen properties, but I did not see the rationality of this consideration from the results they presented. I also have some questions about the logic of the authors' immunogen screening. In addition, there are several problems in the writing of this manuscript, such as typos, inconsistent colors, and unclear experimental methods. It is hoped that the authors will pay attention to the rigor of the writing.

I will elaborate on my questions in detail below:

//Major Concerns//

1. Regarding the rationality and originality of mutation site selection. The authors spent a large part of the first section of the results describing the conformational differences between pre- and post-fusion. Since this is already a widely accepted fact, it is recommended to drastically abbreviate this part. The four-point modification strategy selected by the authors (Lines 126-128) has been systematically reported in previous studies, all based on static structural analysis of pre- and post-fusion. If the authors cannot emphasize the fundamental difference between their screening logic and previously reported ones on this basis, this part should be elaborated in the introduction rather than in the results. The originality here refers not only to the addition or reduction of specific individual sites, but also to the logical basis at the molecular mechanism level of their strategy. It is recommended that the authors re-explore and make a parallel comparison between their selected optimization strategy and all existing reported design methods for stabilizing pre-F to highlight the novelty of their design strategy. This is the key innovation of this manuscript, and it is recommended that the authors rewrite this part to highlight what is novel in their design methodology.

2. The term "High-throughput screening" is inaccurate. From the authors' screening strategy, if my current understanding is correct, it mainly involves preliminary characterization of the expression level and conformation of more than 100 single-point mutations based on ELISA, and screening of combinations of individual prominent single-point mutations. Overall, this strategy is only labor-intensive but not high-throughput, and its bottleneck is the efficiency of ELISA detection. This strategy can neither traverse all combinatorial mutations nor reasonably exclude a large number of invalid mutations in the huge combinatorial mutation space based on ideas such as selecting Pareto optimality. It is recommended that the authors no longer emphasize high-throughput.

3. Regarding the process by which the authors obtained the final four-mutation (N67L-V90Y-S190I-M289F) or five-mutation (N67L-V90Y-S190I-M289F-D486S) combination, there is still a high degree of randomness from the results currently presented. The authors' arguments on the sufficiency and necessity of these mutations being included in the final construct are insufficient; the discussion on the inevitability of obtaining this combinatorial mutation through the screening strategy they adopted is also insufficient. Among these, S190I and D486S have been reported in previous studies. In Fig. 2A of this manuscript and related supplementary figures, the contribution of D486S to the proportion of pre-fusion conformation is not prominent, while S190I is not in the authors' initial mutation set at all. The appearance of these two mutations in the final construct lacks sufficient rationality. In Figure 2C, the data presented by the authors show that the four-mutation construct has a great advantage over the three-mutation construct N67L-V90Y-M289F, but no results of the S190I single mutation are given. Does this indicate that the S190I mutation has the greatest effect on the stability of the pre-fusion conformation among these four single-point mutations? I suggest the authors to re-organize this part in order to highlight the necessity of their selected mutation.

4. The data in Fig. 3B show that L88B has much weaker binding activity to D25 than L88A (by ELISA), but there is no significant difference in the KD values based on SPR. Similarly, L88A has lower affinity for palivizumab, but shows similar affinity to its humanized version motavizumab as L88B. It is generally believed that D25 is not an RSV A-specific antibody. The authors are suggested to explain this inconsistency from the perspective of experimental design and rule out that it is caused by experimental errors.

5 Fig. 5B shows that the error bars of the L88 and ABRYSVO groups on Day 35 are large. Can the authors display each individual data point on the basis of the current graph?

6. For Figure 5G-J, a sham group must be included for the RNA copies assay. If such data are not available, it is recommended to remove all the RNA copy number determination results in this figure. Since cotton rats infected with RSV do not show significant weight loss, it is also recommended to remove Figs. 5E-F and the content related to weight changes in the main text. When comparing whether there are significant differences in other parts of Fig. 5 in the main text, the authors should specify whether it is RNA copies or FFU, because in many groups, the results of these two detection methods are different.

7. Regarding the structural characterization part, I suggest that on the basis of the existing structures, the authors could provide separate crystal or cryo-EM structures of the pre-fusion trimeric forms of L88A/B without any antibodies, even. Even a low resolution structure will help. It is also preferable to provide the determination of the proportion of pre- and post-fusion conformations of the construct sample in solution if available(such as negative staining EM). The reasons are as follows: First, D25 helps stabilize the pre-fusion conformation. Even the WT RSV-F protein can obtain stable pre-fusion conformation crystals when co-crystallized with D25 (PMID: 23618766), which makes the EM structure of the antibody-containing complex unable to be used to judge whether L88A/B spontaneously forms the pre-fusion conformation implied by the authors. I understand that the

authors performed circular dichroism detection in Fig. S5 and implied that its helix ratio is highly consistent with the pre-fusion conformation, but the antibody complex structure does not provide additional information on this basis. On the other hand, D25 and other site-O antibodies help stabilize the local conformation of the antigen, and in fact, RSV-F has an alternative conformation near site O (PMID: 31306469). Since the authors attempt to discuss the differences in specific side chain positions at near-atomic resolution based on the structure, the authors need to ensure that this change is not an artifact caused by antibody binding. Even if the resolution of L88A/B without antibody binding is not sufficient for accurate side chain modeling, this structure should be provided for comparison. If there is no apo structure of the trimer, it is recommended to rewrite the structural analysis of the D25 complexed structure and point out in main text that there is still possibility the pre-fusion structure is maintained in part by the D25 antibody. It is also suggested to pay special caution on the side-chain analysis and the analysis to claim that the L88 is a more "compact" construct solely on the current available structure.

8. The figure legend of Fig. 6 shows five panels (A-E), but there are only four panels (A-D) in the actual figure. Please the authors explain the correspondence.

//Minor Suggestions//

1. Line 64. The term "inherently unstable" cannot be used to describe the pre-fusion conformation; the correct term for this conformation is "metastable", which indicates that it is still stable. "Inherently unstable" tends to be used to describe a thermodynamically transient unstable state.

2. Line 67. The main reason for selecting pre-fusion is not to avoid ERD, but to display pre-fusion-specific epitopes, and these antibody epitopes happen to have high neutralizing activity. The causes of ERD are relatively complex, and non-neutralizing antibodies are only one of the speculated possible related factors, and engineering pre-fusion-specific immunogens is not to eliminate ERD. It is recommended to delete the expressions related to ERD.

3. Line 72. Although RSV has two genotypes (A/B), they belong to the same serotype. Current studies have fully proved that an immunogen of one genotype can produce cross-neutralization and protection against genotypes A/B under the action of strong adjuvants. Therefore, bivalent design is not necessary in RSV vaccine design. It is recommended to revise this part.

4. Lines 84-85. The term "multi-proline" is not mentioned in the literature cited by the authors. The multi-proline design scheme is more famous in the design of HIV and SARS-CoV-2 antigens (S2P or S6P). Please revise the corresponding expressions or add the corresponding reference literature.

5. Line 91. The authors intend to demonstrate how to achieve pre-fusion conformation design with the minimal mutations in this manuscript, but the five-mutation design mentioned in this manuscript is not the one with the least number of mutations to achieve this goal; see, for example: PMID: 39325881, 37023209, etc. In addition, the authors have not proved that these five mutations are the minimal set of essential mutated amino acids, that is, each single mutation is indispensable. Therefore, it is recommended that the authors remove this expression.

6. Lines 128-129: The description "targeted residues within regions that undergo substantial conformational rearrangement (residues 58-226 and 460-513)" is not rigorous. For example, sites such as M289 and I292 are not within this range, although they play a role in stabilizing the prefusion conformation by stabilizing the pre-F protein in the above range. It is recommended that the authors make a more rigorous description.

7. Lines 134-139: Only 16 of the 23 proline-scanning mutations described by the authors are given in Figure S1. Please supplement the remaining seven sites. Whether there are combinatorial mutations (such as the T267P-N268P combinatorial mutation mentioned in Line 194) should also be correctly described. Were the 20 sites of cavity-filling mutations each mutated to seven amino acids (Met, Leu, Ile, Phe, Tyr, His, Trp) or only to a subset of them? Please make a clearer description in the supplementary materials.

8. Excessive colors are used in Fig. 1A and are difficult to distinguish. It is recommended to use the same color for the post-fusion structures of all motifs to display them superimposed with the pre-fusion structures to produce sufficient distinguishability. In addition, the authors should clearly indicate what the criteria for distinguishing "similar" and "different" are? Why are RMSD=0.97 and 0.92 defined as "different", but RMSD=1.47 is instead defined as "similar"? In addition, the RMSD value is missing for the aa. 137-226 part.

9. For Fig. 2A-B and Figure S2, it is recommended that the authors directly present the OD450 values instead of the converted fold change. In addition, if the authors want to highlight that certain mutations cause changes in the proportion of pre-fusion conformation, the horizontal and vertical axes of each subgraph should adopt the same range. In this way, the points above the diagonal (or a certain normalized line) will represent an increase in the proportion of pre-fusion conformation, and the upper right quadrant will correspond to an increase in expression level.

10. Line 214. It may be a typo, which should be M289F.

11. Lines 223-224: The expression "disulfides did not improve..." seems inconsistent with the results presented in the middle graph of the second row of Figure S3. Fig. S3A shows that the N67L-V90Y-S190I-M289F-D486S protein has aggregations, while the N67L-V90Y-S190I-M289F-T267P-N268P-V482C-S502C-D486S protein has no apparent aggregates. From the current results, it is clear that disulfide bonds help enhance the homogeneity and stability of the trimer. In addition, from the results presented in Fig. S5D, I speculate that the authors used proteins purified in different batches for detection at the same time point to characterize the changes in protein homogeneity after being placed at different temperatures for different times. To make the experimental results more rigorous, I suggest that if possible, the authors present the SEC analysis of the same batch of purified protein samples stored for different times and overlay the results. Otherwise, the authors should indicate in the text that the original proteins used in Figs. S3A, S5B, D, etc., are not from the same batch.

12. Line 227. It is recommended that the authors remove the expression "minimal".

13. Line 231. The authors should clearly define "optimal", that is, through which indicators in Fig. S4 can it be quantitatively considered that the selected mutations are optimal. Otherwise, it is recommended to weaken the relevant tone.

14. Line 254. Although the expressions L88A and L88B are not incorrect, they are likely to be misunderstood as certain point mutations. It is recommended that the authors rename these two constructs throughout the full text to enhance the readability of the manuscript.

15. Lines 258-261. What are the specific storage conditions? In which buffer is the protein stored? Is it in a lyophilized state? If it is lyophilized, what is the reconstitution buffer? Was centrifugation performed to remove precipitates before SEC analysis? In Fig. S5, the sample stored at -80{degree sign}C is labeled as L; is this the abbreviation of lyophilization? The authors should clearly state these details in the corresponding parts of the main text and methods.

16. Line 380. The expression "consistently higher" needs to be obtained after statistical significance analysis of the data, which is lacking in the figure legends of current Figs. 5B and C.

17. Line 388. I did not observe "marked weight loss" in the results provided by the authors, and since only the fourth day was observed, the weight change data are of little significance. It is recommended to remove them.

18. Line 445. The authors need to explain the specific meaning of "conformational breathing" or provide the corresponding reference literature.

19. Line 453. Conclusions that N67L can "reinforced apex stability" cannot be drawn only from the analysis of the structural model, and the current structure has D25 antibody binding, making it even more impossible to judge whether the stability improvement is brought about by the introduction of N67L. It is recommended that the authors provide data such as the B-factor of the crystal structure or other observable data as evidence. Otherwise, the relevant expressions may be considered for removal.

20. The following remark is for the current Fig. 6. It is recommended that the authors try to avoid using red, blue, and yellow to display side chain residues to prevent confusion with oxygen, nitrogen, and sulfur atoms.

21. Please the authors check that the color code of each curve in a figure should be consistent, and fully correct the labels such as F1 and F3 to correspond to the main text. Other expressions such as L-12M should be explained in full in the figure legend. This remark applies to multiple figures including Fig. S5.

22. There seems to be a problem between the Model resolution range and Model resolution columns in Table S2. Please the authors check the content in Table S2 and verify it.

Referee #3 (Comments on Novelty/Model System for Author):

Overall, this work ranks very strongly in identifying a new set of RSV preF-stabilized molecules that could be used as antigens for RSV vaccination. However, the superiority of this vaccine over other approved vaccines is not clear. Furthermore, safety data is inconclusive.

Referee #3 (Remarks for Author):

Structure-guided rational 1 design of a compact prefusion RSV F bivalent vaccine with enhanced immunogenicity (Liu et al.)

This work shows a newly designed Pre-fusion F (preF) protein from RSV of the A and B subgroups. The proposed new trimers of preF were generated through structure-guided design, and high-throughput screening was performed using specific mAbs (Motavizumab and D25). The screening and analysis yielded two new preF designs, which were further studied for immunogenicity, efficacy, and safety in mouse and cotton rat models of RSV A and RSV B infection. Comparisons were conducted with ABRYSVO, a recently approved bivalent RSV vaccine of similar composition.

Results from animal models show mild improvements in the generation of neutralizing and total antibodies by the candidate (RVF-L88) COMPARED TO ABRYSVO given at comparable doses in both models, with no statistical significance. Real numbers for serum neutralizing antibody titers should be presented in a table for comparison with previously published studies.

Protection was also analyzed in both models and correlated with the mild improvement in immunogenicity achieved by RVF-L88. In the mouse model (Figure 4), the animals were challenged only with RSV A subtype, and improvement was observed only at the level of RSV RNA analysis. In the cotton rat model, the challenge was performed with both RSV subtypes. Some improvement in lung protection against RSV B and in nasal protection against A2 was observed (Figure 5).

Safety studies shown are very basic. In cotton rats, body weight changes were observed, which are not a good indicator of RSV pathology (in fact, contrary to the mouse model, cotton rats do not lose weight after RSV infection). In addition, Fig. S13 shows lung sections stained with H&E from underinflated samples, which are difficult to compare and from which it is not possible to conclude that RVF-L88 "displayed minimal focal inflammatory lesions." As this model is the established safety model for vaccine-enhanced disease (VED), positive controls should be used and different vaccine doses tested, as VED could arise from vaccination with lower doses.

Overall, this work ranks very strongly in identifying a new set of RSV preF-stabilized molecules that could be used as antigens for RSV vaccination. However, the proof of superiority over ABRYSVO antigens (generated using a similar rationale) at the same dose is not convincing, as the data from both mice and cotton rats show similar results. Furthermore, very preliminary safety studies are presented, resulting in inconclusive findings.

Dear Editor,

Thank you for your decision letter regarding our manuscript entitled "**Structure-guided rational design of a prefusion RSV F bivalent vaccine with enhanced immunogenicity**" (EMM-2026-23468-V2). We sincerely appreciate the valuable comments and suggestions provided by you and the reviewers, which have helped us improve the manuscript substantially.

We have carefully revised the manuscript accordingly, including clarification of the structural design rationale, refinement of data interpretation, and addition of new analyses and supplementary data. In addition, for nomenclature consistency, the construct previously referred to as RVF-L88 has been renamed RVF-88 throughout the revised manuscript and in the responses below.

We hope that the revised manuscript is now suitable for publication in EMBO Molecular Medicine. Detailed point-by-point responses and corresponding manuscript changes are provided below.

Sincerely,
Lijie Liu

******* Reviewer's comments *******

Referee #1 (Remarks for Author):

Review 'Structure-guided rational design of a compact prefusion RSV F bivalent vaccine with enhanced immunogenicity'. This article describes the design and testing of a potential new RSV prefusion F vaccine. The study is well carried out and described in a detailed way. In general I feel the writers could have done more comparison with the DS-Cav1 molecule, which was the first prefusion stabilized version of RSV F, as they also refer to this molecule in the introduction and the discussion.
Below you can find my comments (mostly minor comments).

Line 177: What do you mean with 7 days (day10)?

Response:

We thank the reviewer for this comment. Here, "7 days" refers to 7 days after the supernatant was collected and stored, which corresponds to day 10 after transfection. We have clarified this point in the revised manuscript.

Changes in the manuscript:

1. **Figure 1C and the corresponding legend:** We have been revised to clarify the meaning of 7 days. Page 10, Lines 180.
   "Portions were subjected to heat treatment at 55 °C for 1 hour or stored at 4 °C for 7 days (corresponding to day 10 post-transfection)."

Line 189, what is the rationale for looking at 7days? In Flynn et al, they wait 14d, Zhang et al 1 month. How would DS-Cav1 score in this analysis?

Response:

We thank the reviewer for this important comment. The 7-day incubation at 4 °C was used as a rapid early-stage screening readout for evaluating the relative stability of RSV F variants, based on a screening approach similar to that used in the foundational work of McLellan et al. (Science, 2013). We agree that the 14-day time point in Flynn et al. and the 1-month time point in Zhang et al. were used to evaluate the longer-term conformational stability of purified DS-Cav1 protein during storage. In contrast, the 7-day incubation used in our study was designed as a rapid early-stage screening assay to identify RSV F variants with improved preservation of prefusion antigenicity for downstream candidate selection, rather than to define the long-term shelf stability of purified protein preparations. We have clarified the scope and rationale of this 7-day assay in the revised manuscript.

To address the reviewer's question regarding how DS-Cav1 would score in this analysis, we performed an additional side-by-side comparison using the same transient-transfection and supernatant-based ELISA workflow. Specifically, selected variants carrying favorable substitutions from the initial screen, including the mutations highlighted in red in Fig. 2A and 2B, were compared side by side with DS-Cav1, DS, and Cav1 constructs after transient transfection in 293T cells. Culture supernatants were analyzed either directly or after incubation at 4 °C for 7 days, followed by ELISA detection of binding to the prefusion-sensitive antibodies D25 and Motavizumab. As shown in the newly added Appendix Fig. S3, after 7 days of incubation at 4 °C, most of the selected variants exhibited stronger D25 and/or Motavizumab binding than DS-Cav1 in this screening-format assay, consistent with better preservation of prefusion-like antigenic features following short-term cold storage.

Changes in the manuscript:

1. **Results section:** We revised the text to clarify the rationale for the 7-day 4 °C screening window and added the citation to McLellan et al. (2013):
   Page 11, Lines 190–194:
   "At this stage, we examined individual F substitutions, including engineered cysteine pairs designed to introduce disulfide bonds, and assessed prefusion antigenicity and short-term retention of antibody binding using a 7-day screening window at 4°C, a duration previously established for identifying stabilized RSV F variants (McLellan et al., 2013a)."
2. **Methods section:** We revised the Methods to clarify the rationale and experimental scope of the 7-day 4 °C screening assay used for early-stage variant prioritization.
   Page 41, Lines 722–728:
   "A portion of each supernatant was immediately analyzed by ELISA to compare preF and postF expression levels, while the remaining aliquots were subjected to stability testing, either heat-treated at 55 °C for 1 h or stored at 4 °C for one week before further analysis, consistent with established RSV F screening protocols (McLellan et al., 2013a) to facilitate discrimination of relative variant stability during early-stage screening."
3. **Methods section:** We revised the Methods to clarify normalization of ELISA-based screening data across independently conducted screening rounds:
   Page 44, Lines 792–798:
   "For all ELISA-based screening assays, binding signals were normalized to the concurrently processed wild-type RSV F (A2 strain) control within each experimental batch, and the results are expressed as log2-transformed fold changes relative to wild-type F. This within-batch normalization was applied to minimize the influence of batch-to-batch variation inherent to transient transfection-based expression and to enable comparison of antigenicity across independently conducted screening rounds."
4. **Supplementary figure legend:** We added Supplementary Appendix Fig. S3 and its corresponding legend to present the DS-Cav1/DS/Cav1 comparison.

5. **Results section / Appendix Fig. S3:** We added a new supplementary comparison of selected substitutions with DS-Cav1, DS, and Cav1 under the same HEK293T supernatant ELISA screening workflow:
   Page 12, Lines 218–224:
   "A subset of 13 substitutions, selected based on D25 binding strength and positional representation, was further evaluated by ELISA alongside DS-Cav1, DS, and Cav1 using the same HEK293T supernatant-based screening workflow. Most selected variants showed higher D25 or Motavizumab reactivity than DS-Cav1 both at 72 h post-transfection and after 7 days at 4 °C, providing additional support for their prioritization in downstream combinatorial analysis (Appendix Fig. S3)."

Reference:

McLellan JS, Chen M, Joyce MG, Sastry M, Stewart-Jones GBE, Yang Y, Zhang B, Chen L, Srivatsan S, Zheng A, et al (2013a) Structure-Based Design of a Fusion Glycoprotein Vaccine for Respiratory Syncytial Virus. Science 342: 592–598

Fig2a and b, what do the other colors mean? Please signify or change to black. It seems that some of these should be red?

Response:

We thank the reviewer for pointing this out. We agree that the original color coding was not sufficiently clear. In the original figure, red was used to highlight favorable substitutions, whereas the additional colors were used only to distinguish different cavity-filling sites. To improve clarity, we have simplified the revised figure so that favorable substitutions are shown in red and labeled directly, while the remaining substitutions are shown in black. The figure legend has also been revised accordingly.

Changes in the manuscript:

1. **Figure 2A-B and the corresponding legend:** We have been revised to clarify the meaning of the color coding.
   Page 16, Lines 269–271.
   "Red symbols indicate shortlisted candidates selected for downstream combinatorial analysis."

Fig 3a. Line 286; should be upper and lower instead of left and right; it is not clear what F1 and F3 means.
Fig 3c: square in stead of site zero symbol; no site mentioned for AM14

Response:

We thank the reviewer for the careful observation. We have corrected the directional description from "left and right" to "upper and lower" to accurately reflect the layout of Figure 3A. To avoid any potential misunderstanding caused by the abbreviations F1 and F3, we have revised the SEC trace labels to "1 freeze–thaw" and "3 freeze–thaw," respectively, while RT-7D indicates storage at ambient temperature for 7 days. These clarifications ensure that the stability stress conditions for RVF-88A and RVF-88B are clearly understood.

For Fig. 3C: We have revised the table to use the standard Site Ø symbol instead of the square. We have also added specific antigenic site annotations for AM14 (trimer-specific quaternary epitope) to provide clearer structural context.

We apologize for these clerical oversights and have updated both the figures and the corresponding legends in the revised manuscript.

Changes in the manuscript:

1. **Figure 3A and the corresponding legend:** We have been revised to correct the directional description and clarify the abbreviations used for stress conditions. The SEC trace labels were changed from "F1" and "F3" to "1 freeze–thaw" and "3 freeze–thaw," respectively, to avoid ambiguity.
   Page 19, Lines 319–322.
   "(A) SEC profiles of CHO-expressed RVF-88A (upper) and RVF-88B (lower) under freeze-thaw and ambient storage stress conditions. Traces are color-coded by condition: blue (one freeze-thaw cycle), red (three freeze-thaw cycles), and green (RT-7D, ambient temperature storage for 7 days)."
2. **Figure 3C:** We have been revised to replace the square symbol with the standard Site Ø notation of D25 and to annotate the antigenic site information for AM14.
   Page 18, Lines 317.
   "D25, Ø", "AM14, trimer-specific."

It would be interesting to also confirm lack of postfusion f with a site I specific antibody.

Response:

We thank the reviewer for this helpful suggestion. ADI-14359 has been reported as a site I-directed antibody that preferentially recognizes postfusion RSV F and has been structurally characterized in complex with postfusion F (Goodwin et al., 2018; PDB: 6APB). We therefore used ADI-14359 as an additional postfusion-reactive antibody readout and the ELISA result is shown below (Figure R1).

In the ADI-14359 ELISA, postF-A and postF-B showed strong binding, confirming the expected reactivity of the assay. In contrast, RVF-88B showed minimal ADI-14359 binding across the tested antigen concentrations, while RVF-88A showed only weak binding at high antigen concentrations and remained substantially lower than the postF controls. These results indicate that RVF-88A and RVF-88B did not show strong postfusion-like ADI-14359 reactivity under the tested conditions, although a limited high-concentration signal was observed for RVF-88A.

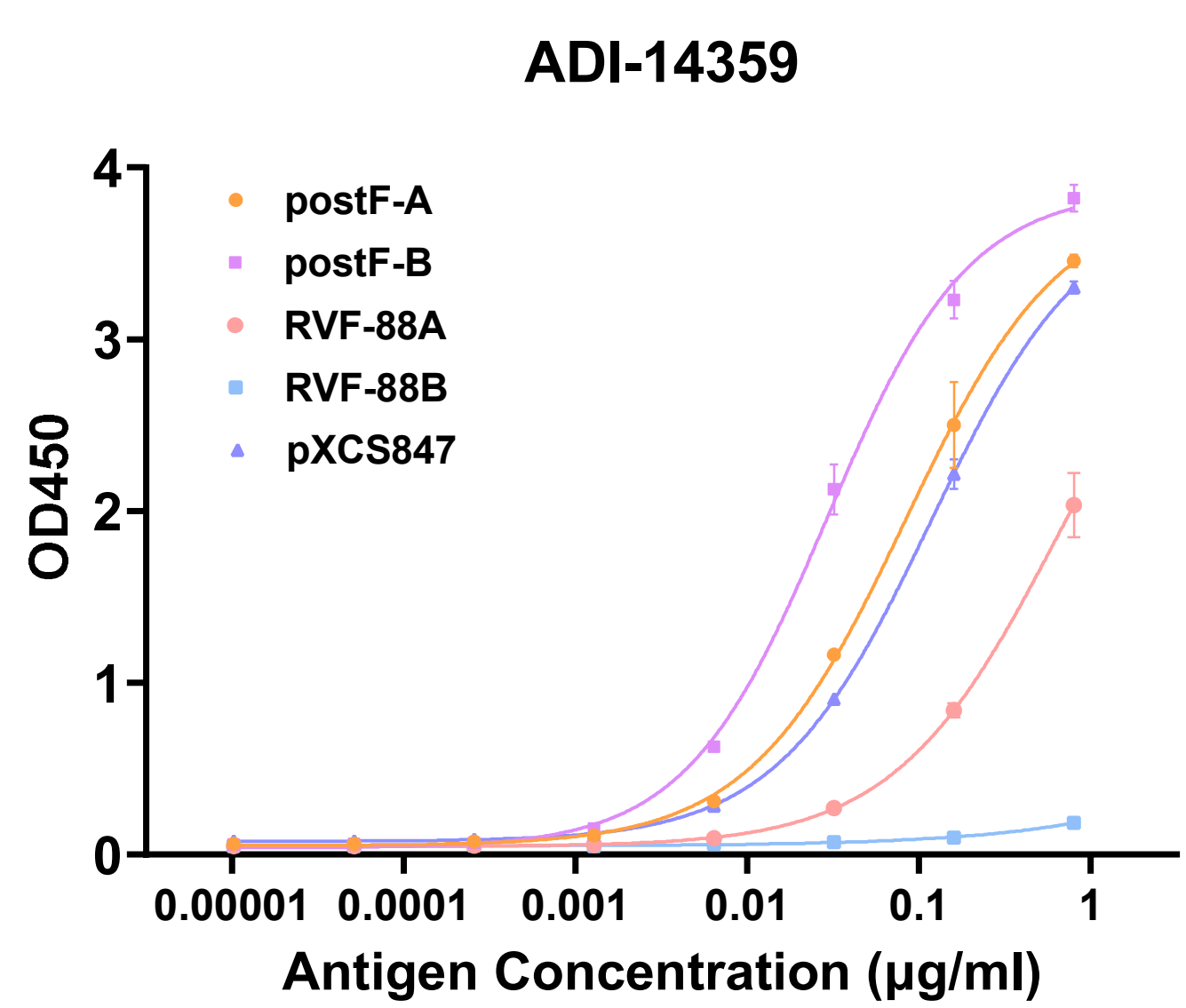

Figure R1. ADI-14359 binding to RVF-88 antigens and postfusion F controls.

Binding of ADI-14359 to postF-A, postF-B, RVF-88A, RVF-88B, and pXCS847 was measured by ELISA across the indicated antigen concentrations. Data are shown as mean ± SD of technical replicates.

Reference:

Goodwin E, Gilman MSA, Wrapp D, Chen M, Ngwuta JO, Moin SM, Bai P, Sivasubramanian A, Connor RI, Wright PF, et al (2018) Infants Infected with Respiratory Syncytial Virus Generate Potent Neutralizing Antibodies that Lack Somatic Hypermutation. Immunity 48: 339-349.e5

Fig 4 b and c, what antigens are used for coating? RVF L88A and B? Maybe also check binding to another prefusion F like DS-Cav1?

Response:

We thank the reviewer for this helpful suggestion. For the serum binding assays shown in Fig. 4B and 4C, the coating antigens were the corresponding immunogens under study, namely RVF-88A and RVF-88B, respectively. We have clarified this information in both the revised Figure 4 legend and the Methods section.

In response to the reviewer's suggestion, we further evaluated Day 35 serum binding to DS-Cav1, a well-established prefusion RSV F benchmark antigen, in parallel with RVF-88A. The RVF-88-immunized sera showed detectable binding to DS-Cav1, and the overall binding pattern was similar to that observed using RVF-88A as the coating antigen. A similar pattern was also observed for the ABRYSVO-immunized sera. These results indicate that the serum binding response was not restricted to the homologous RVF-88A coating antigen, but was also detectable against an independent prefusion F antigen. The corresponding DS-Cav1/RVF-88A serum-binding results are shown below (Figure R2).

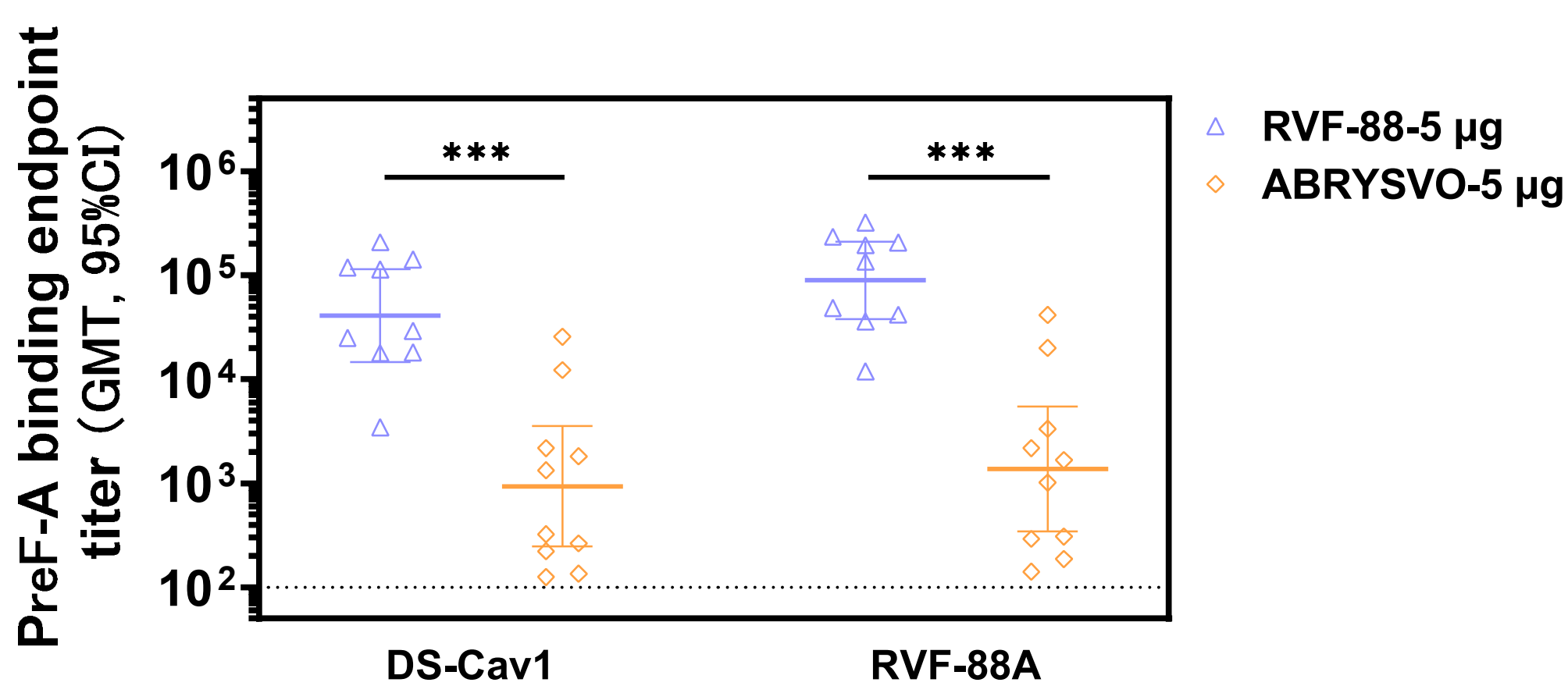

Figure R2. Day 35 serum binding IgG titres to DS-Cav1 and RVF-88A.

Day 35 sera from RVF-88- and ABRYSVO-immunized mice were tested by ELISA using DS-Cav1 or RVF-88A as the coating antigen. Symbols represent individual mice; horizontal lines indicate geometric mean titers (GMT) ± 95% confidence interval (CI).

Changes in the manuscript:

1. **Figure 4 legend:** We clarified the coating antigens used in the serum binding assays shown in Fig. 4B and 4C.
   Page 24, Lines 395–397:
   "PreF-A-specific (B) and PreF-B-specific (C) serum binding IgG titres measured using RVF-88A and RVF-88B, respectively, as coating antigens at the indicated time points."
2. **Methods section:** We revised the serum ELISA Methods to specify the coating antigens used for the Fig. 4B and 4C binding assays.
   Page 43, Lines 774–776:
   "For serum binding (indirect) ELISA, plates were coated overnight at 4 °C with 2 µg ml^{-1} of purified RVF-88A or RVF-88B as the coating antigens for the respective assays."

Line 312: the dose dependent effect is only minimal, probably no significant difference?

Response:

We thank the reviewer for this insightful observation. We have performed a comprehensive statistical analysis using Tukey's multiple comparison test across all key assays, including ELISA binding titers, neutralizing activity, and post-challenge viral loads (both titers and RNA copies).While RVF-88 generally showed dose-dependent trends across several readouts, differences among the 2, 5, and 12.5 µg groups did not reach statistical significance ($P > 0.05$) under the tested conditions. We have therefore revised the Results section to more accurately describe the observed dose-response relationship and avoid overinterpretation of the dose effect in the BALB/c mouse model.

Changes in the manuscript:

1. **Results section:** We revised the description of the dose-response relationship to reflect the statistical findings.
   Page 20, Lines 345–348.
   "Although RVF-88 immunogenicity and protection generally showed dose-dependent trends, differences among the 2, 5, and 12.5 µg groups did not reach statistical significance ($P > 0.05$) across most binding-antibody, neutralization, and viral-burden endpoints under the tested conditions."

Fig 4d, some mice do not show neutralizing titers at d35, with 5 µg vaccine, do you have an explanation for this?

Response:

We thank the reviewer for this careful observation. We agree that in Fig. 4D, several mice in the 5 µg RVF-88 group showed neutralizing titers against RSV A2 that were near or below the limit of detection at day 35. We do not have a definitive mechanistic explanation for why these individual animals showed lower neutralizing activity; however, such inter-animal variability is not unexpected in unadjuvanted protein immunization models, particularly when neutralization rather than total binding antibody is used as the readout.

In support of this interpretation, similar animal-to-animal variability has been reported in RSV preF protein immunization studies. For example, in the Pfizer-reported stabilized RSV F construct study (Che et al, 2023), individual mice immunized with unadjuvanted prefusion F constructs also showed variable neutralizing responses, with some animals displaying titers close to the limit of detection (shown in the below). In our study, we therefore used 10 mice per group to reduce the influence of individual non-responders on group-level interpretation.

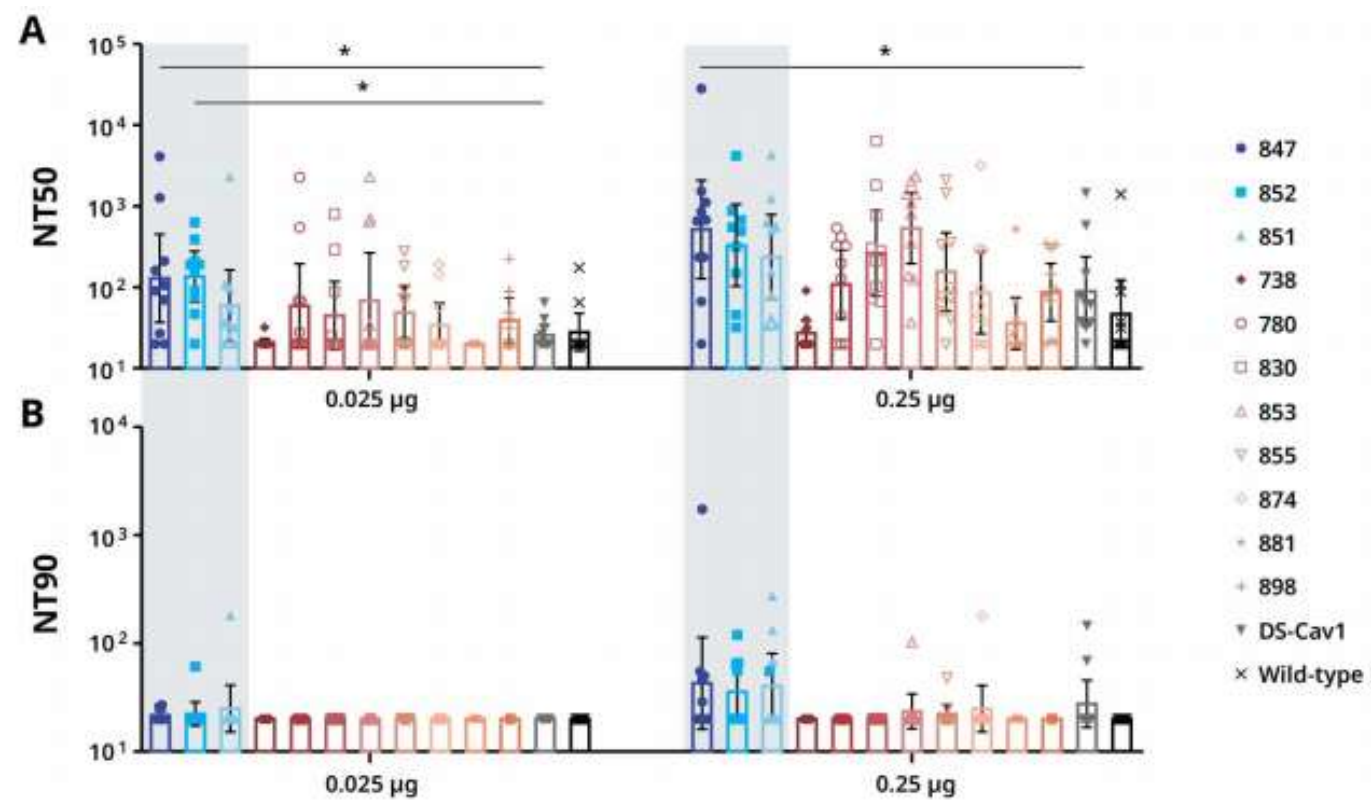

Fig. S3. Mouse immunogenicity of 11 stable prefusion F constructs. Mice (10/group) were immunized intramuscularly at weeks 0 and 3 with 0.025 µg or 0.25 µg of the indicated constructs without $Al(OH)_3$. Neutralizing titers were assessed in week 5 sera. Wild-type F contains a native RSV F ectodomain sequence with no stabilizing mutations and is in the postfusion conformation. Data are displayed as (**A**) 50% (NT50) and (**B**) 90% neutralizing geometric mean titers (NT90) for each construct. Each symbol represents a titer from an individual animal. Limit of detection = 20. Grey boxes highlight the three constructs selected for further immunogenicity testing. For statistical comparisons for panel A (dose 0.025 and dose 0.25 µg), NT data were ranked within each dose level and ANOVA was used. For panel B, 0.025 µg dose level, NT 20 (limit of detection) was assigned into one category and any titer above 20 as the other category followed by an exact statistical method described by Farrington and Manning for statistical comparisons (*54*). *, PreF 847 elicited significantly higher NT50 responses compared to DS-Cav1 at both dose levels ($p < 0.005$ for 0.025 µg dose level; $p < 0.03$ for 0.25 µg dose level).

Importantly, the low neutralizing titers in these individual mice did not correspond to an absence of antigen recognition, as these animals retained detectable preF-binding IgG responses. A similar pattern of variability was also observed in the ABRYSVO benchmark group, suggesting that this was not unique to RVF-88. Overall, this variability did not alter the group-level GMT analysis or the conclusion that RVF-88 induced robust binding and neutralizing antibody responses under the conditions tested.

Reference:

Che Y, Gribenko AV, Song X, Handke LD, Efferen KS, Tompkins K, Kodali S, Nunez L, Prasad AK, Phelan LM, et al (2023) Rational design of a highly immunogenic prefusion-stabilized F glycoprotein antigen for a respiratory syncytial virus vaccine. Science Translational Medicine 15: eade6422

What was the reason not to include an adjuvant? Overall neutralization titers seem quite low

Response:

We thank the reviewer for these important questions regarding the use of adjuvants and the magnitude of the neutralizing titers. The decision not to include an adjuvant was intentional. First, our aim was to evaluate the intrinsic immunogenicity of the RVF-88 antigens themselves, without the confounding effects of exogenous immune stimulation. This design allowed us to assess whether RVF-88 could induce measurable humoral responses based on the antigen design alone.

Second, the use of an adjuvant-free formulation was also motivated by translational and safety-related considerations. RSV vaccines are intended for populations that may include older adults, at-risk adults, and pregnant individuals; therefore, avoiding exogenous adjuvants can minimize adjuvant-related reactogenicity considerations and reduce formulation-related variables during early antigen evaluation.

Third, this formulation also enabled a direct comparison with the clinical benchmark ABRYSVO under matched adjuvant-free conditions. ABRYSVO is an approved unadjuvanted RSV prefusion F protein vaccine with indications in maternal immunization, older adults, and adults aged 18–59 years who are at increased risk for RSV-associated LRTD. We agree with the reviewer that the overall neutralizing titers in the mouse study were modest. However, similar modest or variable neutralizing responses have been observed in unadjuvanted RSV preF protein immunization studies, including Pfizer-reported stabilized preF constructs. Therefore, the magnitude observed in our BALB/c mouse model is consistent with what may be expected for low-dose, unadjuvanted protein immunization. Importantly, despite the modest absolute titers, RVF-88 induced detectable binding and neutralizing antibody responses under the same adjuvant-free conditions used for the ABRYSVO comparison.

Figure 4G is redundant, showing 4H is enough.

Response:

We thank the reviewer for this helpful suggestion. We agree that Figure 4G was redundant with Figure 4H. We have therefore removed Figure 4G from the revised manuscript and retained Figure 4H to present the body weight change data more concisely. All subsequent panels and figure legends have been renumbered accordingly to ensure clarity and consistency.

Changes in the manuscript:

1. **Figure 4 and legends:** We removed redundant panel 4G and updated figure numbering and legend accordingly.
   Page 23, Line 390. Page 24, Line 403.
   The redundant panel showing absolute body weight change was removed from Figure 4 to improve clarity. The remaining panels were renumbered accordingly, and the figure legend and all in-text citations were updated to reflect the revised figure layout.

Line 326, maintained at lower level? Not at the detection limit for 5 and 12.5 µg of RVF-L88? Could you indicate the detection limit on the graph? (4J) (also for the viral titers in the cotton rat experiment)

Response:

We thank the reviewer for this careful observation. Upon re-evaluating the data against a formally calculated limit of detection (LOD), we have confirmed that the viral titers in the 5 µg and 12.5 µg RVF-88 groups actually fell below the LOD. The LOD was calculated based on the lowest initial dilution and the minimum detectable unit (1 FFU) per sample, adjusted for the minimum lung weight within each species. LOD values were determined independently for mouse and cotton rat experiments based on their respective assay parameters and minimum recorded lung weights. We have now indicated this LOD as a dashed horizontal line in Figure 4J (renumbered as 4I) and the corresponding cotton rat panels. Accordingly, the Results section has been revised to state that these high-dose groups achieved viral clearance to undetectable levels. We have also added the detailed LOD calculation formula to the Materials and Methods section for full transparency.

Changes in the manuscript:

1. **Figure 4 and legends:** We added limit of detection (LOD) reference lines and updated figure legends accordingly.
   Page 24, Line 411–412.
   "Dashed lines indicate the limit of detection in panels B-E, H, and I, and no change from initial body weight (0%) in panel G."
2. **Methods:** We clarified the definition and calculation of the limit of detection (LOD) for the focus-forming assay.
   Page 52, Line 972–974.
   "The limit of detection for viral titer assays was defined based on assay sensitivity (1 FFU detection threshold) and adjusted using species-specific minimum tissue weights."
3. **Results section:** We clarified that high-dose RVF-88 immunization reduced viral titers below the limit of detection.
   Page 21, Line 370–372.
   "In particular, the 5 μg and 12.5 μg RVF-88 doses demonstrated exceptional efficacy by reducing viral titers to undetectable levels below the limit of detection (LOD) (Fig. 4I)."

PDF issue with Fig s12, some letters in the Y-axis appear very bold

Response:

We apologize for this formatting and rendering issue in the PDF version of the manuscript. We have carefully revised Appendix Figure S12 (now Appendix Figure S13) to ensure that all axis labels and text are displayed with consistent font weight and clarity. The figure has been replaced with a high-resolution version in the revised submission to prevent any similar rendering artifacts.

Changes in the manuscript:

1. **Appendix Figure S13 (formerly Appendix Figure S12):** We have replaced the figure with a corrected version to resolve the PDF rendering issue and ensure consistent font display in the Y-axis labels.

5C: what does the dotted line mean

Response:

We thank the reviewer for this observation. The dotted line in the original Figure 5C was intended to represent a technical reference level based on Palivizumab neutralization in vitro. However, upon further evaluation, we realized that this reference was not sufficiently defined in the legend and may lead to ambiguity regarding the assay's dynamic range. Since the core conclusion of Figure 5 relies on the relative comparison between the immunized groups and the benchmark, we have removed this dotted line to maintain a clean and focused data presentation. All essential data points and statistical comparisons remain unaffected.

Changes in the manuscript:

1. **Figure 5C:** We have removed the dotted line from the figure for clarity.

Line 388: Weight loss is still quite minimal, as is expected with cotton rats

Response:

We thank the reviewer for this insightful comment. We agree that body-weight change is generally a less sensitive marker of RSV morbidity in the cotton rat model compared to the mouse model. In our study, the observed body-weight fluctuations were indeed minimal and did not provide additional informative value regarding vaccine efficacy. To maintain a focused and concise presentation of the most relevant results (namely viral clearance and lung pathology), we have removed the body-weight data and deleted the corresponding text in the revised manuscript to avoid any potential overinterpretation.

Changes in the manuscript:

1. **Results section:** We have removed the text describing cotton rat body-weight changes.
   Page 25, Line 431.
2. **Figure 5:** We have removed the corresponding body-weight data and revised the figure numbering accordingly.

Line 401: yet in 5K, there is no difference in the inflammation scores between the different conditions. This is not consistent with the text.

Response:

We sincerely thank the reviewer for pointing out this discrepancy. We agree that the quantitative inflammation scores in Figure 5K and 5L (now Fig. 5I, J) did not reach statistical significance between groups, and we apologize for the overstatement in the original text.

The lack of statistical separation in histopathology, despite robust viral clearance, may reflect the temporal dissociation between viral neutralization and the resolution of inflammatory infiltrates in RSV infection models. In addition, variability in tissue processing and histopathological scoring may have introduced baseline noise, which could reduce the sensitivity to detect subtle intergroup differences. We have revised the Results sections accordingly to provide a more descriptive and cautious interpretation that aligns with the quantitative data, emphasizing virological outcomes as the primary indicator of efficacy.

Changes in the manuscript:

1. **Results section:** We revised the description of histopathological scoring to accurately reflect the lack of statistical significance between groups.
   Page 26, Line 440–444.
   "Representative H&E-stained lung sections from buffer-, ABRYSVO-, and RVF-88-immunized cotton rats after viral challenge were generally consistent with the quantitative histopathology scores shown in Fig. 5I and 5J and did not indicate increased lung inflammatory pathology in the RVF-88 group under the tested conditions (Appendix Fig. S14)."

Why don't you show the increased packing with the cavity-filling substitution at site 190 in Fig. 6c?

Response:

We thank the reviewer for this helpful suggestion. We have revised Fig. 6C to include the local structural environment around the S190I substitution at site 190. The updated panel now displays the I190 side chain and neighboring residues in the RVF-88-D25 structure, allowing the cavity-filling context and local contacts around this site to be visualized more clearly.

We have also revised the corresponding Results text and Fig. 6 legend to describe the S190I-associated local contacts more explicitly. Because the local contact environment of I190 can be construct-dependent, we now describe the S190I feature based on the observed RVF-88-D25 structure and avoid implying that the side-chain contacts are identical to those reported for other S190I-containing constructs such as pXCS847A.

Changes in the manuscript:

1. **Figure 6C:** We revised Fig. 6C to include the local structural environment around S190I at site 190, showing the I190 side chain and neighboring residues in the RVF-88-D25 structure.
2. **Figure 6 legend:** We revised the Fig. 6C legend to indicate that the panel now includes S190I together with neighboring residues:
   Page 32, Lines 536–538.
   "(C) Local views around N67, V90, S190I and M289 in RVF-88A, RVF-88B, and pXCS847A. Site residues are highlighted in magenta (stick) together within 4 Å of the displayed atoms (stick)."
3. **Results section:** We updated the Results text to describe the added S190I structural view:
   Page 30, Lines 502–507:
   "The S190I substitution places a β-branched hydrophobic side chain within the local cavity at site 190, where close contacts with neighboring residues are observed in the RVF-88-D25 structure. Although S190I has also been used as a cavity-filling substitution in pXCS847A, the neighboring contacts around I190 differ between the two constructs, indicating that the same substitution can be accommodated through distinct local structural environments."

Line 510: How is manufacturing complexity improved, you did not show that this construct leads to greater yields compared to other constructs.
Line 511: yet you never compare to DS-Cav1

Response:

We thank the reviewer for this important comment. We agree that the original wording regarding improved manufacturing complexity was too strong, as we did not directly evaluate production yields or perform head-to-head manufacturing comparisons with DS-Cav1 in this study.

Our intention was to convey that RVF-88 achieves prefusion stabilization through a distinct design philosophy—utilizing structure-guided hydrophobic reinforcement and charge rebalancing rather than relying on engineered disulfide bonds. We have revised the text to remove the unsupported implication of a demonstrated manufacturing advantage. Furthermore, we have clarified that terms such as "light-lock" are used as conceptual descriptors for this disulfide-free engineering strategy, rather than a claim of superiority in production.

Changes in the manuscript:

1. **Discussion section:** We revised the description of the RVF-88 design framework to clarify the intended meaning of the "light-lock" concept and to remove any implication of manufacturing or production advantage.
   Page 33, Line 550–554.
   "The final construct embodies what we refer to as a "light-lock" design concept, defined here as prefusion stabilization without the introduction of exogenous covalent disulfide locks. This concept differs from disulfide-containing prefusion F designs, such as DS-Cav1, which incorporate engineered cysteines together with additional stabilizing mutations."
   Page 33, Line 563–566.

"In addition, recent work has continued to explore alternative prefusion-stabilization strategies to improve RSV preF antigen stability and manufacturability (Huang et al, 2025; Song et al, 2025)."

Line 654: you probably mean Targetmol

Response:

We apologize for this typographical error. The company name "Targetmol" is indeed correct. We have corrected this spelling throughout the revised manuscript to ensure accuracy.

Changes in the manuscript:

1. **Methods section:** We corrected a typographical error in the reagent supplier name.
   Page 42, Line 745.
   The company name "Targetlol" was corrected to "Targetmol".

Referee #2 (Comments on Novelty/Model System for Author):

Overall, the choice for current cotton rat model is well accepted in studying RSV related infections, except that body weight change is not significance in cotton rat and I would suggest the author to remove the body weight change graph from their manuscript.

Referee #2 (Remarks for Author):

This manuscript describes a method for screening and rational design of RSV pre-F proteins based on known structures, aiming to develop an immunogen with fewer mutations compared to DS-Cav2 or ABRYSVO while maintaining the necessary pre-F-specific immunogenicity. The authors named this immunogen L88 (composed of two antigens, L88A and L88B) and systematically conducted preclinical studies on the adjuvant-free vaccine formulated with this combination. The full text is generally divided into two parts: the screening process of candidate immunogens mainly based on tool antibody ELISA, and the in vivo and in vitro evaluation of the final construct selected by the authors. Overall, the idea of this manuscript is easy to read, and the data are relatively abundant.
However, its most critical shortcoming is that neither the screening method, the selection strategy of candidate mutation sites, nor the evaluation method and results of the immunogen can well reflect the novelty of this manuscript. In addition, I have concerns about the effectiveness and necessity of the screening strategy adopted. The authors need to clearly indicate why the finally selected five-mutation construct is rationally optimal, and discuss the logical relationship between the selection of this construct and the screening strategy as well as the inevitability of obtaining this construct. The authors pointed out in the discussion that they avoided the use of disulfide bonds in their design to optimize antigen properties, but I did not see the rationality of this consideration from the results they presented. I also have some questions about the logic of the authors' immunogen screening. In addition, there are several problems in the writing of this manuscript, such as typos, inconsistent colors, and unclear experimental methods. It is hoped that the authors will pay attention to the rigor of the writing.

I will elaborate on my questions in detail below:

//Major Concerns//

1. Regarding the rationality and originality of mutation site selection. The authors spent a large part of the first section of the results describing the conformational differences between pre- and post-fusion. Since this is already a widely accepted fact, it is recommended to drastically abbreviate this part. The four-point modification strategy selected by the authors (Lines 126-128) has been systematically reported in previous studies, all based on static structural analysis of pre- and post-fusion. If the authors cannot emphasize the fundamental difference between their screening logic and previously reported ones on this basis, this part should be elaborated in the introduction rather than in the results. The originality here refers not only to the addition or reduction of specific individual sites, but also to the logical basis at the molecular mechanism level of their strategy. It is recommended that the authors re-explore and make a parallel comparison between their selected optimization strategy and all existing reported design methods for stabilizing pre-F to highlight the novelty of their design strategy. This is the key innovation of this manuscript, and it is recommended that the authors rewrite this part to highlight what is novel in their design methodology.

Response:

We thank the reviewer for this important comment. We agree that the current first part of the Results section devotes too much space to general prefusion/postfusion structural differences, whereas the key point that should be emphasized is the design logic derived from those comparisons. We also agree that the four stabilization categories considered in this study—disulfide engineering, proline substitution, hydrophobic cavity filling, and electrostatic optimization—have all been described previously in RSV pre-F design

studies. We have therefore substantially revised this part of the manuscript to distinguish more clearly between established background knowledge and the methodological contribution of the present work.

In the revised manuscript, we now emphasize that the methodological contribution of this work is not the isolated use of any single known stabilization category, but the way these categories were organized, region-prioritized, and experimentally filtered within a unified design framework. Specifically, comparative structural analysis was used not merely to describe prefusion/postfusion differences, but to partition the RSV F ectodomain into regions with different degrees of conformational conservation or divergence during fusion. This structure-partitioned analysis allowed us to prioritize conformationally divergent regions, especially the prefusion apex and adjacent supporting structural elements, for focused mutational interrogation. In practice, this prioritization was implemented as a stepwise screening strategy. We first evaluated single substitutions within these prioritized regions, with particular emphasis on hydrophobic cavity-filling or cavity-reducing substitutions in the prefusion head/apex. Supernatant ELISA screening identified favorable single substitutions across multiple prioritized segments, including three conformationally divergent regions and one adjacent supporting region. These candidates were then advanced to combinatorial screening, and additional stabilization classes were subsequently introduced and evaluated on the basis of the better-performing hydrophobic substitution combinations. These screening is summarized in Appendix Table S2. Within this framework, multiple established stabilization mechanisms were evaluated using the same functional screening system, including readouts related to prefusion-specific antigenicity, detectable F antigen output, short-term stress tolerance, and subsequent purified-protein behavior. Importantly, this stepwise filtering process did not converge on an extensively covalent-locked or highly mutation-loaded construct. Instead, the final design represented a comparatively low-mutation solution combining apex-centered cavity-reducing hydrophobic substitutions with D486-centered electrostatic tuning. In this sense, the key methodological innovation of the study lies in how static structural comparison was translated into a region-prioritized stabilization rationale, and how established stabilization classes were experimentally filtered within that rationale.

Importantly, disulfide- and proline-based variants were not ignored. Rather, they were included in the same comparative strategy together with cavity-filling and electrostatic designs. Although some of these variants improved individual readouts, they were not prioritized as the basis of the final construct because, under the multi-parameter criteria used here, they provided a less favorable overall balance of stability gain, purified-protein behavior, mutation burden, and antigenic preservation than the final low-mutation design.

In the revised manuscript, we will (i) markedly abbreviate the general structural description in the Results section, (ii) move more of the established design background to the Introduction where appropriate, and (iii) add a clearer parallel discussion of how our optimization strategy differs from previously reported RSV pre-F stabilization approaches. Our goal in revision is to make it clearer that the key innovation of this work lies in a structure-partitioned, region-prioritized, experimentally filtered optimization strategy, rather than in any single mutation category alone.

Changes in the manuscript:

1. **Introduction:** We revised the introduction to better position the present study relative to previously reported RSV pre-F stabilization strategies and to clarify the rationale of the design framework.
   Page 5, Line 97–106.
   "In broad terms, previously reported RSV preF designs have relied on one or more established stabilization principles, including covalent locking, local rigidification, cavity filling, and electrostatic optimization (McLellan et al, 2013a; Krarup et al, 2015; Huang et al, 2025). Here, we used comparative structural analysis to establish a structure-guided optimization strategy for RSV F, in which selected

regions were prioritized for focused mutational evaluation across multiple stabilization mechanisms. Using this strategy, we identified a lead prefusion-stabilized variant with improved prefusion antigenicity, favorable expression, and enhanced resilience to thermal and storage stress, supporting a practical low-mutation design for bivalent vaccine development."

2. **Results section:** We substantially shortened the description of general prefusion/postfusion structural differences and refocused the section on the design logic derived from structural comparisons.
   Page 6, Line 108–109.
   "Structure-guided regional prioritization and focused mutational design of prefusion RSV-F antigens"
   Page 6, Line 117–124.
   "Based on structural superposition by secondary-structure matching (SSM), residues 26–57, 227–305, and 306-459 were classified as similar between the prefusion and postfusion conformations, while residues 58–72, 73–96, 147–226, and 460-–513 showed substantial conformational divergence and were classified as different (Appendix Table S1). Rather than treating these differences as descriptive structural features alone, we used this organization to prioritize conformationally divergent regions, together with adjacent supporting framework, for focused mutational design."
3. **Results section:** We clarified that multiple established stabilization classes were evaluated within a unified structural partitioning and functional screening framework.
   Page 6, Line 125–132.
   "Using this region-prioritized strategy, we designed variants spanning four established stabilization classes commonly applied to class I viral fusion glycoproteins: engineered disulfide bonds, proline substitutions, hydrophobic cavity filling, and electrostatic optimization at the trimer interface. In particular, regions exhibiting substantial conformational divergence between the prefusion and postfusion states (residues 58–226 and 460–513)were prioritized, while neighboring structural elements predicted to provide conformational support, particularly the apex-adjacent 227–305 region, were also considered."
4. **Supplementary Information:** We added Appendix Table S1 to summarize the region-based single-substitution screening strategy.
   "Appendix Table S1. SSM-based structural alignment statistics for seven segments of RSV F comparing the prefusion (PDB: 4JHW) and postfusion (PDB: 3RRR) conformations."
5. **Discussion section:** We revised the Discussion to better emphasize the structural basis underlying mutation prioritization and the design rationale of the final construct.
   Page 33, Line 554–558.
   "In RVF-88, multiple established stabilization classes were evaluated within the same optimization framework, and the final construct converged on a comparatively low-mutation combination of apex-centered cavity-reducing hydrophobic substitutions and D486-centered electrostatic tuning."
   Page 33, Line 566–569.
   "In this context, RVF-88 provides an alternative prefusion-stabilization strategy that preserves key antigenic and structural features in the assays performed here without introducing exogenous cysteines."

2. The term "High-throughput screening" is inaccurate. From the authors' screening strategy, if my current understanding is correct, it mainly involves preliminary characterization of the expression level and conformation of more than 100 single-point mutations based on ELISA, and screening of combinations of individual prominent single-point mutations. Overall, this strategy is only labor-intensive but not high-throughput, and its bottleneck is the efficiency of ELISA detection. This strategy can neither traverse all combinatorial mutations nor reasonably exclude a large number of invalid mutations in the huge combinatorial mutation space based on ideas such as selecting Pareto optimality. It is recommended that the authors no longer emphasize high-throughput.

Response:

We thank the reviewer for pointing this out. We agree that the term "high-throughput screening" is not the most accurate description of the screening framework used in this study and may overstate the scale and nature of the approach. Our intention was to emphasize that the screening process was systematic and conducted within a structure-guided design framework, rather than to imply exhaustive or truly high-throughput traversal of the combinatorial mutation space. We have therefore removed the term "high-throughput screening" from the manuscript and replaced it with more accurate terminology.

Changes in the manuscript:

1. **Introduction:** We removed the inaccurate use of "high-throughput screening" to better reflect the nature of the experimental workflow.
   Page 5, Line 103–106.
   "Using this strategy, we identified a lead prefusion-stabilized variant with improved prefusion antigenicity, favorable expression, and enhanced resilience to thermal and storage stress, supporting a practical low-mutation design for bivalent vaccine development."
2. **Results section:** We revised the section title to more accurately describe the screening strategy and downstream optimization process.
   Page 11, Line 184–185.
   "Systematic screening and combinatorial optimization prioritized RVF-88A as a candidate design"

3. Regarding the process by which the authors obtained the final four-mutation (N67L-V90Y-S190I-M289F) or five-mutation (N67L-V90Y-S190I-M289F-D486S) combination, there is still a high degree of randomness from the results currently presented. The authors' arguments on the sufficiency and necessity of these mutations being included in the final construct are insufficient; the discussion on the inevitability of obtaining this combinatorial mutation through the screening strategy they adopted is also insufficient. Among these, S190I and D486S have been reported in previous studies. In Fig. 2A of this manuscript and related supplementary figures, the contribution of D486S to the proportion of pre-fusion conformation is not prominent, while S190I is not in the authors' initial mutation set at all. The appearance of these two mutations in the final construct lacks sufficient rationality. In Figure 2C, the data presented by the authors show that the four-mutation construct has a great advantage over the three-mutation construct N67L-V90Y-M289F, but no results of the S190I single mutation are given. Does this indicate that the S190I mutation has the greatest effect on the stability of the pre-fusion conformation among these four single-point mutations? I suggest the authors to re-organize this part in order to highlight the necessity of their selected mutation.

Response:

We thank the reviewer for this important comment. We agree that the current presentation of the four-mutation construct N67L-V90Y-S190I-M289F and the five-mutation construct N67L-V90Y-S190I-M289F-D486S does not yet make the selection logic sufficiently transparent, and may therefore give an impression of randomness. We would like to clarify that our intention was not to imply that the final mutation set was uniquely determined or inevitable. Rather, RVF-88 was selected through a stepwise optimization process within the design space tested in this study, integrating systematic single-site screening, literature-guided inclusion of additional substitutions, and subsequent combinatorial validation.

For S190I, we agree that the rationale for its appearance in the final construct should be explained more clearly. This substitution was not part of the initial 126-member single-site hydrophobic cavity panel. Instead, it was introduced in a literature-guided manner based on prior prefusion-stabilizing designs, including S190F in DS-Cav1 and S190I in construct 847 (Che et al, 2023). We then evaluated it in combinatorial designs. In this context, as shown in Fig. 2C, the four-mutation construct N67L-V90Y-S190I-M289F showed a clearer advantage over the corresponding three-mutation construct N67L-V90Y-M289F, most notably in retention of D25 reactivity after 55 °C for 1 h. At the same time,

because an S190I single-mutation result is not shown, the current dataset does not allow us to rank the relative contribution of S190I against the other selected substitutions on a single-site basis. We will therefore revise the text to clarify that S190I was incorporated based on prior design knowledge and supported in the combinatorial context tested.

For D486S, we agree that its single-site effect in Fig. 2A is not as visually prominent as that of some cavity-filling substitutions, and that the manuscript should better distinguish between its single-site behavior and its role in the final combinatorial design. In our screening framework, D486S emerged as a favorable candidate within the electrostatic class, showing strong D25 reactivity together with stability upon storage. More importantly, when evaluated on the four-mutation core background, its addition was associated with improved overall stability in the purified-protein setting, particularly in SEC homogeneity under the RT-7D condition, together with favorable thermal stability behavior (Appendix Fig. S4). Thus, the rationale for prioritizing the five-mutation design was not that D486S was the strongest single-site hit, but that it improved the overall performance of the optimized core construct in the combinatorial setting tested. In addition, we further examined alternative substitutions at positions 67, 90, 190, 289, and 486 on both WT and RVF-88 backgrounds, which supported that the residues retained in the final construct generally ranked among the better-performing candidates tested in terms of prefusion antigenicity, expression level, and conformational stability (Appendix Fig. S5).

In response to the reviewer's suggestion, we will reorganize this section to make the selection process clearer by distinguishing three layers more explicitly:
(i) substitutions emerging from the systematic single-site screen;
(ii) S190I as a literature-guided inclusion subsequently supported in combinatorial testing; and
(iii) D486S as a mutation prioritized based on its added benefit in the four-mutation core background.
We will also revise the wording throughout this section to avoid any implication that the final five-mutation construct represents the only or inevitable solution, and instead describe RVF-88 more accurately as the construct prioritized for further validation within the tested framework.

Changes in the manuscript:

1. **Results section:** We clarified that S190I was introduced based on prior prefusion-stabilizing designs reported in the literature and then evaluated in combinatorial screening.
   Page 12, Line 226–231.
   "In addition, S190I was incorporated based on prior reports showing that cavity-filling substitutions at position 190 can confer prefusion stabilization, including S190F in DS-Cav1 and S190I in construct pXCS847 (McLellan et al, 2013a; Che et al, 2023); its favorable effect on thermal stability was subsequently supported in combinatorial screening (Fig. 2C)."
2. **Results section:** We revised the wording regarding D486S to distinguish its single-site behavior from its contribution within the tested combinations on the cavity-filling core background.
   Page 13, Line 248–250.
   "Importantly, within the combinations tested on the cavity-filling core background, addition of D486S was associated with improved stability, particularly in purified-protein analyses under storage stress."
3. **Results section:** We clarified that the selected substitutions consistently ranked among the better-performing alternatives evaluated at their respective positions.
   Page 14, Line 253–257.
   "Introducing single-site substitutions at positions 67, 90, 190, 289 and 486 on both the WT and RVF-88 backbones further supported that the selected residues at their respective positions consistently ranked among the better-performing alternatives tested in terms of prefusion antigenicity, expression level, and conformational stability under thermal and storage stress (Appendix Fig. S5)."

4. **Discussion section:** We clarified the current limitations regarding the contribution of S190I and acknowledged the need for further targeted validation.
   Page 35, Line 599–601.
   "The relative contribution of S190I at the single-mutation level was not directly resolved in the present study and could be further clarified by future back-mutation or targeted mutational analyses."

Reference:

Che Y, Gribenko AV, Song X, Handke LD, Efferen KS, Tompkins K, Kodali S, Nunez L, Prasad AK, Phelan LM, et al (2023) Rational design of a highly immunogenic prefusion-stabilized F glycoprotein antigen for a respiratory syncytial virus vaccine. Science Translational Medicine 15: eade6422

4. The data in Fig. 3B show that L88B has much weaker binding activity to D25 than L88A (by ELISA), but there is no significant difference in the KD values based on SPR. Similarly, L88A has lower affinity for palivizumab, but shows similar affinity to its humanized version motavizumab as L88B. It is generally believed that D25 is not an RSV A-specific antibody. The authors are suggested to explain this inconsistency from the perspective of experimental design and rule out that it is caused by experimental errors.

Response:

We thank the reviewer for highlighting this point. We agree that D25 is not an RSV A-specific antibody, and we did not intend to imply subtype-specific recognition by D25. The lower D25 ELISA signal observed for RVF-88B should be interpreted in the context of the assay format, rather than as a direct quantitative measure of intrinsic affinity.

ELISA and SPR provide complementary but non-equivalent binding readouts. ELISA signals can be affected by antigen immobilization, coating efficiency, orientation, and local epitope exposure, whereas SPR provides kinetic binding parameters under a defined biosensor format.Therefore, differences in ELISA signal intensity do not necessarily translate into proportional differences in SPR-derived KD values. Consistent with this interpretation, assay-dependent differences between ELISA and SPR-based immunoassays have been reported previously (Beeg et al, 2021).

This interpretation is also consistent with prior observations for RSV site Ø antibodies. For example, Tian et al. reported subtype-dependent differences in D25 neutralization potency, while SPR still showed broadly comparable high-affinity binding to RSV A2 and RSV B9320 prefusion F proteins (< 76.9 pM vs < 144 pM). Thus, the weaker D25 ELISA signal for RVF-88B does not indicate loss of D25 recognition, consistent with the comparable SPR KD values.

Regarding palivizumab and motavizumab, we have clarified that these assays were used primarily to assess retention of site II antigenicity rather than to overinterpret small quantitative differences in affinity. Motavizumab is an affinity-matured derivative of palivizumab with enhanced binding activity (Wu et al, 2007). In our measurements, the KD values were within the nanomolar to sub-nanomolar range; therefore, the minor differences observed between RVF-88A and RVF-88B were interpreted qualitatively as preserved site II antigenicity rather than as meaningful subtype-specific differences.

Reference:

Beeg M, Burti C, Allocati E, Ciafardini C, Banzi R, Nobili A, Caprioli F, Garattini S & Gobbi M (2021) Surface plasmon resonance unveils important pitfalls of enzyme-linked immunoassay for the detection of anti-infliximab antibodies in patients' sera. Sci Rep 11: 14976

Tian D, Battles MB, Moin SM, Chen M, Modjarrad K, Kumar A, Kanekiyo M, Graepel KW, Taher NM, Hotard AL, et al (2017) Structural basis of respiratory syncytial virus subtype-dependent neutralization by an antibody targeting the fusion glycoprotein. Nat Commun 8: 1877

Wu H, Pfarr DS, Johnson S, Brewah YA, Woods RM, Patel NK, White WI, Young JF & Kiener PA (2007) Development of Motavizumab, an Ultra-potent Antibody for the Prevention of Respiratory Syncytial Virus Infection in the Upper and Lower Respiratory Tract. Journal of Molecular Biology 368: 652–665

5 Fig. 5B shows that the error bars of the L88 and ABRYSVO groups on Day 35 are large. Can the authors display each individual data point on the basis of the current graph?

Response:

We thank the reviewer for this helpful suggestion. We agree that displaying the individual data points would allow a clearer visualization of the data dispersion and within-group variability at Day 35. Accordingly, we have added the individual data points to Fig. 5B and 5C to improve the transparency of data presentation.

Changes in the manuscript:

1. **Figure 5B and 5C:** We added the individual data points to Fig. 5B and 5C and updated the corresponding figure legend accordingly.
   Page 27, Line 450.

6. For Figure 5G-J, a sham group must be included for the RNA copies assay. If such data are not available, it is recommended to remove all the RNA copy number determination results in this figure. Since cotton rats infected with RSV do not show significant weight loss, it is also recommended to remove Figs. 5E-F and the content related to weight changes in the main text. When comparing whether there are significant differences in other parts of Fig. 5 in the main text, the authors should specify whether it is RNA copies or FFU, because in many groups, the results of these two detection methods are different.

Response:

We thank the reviewer for this important and constructive comment. To address the concern regarding the lack of a sham control for the RNA copy number assay, we supplemented a small cohort of unimmunized, unchallenged cotton rats as a sham reference group. This sham group has now been included in Fig. 5G–J to provide a baseline for interpreting RNA copy number and infectious viral titer measurements, with the limit of detection (LOD) indicated by dashed lines. Because the sham cohort was not processed in the same experimental batch as the original challenged animals, it is presented as a baseline reference rather than as a batch-matched comparator.

Following the reviewer's recommendation, we removed the cotton rat body weight data and the associated text from the main manuscript, because body weight change is not a sensitive pathological readout in the cotton rat RSV infection model. We also revised the Results section and figure legend to explicitly distinguish RNA copy number from infectious viral titer, reported as FFU, and to specify which readout was used when describing significant differences.

Changes in the manuscript:

1. **Figure 5E-H and legends (Formerly Figure 5G-J):** We added a sham reference group and updated viral readouts for clearer interpretation, with corresponding sham data points and graphical indicators

included in the figure panels.
Page 27, Line 450.

2. **Figure 5E-F and corresponding descriptions in the Results section:** We removed cotton rat body weight data (Fig. 5E-F) and the corresponding descriptions in the Results section were removed.
Page 27, Line 450.
3. **Results section:** We clarified and expanded the description of viral RNA copy number and infectious viral titers.
Page 25, Line 432–439.
"Viral burden analyses in cotton rats showed that RVF-88 significantly reduced both lung viral RNA loads and lung viral titers after A2 and B9320 challenge relative to the buffer control. In contrast, ABRYSVO significantly reduced both endpoints after A2 challenge. After B9320 challenge, ABRYSVO significantly reduced lung viral titers but not lung viral RNA loads relative to the buffer control (Fig. 5E, F). In nasal turbinates, RVF-88 significantly reduced both viral load and viral titer compared with controls after A2 challenge (Fig. 5G), whereas neither RVF-88 nor ABRYSVO significantly reduced nasal viral burden after B9320 challenge (Fig. 5H)."

7. Regarding the structural characterization part, I suggest that on the basis of the existing structures, the authors could provide separate crystal or cryo-EM structures of the pre-fusion trimeric forms of L88A/B without any antibodies, even. Even a low resolution structure will help. It is also preferable to provide the determination of the proportion of pre- and post-fusion conformations of the construct sample in solution if available(such as negative staining EM). The reasons are as follows: First, D25 helps stabilize the pre-fusion conformation. Even the WT RSV-F protein can obtain stable pre-fusion conformation crystals when co-crystallized with D25, which makes the EM structure of the antibody-containing complex unable to be used to judge whether L88A/B spontaneously forms the pre-fusion conformation implied by the authors. I understand that the authors performed circular dichroism detection in Fig. S5 and implied that its helix ratio is highly consistent with the pre-fusion conformation, but the antibody complex structure does not provide additional information on this basis. On the other hand, D25 and other site-O antibodies help stabilize the local conformation of the antigen, and in fact, RSV-F has an alternative conformation near site O. Since the authors attempt to discuss the differences in specific side chain positions at near-atomic resolution based on the structure, the authors need to ensure that this change is not an artifact caused by antibody binding. Even if the resolution of L88A/B without antibody binding is not sufficient for accurate side chain modeling, this structure should be provided for comparison. If there is no apo structure of the trimer, it is recommended to rewrite the structural analysis of the D25 complexed structure and point out in main text that there is still possibility the pre-fusion structure is maintained in part by the D25 antibody. It is also suggested to pay special caution on the side-chain analysis and the analysis to claim that the L88 is a more "compact" construct solely on the current available structure.

Response:

We thank the reviewer for this important and constructive suggestion. We agree that D25 and other site Ø antibodies can stabilize the prefusion conformation of RSV F, and therefore D25-bound structures alone should not be used as definitive evidence that RVF-88A/B spontaneously adopts the prefusion trimeric conformation in solution.

To address this concern, we have revised the structural section to more clearly distinguish evidence derived from antibody-free structures from that derived from D25-bound cryo-EM structures. Specifically, antibody-free crystal structures of RVF-88A and RVF-88B were included in the revised Appendix Fig. S15. These structures showed trimeric prefusion-like architectures in the absence of D25. In the revised manuscript, these antibody-free structures are used to support the overall prefusion-like trimeric architecture of RVF-88A/B, whereas the D25-bound cryo-EM structures are used primarily to provide high-resolution

structural context for the engineered substitutions and to assess compatibility of the site Ø epitope with D25 recognition.

We agree that direct quantification of the prefusion and postfusion populations in solution, for example by negative-stain EM, would provide additional useful information. Such analysis was not available in the present study, and we have therefore avoided making quantitative claims regarding the relative proportions of prefusion and postfusion particles in solution.

We also agree that the previous use of the term "compact" was too broad and could imply a global structural conclusion that was not sufficiently supported. We have therefore removed this term from the title, abstract, and discussion, and replaced it with more specific structure-based descriptions. In Appendix Fig. S15 and Appendix Table. S5, cavity analysis based on the trimeric prefusion apex of antibody-free RVF-88A structures showed reduced apical hydrophobic cavity volume and surface area compared with the reference prefusion F structure (PDB: 4JHW) and DS-Cav1 (PDB: 4MMU). This analysis is now presented as supportive evidence for the intended apical cavity-reduction design, rather than as direct proof of global structural compaction or enhanced thermodynamic stability.

In addition, side-chain-level interpretations near site Ø have been revised and toned down. Statements implying definitive local packing, reduced conformational breathing, or direct stabilization based solely on the D25-bound structures were replaced with more cautious wording. We also added text to the Discussion to acknowledge that cavity reduction does not by itself constitute direct thermodynamic evidence of enhanced stability, but provides structural support for the rationale that reducing unfavorable apical voids may contribute to local structural support of the prefusion apex.

Changes in the manuscript:

1. **Appendix Figure S15 and corresponding structural characterization section:** We added antibody-free crystal structures of RVF-88A and RVF-88B and revised the structural analysis to distinguish antibody-free structural evidence from D25-bound cryo-EM evidence.
   Page 29, Line 484–491.
   "To complement the D25-bound cryo-EM analysis, antibody-free crystal structures of RVF-88A and RVF-88B were also analyzed and showed trimeric prefusion-like architectures consistent with the D25-bound cryo-EM structures (Appendix Fig. S15A and S15B). Local cavity analysis of the trimeric prefusion apex showed that RVF-88A had lower total cavity volume and surface area than selected prefusion F reference structures within the same local search region and under identical detection parameters, consistent with the intended cavity-reduction design at the prefusion apex (Appendix Fig. S15C, S15D and Appendix Table. S5)."
2. **Results section:** We revised the description and interpretation of the D25-bound cryo-EM structures to clarify that the RVF-88-D25 structure was used to assess compatibility with D25 recognition and preserved epitope presentation.
   Page 29, Line 494–502.
   "Residue 67 is located in the apex/apex-proximal region of prefusion F, a region previously implicated in preF conformational stability. In RVF-88, N67 was replaced with leucine (N67L), which is oriented inward and is positioned near residues in the α1 region, whereas the corresponding N67 in DS-Cav1 is observed in a different local environment near the α4 region (Appendix Fig. S15E). Notably, residue 67 does not directly contact the D25 epitope, as its side chain is oriented inward; accordingly, the mutation had minimal impact on antibody recognition, consistent with preserved epitope presentation in the RVF-88-D25 structure."

3. **Appendix Figure S15 and Appendix Table S5:** We added cavity analysis based on the antibody-free RVF-88A crystal structure, including comparisons of apical hydrophobic cavity volume and surface area with reference prefusion F structures (PDB: 4JHW and 4MMU).
4. **Title and Abstract:** We removed the term “compact” and replaced it with more specific descriptions.
   Title, Page 1, Line 1–3.
   “Structure-guided rational design of a prefusion RSV F bivalent vaccine with enhanced immunogenicity”
   Abstract, Page 2, Line 40–43.
   “Structural analyses showed that RVF-88 preserves the site Ø epitope and exhibits reduced apical hydrophobic cavity volume and surface area consistent with the intended cavity-reduction design.”
5. **Discussion section:** We added a statement clarifying that cavity reduction provides supportive structural evidence for the design rationale.
   Page 35, Line 601–604.
   “These substitutions support a molecular rationale of apex-centered packing reinforcement by reducing the apical hydrophobic cavity volume and are consistent with the intended cavity-filling design, which may contribute to local stabilization of the prefusion apex.”

8. The figure legend of Fig. 6 shows five panels (A-E), but there are only four panels (A-D) in the actual figure. Please the authors explain the correspondence.

Response:

We thank the reviewer for pointing this out. Fig. 6 is intended to contain five panels (A–E), as stated in the figure legend. Panel E shows the comparison of Phe289 in RVF-88A/B with Met289 in pXCS847A. We apologize for the confusion caused by the submitted version, in which this panel may not have been clearly visible or fully displayed. We have ensured that all five panels (A–E) are clearly presented in the revised figure file and correspond fully to the legend.

Changes in the manuscript:

1. **Figure 6:** We have revised the figure file to ensure that all five panels (A–E) are clearly visible and consistent with the figure legend.
   Page 31, Line 524.

//Minor Suggestions//

1. Line 64. The term "inherently unstable" cannot be used to describe the pre-fusion conformation; the correct term for this conformation is "metastable", which indicates that it is still stable. "Inherently unstable" tends to be used to describe a thermodynamically transient unstable state.

Response:

We sincerely thank the reviewer for this precise and important correction. We agree that “metastable” is the scientifically accurate term to describe the prefusion conformation of RSV F, as it correctly identifies the protein’s state in a local energetic minimum before transitioning to the thermodynamically more stable postfusion state. We have revised the sentence to incorporate this terminology.

Changes in the manuscript:

1. **Introduction section:** We replaced the term “inherently unstable” with “metastable” to more accurately describe the prefusion conformation of RSV F.
   Page 4, Line 63–66.
   “While most potent neutralizing antibodies recognize epitopes unique to the pre-F conformation, this state is metastable and readily undergoes irreversible rearrangement into the post-F form (McLellan et al, 2013b; Ngwuta et al, 2015).”

2. Line 67. The main reason for selecting pre-fusion is not to avoid ERD, but to display pre-fusion-specific epitopes, and these antibody epitopes happen to have high neutralizing activity. The causes of ERD are relatively complex, and non-neutralizing antibodies are only one of the speculated possible related factors, and engineering pre-fusion-specific immunogens is not to eliminate ERD. It is recommended to delete the expressions related to ERD.

Response:

We sincerely thank the reviewer for this critical and insightful comment. We agree that the primary rationale for stabilizing RSV F in its prefusion conformation is to preserve and present prefusion-specific epitopes (such as Site Ø and Site V) that elicit potent neutralizing antibody responses.

We also acknowledge that the mechanisms underlying Vaccine-Associated Enhanced Respiratory Disease (ERD) are multifactorial and complex, and that engineering prefusion-specific immunogens is not a direct or guaranteed means of eliminating ERD risk. To ensure scientific rigor and avoid oversimplification of these mechanisms, we have removed all expressions linking prefusion stabilization to the mitigation of ERD in the Introduction and Discussion.

Changes in the manuscript:

1. **Introduction sections:** We removed statements linking prefusion stabilization with mitigation of ERD and revised the text to emphasize preservation of prefusion-specific neutralizing epitopes as the primary rationale for prefusion antigen design.
   Page 4, Line 66–70.
   “Stabilizing the F protein in its prefusion conformation preserves key prefusion-specific epitopes associated with potent neutralizing antibody responses. Because these epitopes are critical for eliciting high-titer protective immunity, prefusion stabilization has emerged as a central strategic objective in RSV vaccine development.”

3. Line 72. Although RSV has two genotypes (A/B), they belong to the same serotype. Current studies have fully proved that an immunogen of one genotype can produce cross-neutralization and protection against genotypes A/B under the action of strong adjuvants. Therefore, bivalent design is not necessary in RSV vaccine design. It is recommended to revise this part.

Response:

We thank the reviewer for this important comment. We agree that RSV A and B belong to the same serotype, and monovalent RSV F immunogens can induce cross-neutralizing responses against both subgroups. However, emerging evidence suggests potential advantages for bivalent approaches.

Recent studies demonstrate that certain potent neutralizing antibodies show subgroup-preferential activity. For example, antibody 5C4 potently neutralizes RSV A strains but has significantly reduced neutralization activity against RSV B strains due to sequence differences at position 201 in antigenic site Ø (Rossey et al., Nat Commun 2017). Additionally, recent clinical evidence indicates that "initial results from the second

season after vaccination suggest specific RSV B efficacy wanes more rapidly than RSV A for RSV PreF-based monovalent vaccines" PubMedPubMed Central (Moyersoen et al., Infect Dis Ther 2024). This differential waning supports the rationale for bivalent formulations to ensure sustained protection against both subgroups. We have therefore revised the text to avoid overstating the necessity of bivalent design and to more accurately reflect this rationale.

Changes in the manuscript:

1. **Introduction sections:** We revised the description of RSV bivalent vaccine design to clarify that RSV A and B belong to the same serotype while noting that subgroup-specific antigenic differences may still support balanced bivalent protection strategies.
   Page 4, Line 73–80.
   "Although the RSV F protein is highly conserved (>90% sequence identity) between the two subgroups (McLellan et al, 2013c; Oraby et al, 2025) and RSV F-based immunogens can induce cross-neutralizing responses, sequence differences remain concentrated in key prefusion epitopes, particularly site Ø, which can influence subgroup-specific antigenic recognition (Oraby et al, 2025; Hashimoto & Hosoya, 2017). Therefore, despite RSV A and B generally being considered to belong to the same serotype, a bivalent design may still be advantageous for achieving more balanced protection against both subgroups."

4. Lines 84-85. The term "multi-proline" is not mentioned in the literature cited by the authors. The multi-proline design scheme is more famous in the design of HIV and SARS-CoV-2 antigens (S2P or S6P). Please revise the corresponding expressions or add the corresponding reference literature.

Response:

We thank the reviewer for this helpful comment. We agree that the term "multi-proline" was not the most appropriate wording in this context and may be confusing, as it is more commonly associated with stabilization schemes described for other viral antigens. We have therefore revised the text to use terminology that is more consistent with the RSV literature cited here, replacing "multi-proline" with "multiple proline substitutions (e.g., preF7P)", consistent with the terminology used in the cited reference (Huang et al, 2025).

Changes in the manuscript:

1. **Introduction sections:** We replaced the term "multi-proline" with "multiple proline substitutions (e.g., preF7P)" to align the terminology with the cited RSV literature.
   Page 5, Line 86–91.
   "Subsequent efforts have yielded rationally stabilized variants, such as uncleaved prefusion-closed (UFC) trimers that maintain the closed preF without disulfide crosslinks, tyrosine-crosslinked forms with extended stability, and variants incorporating multiple proline substitutions (e.g., preF7P) that achieve high expression and robust immunogenicity (Lee et al, 2024; Gidwani et al, 2024; Li et al, 2025; Huang et al, 2025)."

5. Line 91. The authors intend to demonstrate how to achieve pre-fusion conformation design with the minimal mutations in this manuscript, but the five-mutation design mentioned in this manuscript is not the one with the least number of mutations to achieve this goal; see, for example: PMID: 39325881, etc. In addition, the authors have not proved that these five mutations are the minimal set of essential mutated amino acids, that is, each single mutation is indispensable. Therefore, it is recommended that the authors remove this expression.

Response:

We thank the reviewer for this critical assessment and for providing the relevant references, including PMID: 39325881. We agree that the term “minimal” was imprecise. Our design, RVF-88, does not aim to achieve the absolute minimum number of mutations across the field, but rather follows a structure-guided mutagenesis strategy to achieve a balance between conformational stability and immunogenicity.

This five-mutation combination was selected to stabilize the prefusion state while preserving the spatial integrity of neutralizing epitopes. We have removed the term “minimal” and revised the manuscript to state the objectives of our mutational strategy more objectively.

Changes in the manuscript:

1. **Introduction sections:** We removed the term “minimal” and revised the description of the mutagenesis strategy to emphasize a focused structure-guided design aimed at balancing prefusion stabilization and epitope preservation.
   Page 5, Line 100–106.
   “Here, we used comparative structural analysis to establish a structure-guided optimization strategy for RSV F, in which selected regions were prioritized for focused mutational evaluation across multiple stabilization mechanisms. Using this strategy, we identified a lead prefusion-stabilized variant with improved prefusion antigenicity, favorable expression, and enhanced resilience to thermal and storage stress, supporting a practical low-mutation design for bivalent vaccine development.”

6. Lines 128-129: The description "targeted residues within regions that undergo substantial conformational rearrangement (residues 58-226 and 460-513)" is not rigorous. For example, sites such as M289 and I292 are not within this range, although they play a role in stabilizing the prefusion conformation by stabilizing the pre-F protein in the above range. It is recommended that the authors make a more rigorous description.

Response:

We agree that the original description was imprecise. Our design strategy prioritizes the stabilization of regions showing the greatest structural divergence (residues 58–226 and 460–513). Since the stability of these segments depends on their interactions with the surrounding structural framework, our mutagenesis targets both residues within these regions and those in the adjacent environment (such as M289 and I292) that contribute to the stability of the prefusion conformation. The text has been revised to reflect this logic.

Changes in the manuscript:

1. **Results sections:** We revised the description of the mutagenesis framework to clarify that the design targeted both highly rearranged regions and adjacent structural elements contributing to prefusion conformational support.
   Page 7, Line 128–134.
   “In particular, regions exhibiting substantial conformational divergence between the prefusion and postfusion states (residues 58-226 and 460-513)were prioritized, while neighboring structural elements predicted to provide conformational support, particularly the apex-adjacent 227-305 region, were also considered. Accordingly, we engineered 16 intra-protomer disulfide pairs to impose covalent constraints and stabilize the prefusion trimer (Appendix Fig. S1A).”

7. Lines 134-139: Only 16 of the 23 proline-scanning mutations described by the authors are given in Figure S1. Please supplement the remaining seven sites. Whether there are combinatorial mutations (such as the T267P-N268P

combinatorial mutation mentioned in Line 194) should also be correctly described. Were the 20 sites of cavity-filling mutations each mutated to seven amino acids (Met, Leu, Ile, Phe, Tyr, His, Trp) or only to a subset of them? Please make a clearer description in the supplementary materials.

Response:

We thank the reviewer for this helpful comment. In the revised manuscript, we have clarified the full composition of the screening panel as follows:

(1) Of the 23 proline-scanning designs, 16 are single-site substitutions and 7 are double-proline combinatorial substitutions at adjacent positions (D73P-A74P, I148P-A149P, E161P-G162P, S173P-T174P, I217P-E218P, T267P-N268P, and I492P-S493P). The original Appendix Fig. S1 displayed only the 16 single-site variants, which we recognize was a source of confusion.

(2) For the cavity-filling mutations, each of the 20 targeted sites was screened against all seven bulkier hydrophobic residues (Met, Leu, Ile, Phe, Tyr, His, and Trp), with minor exceptions.

These details have now been incorporated into both the main text and the legend of Appendix Fig. S1B–C.

Changes in the manuscript:

1. **Results sections:** We clarified the composition of the screening panel by specifying the 16 single-site and 7 double-proline substitution designs and describing the hydrophobic cavity-filling substitution strategy tested at each targeted site.
   Page 7, Line 134–142.
   "For proline substitutions, we targeted to hinge- or turn-like positions to reduce local flexibility; in total, 23 such substitutions were designed, comprising 16 single-site proline substitutions and 7 double-proline combinatorial substitutions at adjacent positions (Appendix Fig. S1B). For hydrophobic cavity filling on preF, cavities were classified as surface-exposed cavities and interprotomer interface voids to reinforce hydrophobic core packing, increase expression yield, and enhance conformational stability, native residues were replaced with higher-volume hydrophobic side chains (Met, Leu, Ile, Phe, Tyr, His, Trp), with each of the 20 targeted sites screened against all seven substitutions (Appendix Fig. S1C)."
2. **Appendix Figure S1 legend:** We revised the legend to specify the composition of the proline-substitution screening panel, including the 16 single-site and 7 double-proline combinatorial designs, and to describe the hydrophobic cavity–filling mutation categories and substitution screening strategy used for the 20 targeted sites.
   "(B) Positions of proline substitutions, displayed as pink spheres with a fluorescent green outline. A total of 23 proline-substitution designs were generated, comprising 16 single-site substitutions and 7 double-proline combinatorial substitutions at adjacent positions: D73P-A74P, I148P-A149P, E161P-G162P, S173P-T174P, I217P-E218P, T267P-N268P, and I492P-S493P. Only the 16 single-site substitutions are displayed individually; double-proline pairs are indicated by their proximal positions on the structure. (C) Positions of hydrophobic cavity–filling mutations. Mutated residues are shown as red spheres with a fluorescent green outline. The substitutions were designed to fill three types of hydrophobic cavities: (1) surface-exposed cavities, (2) internal cavities in the upper head region, and (3) cavities at interprotomer interfaces with other chain (blue surface). For each of the 20 targeted sites, substitutions to bulkier hydrophobic residues (Met, Leu, Ile, Phe, Tyr, His, and Trp) were screened; all seven amino acids were tested at each position with minor exceptions."

8. Excessive colors are used in Fig. 1A and are difficult to distinguish. It is recommended to use the same color for the post-fusion structures of all motifs to display them superimposed with the pre-fusion structures to produce sufficient distinguishability. In addition, the authors should clearly indicate what the criteria for distinguishing "similar" and "different" are? Why are RMSD=0.97 and 0.92 defined as "different", but RMSD=1.47 is instead defined as "similar"? In addition, the RMSD value is missing for the aa. 137-226 part.

Response:

We thank the reviewer for this comment, which addresses both the color scheme of Fig. 1A and the structural classification criteria. We address each point below.

Color scheme of Fig. 1A. We acknowledge that the large number of colors may appear visually complex. The color scheme was intentionally designed to assign a distinct color pair to each of the seven structural segments, with the prefusion conformation in a deeper shade and the postfusion conformation in a lighter shade of the same color. As the figure includes panel-level close-up views highlighting individual segments separately, we believe the overall presentation remains interpretable. Nevertheless, we have revised the figure legend to more explicitly guide the reader through the color coding and ensured that the color key is clearly stated at the beginning of the legend.

Classification criteria for "similar" vs. "different." We agree that the original manuscript did not explicitly define these criteria. Segments were classified as "similar" only when all four of the following conditions were simultaneously satisfied: (1) SSM superposition succeeded; (2) coverage ≥ 0.90; (3) backbone RMSD ≤ 1.50 Å; and (4) SSM quality factor Q ≥ 0.02. A segment failing any one criterion was classified as "different."

Apparent inconsistency between RMSD values and classifications. The classification was not based on RMSD alone. Segment 306–459 (RMSD = 1.47 Å) was classified as "similar" because it satisfied all four criteria (coverage = 0.90, Q = 0.097). In contrast, segment 73–96 (RMSD = 0.97 Å) was classified as "different" because its coverage fell below 0.90 and its Q value was reduced, reflecting noticeable changes in secondary-structure arrangement. Similarly, segment 460–513 (RMSD = 0.92 Å) was classified as "different" because only 23 of 54 residues could be aligned (coverage = 0.43), indicating extensive rearrangement that precluded reliable superposition.

Missing RMSD for aa. 147–226. This segment could not be assigned an RMSD value because SSM superposition failed entirely ("secondary structure does not match"), indicating profound refolding of secondary-structure elements between the prefusion and postfusion states. This failure itself constitutes strong evidence for major conformational rearrangement. A footnote has been added to Supplementary Table 1 to state this explicitly.

Changes in the manuscript:

1. **Figure 1A and legend:** We revised the figure color scheme and updated the legend to improve the distinguishability between prefusion and postfusion structures, and added the structural alignment coverage information for each segment comparison.
   Page 9–10, Line 162–168.
   “The postfusion conformation is shown in light gray throughout. Each prefusion segment is shown in a distinct color: residues 26–57 (red), 58–72 (chocolate), 73–96 (yellow), 137–226 (forest green), 227–305 (teal), 306–459 (blue), and 460–513 (purple). Segments were classified as similar or different based on SSM-based superposition statistics including coverage, backbone RMSD, and quality factor Q; classification criteria and complete alignment statistics are provided in Appendix Table S1.”

2. **Results section:** We revised the description of the SSM-based structural classification analysis to clarify the criteria used to define conformationally similar and different regions and their application in the mutational design strategy.
   Page 6, Line 117–124.
   "Based on structural superposition by secondary-structure matching (SSM), residues 26–57, 227–305, and 306–459 were classified as similar between the prefusion and postfusion conformations, while residues 58–72, 73–96, 147–226, and 460–513 showed substantial conformational divergence and were classified as different (Appendix Table S1). Rather than treating these differences as descriptive structural features alone, we used this organization to prioritize conformationally divergent regions, together with adjacent supporting framework, for focused mutational design."
3. **Methods section:** We added the SSM-based structural classification criteria used to define "similar" and "different" conformational regions.
   Page 39, Line 689–703.
   "2.Structural comparison and segment classification
   To quantitatively assess the conformational differences between the prefusion (PDB: 4JHW) and postfusion (PDB: 3RRR) states of RSV F, SSM-based structural superpositions were performed for seven functionally defined segments using the SUPERPOSE module implemented in CCP4 (version 8.0.015) (Krissinel & Henrick, 2004; Agirre et al, 2023). For each segment, the number of structurally equivalent residues used in the least-squares fit (Nalign), the coverage (Nalign/L, where L is the total number of residues in the segment), the backbone RMSD over aligned residues, and the SSM quality factor Q were recorded. Segments were classified as similar regions when all four of the following criteria were simultaneously satisfied: (1) SSM superposition succeeded; (2) coverage≥0.90; (3) backbone RMSD≤1.50 Å; and (4) Q≥0.02. Segments failing any one of these criteria, or for which SSM superposition could not be achieved due to mismatched secondary structure, were classified as different regions. Complete alignment statistics for all segments are provided in Appendix Table S1."
4. **Supplementary Table S1:** We added complete structural alignment statistics for all seven analyzed segments and included an annotation explaining the failed superposition of residues 147–226.
   "Appendix Table S1. SSM-based structural alignment statistics for seven segments of RSV F comparing the prefusion (PDB: 4JHW) and postfusion (PDB: 3RRR)."
   "*SSM superposition was not achieved due to mismatched secondary structure."

9. For Fig. 2A-B and Figure S2, it is recommended that the authors directly present the OD450 values instead of the converted fold change. In addition, if the authors want to highlight that certain mutations cause changes in the proportion of pre-fusion conformation, the horizontal and vertical axes of each subgraph should adopt the same range. In this way, the points above the diagonal (or a certain normalized line) will represent an increase in the proportion of pre-fusion conformation, and the upper right quadrant will correspond to an increase in expression level.

Response:

We thank the reviewer for this constructive suggestion regarding data presentation.

Fold change vs. raw OD450 values. We appreciate the rationale for presenting raw OD450 readings. After careful consideration, we have retained the fold change format, as normalization to the within-batch wild-type control is essential to minimize batch-to-batch variation inherent to transfection-based expression screening. Presenting raw OD450 values across independently performed batches would introduce confounding inter-batch variability and could be misleading, whereas fold change more accurately reflects the relative change in prefusion antigenicity for each substitution. A clarification of this normalization rationale has been added to the Methods section.

Unification of axis ranges. We appreciate this suggestion and agree that a uniform axis scale would in principle facilitate cross-panel comparison. However, the four mutation categories were evaluated against different selection thresholds reflecting their distinct design rationales: the proline, disulfide bond, and charge neutralization panels use a threshold of (0, 0), whereas the hydrophobic cavity-filling panel uses a more stringent threshold of (0, 1) owing to the larger dynamic range of this category. Imposing a uniform axis scale would compress the data in the first three panels to a degree that obscures the key substitutions identified within each category, without providing additional interpretive value. We have therefore retained independent axis scales, with dashed reference lines in each panel clearly indicating the respective quadrant boundaries and selection thresholds. The legends of Fig. 2A–B and Appendix Fig. S2 have been revised to explicitly state the threshold used for each category.

Changes in the manuscript:

1. **Methods section:** We added clarification describing the rationale for within-batch fold-change normalization in the expression-screening assays.
   Page 44, Line 792–798.
   "For all ELISA-based screening assays, binding signals were normalized to the concurrently processed wild-type RSV F (A2 strain) control within each experimental batch, and the results are expressed as log_2-transformed fold changes relative to wild-type F. This within-batch normalization was applied to minimize the influence of batch-to-batch variation inherent to transient transfection-based expression and to enable direct comparison of antigenicity across independently conducted screening rounds."
2. **Figure 2A-B and Appendix Figure S2 legends:** We revised the legends to define the dashed reference lines and specify the selection thresholds used for each mutation category.
   Page 15–16, Line 266–271.
   "Dashed lines indicate quadrant boundaries and category-specific selection thresholds: for all categories, vertical and horizontal dashed lines are set at x = 0 and y = 0; for hydrophobic cavity-filling variants, an additional horizontal dashed line at y = 1 indicates the twofold increase in D25 binding used for candidate selection. Red symbols indicate shortlisted candidates selected for downstream combinatorial analysis"

10. Line 214. It may be a typo, which should be M289F.

Response:

We thank the reviewer for catching this typographical error. We confirm that this was indeed a typo and have corrected it accordingly.

Changes in the manuscript:

1. **Results section:** We corrected the typographical error from "M298F" to "M289F."
   Page 13, Line 234.

11. Lines 223-224: The expression "disulfides did not improve..." seems inconsistent with the results presented in the middle graph of the second row of Figure S3. Fig. S3A shows that the N67L-V90Y-S190I-M289F-D486S protein has aggregations, while the N67L-V90Y-S190I-M289F-T267P-N268P-V482C-S502C-D486S protein has no apparent aggregates. From the current results, it is clear that disulfide bonds help enhance the homogeneity and stability of the trimer. In addition, from the results presented in Fig. S5D, I speculate that the authors used proteins purified in different batches for detection at the same time point to characterize the changes in protein homogeneity after being placed at different temperatures for different times. To make the experimental results more rigorous, I suggest that if possible, the authors present the SEC analysis of the same batch of purified protein samples stored for different times and overlay the

results. Otherwise, the authors should indicate in the text that the original proteins used in Figs. S3A, S5B, D, etc., are not from the same batch.

Response:

We thank the reviewer for this careful observation, which raises two related points. We address each below.

Wording of "disulfide bonds did not improve stability." We agree that the original wording at Lines 223-224 was overly broad and did not accurately reflect the full range of results shown in Appendix Fig. S3 (now Appendix Fig. S4). Upon re-examination, we wish to clarify the following: adding a single engineered disulfide bond to the cavity-filling core (N67L-V90Y-S190I-M289F-V482C-S502C) provided only marginal improvements in SEC peak homogeneity under thermal stress and ambient storage conditions relative to the four-mutation construct (Appendix Fig. S3A-B, now Appendix Fig. S4A-B). In contrast, the more complex multi-substitution combination incorporating both proline substitutions and disulfide bonds (N67L-V90Y-S190I-M289F-T267P-N268P-V482C-S502C-D486S) showed more pronounced homogeneity improvements, consistent with the reviewer's observation (Appendix Fig. S3C, now Appendix Fig. S4C). However, this came at the cost of a substantially higher number of non-native mutations. As the primary goal of this study was to achieve reliable prefusion stabilization while limiting the introduction of non-native substitutions to better preserve the integrity of key neutralizing epitopes and maintain a tractable molecular architecture. We prioritized the five-substitution design (N67L, V90Y, S190I, M289F, D486S). The text has been revised to more accurately describe the differential effects of the disulfide-containing constructs.

Batch identity and experimental workflow for Appendix Fig. S5 (now Appendix Fig. S6). We wish to clarify that all samples shown in Appendix Fig. S5B and S5D (now Appendix Fig. S6B and S6D) were derived from the same purified batch. The purified protein was aliquoted and stored at −80 °C. At the designated time points, aliquots were subjected to their respective storage conditions (−80 °C for 6 or 12 months, 4 °C for 6 months, or −20 °C for 6 months). CD measurements were performed immediately upon completion of each storage period. SEC and ELISA analyses for all conditions were performed in a single analytical session to ensure direct comparability and eliminate inter-session instrument variability. We acknowledge that the original text did not describe this workflow in sufficient detail and have revised the figure legends accordingly.

Changes in the manuscript:

1. **Results section:** We revised the description of the disulfide-containing constructs to distinguish the differential effects of single-disulfide and multi-substitution designs on trimer homogeneity and conformational stability, and clarified the rationale for prioritizing the five-substitution RVF-88 design despite the improved homogeneity observed in more complex disulfide-containing constructs..
   Page 13, Line 242–247.
   "However, biophysical characterization of purified proteins revealed that adding a single engineered disulfide bond to the cavity-filling core provided only marginal improvements in size-exclusion chromatography (SEC) homogeneity under thermal or storage stress. More complex multi-substitution combinations incorporating proline substitutions and disulfide bonds showed more consistent homogeneity (Appendix Fig. S4), but at the cost of a substantially higher number of non-native mutations."
2. **Results section:** We clarified that the stability and homogeneity analyses shown in Appendix Fig. S6 (formerly Appendix Fig. S5) were performed using samples derived from the same purified protein batch under different storage conditions.
   Page 17, Line 290–295.

"The purified RVF-88A and RVF-88B proteins exhibited high purity and retained stability after storage at 4 °C for six months and at -80 °C for twelve months, with RVF-88A showing a single symmetric main peak by SEC and RVF-88B maintaining a predominant monodisperse peak with minor secondary species under some conditions; both constructs maintained prefusion antigenicity throughout as confirmed by CD and ELISA (Appendix Fig. S6)."

3. **Methods section:** We added details describing the single-batch sample preparation workflow used for the stability analyses shown in Appendix Fig. S6 (formerly Appendix Fig. S5).
   Page 45, Line 810–812.
   "For both accelerated and long-term stability assessment of CHO-expressed RVF-88A and RVF-88B, proteins were derived from a single purified batch, aliquoted, and stored at −80°C until allocation to their respective storage conditions."
4. **Appendix Figure S5 legend:** We revised the legend to clarify that all stability analyses were performed using samples derived from a single purified protein batch.
   "All samples were derived from a single purified batch and subjected to the indicated storage conditions. "

12. Line 227. It is recommended that the authors remove the expression "minimal".

Response:

We thank the reviewer for this suggestion. We agree that "minimal" was not the most precise term in this context and have removed it accordingly.

Changes in the manuscript:

1. **Results section:** We removed the term "minimal" from the description of the RVF-88 design.
   Page 14, Line 250–253.
   "We therefore prioritized the five-substitution design (N67L, V90Y, S190I, M289F and D486S), termed RVF-88, for mechanistic validation as a selected optimized construct within the tested design framework."

13. Line 231. The authors should clearly define "optimal", that is, through which indicators in Fig. S4 can it be quantitatively considered that the selected mutations are optimal. Otherwise, it is recommended to weaken the relevant tone.

Response:

We thank the reviewer for this comment. We agree that "optimal" required clearer justification. The basis for this assessment was the systematic single-site substitution screen shown in Appendix Fig. S5, in which nearly all possible amino acid substitutions at positions 67, 90, 190, 289, and 486 were evaluated on both the WT and RVF-88 backbones using D25 and motavizumab binding as readouts under multiple conditions including thermal challenge and storage stress. The substitutions retained in the final design consistently ranked among the top performers across these readouts. Nevertheless, we agree that "optimal" may overstate the certainty of the selection, and have revised the wording accordingly.

Changes in the manuscript:

1. **Results section:** We removed the term "optimal" and revised the wording to describe the mutation-selection basis according to the systematic substitution-screening results shown in Appendix Fig. S5.

Page 14, Line 253–257.
"Introducing single-site substitutions at positions 67, 90, 190, 289 and 486 on both the WT and RVF-88 backbones further supported that the selected residues at their respective positions consistently ranked among the better-performing alternatives tested in terms of prefusion antigenicity, expression level, and conformational stability under thermal and storage stress (Appendix Fig. S5)."

14. Line 254. Although the expressions L88A and L88B are not incorrect, they are likely to be misunderstood as certain point mutations. It is recommended that the authors rename these two constructs throughout the full text to enhance the readability of the manuscript.

Response:

We thank the reviewer for this helpful suggestion. The original names RVF-88A and RVF-88B followed our internal nomenclature, in which "RVF" denotes RSV vaccine fusion protein, "L" denotes lead candidate, "88" refers to the screening identifier, and "A" and "B" denote the RSV A and B subtype backbones, respectively. However, we agree that the abbreviations L88A and L88B could be misread as point mutation notation. To improve readability and avoid ambiguity, we have renamed the constructs throughout the manuscript as RVF-88A and RVF-88B, and the corresponding bivalent formulation as RVF-88. We have also clarified at first mention that RVF-88A and RVF-88B denote the RSV A- and RSV B-derived antigen components, respectively, rather than amino-acid substitutions.

Changes in the manuscript:

1. **Throughout the manuscript:** We replaced "RVF-L88A" and "RVF-L88B" with "RVF-88A" and "RVF-88B," respectively, and renamed the corresponding bivalent formulation as "RVF-88".
   Page 14, Line 253–257.
   "Introducing single-site substitutions at positions 67, 90, 190, 289 and 486 on both the WT and RVF-88 backbones further supported that the selected residues at their respective positions consistently ranked among the better-performing alternatives tested in terms of prefusion antigenicity, expression level, and conformational stability under thermal and storage stress (Appendix Fig. S5)."
   Page 16, Line 279–281.
   "To enable translational development and broad subgroup coverage, we generated two RSV vaccine constructs based on the prevalent RSV-A ON1 and RSV-B BA9 lineages, termed RVF-88A and RVF-88B."
   Page 20, Line 337–340.
   "To assess immunogenicity, RVF-88 was formulated as a bivalent vaccine comprising RVF-88A and RVF-88B in a 1:1 mixture (by mass) and administered intramuscularly to mice (n=10 per group) on days 0 and 21 at total antigen doses of 2, 5, or 12.5 μg (Fig. 4A). "

15. Lines 258-261. What are the specific storage conditions? In which buffer is the protein stored? Is it in a lyophilized state? If it is lyophilized, what is the reconstitution buffer? Was centrifugation performed to remove precipitates before SEC analysis? In Fig. S5, the sample stored at -80{degree sign}C is labeled as L; is this the abbreviation of lyophilization? The authors should clearly state these details in the corresponding parts of the main text and methods.

Response:

We thank the reviewer for these detailed questions. We wish to clarify the following points. The purified RVF-88A and RVF-88B proteins were stored in liquid form in a 20 mM phosphate buffer containing stabilizing excipients. The proteins were not lyophilized. Prior to SEC analysis, samples were centrifuged at 12,000 rpm for 5 minutes to remove any potential particulates; no visible precipitate was observed under any

of the tested storage conditions. SEC analysis was performed on a Superose 6 Increase column with 1×PBS as the mobile phase.

Regarding the labels "A" and "L" in Appendix Fig. S5 (now Appendix Fig. S6), we wish to clarify that these are not abbreviations for lyophilization. Specifically, "A-6M" denotes accelerated stability testing at 4 °C for 6 months for RVF-88A and at −20 °C for 6 months for RVF-88B, as the accelerated stability condition for RVF-88B was set at −20 °C based on its formulation characteristics. "L-6M" and "L-12M" denote long-term stability testing at –80°C for 6 and 12 months, respectively. We apologize that these abbreviations were not clearly defined in the original figure legend and have now updated the legend accordingly. The storage conditions and SEC methodology have also been added to the Methods section.

Changes in the manuscript:

1. **Results section:** We added descriptions of the storage-stability evaluation workflow for RVF-88A and RVF-88B, including transient-expression SEC analysis, clinical-grade CHO production, and long-term stability characterization under different storage conditions.
   Page 16, Line 281–286.
   "To evaluate whether the RVF-88 mutation combination maintained favorable biophysical properties across different RSV F sequence backgrounds, corresponding constructs based on the A2 and BA9 backbones were transiently expressed in HEK293F cells and analyzed by SEC after ambient storage for 7 days. Both constructs showed predominant monodisperse peaks without evident degradation or major aggregation (Fig. 3A)."
   Page 17, Line 290–295.
   "The purified RVF-88A and RVF-88B proteins exhibited high purity and retained stability after storage at 4 °C for six months and at -80 °C for twelve months, with RVF-88A showing a single symmetric main peak by SEC and RVF-88B maintaining a predominant monodisperse peak with minor secondary species under some conditions; both constructs maintained prefusion antigenicity throughout as confirmed by CD and ELISA (Appendix Fig. S6)."
2. **Methods section:** We added detailed descriptions of protein storage conditions, buffer composition, SEC sample preparation, and centrifugation procedures prior to SEC analysis.
   Page 44–45, Line 801–820.
   "SEC was used to assess the homogeneity and stability of candidate proteins under multiple stress conditions on a Superose 6 Increase column (Cytiva) with 1× PBS as the mobile phase. Prior to injection, all samples were centrifuged at 12,000 rpm for 5 minutes; no visible precipitate was observed under any tested condition. For the initial stability screen of HEK293F-expressed constructs, samples formulated in 1× PBS were subjected to one freeze-thaw cycle, three freeze-thaw cycles, ambient storage for 7 days (RT-7D), or heat challenge at 55 °C for 1 hour. Freeze-thaw cycles were performed by thawing at room temperature until fully liquefied before re-freezing at −80 °C. For both accelerated and long-term stability assessment of CHO-expressed RVF-88A and RVF-88B, proteins were derived from a single purified batch, aliquoted, and stored at −80 °C until allocation to their respective storage conditions. All samples were formulated in 20 mM phosphate buffer and buffer-exchanged into 1× PBS prior to SEC analysis. Accelerated stability samples were stored at 4 °C for 6 months for RVF-88A and at −20 °C for 6 months for RVF-88B (A-6M), as the accelerated stability condition for RVF-88B was set at −20 °C based on its formulation characteristics. Long-term stability samples were stored at −80 °C for 6 months (L-6M) and 12 months (L-12M). SEC analyses for all time points and conditions were performed in a single analytical session to ensure direct comparability and eliminate inter-session instrument variability."
3. **Appendix Figure S6 (formerly Appendix Figure S5) legend:** We revised the legend to define the abbreviations "A-6M," "L-6M," and "L-12M," and to clarify the corresponding accelerated and

long-term stability conditions.

"Spectra (190–260 nm) are color-coded by storage condition: pink (0M, fresh untreated), blue (A-6M, accelerated stability: 4 °C for 6 months for RVF-88A, −20°C for 6 months for RVF-88B), purple (L-6M, long-term stability at –80 °C for 6 months), and green (L-12M, long-term stability at –80 °C for 12 months)."

16. Line 380. The expression "consistently higher" needs to be obtained after statistical significance analysis of the data, which is lacking in the figure legends of current Figs. 5B and C.

Response:

We thank the reviewer for this comment. We agree that the original expression "consistently higher" was not supported by formal statistical analysis. We have now performed statistical comparisons for all day-35 data shown in Fig. 5B and 5C. The results indicate that RVF-88 induced significantly higher antigen-specific binding antibody titers than ABRYSVO against both preF-A and preF-B ($P < 0.01$), whereas the differences in neutralizing antibody titers against RSV A2 and RSV B9320, although numerically higher in the RVF-88 group, did not reach statistical significance. The text and figure legends have been revised accordingly to accurately reflect these results.

Changes in the manuscript:

1. **Results section:** We revised the description of antibody responses to distinguish statistically significant differences in binding-antibody titers from numerically higher but non-significant neutralizing-antibody responses.
   Page 25, Line 423–428.
   "At two weeks after the booster immunization (day 35), RVF-88 induced significantly higher antigen-specific binding antibody titers than ABRYSVO against both preF-A and preF-B ($P < 0.001$). Neutralizing antibody titers against RSV A2 and RSV B9320 were also numerically higher in the RVF-88 group (2.6-fold and 3.4-fold, respectively), although these differences did not reach statistical significance."
2. **Figure 5B-C and legends:** We added statistical significance annotations and revised the legends to describe the statistical analyses and significance thresholds used.
   Page 28, Line 468–469.
   "Dashed lines indicate the limit of detection. Statistical significance was determined using one-way ANOVA with Tukey's post-hoc test; P values are indicated (ns not significant, *P<0.05, **P<0.01, ***P<0.001, ****P<0.0001). Group symbols and colours are defined in the legend."

17. Line 388. I did not observe "marked weight loss" in the results provided by the authors, and since only the fourth day was observed, the weight change data are of little significance. It is recommended to remove them.

Response:

We thank the reviewer for this comment. We agree that the body weight changes observed were minimal and that the four-day observation period further limits their informative value. We have therefore removed the body weight data, corresponding text, and associated figure panel from the revised manuscript.

Changes in the manuscript:

1. **Results section:** We removed the body-weight change data and corresponding text from the manuscript.
   Page 25, Line 431.

2. **Figure 5 and legends:** We removed the body-weight panel and renumbered the remaining figure panels accordingly.
   Page 27, Line 450.

18. Line 445. The authors need to explain the specific meaning of "conformational breathing" or provide the corresponding reference literature.

Response:

We thank the reviewer for this helpful comment. We agree that the term "conformational breathing" required clearer definition and supporting references if retained. To avoid introducing an insufficiently defined or potentially overinterpreted concept, we have removed this term from the revised manuscript. Instead, we now describe the structural analysis more specifically in terms of antibody-free prefusion-like architectures and local cavity reduction at the prefusion apex. The revised text focuses on antibody-free crystal structures of RVF-88 and cavity analysis performed within the same local search region and under identical detection parameters, without invoking "conformational breathing."

Changes in the manuscript:

1. **Results section:** We removed the term "conformational breathing" and revised the structural interpretation to focus on antibody-free prefusion-like architectures and local cavity-reduction analysis at the prefusion apex.
   Page 29, Line 484–491.
   "To complement the D25-bound cryo-EM analysis, antibody-free crystal structures of RVF-88A and RVF-88B were also analyzed and showed trimeric prefusion-like architectures consistent with the D25-bound cryo-EM structures (Appendix Fig. S15A and S15B). Local cavity analysis of the trimeric prefusion apex showed that RVF-88A had lower total cavity volume and surface area than selected prefusion F reference structures within the same local search region and under identical detection parameters, consistent with the intended cavity-reduction design at the prefusion apex (Appendix Fig. S15C, S15D; Appendix Table. S5)."

19. Line 453. Conclusions that N67L can "reinforced apex stability" cannot be drawn only from the analysis of the structural model, and the current structure has D25 antibody binding, making it even more impossible to judge whether the stability improvement is brought about by the introduction of N67L. It is recommended that the authors provide data such as the B-factor of the crystal structure or other observable data as evidence. Otherwise, the relevant expressions may be considered for removal.

Response:

We thank the reviewer for this important comment. We agree that the previous statement that N67L "reinforced apex stability" was too strong, because the independent stabilizing contribution of N67L cannot be concluded from the structural model alone, particularly in the context of a D25-bound structure. We have therefore removed this causal wording and revised the text to avoid assigning an independent stabilizing effect to this single substitution.

In the revised manuscript, we limit the interpretation of N67L to a structural observation. Specifically, N67L is located in the apex/apex-proximal region, and the local neighboring-residue environment around residue 67 is now shown descriptively in the antibody-free RVF-88A crystal structure and compared with the DS-Cav1 reference structure (PDB: 4MMU) in Appendix Fig. S15E. This figure is intended to illustrate the local structural environment around residue 67, not to infer that N67L alone improves prefusion F stability.

Accordingly, the revised text attributes the observed structural and antigenic properties of RVF-88 to the combined design rather than to an independently demonstrated effect of N67L.

Changes in the manuscript:

1. **Results section:** We removed the statement that N67L "reinforced apex stability" and revised the N67L-related wording to avoid assigning an independent stabilizing effect to this single substitution.
Page 29, Lines 494–498.
"Residue 67 is located in the apex/apex-proximal region of prefusion F, a region previously implicated in preF conformational stability. In RVF-88, N67 was replaced with leucine (N67L), which is oriented inward and is positioned near residues in the α1 region, whereas the corresponding N67 in DS-Cav1 is observed in a different local environment near the α4 region (Appendix Fig. S15E)."
2. **Appendix Figure S15E and legend:** We added Appendix Fig. S15E and revised the corresponding legend to show the local structural environment around residue 67 in RVF-88A and DS-Cav1 as a structural comparison.
"Appendix Fig. S15 (E) Local structural comparison at residue 67 between RVF-88A and DS-Cav1. Left, superposition of RVF-88A (plum) and DS-Cav1 (orange), with residue 67 displayed as spheres. Right, local views of residue 67 in RVF-88A and DS-Cav1 shown separately. Site residues are highlighted in magenta (stick) together with surrounding residues within 4 Å of the displayed atoms (stick)."

20. The following remark is for the current Fig. 6. It is recommended that the authors try to avoid using red, blue, and yellow to display side chain residues to prevent confusion with oxygen, nitrogen, and sulfur atoms.

Response:

We thank the reviewer for this suggestion. We agree that using red for mutated residues could potentially cause confusion with element-based heteroatom coloring. We have therefore changed the carbon backbone color of the mutated residues in Fig. 6 to magenta to clearly distinguish them from heteroatom colors (oxygen in orange, nitrogen in blue, sulfur in yellow). The figure legend has been updated accordingly.

Changes in the manuscript:

1. **Figure 6 and legend:** We changed the color of mutated residue carbon atoms to magenta to avoid confusion with element-based heteroatom coloring.
Page 32, Line 537–540.
"Site residues are highlighted in magenta (stick) together within 4 Å of the displayed atoms (stick). Cryo-EM density is shown as a mesh. Heteroatoms are colored by element: oxygen in orange, nitrogen in blue, and sulfur in yellow."

21. Please the authors check that the color code of each curve in a figure should be consistent, and fully correct the labels such as F1 and F3 to correspond to the main text. Other expressions such as L-12M should be explained in full in the figure legend. This remark applies to multiple figures including Fig. S5.

Response:

We thank the reviewer for this comment. We have carefully reviewed all figures in the manuscript for consistency in color coding between figure panels and their corresponding legends. Specifically, we have updated the figure legends to ensure that all color assignments accurately correspond to the curves and conditions shown. The labels F1 and F3, denoting one and three freeze-thaw cycles respectively, have been

corrected by the SEC trace labels to “1 freeze–thaw” and “3 freeze–thaw” respectively and defined consistently across all relevant figures and their legends. The abbreviation L-12M has been defined in full in the legend of Appendix Fig. S5 (now Appendix Fig. S6) as "long-term stability at −80°C for 12 months." We have also ensured that other abbreviated labels, including A-6M and L-6M, are defined in the same legend. These revisions apply to Appendix Fig. S5 (now Appendix Fig. S6) and all other figures where similar inconsistencies were identified.

Changes in the manuscript:

1. **Figure legends throughout the manuscript:** We corrected and standardized figure color coding and label definitions across all relevant figures.
   Page 19, Line 320–322.
   “Traces are color-coded by condition: blue (one freeze-thaw cycle), red (three freeze-thaw cycles), and green (RT-7D, ambient temperature storage for 7 days).”
2. **Appendix Figure S6 legend (formerly Appendix Figure S5):** We defined the abbreviations F1 and F3 as one and three freeze-thaw cycles, respectively, and clarified the meanings of A-6M, L-6M, and L-12M.
   “Spectra (190–260 nm) are color-coded by storage condition: pink (0M, fresh untreated), blue (A-6M, accelerated stability: 4 °C for 6 months for RVF-88A, −20°C for 6 months for RVF-88B), purple (L-6M, long-term stability at –80 °C for 6 months), and green (L-12M, long-term stability at –80 °C for 12 months).”

22. There seems to be a problem between the Model resolution range and Model resolution columns in Table S2. Please the authors check the content in Table S2 and verify it.

Response:

We thank the reviewer for identifying this inconsistency. Upon careful review of Appendix Table S2 (now Appendix Table S6), we confirmed that the previously reported “Model resolution (Å)” values in the Refinement section were inconsistent with the refined model–map FSC statistics. We have corrected these values to correspond to the masked model–map FSC = 0.5 resolutions of the refined structures: 3.18 Å for RVF-88A–D25 Fab and 3.00 Å for RVF-88B–D25 Fab. We have also removed the “Model resolution range (Å)” row from the Refinement section, as local map-resolution ranges are already reported separately in the Map resolution section of Appendix Table S2 (now Appendix Table S6).

Changes in the manuscript:

1. **Appendix Table S6 (formerly Appendix Table S2):** We corrected the “Model resolution (Å)” values in the Refinement section to correspond to the masked FSC = 0.5 threshold and removed the redundant “Model resolution range (Å)” row.
   “Model resolution (Å): 3.18 Å for RVF-88A–D25 Fab and 3.00 Å for RVF-88B–D25 Fab; FSC threshold: 0.5.”

Referee #3 (Comments on Novelty/Model System for Author):

Overall, this work ranks very strongly in identifying a new set of RSV preF-stabilized molecules that could be used as antigens for RSV vaccination. However, the superiority of this vaccine over other approved vaccines is not clear. Furthermore, safety data is inconclusive.

Response:

We thank the reviewer for the positive assessment of the novelty of this work, particularly the recognition of our structure-guided design strategy to identify new RSV preF-stabilized molecules as vaccine antigens. We also sincerely appreciate the reviewer's thoughtful comments, especially regarding the strength of the head-to-head evidence versus ABRYSVO and the still limited scope of the current safety assessment. We have carefully considered these concerns and revised the manuscript accordingly, including tempering the comparative claims versus ABRYSVO, adding the actual serum neutralizing antibody values, and more cautiously defining the scope of the protection and safety conclusions supported by the present data. We believe these revisions have improved the accuracy and overall rigor of the manuscript.

Referee #3 (Remarks for Author):

Structure-guided rational 1 design of a compact prefusion RSV F bivalent vaccine with enhanced immunogenicity (Liu et al.)

This work shows a newly designed Pre-fusion F (preF) protein from RSV of the A and B subgroups. The proposed new trimers of preF were generated through structure-guided design, and high-throughput screening was performed using specific mAbs (Motavizumab and D25). The screening and analysis yielded two new preF designs, which were further studied for immunogenicity, efficacy, and safety in mouse and cotton rat models of RSV A and RSV B infection. Comparisons were conducted with ABRYSVO, a recently approved bivalent RSV vaccine of similar composition.

Results from animal models show mild improvements in the generation of neutralizing and total antibodies by the candidate (RVF-L88) COMPARED TO ABRYSVO given at comparable doses in both models, with no statistical significance. Real numbers for serum neutralizing antibody titers should be presented in a table for comparison with previously published studies.

Response:

We thank the reviewer for this important and constructive comment. We agree that, when directly compared at comparable doses, the overall differences in binding and neutralizing antibody responses between RVF-88 and ABRYSVO were modest, and did not support a broad conclusion of superiority across all measured endpoints. We appreciate the opportunity to clarify our interpretation of the data and to provide more complete quantitative information.

Based on the current dataset, we believe a more accurate interpretation is that RVF-88 induced robust and reproducible immune responses relative to buffer control, whereas direct head-to-head comparisons with ABRYSVO showed statistically significant differences only for specific endpoints. Importantly, these selected differences still support the immunogenic potential of RVF-88. For example, in the mouse model, RVF-88 showed statistically significant advantages over ABRYSVO for selected neutralizing antibody readouts, including RSV A neutralization at the matched 5 µg dose and RSV A/B neutralization at the higher 12.5 µg dose. However, we have revised the manuscript to make clear that these findings should be interpreted as endpoint-specific differences rather than evidence of overall superiority over ABRYSVO.

In the cotton rat model, RVF-88 also induced broad binding antibody, neutralizing antibody, and protection-related responses relative to buffer control. However, we agree with the reviewer that direct RVF-88-versus-ABRYSVO comparisons in this model did not demonstrate a consistent statistically significant advantage across endpoints. We have therefore revised the corresponding text to avoid overstatement and to distinguish responses relative to the control group from direct comparisons with ABRYSVO.

We fully agree that presentation of the actual serum neutralizing antibody values would facilitate comparison with previously published studies. Accordingly, as suggested by the reviewer, we added a supplementary table (Appendix Table S4) that reports the real-number serum neutralizing antibody titers for both animal models, presented as geometric mean titers (GMTs) with 95% confidence intervals (95% CIs). Individual neutralization values are also provided in the Source Data files. This table facilitates direct quantitative comparison with previously published studies and improves transparency of data presentation.

We appreciate the reviewer's valuable suggestion, which has helped improve the clarity and rigor of the manuscript.

Changes in the manuscript:

1. **Results section 4:** We revised the wording describing the mouse immunogenicity and protection data to avoid overgeneralization of superiority over ABRYSVO and to clarify that statistically significant differences were limited to selected binding and neutralizing antibody endpoints.
   Page 20, Line 348–351.
   "Consistent with this, RVF-88 dose groups were broadly comparable to ABRYSVO across the measured immunogenicity and protection readouts, with significantly higher responses observed for selected binding and neutralizing antibody endpoints."
   Page 20, Line 354–357.
   "Serum neutralization titres (NT_{50} GMT) were numerically higher for RVF-88 than for ABRYSVO against RSV A2 (246 versus 51) and RSV B9320 (238 versus 74), with a statistically significant difference for RSV A at the matched 5 µg dose (Fig. 4D, E and Appendix Table. S4)."
2. **Results section 5:** We revised the wording describing the cotton rat immunogenicity and protection data to clarify that RVF-88 induced broad immune responses relative to the control group, whereas direct statistically significant differences versus ABRYSVO were limited to selected endpoints.
   Page 25, Line 423–428.
   "At two weeks after the booster immunization (day 35), RVF-88 induced significantly higher antigen-specific binding antibody titers than ABRYSVO against both preF-A and preF-B ($P < 0.001$). Neutralizing antibody titers against RSV A2 and RSV B9320 were also numerically higher in the RVF-88 group (2.6-fold and 3.4-fold, respectively), although these differences did not reach statistical significance."
3. **Appendix Table S4:** We added a table reporting serum neutralizing antibody titers as geometric mean titers (GMTs) with 95% confidence intervals (95% CIs) for both animal models.
   "Appendix Table S4. Serum neutralizing antibody titers in mouse and cotton rat immunogenicity studies."
4. **Figure 4, Figure 5, and corresponding legends:** We revised the figures and legends to explicitly indicate the statistical comparisons among RVF-88, ABRYSVO, and control groups.
   Page 24, Line 399–400 in Fig. 4; Page 28, Line 457–458 in Fig. 5.
   "The corresponding GMTs and 95% CIs are provided in Appendix Table S4, and individual data points are included in the Source Data files."

Protection was also analyzed in both models and correlated with the mild improvement in immunogenicity achieved by RVF-L88. In the mouse model (Figure 4), the animals were challenged only with RSV A subtype, and improvement was observed only at the level of RSV RNA analysis. In the cotton rat model, the challenge was performed with both RSV subtypes. Some improvement in lung protection against RSV B and in nasal protection against A2 was observed (Figure 5).

Response:

We thank the reviewer for this important comment. We agree that the protective effects of RVF-88 were not uniformly improved across all animal models, viral subtypes, and tissue compartments, and that the overall protection profile is broadly consistent with the trends observed in the immunogenicity data. At the same time, we would like to clarify that the protective effects shown by the current dataset were somewhat broader than suggested in the reviewer's summary.

In the mouse model, we acknowledge that only RSV A2 challenge was performed. This experiment followed an established A2 challenge setting in mice, whereas broader subtype-specific protection was further evaluated in the cotton rat model using both A2 and B9320. We would also like to clarify that the protective effect observed in mice was not limited to RSV RNA analysis. In addition to the significant reduction in lung viral RNA copies, all RVF-88-immunized groups also showed significant reductions in lung viral titers. ABRYSVO likewise showed a significant reduction in lung viral titers, but did not reach statistical significance for lung viral RNA copies.

In the cotton rat model, we agree that the protective effects were not uniform across viral subtypes and tissue compartments. However, a more precise summary is that RVF-88 showed significant protection in the lung against both A2 and B9320, as reflected by significant reductions in both lung viral RNA loads and lung viral titers. In the nasal turbinate, RVF-88 showed significant protection against A2, whereas protection against B9320 did not reach statistical significance. By contrast, ABRYSVO reached statistical significance for lung endpoints after A2 challenge, but did not show the same consistent pattern of protection for lung B9320 endpoints or nasal endpoints.

We have therefore revised the corresponding manuscript text to avoid overgeneralization and to more accurately describe the protection data as a tissue- and subtype-dependent pattern of virological protection. This revised wording also clarifies that, in the mouse model, protection was not limited to RNA-level improvement, and that, in the cotton rat model, clear virological protection was observed in the lung against both viral subtypes and in the nasal turbinate against A2.

We appreciate the reviewer's comment, which helped us more precisely define the scope and strength of the protection data.

Changes in the manuscript:

1. **Results sections 4:** We revised the wording describing the mouse protection data to avoid overgeneralization and to clarify that protection following RSV A2 challenge was observed at both the lung viral RNA and lung viral titer levels.
   Page 21, Line 363–372.
   "RVF-88 vaccination reduced lung viral RNA copies by 4.2- to 5.9-fold, whereas ABRYSVO showed a 2.9-fold reduction (Fig. 4H). Notably, all RVF-88 dose groups showed statistically significant reductions in lung viral RNA copies, whereas the reduction observed in the ABRYSVO group did not reach statistical significance (Fig. 4H). Additionally, lung viral titers in all RVF-88 immunized groups were reduced to minimal levels, representing highly significant protection compared with the buffer

control group (P<0.0001). In particular, the 5 µg and 12.5 µg RVF-88 doses demonstrated exceptional efficacy by reducing viral titers to undetectable levels below the limit of detection (LOD) (Fig. 4I)."

2. **Results sections 5:** We revised the wording describing the cotton rat protection data to more accurately reflect the tissue- and subtype-dependent nature of the observed virological protection.
   Page 25–26, Line 432–439.
   "Viral burden analyses in cotton rats showed that RVF-88 significantly reduced both lung viral RNA loads and lung viral titers after A2 and B9320 challenge relative to the buffer control. In contrast, ABRYSVO significantly reduced both endpoints after A2 challenge. After B9320 challenge, ABRYSVO significantly reduced lung viral titers but not lung viral RNA loads relative to the buffer control (Fig. 5E, F). In nasal turbinates, RVF-88 significantly reduced both viral load and viral titer compared with controls after A2 challenge (Fig. 5G), whereas neither RVF-88 nor ABRYSVO significantly reduced nasal viral burden after B9320 challenge (Fig. 5H)."

Safety studies shown are very basic. In cotton rats, body weight changes were observed, which are not a good indicator of RSV pathology (in fact, contrary to the mouse model, cotton rats do not lose weight after RSV infection). In addition, Fig. S13 shows lung sections stained with H&E from underinflated samples, which are difficult to compare and from which it is not possible to conclude that RVF-L88 "displayed minimal focal inflammatory lesions." As this model is the established safety model for vaccine-enhanced disease (VED), positive controls should be used and different vaccine doses tested, as VED could arise from vaccination with lower doses.

Response:

We thank the reviewer for raising this important point. We agree that the safety assessment presented in the current manuscript is preliminary and is not sufficient to constitute a rigorous xevaluation of vaccine-enhanced disease (VED). We also agree that body weight change is not an appropriate indicator of RSV pathology or VED in cotton rats. Accordingly, we have removed the cotton rat body weight data and the related interpretation from the revised manuscript.

We further agree that the original representative H&E-stained lung sections in Appendix Fig. S13 (now Appendix Fig. S14) were not sufficiently comparable in lung inflation status and did not support strong conclusions regarding inflammatory pathology. We have therefore replaced Appendix Fig. S13 (now Appendix Fig. S14) with representative sections of improved technical quality and revised the corresponding text to avoid overinterpretation based on qualitative histology images alone. In the revised manuscript, we limit the interpretation to the observation that RVF-88 did not show higher blinded quantitative lung pathology scores than the control groups under the tested conditions.

In addition to the safety-related observations from the RSV challenge models, we would also like to clarify that RVF-L88 has been evaluated in a repeated-dose intramuscular toxicity study in Sprague-Dawley rats. This study included a 6-week repeated dosing period followed by a 4-week recovery period. The evaluation covered clinical observations, mortality, body weight, food consumption, body temperature, ophthalmic examination, clinical pathology, immunological indicators, necropsy, gross pathology, and histopathological examination. Because the present manuscript focuses on antigen design, structural characterization, immunogenicity, and protective efficacy rather than formal toxicological development, we did not incorporate the full toxicology dataset into the manuscript. Instead, we have revised the manuscript to avoid overstating the safety-related conclusions and provide below a high-level summary of the additional repeat-dose toxicity findings to address the reviewer's concern.

Table R1. Summary of the repeated-dose intramuscular toxicity study of RVF-L88 in Sprague-Dawley rats

Assessment	Summary of findings
Study design	Repeated intramuscular administration in SD rats once every 3 weeks for 6 weeks, followed by a 4-week recovery period
Clinical observations and mortality	No mortality or moribundity was observed; no test article-related clinical abnormalities were identified
General toxicology endpoints	No toxicologically meaningful test article-related effects were identified in body weight, food consumption, body temperature, ophthalmic examination, hematology, coagulation, serum chemistry, urinalysis, cytokines, gross pathology, or systemic histopathology
Local tolerability	No test article-related local irritation was identified; mild microscopic injection-site findings were also present in controls and were considered related to mechanical irritation
Immunological findings	preF-A- and preF-B-specific IgG responses were induced. Transient T-cell subset changes and thyroid/parathyroid organ weight changes were observed after the final administration, recovered by the end of the recovery period, and were considered non-adverse and possibly related to immune responses
Overall conclusion	RVF-L88 was generally well tolerated under the tested repeat-dose intramuscular regimen, with no systemic toxicity, no test article-related local irritation, and no evidence of immune toxicity

Taken together, these results provide supportive information on the general tolerability of RVF-L88 under the tested repeated-dose intramuscular regimen. However, we recognize that these observations provide only supportive information on general tolerability and do not substitute for a dedicated VED assessment in the cotton rat RSV challenge model.

We also acknowledge the reviewer's point that a more rigorous VED assessment would ideally include a VED-positive control and dose-ranging conditions, particularly because lower-dose vaccination could potentially reveal different safety outcomes. As these conditions were not included in the present study, we have revised the manuscript to avoid interpreting the current findings as evidence excluding VED risk and instead describe them as preliminary safety-related observations under the tested conditions.

We appreciate the reviewer's valuable comments, which helped us more accurately define the scope and limitations of the current safety assessment.

Changes in the manuscript:

1. **Results section 4:** We revised the safety-related wording to avoid overinterpreting the current findings as evidence excluding ERD or VED risk.
   Page 21, Line 375–377.
   "Histopathological scoring revealed no increase in lung pathology in RVF-88 immunized mice compared with buffer or ABRYSVO under the conditions tested (Fig. 4J, Appendix Fig S11). "
2. **Results section 5:** We removed the cotton rat body-weight data and the related interpretation because body-weight change is not an appropriate indicator of RSV pathology or vaccine-enhanced disease in this model.
   Page 25, Line 431.
3. **Results section 5:** We removed wording stating that RVF-88 "displayed no or only minimal focal inflammatory lesions" and replaced it with a more conservative description aligned with the blinded

quantitative histopathology scoring results.
Page 26, Line 440–444.
"Representative H&E-stained lung sections from buffer-, ABRYSVO-, and RVF-88-immunized cotton rats after viral challenge were generally consistent with the quantitative histopathology scores shown in Fig. 5I and 5J and did not indicate increased lung inflammatory pathology in the RVF-88 group under the tested conditions (Appendix Fig. S14)."

4. **Appendix Figure S14 (formerly Appendix Figure S13):** We replaced the representative H&E-stained lung sections with images showing improved comparability of lung inflation status across groups and revised the legend to clarify that the images are provided as representative qualitative illustrations.
"These representative images are provided for qualitative illustration, while quantitative histopathology scoring is shown in Fig. 5I and 5J."

Overall, this work ranks very strongly in identifying a new set of RSV preF-stabilized molecules that could be used as antigens for RSV vaccination. However, the proof of superiority over ABRYSVO antigens (generated using a similar rationale) at the same dose is not convincing, as the data from both mice and cotton rats show similar results. Furthermore, very preliminary safety studies are presented, resulting in inconclusive findings.

20th Jul 2026

Dear Dr. Liu,

Thank you for submitting your revised manuscript to EMBO Molecular Medicine. Your manuscript will be ready for acceptance pending the following final amendments:

1) Please address referees' remaining minor concerns.
2) Title: Please consider revising the title to better highlight the main findings, e.g. "A cavity-reduced prefusion RSV F bivalent vaccine elicits durable and protective immunity".
3) Abstract: I have gone through your text and revised it (see below). Please review it for accuracy and amend as you see fit:

Respiratory syncytial virus (RSV) remains a major cause of severe respiratory disease, and stabilization of the prefusion (preF) conformation of the F glycoprotein is central for vaccine development. Here, we report a structure-guided engineering strategy that enhances preF stability by reducing the hydrophobic cavity within the trimeric F protein. Targeted modifications at metastability-associated sites generated RVF-88, a disulfide-free stabilized preF immunogen that preserves key neutralizing epitopes, including antigenic site Ø, while exhibiting improved structural integrity and long-term storage stability. Formulated as an unadjuvanted bivalent vaccine, RVF-88 elicited potent neutralizing antibody responses, robust antigen-specific cellular immunity, and durable immune protection lasting up to five months in mice. Vaccination also protected both mice and cotton rats against RSV challenge. Structural analyses confirmed the intended cavity-reduction design, revealing a reduced apical hydrophobic cavity volume and surface area while maintaining the prefusion architecture. Together, these findings establish hydrophobic cavity reduction as a rational strategy for stabilizing prefusion RSV F and provide a promising next-generation vaccine candidate with improved stability and immunogenicity for further clinical development.

4) Source Data: Please upload source data for the main figures as one zipped file per figure containing separate files for each panel in a given figure.
5) In the main manuscript file, please do the following:
- Please address all comments suggested by our data editors listed below:
o Figure legends:
1. Please note that the exact p values are not provided in the legends of 4B-E,H,I; 5B,E,F,G,H.
2. Please note that information related to n is missing in the legends 4B-E,G-J; 5B,C,E-J.
- Remove all figures and move their legends to the end of the file.
- Please correct the callout for Fig. S17 to Appendix Fig. S17. Please make sure that all figure and table callouts are accurate.
- In Methods, provide the antibody dilutions that were used for each antibody.
- In Methods, provide primer sequences used for the qRT-PCR reactions.
- In Methods, statistical paragraph should reflect all information that you have filled in the Authors Checklist, especially regarding randomization, blinding, replication etc.
- Indicate in legends number and nature of replicates and exact p= values, not a range, along with the statistical test used. To keep the figures "clear" some authors found providing an Appendix table Sx with all exact p-values preferable. You are welcome to do this if you want to.
- Please rename "Conflict of interest" to "Disclosure and competing interests statement". We updated our journal's competing interests policy in January 2022 and request authors to consider both actual and perceived competing interests. Please review the policy https://www.embopress.org/competing-interests and update your competing interests if necessary.
- Author contributions: Please remove it from the manuscript and specify author contributions in our submission system. CRediT has replaced the traditional author contributions section because it offers a systematic machine-readable author contributions format that allows for more effective research assessment. You are encouraged to use the free text boxes beneath each contributing author's name to add specific details on the author's contribution. More information is available in our guide to authors:
https://www.embopress.org/page/journal/17574684/authorguide#authorshipguidelines
- In Data availability statement please remove "The original contributions presented in the study are included in the main text or the supplementary materials" and "All materials and reagents generated in this study are available from the corresponding author with a completed Materials Transfer Agreement."
- Remove "Expanded View for this article is available online".
6) Appendix: Please remove separate figures and combined tables in Excel and upload the Appendix file in PDF format without track changes.
7) The Paper Explained: Please add it to the main manuscript file.
8) Synopsis:
- Synopsis image: Please upload the image as a high-resolution jpeg/png file exactly 550 px-wide x 300 pixels.
- Synopsis text: Please upload it as a separate .doc file.
- Please check your synopsis text and image before submission with your revised manuscript. Please be aware that in the proof stage minor corrections only are allowed (e.g., typos).
9) As part of the EMBO Publications transparent editorial process (see our Editorial at

http://embomolmed.embopress.org/content/2/9/329), EMBO Molecular Medicine will publish online a Review Process File (RPF) to accompany accepted manuscripts. This file will be published in conjunction with your paper and will include the anonymous referee reports, your point-by-point response and all pertinent correspondence relating to the manuscript. Let us know if you want to remove or not any figures from it prior to publication. Please note that the Authors checklist will be published at the end of the RPF.
10) Please provide a point-by-point letter INCLUDING my comments as well as the reviewer's reports and your detailed responses (as Word file).

I look forward to reading a new revised version of your manuscript as soon as possible.

Yours sincerely,

Zeljko Durdevic

Zeljko Durdevic
Senior Editor
EMBO Molecular Medicine

*** Instructions to submit your revised manuscript ***

*** PLEASE NOTE *** As part of the EMBO Publications transparent editorial process initiative (see our Editorial at https://www.embopress.org/doi/pdf/10.1002/emmm.201000094), EMBO Molecular Medicine will publish online a Review Process File to accompany accepted manuscripts.

In the event of acceptance, this file will be published in conjunction with your paper and will include the anonymous referee reports, your point-by-point response and all pertinent correspondence relating to the manuscript. If you do NOT want this file to be published, please inform the editorial office at contact@embomolmed.org.

*****

When preparing your revised manuscript, please refer to our guidelines: https://link.springer.com/journal/44321/submission-guidelines#cms-Revised-submissions. We perform an initial quality control of all revised manuscripts before re-review; failure to include requested items will delay the evaluation of your revision.

We require:

1) A .docx formatted version of the manuscript text (including legends for main figures, EV figures and tables). Please make sure that the changes are highlighted to be clearly visible.

2) Individual production quality figure files as .eps, .tif, .jpg (one file per figure). For guidance, download the 'Figure Guide PDF': https://media.springernature.com/original/springer-cms/rest/v1/content/27825798/data/v1.

3) A .docx formatted letter INCLUDING the reviewers' reports and your detailed point-by-point responses to their comments. As part of the EMBO Press transparent editorial process, the point-by-point response is part of the Review Process File (RPF), which will be published alongside your paper.

4) A complete author checklist, which you can download from our author guidelines. Please insert information in the checklist that is also reflected in the manuscript. The completed author checklist will also be part of the RPF.

5) Please note that all corresponding authors are required to supply an ORCID ID for their name upon submission of a revised manuscript.

6) It is mandatory to include a 'Data Availability' section after the Materials and Methods. Before submitting your revision, primary datasets produced in this study need to be deposited in an appropriate public database, and the accession numbers and

database listed under 'Data Availability'. Please remember to provide a reviewer password if the datasets are not yet public.

In case you have no data that requires deposition in a public database, please state so in this section. Note that the Data Availability Section is restricted to new primary data that are part of this study.

7) For data quantification: please specify the name of the statistical test used to generate error bars and P values, the number (n) of independent experiments (specify technical or biological replicates) underlying each data point and the test used to calculate p-values in each figure legend. The figure legends should contain a basic description of n, P and the test applied. Graphs must include a description of the bars and the error bars (s.d., s.e.m.).

8) At EMBO Press we ask authors to provide source data for the main manuscript figures. You will receive a separate email with instructions for providing source data with your revised manuscript, including how to upload and organize the files.

9) Our journal encourages inclusion of *data citations in the reference list* to directly cite datasets that were re-used and obtained from public databases. Data citations in the article text are distinct from normal bibliographical citations and should directly link to the database records from which the data can be accessed. In the main text, data citations are formatted as follows: "Data ref: Smith et al, 2001" or "Data ref: NCBI Sequence Read Archive PRJNA342805, 2017". In the Reference list, data citations must be labeled with "[DATASET]". A data reference must provide the database name, accession number/identifiers and a resolvable link to the landing page from which the data can be accessed at the end of the reference.

10) We replaced Supplementary Information with Expanded View (EV) Figures and Tables that are collapsible/expandable online. A maximum of 5 EV Figures can be typeset. EV Figures should be cited as 'Figure EV1, Figure EV2" etc... in the text and their respective legends should be included in the main text after the legends of regular figures.

- For the figures that you do NOT wish to display as Expanded View figures, they should be bundled together with their legends in a single PDF file called *Appendix*, which should start with a short Table of Content. Appendix figures should be referred to in the main text as: "Appendix Figure S1, Appendix Figure S2" etc.

- Additional Tables/Datasets should be labeled and referred to as Table EV1, Dataset EV1, etc. Legends have to be provided in a separate tab in case of .xls files. Alternatively, the legend can be supplied as a separate text file (README) and zipped together with the Table/Dataset file.

11) The paper explained: EMBO Molecular Medicine articles are accompanied by a summary of the articles to emphasize the major findings in the paper and their medical implications for the non-specialist reader. Please provide a draft summary of your article highlighting

- the medical issue you are addressing,

- the results obtained and

- their clinical impact.

This may be edited to ensure that readers understand the significance and context of the research. Please refer to any of our published articles for an example.

12) Author contributions: You will be asked to provide CRediT (Contributor Role Taxonomy) terms in the submission system. These replace a narrative author contribution section in the manuscript.

13) A Conflict of Interest statement should be provided in the main text.

14) Every published paper includes a 'Synopsis' to further enhance discoverability. Synopses are displayed on the journal webpage and are freely accessible to all readers. They include a short stand first (maximum of 300 characters, including space) as well as 2-5 one-sentences bullet points that summarizes the paper. Please write the bullet points to summarize the key NEW findings. They should be designed to be complementary to the abstract - i.e. not repeat the same text. We encourage inclusion of key acronyms and quantitative information (maximum of 30 words / bullet point). Please use the passive voice. Please attach these in a separate file or send them by email, we will incorporate them accordingly.

Please also suggest a striking image or visual abstract to illustrate your article as a PNG file 550 px wide x 300 px high.

15) Include a Reagents and Tools Table as part of the Methods section, which can be downloaded from our author guidelines.

EMBO Molecular Medicine has a "scooping protection" policy, whereby similar findings that are published by others during review or revision are not a criterion for rejection. Should you decide to submit a revised version, I do ask that you get in touch

after three months if you have not completed it, to update us on the status.

*Additional important information regarding Figures

Each figure should be given in a separate file and should have the following resolution:
Graphs 800-1,200 DPI
Photos 400-800 DPI
Colour (only CMYK) 300-400 DPI"

Figures are not edited by the production team. All lettering should be the same size and style; figure panels should be indicated by capital letters (A, B, C etc). Gridlines are not allowed except for log plots. Figures should be numbered in the order of their appearance in the text with Arabic numerals. Each Figure must have a separate legend and a caption is needed for each panel.

*Additional important information regarding figures and illustrations can be found at
https://media.springernature.com/original/springer-cms/rest/v1/content/27825798/data/v1

***** Reviewer's comments *****

Referee #2 (Comments on Novelty/Model System for Author):

I consider the authors' screening approach to be effective, yet the process remains somewhat cumbersome, and the technical route is relatively conventional. Given that multiple publications on mutations stabilizing the RSV prefusion conformation have already been reported, the novelty of this work is to some extent diminished. Nevertheless, the novel and effective mutation combination for stabilizing the prefusion conformation reported herein still holds certain reference value and is worthy of publication.
There are several instances throughout this manuscript where essential data are lacking the requisite three biological replicates. The authors are advised to provide triplicate experiments wherever feasible; for screening results, duplicate experimental data should also be presented. This pertains to Figure 2, Figure 3B-C, and the corresponding supplementary figures. In light of these considerations, I would classify the technical quality of this manuscript as medium.

Referee #2 (Remarks for Author):

I thank the authors for their detailed and well-organized response. The revised manuscript has improved substantially compared to the initial draft. I believe the authors have addressed the vast majority of my concerns, and I consider this manuscript acceptable for publication. Beyond this, regarding the current revision and response, I would like the authors to provide further clarification on the following minor points to avoid potential confusion:

The substantial variation in ELISA values shown in Figure 3B remains a phenomenon that may significantly undermine its credibility. While the authors maintain that these differences are justified, I recommend that the authors provide three or more biological replicates to demonstrate the robustness of their experimental approach.

In my original response, minor point 9 referred to the data presentation in Figure 2A and Figure S2. My understanding is that the ELISA fold change for Mota corresponds to changes in expression level, whereas the ELISA mut-to-WT fold change for D25 reflects the enhancement of prefusion conformation expression. Under this interpretation, should not the fold change for D25 be greater than that for Mota to indicate both increased expression and an elevated proportion of prefusion conformation? If my understanding above is correct, I would still like the authors to explain the rationale for selecting (0, 0) or (0, 1) as threshold values. Are there analogous references in the literature that have employed such screening criteria?

Referee #3 (Comments on Novelty/Model System for Author):

There is a strong and well-detailed characterization of the vaccine antigen. In this resubmission, the in vivo efficacy studies are now stronger, with results that are adequately presented and interpreted. However, an attempt to define RSV vaccine safety falls short due to technical shortcomings in demonstrating clear features of RSV-induced lung pathology in cotton rats and Th1-Th2 profiles in mice.

Referee #3 (Remarks for Author):

Resubmission: Structure-guided rational design of a prefusion RSV F bivalent vaccine with enhanced immunogenicity (Liu et al.)

This work presents a newly designed pre-fusion F (preF) protein from RSV subgroups A and B. The proposed preF trimers were generated through structure-guided design, and high-throughput screening was performed using specific mAbs (Motavizumab and D25). The screening and analysis yielded two new preF designs, which were further evaluated for immunogenicity, efficacy, and safety in mouse and cotton rat models of RSV A and RSV B infection. Comparisons were conducted with ABRYSVO, a recently approved bivalent RSV vaccine of similar composition.

Results from animal models still show mild improvements in the generation of neutralizing and total antibodies by the candidate (RVF-L88) compared with ABRYSVO when both are administered at comparable doses in each model. Now, many of the previous conclusions of superiority have been tempered, and statistical differences are reported alongside the numerical results.

It is worth noting that serum IgG titers against the pre-Fs were generally higher in RVF-88-vaccinated animals than in ABRYSVO-vaccinated animals (as mentioned in lines 351 and 423 and shown in Fig. 4B and 4C and in Fig. 5B, left graphs). However, this may be because the ELISA setup uses RVF-88A and B to coat plates (line 774), which could give an advantage to the homologous antigen. It will be helpful to add this to the discussion. In addition, the statement starting at line 370 must be revised to reflect that one animal in the 12.5 g dose RV-F88-vaccinated group shows detectable viral titers in the lung (Fig. 4I). Line 374 should specify the day after viral challenge on which the pathology was assessed.

It is unclear what the relevance of the IFN-γ ELISpot is. It needs to be explained why it was analyzed. First, the differences are not significant enough to conclude that there is an increase in T-cell response. Second, the IL-4 ELISpot is missing if the purpose is to assess a Th1 vs Th2 response.

Regarding the durability of the antibody response, it should be discussed why some animals do not show detectable serum-neutralizing antibodies (Figure S13).

Line 426. "Neutralizing antibody titers.... were also numerically higher.... than in the AVRYSVO group...(add underlined)

Line 428, "To evaluate protection..." should start a new paragraph that contains the following sentence.

Remove "In contrast" from the sentence on line 516, and add "However," at the beginning of the following sentence before the word "after".

Fig. S14 still shows H&E-stained lung sections from underinflated samples, mostly from animals that were mock-vaccinated or ABRYSVO-vaccinated, which confound the observations. In addition, the same underinflation prevents one from appreciating the arrow-marked thickness of alveolar walls or the hyperplasia of alveolar epithelial cells mentioned in the figure's text. I would recommend selecting similar fields without collapsed airways and omitting arrows if the lung pathology is considered indistinguishable between groups.

It would be helpful to provide a more detailed description of the pathology scoring methodology, as RSV-induced lung damage affects lung structures in different ways.

***** Reviewer's comments *****

Referee #2 (Comments on Novelty/Model System for Author):

I consider the authors' screening approach to be effective, yet the process remains somewhat cumbersome, and the technical route is relatively conventional. Given that multiple publications on mutations stabilizing the RSV prefusion conformation have already been reported, the novelty of this work is to some extent diminished. Nevertheless, the novel and effective mutation combination for stabilizing the prefusion conformation reported herein still holds certain reference value and is worthy of publication.
There are several instances throughout this manuscript where essential data are lacking the requisite three biological replicates. The authors are advised to provide triplicate experiments wherever feasible; for screening results, duplicate experimental data should also be presented. This pertains to Figure 2, Figure 3B-C, and the corresponding supplementary figures. In light of these considerations, I would classify the technical quality of this manuscript as medium.

Response:

We thank the reviewer for this important comment regarding experimental reproducibility. We agree that reproducibility is essential for both screening and subsequent validation of RSV F variants. For the large-scale screening shown in Figure 2 and the corresponding supplementary figures, the assays were used to prioritize candidate substitutions from a large mutation library, and selected candidates were further evaluated in independent experiments. For the antigenic characterization of RVF-88A, RVF-88B, and pXCS847A, independent validation experiments were performed to confirm the reproducibility of the observed antibody-binding profiles.

Referee #2 (Remarks for Author):

I thank the authors for their detailed and well-organized response. The revised manuscript has improved substantially compared to the initial draft. I believe the authors have addressed the vast majority of my concerns, and I consider this manuscript acceptable for publication. Beyond this, regarding the current revision and response, I would like the authors to provide further clarification on the following minor points to avoid potential confusion:

The substantial variation in ELISA values shown in Figure 3B remains a phenomenon that may significantly undermine its credibility. While the authors maintain that these differences are justified, I recommend that the authors provide three or more biological replicates to demonstrate the robustness of their experimental approach.

Response:

We thank the reviewer for highlighting this important concern. We agree that independent validation is essential to demonstrate the robustness of the antigenicity characterization. We have performed additional independent experiments for the binding characterization of RVF-88A, RVF-88B, and pXCS847A against the monoclonal antibody panel and incorporated the variability among independent experiments into the revised analysis.

Changes in the manuscript:

1. **Figure 3B and legend**: The quantitative summary of monoclonal antibody binding profiles has been revised to include independent experimental replicates.
Page 57, Lines 1236-1238.
"Data are presented as geometric mean EC_{50} values with geometric SD from four independent experiments, each performed in technical duplicate (n = 4)."

2. **Appendix Figure S7**: Representative ELISA binding curves are provided from one independent experiment to illustrate the binding profiles of RVF-88A, RVF-88B, and pXCS847A.
"Representative ELISA binding curves from one independent experiment are shown. Data are shown as mean ± SD of duplicate technical replicates, with fitted EC_{50} values indicated in each panel. Quantitative EC_{50} values from four independent experiments are summarized in Fig. 3B."

In my original response, minor point 9 referred to the data presentation in Figure 2A and Figure S2. My understanding is that the ELISA fold change for Mota corresponds to changes in expression level, whereas the ELISA mut-to-WT fold change for D25 reflects the enhancement of prefusion conformation expression. Under this interpretation, should not the fold change for D25 be greater than that for Mota to indicate both increased expression and an elevated proportion of prefusion conformation? If my understanding above is correct, I would still like the authors to explain the rationale for selecting (0, 0) or (0, 1) as threshold values. Are there analogous references in the literature that have employed such screening criteria?

Response:

We thank the reviewer for this insightful comment. We agree that D25 binding provides an important readout of prefusion-specific antigenicity, and variants with improved prefusion enrichment may be expected to show increased D25 reactivity. In our screening strategy, D25 and Motavizumab were used as complementary readouts to evaluate prefusion-specific antigenicity and overall F protein expression, respectively.

We acknowledge that D25 binding is influenced by both the abundance of prefusion F antigen and the preservation/accessibility of the prefusion-specific site Ø epitope. Therefore, D25 and Motavizumab signals share a dependence on antigen abundance

but provide different information regarding antigenic conformation. Because D25 binding is additionally affected by epitope accessibility and local conformational presentation, the relationship between D25 and Motavizumab fold changes is not expected to be a simple proportional relationship. Therefore, increased D25 binding does not necessarily require a proportional increase relative to Motavizumab binding.

The selection thresholds were designed to prioritize variants with improved prefusion-specific antigenicity while maintaining sufficient expression. The $x = 0$ threshold represents preservation of wild-type-level Motavizumab binding, whereas the $y = 0$ threshold represents preservation of wild-type-level D25 binding. Thus, variants located above the $y = 0$ threshold were considered to maintain or improve prefusion-specific antigenicity relative to WT while avoiding loss of overall F protein expression. For hydrophobic cavity-filling variants, the additional $y = 1$ threshold was applied to prioritize substitutions showing at least a twofold increase in D25 binding, reflecting the larger dynamic range observed in this screening category. The purpose of this initial screening was to identify substitutions that preserved or improved prefusion antigenicity rather than simply maximizing D25 binding signals.

Previous RSV F antigen engineering studies have used prefusion-specific antibodies such as D25 together with antibodies recognizing conserved F epitopes, including Motavizumab, as complementary indicators to evaluate prefusion antigenicity and antigenic integrity (Crank et al, 2015; Lee et al, 2024).

References:

Crank MC, Ruckwardt TJ, Chen M, Mas V, Kumar A, et al (2015) A proof of concept for structure-based vaccine design targeting RSV prefusion F protein. *Science* 350: 835–839

Lee J, Kim H, Park J, Lee S, et al. (2024) Engineered dityrosine-bonding of the RSV prefusion F protein imparts stability and potency advantages. *Nature Communications* 15: 2514.

Referee #3 (Comments on Novelty/Model System for Author):

There is a strong and well-detailed characterization of the vaccine antigen. In this resubmission, the in vivo efficacy studies are now stronger, with results that are adequately presented and interpreted. However, an attempt to define RSV vaccine safety falls short due to technical shortcomings in demonstrating clear features of RSV-induced lung pathology in cotton rats and Th1-Th2 profiles in mice.

Referee #3 (Remarks for Author):

Resubmission: Structure-guided rational design of a prefusion RSV F bivalent vaccine with enhanced immunogenicity (Liu et al.)

This work presents a newly designed pre-fusion F (preF) protein from RSV subgroups A and B. The proposed preF trimers were generated through structure-guided design, and high-throughput screening was performed using specific mAbs (Motavizumab and D25). The screening and analysis yielded two new preF designs, which were further evaluated for immunogenicity, efficacy, and safety in mouse and cotton rat models of RSV A and RSV B infection. Comparisons were conducted with ABRYSVO, a recently approved bivalent RSV vaccine of similar composition.

Results from animal models still show mild improvements in the generation of neutralizing and total antibodies by the candidate (RVF-L88) compared with ABRYSVO when both are administered at comparable doses in each model. Now, many of the previous conclusions of superiority have been tempered, and statistical differences are reported alongside the numerical results.

It is worth noting that serum IgG titers against the pre-Fs were generally higher in RVF-88-vaccinated animals than in ABRYSVO-vaccinated animals (as mentioned in lines 351 and 423 and shown in Fig. 4B and 4C and in Fig. 5B, left graphs). However, this may be because the ELISA setup uses RVF-88A and B to coat plates (line 774), which could give an advantage to the homologous antigen. It will be helpful to add this to the discussion. In addition, the statement starting at line 370 must be revised to reflect that one animal in the 12.5 μg dose RV-F88-vaccinated group shows detectable viral titers in the lung (Fig. 4I). Line 374 should specify the day after viral challenge on which the pathology was assessed.

Response:

We thank the reviewer for pointing out these issues. We agree that the use of RVF-88A and RVF-88B as coating antigens in ELISA assays may influence the comparison of binding antibody titers between vaccine groups. We have revised the statement to avoid overstating the protective effect and to accurately reflect the variability in viral titers among individual animals. In addition, we have added the specific time point of lung pathology assessment.

Changes in the manuscript:

1. **Discussion:** We have added a statement regarding the potential influence of homologous antigen coating on ELISA-based binding antibody measurements and clarified the complementary evidence provided by functional assays.
Page 26, Lines 537-541.
"The higher binding antibody titers observed in RVF-88-vaccinated animals may be partially influenced by the use of RVF-88A and RVF-88B as coating antigens in the

ELISA assays, which could favor detection of homologous antibody responses. Nevertheless, functional neutralization assays and in vivo challenge studies further supported the immunogenicity and protective potential of RVF-88."

2. **Result:** We have revised the description of viral reduction after RSV challenge to clarify that RVF-88 substantially reduced pulmonary viral titers in most animals.
Page 17, Lines 342-344.
"In particular, the 5 μg and 12.5 μg RVF-88 doses demonstrated strong efficacy by substantially reducing pulmonary viral titers, with viral levels reduced to below the limit of detection (LOD) in most animals (Fig. 4I)."

3. **Result:** We have revised the description of pathological analysis to clarify the timing of lung tissue collection after RSV challenge.
Page 17, Lines 346-348.
"Pathological analysis was performed on lung tissues collected at day 5 post-challenge to assess any tissue damage across different treatment groups."

It is unclear what the relevance of the IFN-γ ELISpot is. It needs to be explained why it was analyzed. First, the differences are not significant enough to conclude that there is an increase in T-cell response. Second, the IL-4 ELISpot is missing if the purpose is to assess a Th1 vs Th2 response.

Response:

We thank the reviewer for this important comment. We agree that the IFN-γ ELISpot results do not demonstrate enhanced cellular immunity, as no significant differences were observed between groups. The assay was intended to evaluate RSV-specific cellular immune responses following vaccination rather than to define T-cell polarization. We have revised the interpretation accordingly.

Changes in the manuscript:

Result: We have revised the description of the IFN-γ ELISpot results to clarify that these data indicate detectable RSV-specific cellular immune responses without implying enhanced T-cell immunity.
Page 17, Lines 350-352.
"ELISpot (Appendix Fig. S12) showed detectable RSV-specific IFN-γ responses following RVF-88 vaccination, although differences versus controls were not significant."

Regarding the durability of the antibody response, it should be discussed why some animals do not show detectable serum-neutralizing antibodies (Figure S13).

Response:

We thank the reviewer for highlighting this point. We have added a discussion regarding the variability observed in long-term neutralizing antibody responses and possible factors contributing to undetectable antibody titers at later time points.

Changes in the manuscript:

Discussion: We have added a statement discussing individual variability and assay-related factors affecting long-term neutralizing antibody detection.
Page 26, Lines 541-546.
"During long-term follow-up, some individual animals showed undetectable serum neutralizing antibody titers at later time points, potentially reflecting individual variability in antibody responses and the detection limits of the assay. Importantly, most animals maintained detectable neutralizing activity throughout the observation period."

Line 426. "Neutralizing antibody titers.... were also numerically higher.... than in the AVRYSVO group...(add underlined)

Response:

We thank the reviewer for this suggestion. We have revised the sentence to explicitly indicate that the higher neutralizing antibody titers were numerical differences without statistical significance.

Changes in the manuscript:

Result: We have revised the description of neutralizing antibody responses to clarify the comparison between RVF-88 and ABRYSVO groups.
Page 18, Lines 372-375.
"Neutralizing antibody titers against RSV A2 and RSV B9320 were also numerically higher in the RVF-88 group than in the ABRYSVO group (2.6-fold and 3.4-fold, respectively), although these differences did not reach statistical significance."

Line 428, "To evaluate protection..." should start a new paragraph that contains the following sentence.

Response:

We thank the reviewer for this suggestion.

Changes in the manuscript:

We have reorganized the paragraph structure in the Results section accordingly.
Page 18, Lines 376.

Remove "In contrast" from the sentence on line 516, and add "However," at the beginning of the following sentence before the word "after".

Response:

We thank the reviewer for this careful reading.

Changes in the manuscript:

We have revised the wording as suggested.
Page 18-19, Lines 380-383.
“ABRYSVO significantly reduced both endpoints after A2 challenge. However, after B9320 challenge, ABRYSVO significantly reduced lung viral titers but not lung viral RNA loads relative to the buffer control (Fig. 5E, F).”

Fig. S14 still shows H&E-stained lung sections from underinflated samples, mostly from animals that were mock-vaccinated or ABRYSVO-vaccinated, which confound the observations. In addition, the same underinflation prevents one from appreciating the arrow-marked thickness of alveolar walls or the hyperplasia of alveolar epithelial cells mentioned in the figure's text. I would recommend selecting similar fields without collapsed airways and omitting arrows if the lung pathology is considered indistinguishable between groups.

Response:

We thank the reviewer for this valuable suggestion. We agree that tissue inflation status and representative field selection are important considerations for lung histopathological evaluation. We have therefore carefully reviewed the lung sections and replaced the representative H&E images in Fig. S14 with fields showing better-preserved lung architecture and more comparable alveolar structures across groups. In addition, arrows highlighting specific morphological features were removed to avoid potential overinterpretation. Quantitative histopathological scoring was retained as the primary approach for comparative evaluation between groups.

Changes in the manuscript:

Figure S14 and legend: We replaced the representative H&E-stained lung images with fields showing better-preserved lung architecture and removed arrows indicating specific morphological features. The figure legend was revised accordingly.
“Appendix Figure S14. Histological analysis of lung tissue in cotton rat following immunization and viral challenge.
Representative hematoxylin and eosin (H&E) staining of lung sections from cotton rats immunized with buffer control, ABRYSVO, or RVF-88, and challenged intranasally with RSV A2 (A) or RSV B9320 (B). Representative images are shown for qualitative assessment of lung histopathology, while quantitative histopathology

scoring is shown in Fig. 5I and 5J."

It would be helpful to provide a more detailed description of the pathology scoring methodology, as RSV-induced lung damage affects lung structures in different ways.

Response:

We thank the reviewer for this valuable suggestion. We agree that RSV-induced lung pathology involves multiple structural components and that a more detailed description of the histopathological scoring methodology is important for interpretation and reproducibility. We have revised the Methods section to clarify the evaluated pathological features and the criteria used for severity scoring.

Changes in the manuscript:

Methods: We have expanded the description of the histopathological scoring methodology to include the evaluated lung structures, severity criteria, and scoring procedure.

Page 43-44, Lines 905-913.

"Histopathological changes were evaluated by examining inflammatory infiltration in the airways, blood vessels, and interstitial spaces. Lesions were semi-quantitatively scored according to the severity and extent of lung involvement as follows: 1, minimal inflammation, with less than 20% of lung tissue affected; 2, mild inflammation, with 20–40% of lung tissue affected; 3, moderate inflammation, with 40–60% of lung tissue affected; 4, marked inflammation, with more than 60% of lung tissue affected; and 5, severe inflammation, characterized by extensive tissue damage or structural disruption. Scoring was performed in a blinded manner by two independent investigators."

11th Aug 2026

Dear Dr. Liu,

We are pleased to inform you that your manuscript is accepted for publication and is now being sent to our publisher to be included in the next available issue of EMBO Molecular Medicine.

Your manuscript will be processed for publication by EMBO Press. It will be copy edited and you will receive page proofs prior to publication. Please note that you will be contacted by Springer Nature Author Services to complete licensing and payment information.

You may qualify for financial assistance for your publication charges - either via a Springer Nature fully open access agreement or an EMBO initiative. Check your eligibility: https://link.springer.com/journal/44321/how-to-publish-with-us

Should you be planning a Press Release on your article, please notify the production team via this form in order to coordinate publication and release dates: https://forms.cloud.microsoft/e/eH4A8JVP6g

If you have any questions, please do not hesitate to contact the Editorial Office. Thank you for your contribution to EMBO Molecular Medicine.

Yours sincerely,
Zeljko Durdevic

Zeljko Durdevic
Senior Editor
EMBO Molecular Medicine

>>> Please note that it is EMBO Molecular Medicine policy for the transcript of the editorial process (containing referee reports and your response letter) to be published as an online supplement to each paper. If you do NOT want this, you will need to inform the Editorial Office via email immediately. More information is available here: https://link.springer.com/partners/embo-press/editorial-policies#Peer%20review

EMBO Press Author Checklist

Corresponding Author Name: Lijie Liu	
Journal Submitted to: EMBO Molecular Medicine	
Manuscript Number: EMM-2026-23468	

USEFUL LINKS FOR COMPLETING THIS FORM
The EMBO Journal - Author Guidelines
EMBO Reports - Author Guidelines
Molecular Systems Biology - Author Guidelines
EMBO Molecular Medicine - Author Guidelines

Reporting Checklist for Life Science Articles (updated January
This checklist is adapted from Materials Design Analysis Reporting (MDAR) Checklist for Authors. MDAR establishes a minimum set of requirements in transparent reporting in the life sciences (see Statement of Task: 10.31222/osf.io/9sm4x). Please follow the journal's guidelines in preparing your
Please note that a copy of this checklist will be published alongside your article.

Abridged guidelines for figures
1. Data
The data shown in figures should satisfy the following conditions:
- the data were obtained and processed according to the field's best practice and are presented to reflect the results of the experiments in an accurate and unbiased manner.
- ideally, figure panels should include only measurements that are directly comparable to each other and obtained with the same assay.
- plots include clearly labeled error bars for independent experiments and sample sizes. Unless justified, error bars should not be shown for technical
- if n<5, the individual data points from each experiment should be plotted. Any statistical test employed should be justified.
- Source Data should be included to report the data underlying figures according to the guidelines set out in the authorship guidelines on Data

2. Captions
Each figure caption should contain the following information, for each panel where they are relevant:
- a specification of the experimental system investigated (eg cell line, species name).
- the assay(s) and method(s) used to carry out the reported observations and measurements.
- an explicit mention of the biological and chemical entity(ies) that are being measured.
- an explicit mention of the biological and chemical entity(ies) that are altered/varied/perturbed in a controlled manner.
- the exact sample size (n) for each experimental group/condition, given as a number, not a range;
- a description of the sample collection allowing the reader to understand whether the samples represent technical or biological replicates (including how many animals, litters, cultures, etc.).
- a statement of how many times the experiment shown was independently replicated in the laboratory.
- definitions of statistical methods and measures:
  - common tests, such as t-test (please specify whether paired vs. unpaired), simple χ2 tests, Wilcoxon and Mann-Whitney tests, can be unambiguously identified by name only, but more complex techniques should be described in the methods section;
  - are tests one-sided or two-sided?
  - are there adjustments for multiple comparisons?
  - exact statistical test results, e.g., P values = x but not P values < x;
  - definition of 'center values' as median or average;
  - definition of error bars as s.d. or s.e.m.

Please complete ALL of the questions below.
Select "Not Applicable" only when the requested information is not relevant for your study.

Materials

Newly Created Materials	Information included in the manuscript?	In which section is the information available? (Reagents and Tools Table, Materials and Methods, Figures, Data Availability Section)
New materials and reagents need to be available; do any restrictions apply?	Yes	Reagents and Tools Table; Methods

Antibodies	Information included in the manuscript?	In which section is the information available? (Reagents and Tools Table, Materials and Methods, Figures, Data Availability Section)
For **antibodies** provide the following information:<br>- Commercial antibodies: RRID (if possible) or supplier name, catalogue number and or/clone number<br>- Non-commercial: RRID or citation	Yes	Reagents and Tools Table; Methods; Figure legends

DNA and RNA sequences	Information included in the manuscript?	In which section is the information available? (Reagents and Tools Table, Materials and Methods, Figures, Data Availability Section)
**Short novel DNA or RNA including primers, probes**: provide the sequences.	Yes	Reagents and Tools Table

Cell materials	Information included in the manuscript?	In which section is the information available? (Reagents and Tools Table, Materials and Methods, Figures, Data Availability Section)
**Cell lines:** Provide species information, strain. Provide accession number in repository **OR** supplier name, catalog number, clone number, and/**OR** RRID.	Yes	Reagents and Tools Table; Methods
**Primary cultures:** Provide species, strain, sex of origin, genetic modification status.	Not Applicable	
Report if the cell lines were recently **authenticated** (e.g., by STR profiling) and tested for mycoplasma contamination.	Yes	Methods

Experimental animals	Information included in the manuscript?	In which section is the information available? (Reagents and Tools Table, Materials and Methods, Figures, Data Availability Section)
**Laboratory animals or Model organisms:** Provide species, strain, sex, age, genetic modification status. Provide accession number in repository **OR** supplier name, catalog number, clone number, **OR** RRID.	Yes	Reagents and Tools Table; Methods; Figure legends
**Animal observed in or captured from the field:** Provide species, sex, and age where possible.	Not Applicable	
Please detail **housing and husbandry conditions**.	Yes	Methods

Plants and microbes	Information included in the manuscript?	In which section is the information available? (Reagents and Tools Table, Materials and Methods, Figures, Data Availability Section)
**Plants:** provide species and strain, ecotype and cultivar where relevant, unique accession number if available, and source (including location for collected wild specimens).	Not Applicable	
**Microbes:** provide species and strain, unique accession number if available, and source.	Yes	Reagents and Tools Table; Methods

Human research participants	Information included in the manuscript?	In which section is the information available? (Reagents and Tools Table, Materials and Methods, Figures, Data Availability Section)
If collected and within the bounds of privacy constraints report on age, sex and gender or ethnicity for all study participants.	Not Applicable	

Core facilities	Information included in the manuscript?	In which section is the information available? (Reagents and Tools Table, Materials and Methods, Figures, Data Availability Section)
If your work benefited from core facilities, was their service mentioned in the acknowledgments section?	Yes	Acknowledgements

Design

Study protocol	Information included in the manuscript?	In which section is the information available? (Reagents and Tools Table, Materials and Methods, Figures, Data Availability Section)
If study protocol has been **pre-registered, provide DOI in the manuscript**. For clinical trials, provide the trial registration number **OR** cite DOI.	Not Applicable	
Report the **clinical trial registration number** (at ClinicalTrials.gov or equivalent), where applicable.	Not Applicable	

Laboratory protocol	Information included in the manuscript?	In which section is the information available? (Reagents and Tools Table, Materials and Methods, Figures, Data Availability Section)
Provide DOI OR other citation details if **external detailed step-by-step protocols** are available.	Not Applicable	

Experimental study design and statistics	Information included in the manuscript?	In which section is the information available? (Reagents and Tools Table, Materials and Methods, Figures, Data Availability Section)
Include a statement about **sample size** estimate even if no statistical methods were used.	Yes	Methods; Figures
Were any steps taken to minimize the effects of subjective bias when allocating animals/samples to treatment (e.g. **randomization procedure**)? If yes, have they been described?	Yes	Methods
Include a statement about **blinding** even if no blinding was done.	Not Applicable	
Describe **inclusion/exclusion criteria** if samples or animals were excluded from the analysis. Were the criteria pre-established?<br><br>If sample or data points were omitted from analysis, report if this was due to attrition or intentional exclusion and provide justification.	Not Applicable	
For every figure, are **statistical tests** justified as appropriate? Do the data meet the assumptions of the tests (e.g., normal distribution)? Describe any methods used to assess it. Is there an estimate of variation within each group of data? Is the variance similar between the groups that are being statistically compared?	Yes	Methods; Figure legends

Sample definition and in-laboratory replication	Information included in the manuscript?	In which section is the information available? (Reagents and Tools Table, Materials and Methods, Figures, Data Availability Section)
In the figure legends: state number of times the experiment was **replicated** in laboratory.	Yes	Methods; Figure legends
In the figure legends: define whether data describe **technical or biological replicates**.	Yes	Methods; Figure legends

Ethics

Ethics	Information included in the manuscript?	In which section is the information available? (Reagents and Tools Table, Materials and Methods, Figures, Data Availability Section)
Studies involving **human participants**: State details of **authority granting ethics approval** (IRB or equivalent committee(s), provide reference number for approval.	Not Applicable	
Studies involving **human participants**: Include a statement confirming that **informed consent** was obtained from all subjects and that the experiments conformed to the principles set out in the WMA Declaration of Helsinki and the Department of Health and Human Services Belmont Report.	Not Applicable	
Studies involving **human participants: For publication of patient photos**, include a statement confirming that consent to publish was obtained.	Not Applicable	
Studies involving experimental **animals**: State details of **authority granting ethics approval** (IRB or equivalent committee(s), provide reference number for approval. Include a statement of compliance with ethical regulations.	Yes	Ethics statement
Studies involving **specimen and field samples:** State if relevant **permits** obtained, provide details of authority approving study; if none were required, explain why.	Not Applicable	

Dual Use Research of Concern (DURC)	Information included in the manuscript?	In which section is the information available? (Reagents and Tools Table, Materials and Methods, Figures, Data Availability Section)
Could your study fall under dual use research restrictions? Please check biosecurity documents and list of **select agents and toxins** (CDC): https://www.selectagents.gov/sat/list.htm	Not Applicable	
If you used a select agent, is the security level of the lab appropriate and reported in the manuscript?	Not Applicable	
If a study is subject to dual use research of concern regulations, is the name of the **authority granting approval and reference number** for the regulatory approval provided in the manuscript?	Not Applicable	

Reporting

The MDAR framework recommends adoption of discipline-specific guidelines, established and endorsed through community initiatives. Journals have their own policy about requiring specific guidelines and recommendations to complement MDAR.

Adherence to community standards	Information included in the manuscript?	In which section is the information available? (Reagents and Tools Table, Materials and Methods, Figures, Data Availability Section)
State if relevant guidelines or checklists (e.g., **ICMJE, MIBBI, ARRIVE, PRISMA**) have been followed or provided.	Not Applicable	
For **tumor marker prognostic studies,** we recommend that you follow the **REMARK** reporting guidelines (see link list at top right). See author guidelines, under 'Reporting Guidelines'. Please confirm you have followed these guidelines.	Not Applicable	
For **phase II and III randomized controlled trials**, please refer to the **CONSORT** flow diagram (see link list at top right) and submit the CONSORT checklist (see link list at top right) with your submission. See author guidelines, under 'Reporting Guidelines'. Please confirm you have submitted this list.	Not Applicable	

Data Availability

Data availability	Information included in the manuscript?	In which section is the information available? (Reagents and Tools Table, Materials and Methods, Figures, Data Availability Section)
Have **primary datasets** been deposited according to the journal's guidelines (see 'Data Deposition' section) and the respective accession numbers provided in the Data Availability Section?	Not Applicable	
Were **human clinical and genomic datasets** deposited in a public access-controlled repository in accordance to ethical obligations to the patients and to the applicable consent agreement?	Not Applicable	
Are **computational models** that are central and integral to a study available without restrictions in a machine-readable form? Were the relevant accession numbers or links provided?	Not Applicable	
If publicly available data were reused, provide the respective **data citations in the reference list**.	Not Applicable	
